# The 330 risk loci known for systemic lupus erythematosus (SLE): a review

**DOI:** 10.3389/flupu.2024.1398035

**Published:** 2024-05-23

**Authors:** Viktoryia Laurynenka, John B. Harley

**Affiliations:** 1 US Department of Veterans Affairs Medical Center, Research Service, Cincinnati, OH, United States; 2 Division of Human Genetics, Cincinnati Children’s Hospital Medical Center, Center for Autoimmune Genomics and Etiology (CAGE), Cincinnati, OH, United States; 3 Cincinnati Education and Research for Veterans Foundation (CERVF), Cincinnati, OH, United States

**Keywords:** systemic lupus erythematosus (SLE), lupus, genetic variant, genome-wide association study (GWAS), ancestry, gene, pathway, review

## Abstract

An in-depth literature review of up to 2023 reveals 330 risk loci found by genetic association at *p* ≤ 5 × 10^−8^, with systemic lupus erythematosus (SLE) in at least one study of 160 pertinent publications. There are 225 loci found in East Asian (EAS), 106 in European (EU), 11 in African-American (AA), 18 Mixed American (MA), and 1 in Egyptian ancestries. Unexpectedly, most of these associations are found to date at *p* ≤ 5 × 10^−8^ in a single ancestry. However, the EAS and EU share 40 risk loci that are independently established. The great majority of the identified loci [250 (75.8%) of 330] do not contain a variant that changes an amino acid sequence. Meanwhile, most overlap with known regulatory elements in the genome [266 (80.6%) of 330], suggesting a major role for gene regulation in the genetic mechanisms of SLE. To evaluate the pathways altered by SLE-associated variants, we generated gene sets potentially regulated by SLE loci that consist of the nearest genes, published attributions, and genes predicted by computational tools. The most useful insights, at present, suggest that SLE genetic mechanisms involve (1) the regulation of both adaptive and innate immune responses including immune cell activation and differentiation; (2) the regulation of production and response to cytokines, including type I interferon; (3) apoptosis; (4) the sensing and removal of immune complexes and apoptotic particles; and (5) immune response to infections, including Epstein–Barr Virus, and symbiont microorganisms. These mechanisms affected by SLE genes involve multiple cell types, including B cells/plasma cells, T cells, dendritic cells, monocytes/macrophages, natural killer cells, neutrophils, and endothelial cells. The genetics of SLE from GWAS data reveal an incredibly complex profusion of interrelated molecular processes and interacting cells participating in SLE pathogenesis, mostly unified in the molecular regulation of inflammatory responses. These genetic associations in lupus and affected molecular pathways not only give us an understanding of the disease pathogenesis but may also help in drug discoveries for SLE treatment.

## Introduction

Systemic lupus erythematosus (SLE or lupus, OMIM: 152700), a potentially fatal systemic autoimmune disorder predominantly affecting young and middle-aged women, remains largely idiopathic and responds to immunosuppressive therapies. Particular features, such as complement activation, interferon (IFN) induction, apoptosis, and infection, especially by Epstein–Barr virus (EBV), are important components of the disease’s pathogenetic mechanisms (1, 2). Understanding how these different components combine to the development of the disease in patients who lack single-gene variants that strongly predispose to SLE (2) remains a challenging mystery.

A comprehensive understanding of SLE is not possible without robust explanations of how the variation in human genetics is incorporated into the mechanisms that lead to pathogenesis. At this juncture, assimilating the risk loci for the overall risk of SLE is reasonable, given the enormous effort made and the abundance of results now available in the literature. We remain at the beginning of this task, given that many of the mechanisms operating remain poorly defined. In addition, assembling genetics of the extraordinary heterogeneity of clinical SLE expression, diagnostic variation, and therapeutic responses will be tasks awaiting the work of future generations, since only a few of these genetic effects have been defined.

What we currently understand is probably only a minor part of the many different explanations needed to understand the mechanisms leading to SLE across the affected population. Genome-wide association studies (GWASs) and candidate gene association studies reveal variants in the human population that influence processes that change disease risk. These approaches have now been applied to the investigation of more than 5,000 traits (3).

The application of genetic association with SLE began in 1971 with the discovery of HLA (4, 5) and now carries through the genome-wide association study (GWAS) era to the present with hundreds of genetic loci discovered, many of which have been convincingly confirmed. To develop a better overall understanding of SLE genetics, we conducted an extensive review of the world’s literature on genetic association to understand what we now have in hand, in aggregate. At the beginning of 2023, we found 330 published genetic risk loci that satisfy the stringent requirement of association at *p* ≤ 5 × 10^−^8 in at least one study. However, the distribution by ancestry shows marked unfortunate differences in the extent to which discovery has been achieved to date. Nevertheless, the genes involved provide deep insight into the pathways and molecular relationships underlying the destructive autoimmunity characterizing this disease.

## Methods

### Literature review

We explored the published biomedical literature available on the PubMed website sponsored by the National Library of Medicine (https://pubmed.ncbi.nlm.nih.gov/) and the GWAS catalog (https://www.ebi.ac.uk/gwas/) for association studies that established genetic association at *p* < 5 × 10^−8^ after quality control measures were applied including filtering to remove technical artifacts and cryptic associations between cases and controls that were not related to SLE. We used various search combinations such as “systemic lupus erythematosus,” “SLE,” “lupus,” “genetic association,” “GWAS,” “genome-wide association study,” and “genetics.” We then searched through the references and citations for publications containing genetic associations that may have been missed. We included publications that (1) reported associations of a candidate gene study or GWAS with probabilities against chance to be spurious at *p* < 5 × 10^−8^; (2) compared SLE cases to healthy controls; and (3) presented original contributions to the literature (meta-analyses were accepted if they contained contributing results, but not strictly review articles). After reviewing >1,204 articles, we found 160 publications that reported a qualifying genetic association with SLE (Supplementary Material Table S1). We extracted information from each study, such as presumed ancestry, the most closely associated marker at each locus (“lead variant”) and its probability (OR), *p*-value, the risk and non-risk alleles, and the candidate genes that are supported by functional association (gene expression association, 3D interaction, experimental confirmation, computational modeling, and others; see Supplementary Material Table S1 for more details).

### SLE risk loci

An independent locus for our purposes herein was defined via the marker with the least probability of occurring by chance, determined by probability (*p*-value). To tag a locus, such markers must have a probability of <5 × 10^−8^ in any of the 160 studies. Neighboring associations with *p* < 5 × 10^−8^ were distinguished as separate loci if they were not in linkage disequilibrium (LD) at *r*^2^ < 0.2 or if there was evidence of independence reported in the literature based on regression analysis (logistic regression or a gene-based association analysis). The LD was defined for each variant pair in all reported ancestries using LDlink from NIH (6) and/or HaploReg v4.2 (7).

#### Disequilibrium expansion

The variant showing the closest association is often not a causative variant. These locus tagging variants cannot be statistically distinguished from their nearer neighboring variants that tend to travel with them from generation to generation. Therefore, we expanded the group of candidate causal variants by including all local variants with an association with the lead variant at *r*^2^ ≥ 0.8 using PLINK (v1.90b3.44) (8) or HaploReg v4.2 (7) if the variant was missing in PLINK. This disequilibrium expansion was performed in reported ancestries and in all ancestries [EUR, African-American (AA), East Asian (EAS), Mixed American (MA)] for variants from transancestral (TA) studies. Global and ancestry minor allele frequency (MAF), variant genomic position, and variant annotation were determined using Ensembl and BioMart tools (9).

Approximately 90% of loci in complex genetic diseases are located in intergenic regions of the genome (10). We constructed two lists of possible candidate genes through which genetic mechanisms are mediated. First, for each variant, we included the nearest neighbor as the default and then added the causal candidates identified from the literature in Group 1. From the 760 significant (*p* < 5 × 10^−8^) published variants, we accepted the most proximal gene (protein and non-coding RNA) identified using Ensembl (9) or the UCSC Genome Browser https://genome.ucsc.edu/ (11). Estimates vary with some concluding that about half (10) of the nearest genes are influenced by locus variants that do not alter amino acid sequence or slice junctions. Therefore, this method accommodates an unknown but probably high level of misattribution. Second, the top three genes associated with variants identified by the Open Target Genetics tool (12) were included in Group 2 from each of the 760 variants. Together, Groups 1 and 2 consist of 966 genes.

To evaluate the candidate genes, we used the Enrichr (13) analytical approach. We also independently evaluated gene lists with Reactome (14), GO by PANTHER (15), and ToppGene (16), which contain databases mostly incorporated in Enrichr and also provided identical results to the Enrichr analysis (data not included). From the 218 datasets integrated in the Enrichr analysis, we selected 86 datasets that were related to and had a clear description of pathways, ontologies, cell types, diseases, and drugs. Among these, 75 datasets showed results with a false discovery rate of *p* < 0.05. The results with *p* > 0.05 after correcting for multiple comparisons within the database were not further considered. We grouped the results from various databases in KEGG into pathways, cell types, diseases, therapeutics, and others (Supplementary Material Table S7).

## Results

### The 330 putative SLE risk loci

The current era of lupus genetic discovery by GWAS began with the mapping and detailed DNA sequence of the human genome (17). Closely following, the technology to genotype rapidly hundreds of thousands of variants simultaneously was developed, which led to the near synchronous publication of four studies of genome-wide SLE genetics in 2008 (18-21). We assembled the publications that present genetic associations with SLE at *p* < 5 × 10^−8^, which is the generally accepted threshold for probable genome-wide significance of genetic association. There are 160 such genetic association studies of SLE published before 2023 that purport to establish genetic associations with SLE at this level of significance. We relied on peer review to enforce community standards for analytical methods applied to avoid artifacts. The loci identified in these 160 publications are from GWAS projects, candidate gene studies, and meta-analyses, all contributing significantly to our current perception of SLE association genetics. In aggregate, these results provide insights into the genetic architecture of SLE, a perspective from which to understand the pathophysiology of SLE, and a foundation from which to explore genetic mechanisms that operate to generate SLE.

**FIGURE 1:**
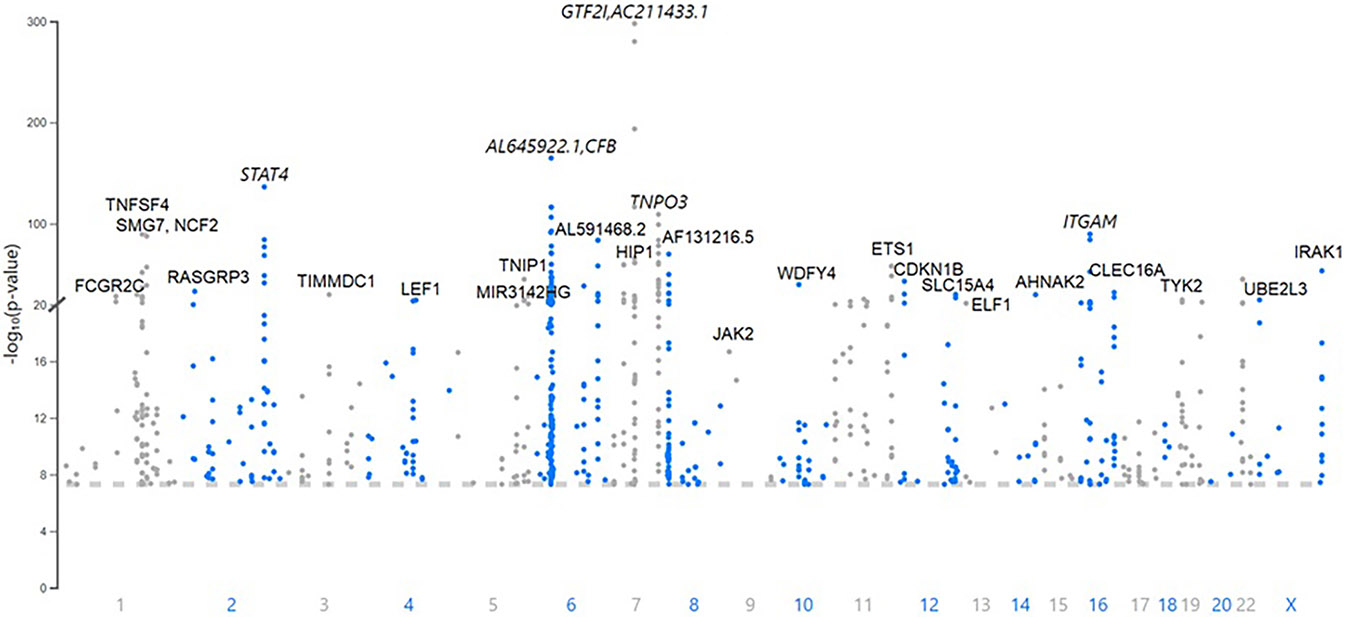
A Manhattan plot of the 760 variants published as associations with systemic lupus erythematosus (SLE) at p <5× 10–8. The top loci with lead variants with the lowest probability of occurring by chance are labeled with the expressed gene closest to the lead variant. The genomic position is indicated with the published probability.

**FIGURE 2:**
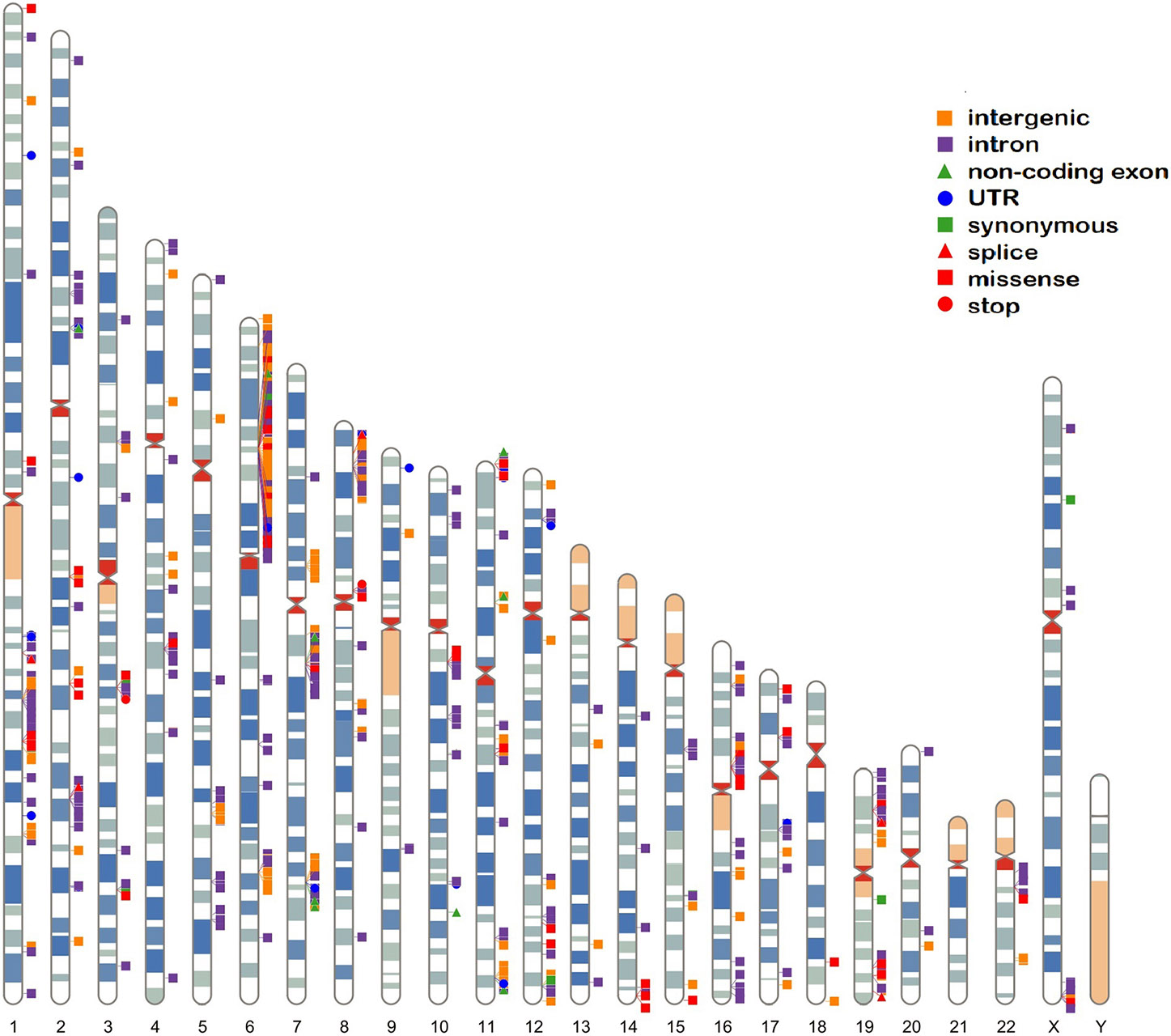
Chromosome plot showing the location and expected functional consequence of the 760 published systemic lupus erythematosus (SLE) risk variants. The HLA region on chromosome 6 contains 112 of the 760 variants [44 (13.3%) of the 330 risk loci], representing all of the possibilities given in the legend.

There are 760 published variants associated with SLE at *p* < 5 × 10^−8^ (Figures 1, 2 and Supplementary Material Table S1) in the 160 qualified publications. The patterns of association across the genome reveal many loci with multiple significantly associated variants (Figure 1). If disequilibrium was observed at *r*^2^ > 0.2 for any pair of variants, then they were aggregated into a single locus, unless there was published statistical evidence that they were independent. This resulted in 330 distinct loci (Table 1). We then performed an LD expansion of all literature-reported variants at each locus for each ancestry with an association with SLE at *p* < 5 × 10^−8^. We included all variants that were associated with literature-reported variants with *r*^2^ > 0.8. This led to a collection of 16,318 variants, a subset of which are anticipated to be causal variants for SLE (Supplementary Material Table S2). Consequently, seven genomic locations contain variants that are members of 2 neighboring loci, constituting 14 (4.2%) of the 330 SLE risk loci.

### SLE risk locus location in the genome

The published SLE susceptibility loci are distributed on the X chromosome and virtually all autosomes, except chromosome 21. The region with the highest locus density, with 44 (13.3%) of the 330 risk loci, is the 6p21 region containing the *HLA* genes and other immune-related genes, an unknown total number of which are involved in SLE pathogenesis (Figure 2, Supplementary Material Table S1). From human genome Build 38, these loci range from nucleotide 30,795,514 to 36,747,255 (or 30,507,590–36,755,012 for LD expanded variant set with *r*^2^ ≥ 0.8) in 6p21, covering ~6 Mb. Some of these loci overlap with each other on the chromosome and neighboring chromosome band 6p22.1, making this region even larger. The HLA region is the most gene-dense and polymorphic region in the genome with the highest complexity due to its dense LD, spanning very long regions of up to 0.54 Mb (71, 72). At this point and given this complexity, a comprehensive model of the genetic architecture of the genetic risk in the HLA region remains beyond a definitive solution.

### Salient results

The results with the lowest probability in the literature include the *NCF1* locus (OR = 2.1, *p* = 2.2 × 10^−298^); the *CFB* locus, which potentially regulates *C4A* expression (OR = 2.3, *p* = 2.3 × 10^−165^); a variant in the third intron of *STAT4* (OR =1.6, *p* = 5.9 × 10^−137^), which is equidistant between the translational start sites for *STAT4* and *STAT1*; and the *HLA-DRA* locus (OR = 1.6, *p* = 4.9 × 10^−117^) (refer to Table 1 for references to these results). There is evidence, for example, that the *STAT4* variant has a preferential influence on *STAT1* expression in B cells (73) with both *STAT4* and *STAT1* being regulated in monocytes (74).

The most consistently replicated loci beyond the HLA genetic association(s) are found near or in *STAT4, BLK, TNFAIP3, ITGAM, IRF5* and *TPNO3, PRDX6-AS1* and *TNFSF4*, and *CFB*, all of which have been found in >20 publications. Indeed, 77 genetic associations have been found in ≥3 publications (Supplementary Material Table S1). These associations are the most reliable of those now reported to be associated with SLE.

### Ancestry

**FIGURE 3:**
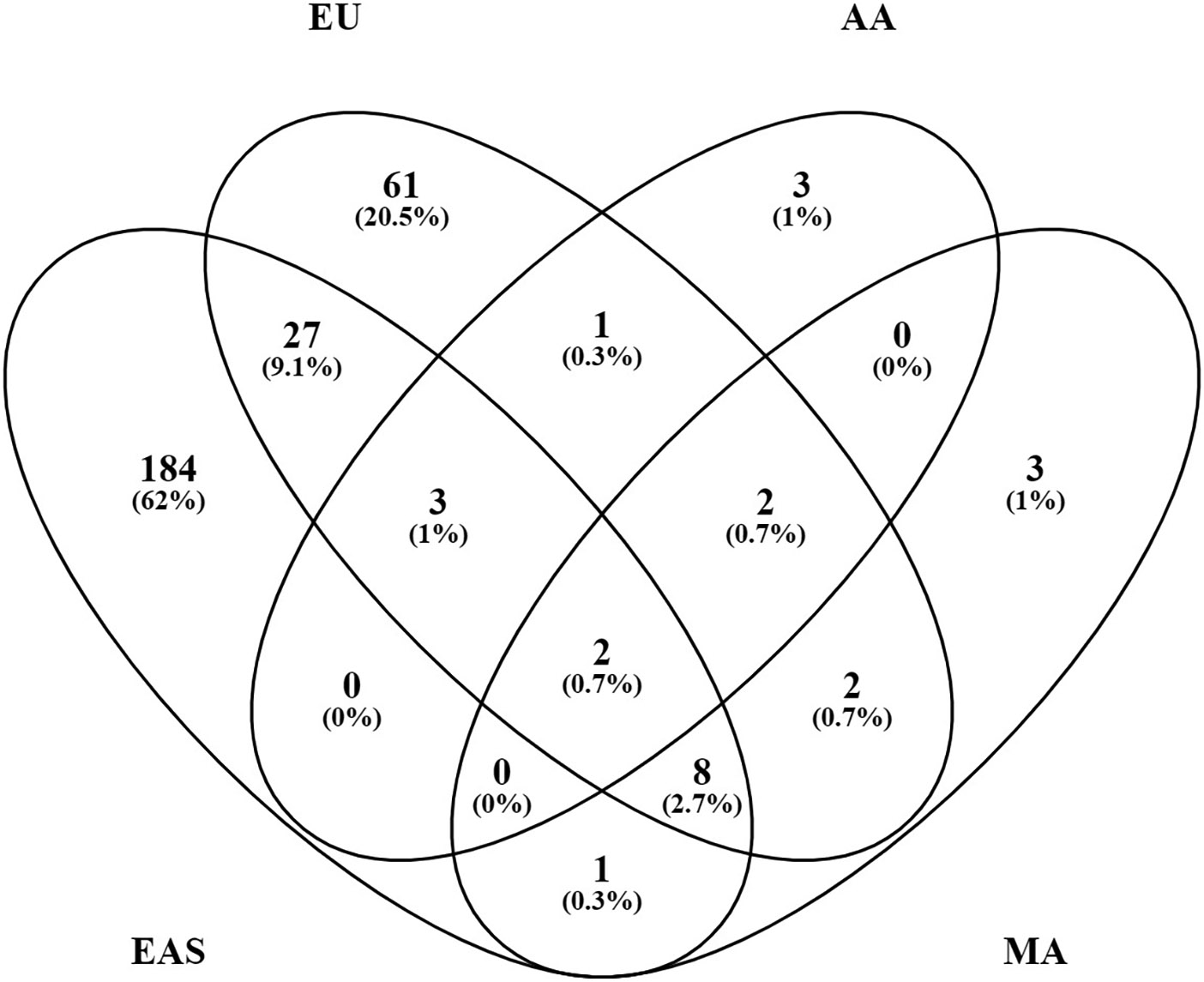
Systemic lupus erythematosus (SLE) loci overlap between ancestries. Of the 330 known loci, 297 can be assigned to a relatively defined population group or ancestry. The number of loci in each category is shown with the % of the total 297.

The majority of these genetic risk loci were discovered in EAS (225 loci) and European (EU) (106 loci) populations where large sample sizes have been studied. Other ancestries, where the sample sizes studied to date are far smaller, remain relatively unexplored: 11 loci in admixed AA ancestry; 18 loci in MA ancestry, a group which includes variously termed Latinos, Hispanics, Mestizos, and Native Americans or Amerindians; and 1 locus in Egyptian ancestry (Figure 3). There are 133 loci that have been established in TA cohorts composed of individuals from various ancestral origins from which the original authors made no ancestry assignment. There are no established SLE loci in African ancestry at this point. African-Americans are admixed with Europeans and various contributions from Native Americans and even EAS ancestry in different parts of the Americas.

Each population cohort and ancestry, however, has its own unique genetic history. Their ancestry-specific genetic architecture defines predisposition to SLE and affects disease manifestations, which may or may not involve similar genetic mechanisms. Indeed, most of the known loci, 252 (76%) of the 330, have only been established in a single ancestry. These include 184 (82%) of the 225 EAS loci, 61 (58%) of the 106 EU loci, 3 (27%) of the 11 AA loci, 3 (17%) of the 18 MA loci, and 1 Egyptian locus (Figure 3). Additionally, 32 (24%) of the 133 TA loci were discovered only in combined population cohorts. Of the 330 SLE risk loci, 46 (14%) are found in two or more ancestries, suggesting that susceptibility to SLE crosses ancestral barriers in humans, at least in part. Presumably, these consistencies originated with variation present before the present existing populations differentiated and not from convergent evolution. These results provide independent confirmation of the existence of these genetic associations.

Surprisingly, only 40 (14%) of 291 loci formed from combining the loci from EU and EAS ancestries, the two most extensively studied populations, are shared by both ancestries when requiring *p* < 5 × 10^−8^ for consideration (Figure 3). Perhaps, some of the results not yet confirmed are spurious. The majority of the ancestry-specific loci [166 (66%) of 252 loci] were reported in one study only and have not yet been confirmed at *p* < 5 × 10^−8^ in any other studies. Furthermore, 50 (20%) loci are significant in 2 studies and only 36 (14%) loci (14 in EU and 22 in EAS) were replicated in ≥3 studies. In addition to the artifacts created by cryptic systematic differences between cases and controls, there are additional problems in finding genetic associations in admixed samples (75, 76).

### Association magnitude

As is typical of complex disease association genetics, the usual effect size for reported lupus associated loci is small (Supplementary Material Table S1 and S2), ranging from an odds ratio (OR) of 1.09–1.5 for 267 (81%) of the 330 loci. Moderately larger effect sizes with 1.5 < OR < 3 has been observed in 52 (16%) of loci. Only nine (3%) SLE risk loci had an effect size of >3. Those in this highest category have only been reported in single studies (eight in EAS and one in MA) and are virtually all rarer variants with a MAF of <5%, except at rs933717 (64). The tendency to increase in effect size with a decrease in MAF, as found in other complex diseases (77), is also observed in these 330 SLE risk loci (Supplementary Material Table S1). The extent to which this relationship is the result of evolutionary pressures over time or improved statistical power to detect smaller differences as the MAF increases is not known. Rare variants depend on a relatively smaller number of cases, which makes the procedures for purging associations erroneously attributed to the phenotype especially important, raising the concern that a proportion of these may be artifacts.

### Distribution of allele frequency

The majority of loci [274 (83%) of the 330 leading variants] are represented by common variants with a MAF of >5%, where 50% (166 loci) belong to a category of variants with higher MAFs (>20%), while 33% (108 loci) have intermediate MAFs (5%–20%). The distribution of MAF of loci associated with complex traits is skewed toward higher MAFs rather than intermediate MAFs in comparison with the general human populations, where the fraction of SNPs with intermediate MAF categories is approximately 55.0%. This finding has been reported before for other non-SLE traits (77). The minor fraction of SLE loci is represented by rare and very rare variants: 30 (9%) loci with a MAF of 1% ≤ 5%, 23 (7%) loci with a MAF of 0.1% ≤ 1%, and 2 (1%) loci with a MAF of <0.1% (Supplementary Material Table S2. When considering loci based on their MAFs, 57% of the SLE risk variants are minor alleles (Table 1).

### Function derived from variant location

Genomic location is often an indicator of variant function. We compared the distribution of the functional predictions of the genetic variants with the published distribution of variants in the human genome (78) (Table 2). Overall, the associated variants are concentrated at transcribed genes more so than would be expected by chance at 67.5% for the lead variants vs. 42.3% for variants across the genome (OR = 2.82, *p* = 2.9 × 10^−20^). The DNA sequence variation predicts that amino acid changes are enriched by over 34-fold among the leading variants of 330 loci, over 28-fold in the 760 significant published variants, and over 4-fold in the 16,318 variants after LD expansion at *r*^2^ > 0.8 of the 760 significant variants. The frequency that these SNPs are found outside of genomic locations defined by genes is lower than expected. For the intergenic regions for all three categories of SNPs, the frequency ranges from 0.33- to 0.57-fold. Meanwhile, in all three categories of potentially associated SNPs, the most enriched non-synonymous amino acid changes (from 4.6- to 34-fold) are followed by synonymous coding changes (from 2.5- to 7.9-fold), which are roughly equivalent to the untranslated regions of the RNA (from 3.1- to 7.4-fold), followed by introns (from 1.6- to 1.9-fold) ( Table 2).

Among the 330 SLE risk loci, 26 (8%) have leading variants that change the amino acid sequence of protein products in ways that make such a change a strong candidate for genetic causality, including *AHNAK2, C1QTNF12, CD226, FCGR2A, HLA-DQB1, IFIH1, IKBKB, IRAK1, IRF3, IRF7, ITGAM, LRRK1, NCF2, NOTCH4, OAS1, PLAT, PLD2, PTPN22, TAOK3, TCP11, TSBP1, TYK2*, and *WDFY4* (Supplementary Material Table S1). These variants differ from the remaining 92% of the known SLE risk loci, whose mechanism is much more consistent with a gene regulatory mechanism rather than by altering gene product activity through a structural change, such as amino acid changes. Certainly, any potentially attributed function for DNA sequencing variants, including the codes that change the amino acid composition of proteins, remain only candidates for genetic mechanism in the absence of evidence establishing causation.

There are several examples where SLE-associated causal variants change the amino acid sequence and protein function and regulate gene expression simultaneously within one locus. For example, integrin alpha M (*ITGAM*; *CD11B*) is a component of the macrophage-1 antigen complex [Mac-1, or complement receptor 3 (CR3)] mainly expressed in neutrophils, monocytes, macrophages, and dendritic cells, where it mediates leukocyte adhesion, extravasation, migration, phagocytosis, complement activation, and inflammation. Missense polymorphism, rs1143679 (R77H), in *ITGAM* is in the most active region of chromatin regulation with enhancer activity and transcription factor binding [including XRCC5 (Ku70)/XRCC6 (Ku80), NFKB1 and EBF1], and SLE risk allele (“A”) correlates with lower RNA transcript and surface-displayed protein levels (from 10- to 15-fold reduction) and also leads to the significantly reduced binding of CD11b to fibrinogen, vitronectin, iC3b, DC-SIGN, ICAM-1, and ICAM-2; to the polarization of Mac-1 in the membrane instead of even distribution; and to the reduction of both phagocytosis and toll-like receptor 7/8 (TLR7/8)-induced cytokine release (62, 79, 80).

Another example is the *NCF2* locus at 1q25.3. Neutrophil cytosolic factor 2 (*NCF2*) encodes p67^*phox*^, a core component of the multi-protein NADPH oxidase complex that produces reactive oxygen species (ROS) such as superoxide ($O_{2}^{•-}$) and hydrogen peroxide (H_2_O_2_), which are required for pathogen clearance by phagocytosis in neutrophils, monocytes, and macrophages. Moreover, NADPH oxidase is a key player in the formation of neutrophil extracellular traps (NETs) secreted by neutrophils to entrap and kill microorganisms (81). In other immune cells (e.g., antigen-presenting cells), ROS production is much more limited, and NADPH oxidase activity regulates phagosomal pH and participates in antigen processing and presentation including cross-presentation (82, 83). Derivative ROS functions as signaling molecules in immune cells, participating in immunoregulation, including regulation of type I IFNs (82, 84).

The NADPH oxidase complex is activated by one or more soluble GTPases with the involvement of a specific guanine nucleotide exchange factor (GEF), such as Vav1. Vav1 directly interacts with NCF2 (85, 86). The variants at *NCF2* disrupt NADPH oxidase activity. Here, the likely causal missense variant rs17849502 (H389Q) of *NCF2* has been shown to adversely affect the binding between the p67*phox*-PB1 domain and Vav1 (85). The substitution of histidine-389 with glutamine (SLE risk allele) causes a twofold decrease in ROS production induced by the activation of the Vav-dependent Fcγ receptor-elicited NADPH oxidase activity (85). The NCF2 variants may disrupt ROS production which is often dysregulated in SLE patients, thus leading to the accumulation of NET debris and auto-antigenicity, altered profile of epitopes selected for presentation, and immune dysregulation (81-84, 87, 88).

Another likely causal variant in this locus is the synonymous variant rs17849501 (A202A) in exon 6 of NCF2 that is 9,793 bp downstream and is in strong LD (*r*^2^ = 1, *D*’ = 1) with the missense variant rs17849502 (H389Q) in exon 12. rs17849501 is located in a conserved transcriptional regulatory region with enhancer/silencer function affected by this SNP that has been confirmed in a luciferase expression assay. The risk allele rs17849501-A is associated with decreased expression of adjacent gene *SMG7* (30). *SMG7* encodes a protein that is essential for non-sense-mediated mRNA decay that degrades mRNAs with premature termination codons, preventing the production of truncated, deleterious proteins. Decreased *SMG7* expression may cause RNA–protein complex accumulation and was shown to be associated with increased antinuclear antibody (ANA) titers in SLE patients (89).

When the SLE risk loci are defined as all of the variants that are in disequilibrium at *r*^2^ > 0.8 (*n* = 16,318) with all of the published variants (*n* = 760) across all 330 of the SLE loci, then protein amino acid sequence changes are found in 24% (80 of 330) of the SLE loci (Supplementary Material Table S3). This finding is similar to those of the previous analyses reporting that 19% of SLE loci include a gene with an amino acid sequence change (90). In all of these cases, whether or not the amino acid sequence change is responsible for SLE risk remains unestablished.

Changes in splicing, which generate variations at the level of gene product isoforms, may affect the activity of the gene product. Heteronuclear RNA spicing sites are polymorphic affecting 1 of the 330 SLE lead variants and 6 of the 760 associated reported variants. An additional example is a splice variant found in the FAM86B3P, which is a pseudogene. There are several splice variants that may influence protein product isoform expression; for example, rs2004640 affects *IRF5* splicing (91, 92), potentially contributing to the complex genetic mechanisms operating in the *IRF5* association complex (93).

Some lupus risk variants located in regulatory RNA sequences may affect gene product function. Examples include variants in long non-coding RNAs (lncRNAs) that are also present in SLE risk loci: *PRDX6-AS1, DGUOK-AS1, ENSG00000289526,* and micro-RNA (miR) *MIR210HG* (aka, *miR210*) (Supplementary Material Table S1).

### Gene regulation

The vast majority of reported variants are found in non-coding regions (>90%) (Table 2, Supplementary Material Table S3). Since they do not directly change the structure of gene products, a regulatory role, especially of enhancers and suppressors, is the primary suspected genetic mechanism operating to alter the risk of developing SLE. Most loci contain variants in LD (*r*^2^ > 0.8) that overlap with known regulatory elements in the genome [266 of 330 (81%), Supplementary Material Table S3]. This is >7-fold higher than the distribution of variants at known regulatory elements in the average genome where only 10.5% of genomic variants overlap promoters, insulators, enhancers, or transcription factor binding sites (78) and consistent with a major role for gene regulation in the genetic mechanisms of SLE. Previous studies have also shown that SLE variants are enriched in transcription start sites and enhancers (22). Such variants may alter the binding of transcription factors and other regulatory molecules at these sites and also change the expression through DNA methylation and histone modification. There are multiple examples of potential gene regulation for hundreds of genes by SLE variants (Supplementary Material Table S1), including the regulation of expression of proteins [*STAT4, BLK, TNFAIP3, ITGAM, IRF5, TNFSF4, TNIP1* (aka, *ABIN1*), *WDFY4, UBE2L3, BANK1, ETS1,* and others] and regulatory RNAs [*miR146a*, lncRNAs *DGUOK-AS1, LINC02694* (*C15orf53*), and others].

Screening of thousands of candidates in LD with SLE-associated variants with massively parallel reporter assay (MPRA) discovered that 482 variants lay in regions with enhancer activity at least *in vivo*, of which 51 demonstrated genotype-dependent (allelic) enhancer activity at 27 risk loci (30% of the 91 tested loci) in lymphoblastoid cell line GM12878 (94). Moreover, SLE variants demonstrated the cell type specificity of allelic enhancer activity: 92 SLE risk variants in the T-cell line Jurkat had allelic activity. Only 25% of these variants were also found in GM12878 (94). In addition to this complexity, allelic behavior changed for some variants upon stimulation: In Jurkat cells stimulated with the inflammatory cytokine TNF-α, a key cytokine in SLE development, 102 SLE variants had allelic regulation properties, 28 of which were specific to the stimulated Jurkat cells. Altogether, this study identified 145 candidate causal variants with allelic behavior for 50 SLE risk loci (94).

In another more recent MPRA study, 17 variants (including six SLE index SNPs and 11 novel candidates) in the non-HLA region and 18 variants in the HLA region were identified as potential causal variants for SLE in an EBV-transformed B-cell line generated from an SLE case (95). However, the concordance between these two studies was only 60% (for 99 of 166 variants tested in both studies) (94, 95). A CRISPR-based genomic screen attempting to identify SLE risk variants important for the type I IFN pathway will hopefully enable the identification of probable causal alleles and genes (96). The preliminary data from this study show that candidate functional variants were associated with the expression of critical regulators within the JAK-STAT pathway and IFN-stimulated genes, which appear to usually act in a cell type-specific manner (96).

Gene expression is controlled by regulatory elements including enhancers, promoters, CTCF-occupied elements (silencers and insulators), and elements that alter chromatin structure and transcription factor accessibility including DNA methylation and histone modifications (e.g., acetylation, methylation, and phosphorylation).

Many transcription factors and cofactors, along with histone marks associated with active enhancer and transcription, such as H3K27ac and H3K4me1, have enriched ChIP-seq peaks at SLE risk loci binding to a majority of SLE loci and demonstrating allelic binding preference (allelic imbalance between ChIP-seq read counts for SLE risk and non-risk alleles) (22, 94, 97). SLE variants if they are located in DNA-binding sites can directly affect the binding of TFs or alter the binding of adjacent TFs or TFs made proximal by DNA looping (95, 97, 98).

The regulation of gene expression is very complicated and typically involves multiple regulatory elements that are often active differently across cell types. Certainly, SLE loci may involve only one regulatory causal variant, such as rs34330 at the *CDKN1B* locus, or, at this point, have many potential causal variants, perhaps having different consequences for the phenotype, as may be happening for IRF5 (Supplementary Material Table S1). In the latter case, an analysis focusing on the composition of haplotypes instead of individual variants will be a better model for the risk architecture in this region.

The tumor suppressor gene cyclin-dependent kinase (CDK) inhibitor 1B gene (*CDKN1B*) encodes an inhibitor of cyclin/CDK complexes that participate in many cellular events such as cell cycle arrest during the G1/S transition for repair DNA damage and replication errors; promotion of apoptosis by inhibiting p27 and RhoA; autophagy modulation and autoimmunity development; inhibition of the development of CD4+ T-cell effector function and proliferation of thymic and mature T cells; and promotion of T-cell anergy and immune tolerance. Dysregulated expression of *CDKN1B* is a frequent event in several human cancers and it also may contribute to cellular damage and SLE progression (60, 99, 100).

rs34330 in the 3′ UTR of *CDKN1B* is the only candidate variant for this locus (35, 60). None of the neighboring variants achieve a maximal disequilibrium of *r*^2^ > 0.6 in EAS. Many experiments (such as luciferase reporter assays, ChIP-qPCR, EMSA, Western blot, mass spectrometry, chromosome conformation capture 3C, and CRISPR-based genome editing) coalesce to support the idea that SLE risk allele rs34330-C provides a higher promoter and enhancer activity, with an increase of the histone modifications H3K27ac, H3K4me3, and H3K4me1 at that location (60). In addition, there is also increased binding of transcription factors RNA pol II and IFN regulatory factor 1 (IRF1). These changes lead to the increased expression of the neighboring genes, namely, *CDKN1B, DDX47*, and *GPR19*, and decreased expression of *APOLD1*. Gene editing in this region also leads to increased proliferation and apoptosis *in vitro* (60).

*DDX47* belongs to the DEAD-box RNA helicase protein family, which is involved in the alteration of RNA secondary structure, such as translation initiation, nuclear and mitochondrial splicing, ribosomal and spliceosomal assembly, and antiviral innate immunity. As previously reported, the dysregulation of antiviral helicases that normally function as sensors of cytosolic viral nucleic acids leads to the overactivity of the type I IFN pathway and may contribute to the development of SLE (101).

Apolipoprotein L domain-containing 1 (APOLD1) is an endothelial cell early response protein that regulates endothelial cell signaling, cell junctions, cytoskeletal architecture, and vascular function (102). The breakdown of vascular integrity is a key feature of numerous pathologies including SLE (103). Moreover, variants in *APOLD1* are potentially associated with an increased risk of advanced lupus nephritis (104). This locus is another example where a single locus (and sometimes a single polymorphism) regulates many genes that may independently contribute to SLE pathogenesis in different ways.

Certainly, we must bear in mind that mechanisms are only candidates for causation. In most cases, there is no direct evidence that they alter disease risk; consequently, an unknown proportion of the variants and their mechanisms now known are “bystanders” with no involvement in disease pathogenesis.

IFN regulatory factor 5 (*IRF5*), as mentioned earlier, is a transcription factor expressed in B cells, monocytes and macrophages, and dendritic cells. It plays a central role in signaling by toll-like receptors (TLRs) via the TLR–MYD88 pathway, thereby regulating the production of proinflammatory cytokines. IRF5 induces the production of type I IFNs; proinflammatory cytokines, such as interleukin (IL) 6, IL12, and IL23; and tumor necrosis factor-α (TNF-α). IRF5 is involved in the regulation of cell growth, differentiation, apoptosis, immune system activity, and response to viral infection and is a key factor in promoting the inflammatory macrophage phenotype (105-107).

Of the likely multiple *IRF5* loci, one has the leading variant rs4728142 with at least six functional variants in strong LD with each other that affect the enhancer regulation of *IRF5* expression (rs77571059, rs3778754, rs3807307, rs11269962, rs4728142). In addition, changes in IRF5 isoforms through differential splicing of *IRF5* mRNA from rs2004640 have been identified as potentially causal in multiple studies for this locus (91, 93, 108-110) (Supplementary Material Table S1). Adding further complexity to the association between *IRF5* and lupus, there are at least four relatively independent loci that regulate *IRF5* expression: three loci previously published (Table 1, Supplementary Material Table S1) and one locus not yet published discovered in an ongoing research involving African-Americans (K. Kaufman, personal communication). Note that another *IRF5* locus with leading variant rs41298401 also has at least three potential functional variants, namely, rs729302, rs12706860, and rs13245639 (110) (Supplementary Material Table S1). All these loci with multiple functional variants form different combinations and result in at least nine different haplotypes, containing risk/non-risk alleles, associated with different levels of *IRF5* expression (110-112).

Lupus risk loci for the majority of these IRF5 causal candidates are associated with increased total *IRF5* expression in blood and lymphoblastoid cell lines (22, 91, 93, 108, 110, 111, 113-119), except for rs729302-A where results are contradictory (110, 112). In other tissues, these same alleles may decrease *IRF5* expression. For example, in thymic tissue, SLE risk alleles at IRF5 were associated with lower *IRF5* expression (92). *IRF5* expression changes were in opposing directions when results for monocytes were compared with those for brain tissue (111, 119-121), which might be the result of other factors, such as changes in *IRF5* isoform representation.

The splicing of *IRF5* is highly complex and affected by SLE risk variants. The *IRF5* gene contains nine exons and produces between 11 and 17 isoforms (91, 122). Exon 1 encodes the 5′ untranslated region (5′ UTR) and has four alternate start sites: exons 1A, 1B, 1C, and 1D with four alternative promoters containing putative binding sites for different transcription factors. In addition, these promotors respond distinctly to stimuli (123). rs2004640, associated with SLE, is located 2 bp downstream of the intron–exon border of exon 1B, creating a consensus GT donor splice site. Exon 1B is expressed only in individuals having rs2004640-T (91, 92). Although exon 1 is non-coding and does not affect protein sequence, it influences translation efficiency. Exon 1A transcripts were expressed at higher levels and were more efficient in initiating protein synthesis compared with the other exon 1 transcripts in both blood cells left unstimulated and those stimulated with IFN-α (113). In lupus patients, *IRF5* expression and alternative splicing with the production of new isoforms with unknown biological function in peripheral blood mononuclear cells were significantly upregulated compared with healthy donors (111).

Thus, the regulatory disruption of *IRF5* expression in lupus is highly complex. There are many potential functional variants, providing alternative genetic mechanisms competing on the various haplotypes, some splicing-dependent, some stimulation-dependent, and others cell type-dependent. These observations lead to the conclusion that this locus is still poorly understood, despite the major efforts of many scientific groups.

*IRF5* is an example of regulating the regulators. There are many more examples where lupus loci affect the structure or expression of transcription regulators including transcription factors and cofactors or non-coding regulatory RNA that control the expression of many other genes. These transcription regulators (transcription factors and cofactors) may act at the level of transcription or translation (i.e., RNA-binding regulatory proteins), interact with DNA/RNA directly via transcription factors or indirectly through cofactors participating in the formation of regulatory complexes, and act as activators or repressors. Moreover, some SLE-associated genes that bind to DNA participate in the regulation of DNA replication or DNA repair.

There are 966 genes associated with the 330 loci (combined from Groups 1 and 2, see Methods); however, of these, only 291 genes are shared between Groups 1 and 2. The pathways that appeared to be significantly associated in the two groups were almost identical. Both the redundancy between the groups (in SLE multiple genes involved in one pathway are affected) and the independent tendency to reveal shared processes from the two approaches probably account for the high level of similarity in both groups.

The classification system used in the Human Transcription Factor Database captures 78 of these TFs (124). In addition, we used three other databases focusing on transcriptional regulators or DNA-interacting proteins, along with the TFs evaluated in 18,076 ChIP-seq experiments (RELI database, unpublished). This process identified 103 transcriptional regulators and DNA-interacting proteins in the groups of candidates for causation in SLE. A few examples include *BCL6, GATA4, IKZF1, IRF1, IRF3, IRF4, IRF5, MECP2, ELF1, RELA, STAT1, STAT4*, and *TCF7* (Supplementary Material Table S5). Proteins that regulate other genes have the potential to change the expression of thousands of regulated downstream genes, a subset of unknown proportional size that influences SLE risk.

In addition to the observation that one SLE locus can regulate several genes, we can see that one gene may be regulated by several SLE loci. There are around 220 (22.7%) genes, including the *IRF5* gene mentioned earlier, regulated by two or more loci (Supplementary Material Table S1, Supplementary Material Figure S2, Supplementary Material Table S4). The following group of expressed protein is potentially regulated by 6–11 independent SLE risk loci: *GTF2I, HLA-DQA1, HLA-DRA, HLA-DRB5, GTF2IRD1, HLA-DQB1, NCF1, HLA-C, HLA-DRB1, HLA-DQA2, FDFT1, IRF8, MICA, MICB, NCF2, RASGRP1,* and *TYK2*. The complexity of the risk architecture is so high that one imagines that the entire organism contributes to the risk assessment, analogous to the “omnigenic” model where all genes are involved in the genetic risk in complex diseases (125).

#### Reproducibility

Among the 330 SLE loci, 197 (59.7%) were reported in only one publication, 56 (16%) loci have been found in only two studies, and 77 (23.3%) loci have been established at *p* ≤ 5 × 10^−8^ in three or more studies of presumably independently ascertained cases and controls. Replication and confirmation of association are requirements of the scientific method, while a single instance of a small probability is not. While the 5 × 10^−8^ has proven to be a generally reliable threshold for results that are usually replicated, this is not the universal experience and may depend on the size of the study cohort, MAF, population, and genotyping approach (126-128).

**FIGURE 4:**
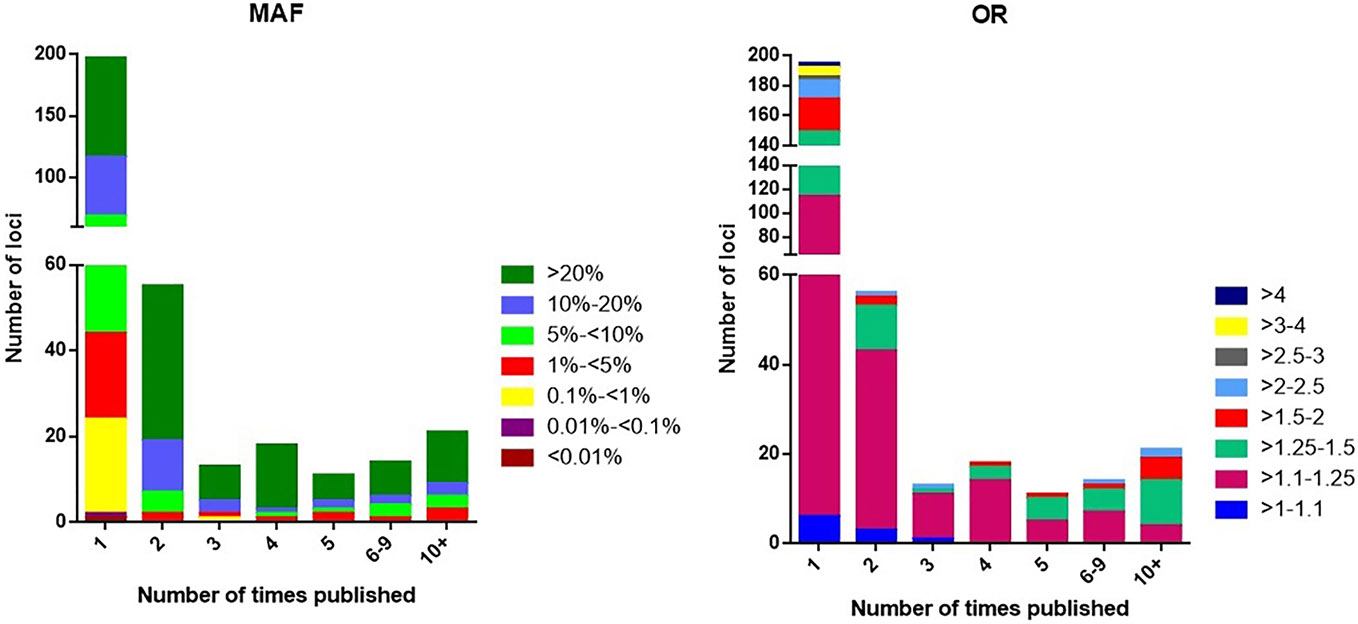
The distribution of loci according to minor allele frequency (MAF) or odds ratio (OR) and the number of times the locus has been published with p < 5 X10–8.

The 197 loci that have not yet been confirmed in other studies remain candidates that are highly probable to be associated, although some of these may be false-positive results. We note that 75% of the single report loci have MAF < 5%. The distribution is also altered for rare variants (<1%), which tend to have either smaller effect sizes (OR < 1.25) or larger effect sizes (OR > 2.5) (Figure 4). The absence of confirmation of the single report loci may have one of the following explanations:

1. A limited number of studies in AA, MA, and Egyptian populations. Only 9 and 13 studies reported associations with *p* < 5 × 10^−8^ in AA and MA, respectively. Only 3 of 11 loci in AA and 9 of 18 loci in MA were replicated. Elghzaly et al. (38) conducted the first and only study in Egyptians .
2. Small sample sizes for some studies. The recent largest study done by Yin et al. in 2021 (26) in EAS with >200,000 participants identified 88 new loci that account for almost half of 197 loci reported one time. Most of these loci are expected to be verified by the next generation of large GWASs; however, there is no guarantee that publications are using only independently ascertained subjects, which has the potential to compromise meta-analyses and other efforts to confirm findings.
3. Cryptic systemic differences may not be removed by the usual principal component analysis, which is a particularly serious problem in samples with admixed ancestries (75, 76).
4. Study ascertainment differences may accentuate the heterogeneity intrinsic to lupus in ways that differentially concentrate the genetics important for subsets with respect to clinical findings or sex. An example is the rare variant rs529561493 (MAF = 0.0004) at 1q32.1 in *RASSF5* that was associated (OR = 3.66) with SLE in SLE patients with steroid-associated osteonecrosis of the femoral head compared with healthy controls but not reported in other studies that included patients with more broad SLE spectrum (32). Some studies (20) include only females while most include both sexes.
5. Methodologic differences in genotyping, imputation, or analysis. The different genotyping platforms lead to differences in the imputation error for individual variants. Rare variants (<1%) are often excluded from the analysis, particularly in small studies, leading to poor replication of association results for rare variants.

### Artifacts

Some works in the 160 publications presenting genetic associations with SLE are likely to contain some spurious false-positive results. rs933717 at 16q24.2 is an example of an inconsistent result. Interestingly, the rs933717-C allele has the highest OR at 7.7, calculated from the cases having the rs933717-C allele in 89% of cases and 13% of controls (64). Meanwhile, the 1000 Genomes Project Phase 3 (78) presents this allele in their sample of Asians at 97.3%, thereby suggesting that technical issues are a possible source of an artifact. This allele is found in approximately 44% of Europeans where no association with SLE genetic risk has yet been reported.

Furthermore, some results have not yet been replicated even within the same study. For example, rs2714333 (*RREB1*), which is significant in a sample of Japanese with an alleged OR = 3.11 (*p* = 10^−08^), did not show even a trend in the same direction from a meta-analysis of a larger EAS sample set (*p* = 0.18) (32). The examples of rs933717 and rs2714333 appear to be potentially spurious putative associations with SLE, highlighting the caution needed for alleged association results with large effect sizes (OR > 3) reported in a single study.

### Missing data: large structural variation (copy number variants, large InDels, transposons, etc.)

The great majority of reported variants associated with lupus (95%) are represented by single-nucleotide polymorphisms (SNPs). A few variants (5%) are small deletions or insertions, and only one established locus at *p* < 5 × 10^−8^ (*FCGR3B*) is a copy number variant (CNV). CNVs are polymorphisms that arise when the number of copies of a specific segment of DNA varies among human chromosomes. Even though we now have 330 loci, the published genetic association literature largely ignores the probably major impact that the 20 kb complement C4 gene (C4) repeats at 6p21.33 in the HLA region have upon overall SLE risk, because this variation has not yet been established in association studies to reach *p* < 5 × 10^−8^ (129). C4 is also associated with monogenic lupus (2). The omission of this very important CNV in GWAS studies certainly contributes to the present difficulty in constructing a clear, nearly complete, robust model of the HLA genetic association architecture.

There is also a technical bias toward identifying physically smaller variants as a consequence of the genotyping methods currently used. The detection of the larger variants is more technically complicated, making their measurement in large samples prohibitively expensive. The impact of these relatively inaccessible variants on SLE risk at the scale of the whole genome in larger cohorts remains unknown. This probable deficiency of the available results seems likely since some large variants are widespread in the human genome. When these are located in critical coding or regulatory regions, they have the potential to have large effect sizes, such as the *C4* repeat with OR = 5.7, as mentioned above (129). At least 10% of the human genome is composed of CNVs. Some of them repeat several megabases of DNA and contain many genes, estimated to be responsible for 18% of inter-individual heterogeneity in protein expression (130, 131). Moreover, in general, approximately 20% of the detected CNVs intersect SNP associations. Thus, large genomic variations represent an as-yet largely unexplored opportunity to identify important new SLE loci and to improve our model of SLE genetic risk.

A low copy number, that is, <2 copies, of the Fc receptor gene *FCGR3B* (1q23.3 locus), for example, is reported to be associated with SLE (OR =1.8) (27, 132). *FCGR3B* is a low-affinity IgG receptor, expressed mostly on neutrophils. The CNV here influences the expression level of *FCGR3B*. Low expression correlates with reduced adherence to and uptake of immune complexes in neutrophils (133).

The impact of other large variants such as large insertions, deletions, and inversions on SLE predisposition remains unexplored. Transposable elements represent a promising category of variation that provides hints for mechanisms that influence SLE risk. At least 18 of the SLE risk loci are in LD at *r*^2^ > 0.8, with transposable elements published previously (134), 4 of which are also known to be located in enhancers (Supplementary Material Table S1).

### Ancestry-specific loci

If we limit our attention to the most convincing SLE loci, which are the 77 loci that have been found in ≥3 studies, the interpretation of the ancestry-specific differences is more likely to be reliable. While we will be ignoring results that have yet to be confirmed, we will have more confidence in the general principles derived. In addition, consideration of the 77 loci is limited to the EAS and EU results, where we have sufficiently large sample sizes.

Of the 77 loci, 36 (46%) superficially appear to be potentially ancestry-specific (Table 3). The MAFs for these 36 loci cover a wide range of MAFs in these two populations: 4 (11%) of the 36 markers are not polymorphic in the second population; 3 (8%) are polymorphic but have >100-fold MAF difference; 4 had an 11- to 100-fold MAF difference; 16 (44%) had a 1.5- to 10-fold difference; and 9 (25%) had a >1- to 1.5-fold difference (Table 3). From the perspective of the 1000 Genomes Project (78), a MAF at >5% in one population, but <0.5% in the human species overall, is uncommon at ~0.9%. Compared to this expectation, the frequency of leading variants at these 77 SLE loci with MAF differences between populations appears to occur at a higher rate than would be expected.

Genetics are informative only in the presence of variation, whether naturally occurring or artificially introduced. When variation is absent, the importance of a specific gene (or genetic element, however defined) is invisible with respect to the phenotype. Four (11%) of these 36 loci appear to be present exclusively in one ancestry, when considering EU or EAS differences as the extreme of the ancestral difference in MAF (Table 3). For example, the SLE risk alleles for variants rs2476601 at *PTPN22* in EU with MAF = 0.094 and rs4252665 at *ERBB2* in EU with MAF = 0.04 were exclusively present in EU. Both of these EU MAFs are higher than the global MAFs presented in Table 1. For both of these examples, the risk allele is not detected in EAS. Similarly, the risk allele for rs77009341 at *HIP1* in EAS with MAF = 0.018 and rs77971648 at *FCHSD2* in EAS with MAF = 0.104 were found only in EAS, and they are not present in EU. These four examples are truly ancestry-specific loci since no reasonable sample size would ever be expected to establish their contribution to risk in the ancestry where the MAF of the risk allele was below the limit of experimental detection. This does not mean that the target gene is not involved in the pathogenesis of SLE; rather, when the locus is invariant in a second ancestry, the association cannot be detected using a genetics methodology in that ancestry.

Consider the following seven examples (presented in descending order based on the MAF difference between EU and EAS), where allele frequency differences likely explain the failure to detect an association in the second ancestry: rs9311676 is found in EU at the *KCTD6* locus (MAF = 0.414 vs. 0.001); rs10774625 is significant in EU at the *ATXN2* locus (0.477 vs. 0.003); rs4251697 in *CDKN1B* is significant in EAS (0.001 vs. 0.125); rs702814 at *JAZF1* is detected in EU (0.508 vs. 0.015); rs6985109 at *XKR6* is found in EU (0.528 vs. 0.021); rs6705628 at *DGUOK-AS1* is associated in EAS (0.009 vs. 0.156); and rs1131665 at *IRF7* is found in EU (0.267 vs. 0.021). For these markers, the leading hypothesis to explain the lack of association concordance between EU and EAS is, therefore, an inadequate sample size to provide a robust test for concordant association in the second ancestry.

On the other hand, there are 9 (25%) ancestry-specific variants that pass the *p* < 5 × 10^−8^ threshold ≥3 times but have a small difference in MAF <1.5 times and did not pass that threshold in the second population. Eleven (31%) of variants have a difference in MAF 1.5 to <3 times between EAS and EU (Table 3). Most of these loci will probably be confirmed in the second population with a larger sample size and by increasing the number of genotyped variants (Supplementary Material Table S6). We pooled the information for these 36 ancestry-specific variants from two large studies in EUR (25) and EAS (22) to show that there is a tendency toward association with SLE in the opposite ancestry, but without reaching purported significance (*p* < 5 × 10^−08^). When these variants are not included in genotyping panels used for SLE GWAS, detecting the associated loci may fall victim to the vagaries of imputation error (Supplementary Material Table S6).

### Patterns of gene function

To evaluate the pathways, processes, and environmental relationships, we used the 760 published significant associations for the 330 risk loci as the foundation. To define candidate causal genes, we used two approaches. Group 1 with 493 genes included the nearest neighbor expressed gene as the default causal relationship. In addition, we added the causal candidates identified from the literature to Group 1. Group 2 was composed of the top three genes (a total of 764 genes) identified from Open Target Genetics (12), for each of the 760 variants significantly associated with SLE (*p* < 5 × 10^−8^) in any study. There were 291 genes shared by Groups 1 and 2 (Supplementary Material Figure S3).

The results of gene set analysis with either Groups 1 or 2 analyses using Enrichr (13) show that the genes putatively involved in lupus influence a myriad of pathways and processes (Table 4, Supplementary Material Tables S7 and S8). Almost 700 traits are associated with many related pathways, all of which become perspectives on SLE pathogenesis.

The major themes and pathways implicated in this analysis show much involvement, no surprise, of the immune system, both adaptive and innate. Detected in the set analyses are as follows: production and response to cytokines, antigen sensing; immune cell activation and differentiation, immune response and inflammation, immune tolerance, cytokine-mediated signaling including IFN-γ in particular; IFN-α/β; interleukins IL1, IL2, IL4, IL6, IL10, IL13, IL23, and IL35; IFN regulatory factor IRF3, IRF5, and IRF7 pathways; antigen receptor (TCR and BCR)-mediated signaling; toll-like receptor and pattern recognition receptor signaling; C-type lectin receptor signaling; Fc receptor-mediated signaling; NF-κB, JAK-STAT, RAS, MAPK, and AHR pathways; antigen processing and presentation; B-cell proliferation; V(D)J recombination activation; immunoglobulin (Ig) production; Th1, Th2, Th17, Treg and memory T-cell differentiation; T- and NK-cell-mediated cytotoxicity; complement activation; monocyte and dendritic cell activation; neutrophil-mediated immunity; regulation of phagocytosis; basophil activation; mast cell activation; control of immune tolerance by vasoactive intestinal peptide; peripheral T-cell tolerance; CTLA4 inhibitory signaling; and others. SLE-associated genes also regulate immune response processes of cell adhesion and migration, regulation of blood vessels (including leukocyte adhesion to endothelial cell and leukocyte transendothelial migration; regulation of blood vessel endothelial cell migration; integrin pathways; platelet-mediated interactions with vascular and circulating cells; VEGFR signaling; angiopoietin receptor signaling; and erythropoietin signaling).

Other gene sets involving regulation of apoptosis, cell cycle, and cell homeostasis are also highly statistically relevant, including regulation of an immune checkpoint that guards against autoimmunity via apoptosis-like PD-1 signaling; Bcl-2, BAX, BAK family pathways, and regulation of B- and T-cell apoptotic processing; regulation of apoptotic cell clearance; cellular senescence and autophagy; α-synuclein signaling; cyclin D-associated events; mitotic G2/M transition checkpoint; oxidative damage; and DNA damage response. The detection and removal of immune complexes and apoptotic materials appear to be important through complement and Fcγ receptor-mediated phagocytosis.

Among other pathways, SLE-associated genes are involved in metabolic processes, protein modifications, and gene expression regulation (fatty acid synthesis, lipid and lipoprotein metabolism, protein phosphorylation and dephosphorylation, ubiquitination, regulation of nuclease activity, nucleic acid metabolism, vitamin D receptor pathway, histone acetylation, regulation of transcription, phosphatidylglycerol biosynthesis, regulation of glucose transmembrane transport, protein transport, nicotinamide nucleotide biosynthetic process).

Some mechanisms have not been previously reported or, if so, have not been emphasized in the SLE literature, including osteoclast differentiation (135), synapse pruning (136, 137), mucin production in goblet and mucous cells (138), leptin signaling pathway (139), gastrin signaling (140), neurotrophin signaling (141, 142), and prolactin receptor signaling (143, 144), which may contribute to the female predominance in lupus.

Among other phenotypic traits that are potentially controlled by SLE-associated genes are blood cell count and their characteristics (lymphocyte, eosinophil, basophil, neutrophil, monocyte, platelet, red blood cell, and erythrocyte hemoglobin level), serum components level (level of protein, non-albumin protein, complement C4, cholesterol, β-2 microglobulin, immunoglobulin G, and bilirubin), blood pressure, body mass and body shape indexes, aging, psychological traits (anxiety, mood, and irritability), and others (Supplementary Material Table S7).

Another very interesting aspect of SLE pathogenesis is interaction with pathogens and microbiota. On the one hand, genes associated with lupus are involved in the antimicrobial immune response, implicated with responses to molecules of bacterial origin, antiviral signaling through pattern recognition receptors, defensive response to symbionts, and defensive responses against viruses. On the other hand, pathogenic agents may exploit the genes associated with lupus for their survival and benefits, thus modulating the risk of SLE or causing overlapping symptoms that complicate differential diagnosis in some cases. Among hundreds of pathogens and infectious diseases (Supplementary Material Table S7) are leishmaniasis, EBV infection, influenza A, mycobacterium, staphylococcus, hepatitis, SARS-CoV, and many others. Visceral leishmaniasis mimics SLE symptoms including autoantibody production, which in places with endemic leishmaniasis may lead to misdiagnosis (145). A very important association is the EBV that exploits 98 (10%) of 968 SLE-associated genes upon cell infection, with much other evidence implicating this virus as an etiological agent for SLE (1, 97, 146).

Cell and tissue enrichment analysis for SLE-associated genes revealed >200 different cell types and cell states and showed high enrichment for immune cells: B and plasma cells, T cells, dendritic cells, monocytes/macrophages, natural killer cells, neutrophils, and others.

Pathway analysis identified 949 drugs and compounds that may affect the expression of SLE-associated genes (Supplementary Material Table S7), including some now being used for SLE treatment (147): chloroquine, glucocorticoids, cyclophosphamide, methotrexate, cyclosporin A, prednisolone, sirolimus (rapamycin), bortezomib, baricitinib, N-acetyl-L-cysteine, atorvastatin, and vitamin D. Another identified group may cause drug-induced lupus erythematosus (148): IFN-α, IFN-β, minocycline, and sulfasalazine. There are many candidate compounds (Supplementary Material Table S7) that affect the expression of SLE-associated genes that may one day prove efficacious for SLE treatment.

## Discussion

The identification of 330 risk loci for SLE represents enormous progress toward understanding the mechanisms that generate the predisposition to develop SLE, especially compared with having no specific genetic insight before the first gene locus discovery, the HLA association with SLE in 1971 (4, 5). Indeed, from the perspective of hundreds of risk loci, the complexity of the genetic mechanisms and their interrelationships potentially involved in SLE is daunting (Supplementary Material Table S8). Moreover, these 330 loci are only an interim report, with large genetic studies of African ancestry and other populations being absent. If the theoretical considerations presented by Boyle et al. (125) are correct, then the only upper limit as sample sizes enlarge is the entire complement of expressed genes. Based on the work in the literature done to date, the detection limit for effect sizes to achieve *p* < 5 × 10^−8^ has been approximately OR > 1.1 or OR < 0.9. This suggests that, as a community, we are likely to have captured virtually all of the loci in the EU and EAS with OR > 1.2 or OR < 0.8 in variants with more common MAF (>20%).

Certainly, the relative adequacy of the loci known for EU and EAS does not excuse the almost absence of work in African ancestry. Compared with the EAS based on the loci established, research on African ancestry SLE has provided less than 1/20th the locus discovery, and the genes are found in the admixed AA population. The threefold or greater fine mapping discrimination available in African ancestry compared with EAS and EU along with the capacity to perform cross ancestry mapping would be very important for causal variant identification in those loci shared by EAS or EU with African ancestry SLE. Certainly, the deficiency of results from African ancestry is a major goal for future studies of SLE GWAS genetics.

In addition to the large number of published loci, the idea that SLE has a strong genetic component is supported by familial aggregation studies and estimates of heritability. Despite considerable clinical heterogeneity, SLE ranks among the more heritable autoimmune diseases, a conclusion reached from higher heritability (149-151), familial clustering (149, 152-158), and concordance in twin studies (159, 160).

Early studies estimated SLE heritability (the proportion of the phenotypic variance explained by genetic factors) between 44% and 66% (150, 151) but did not identify shared environmental contributions to the risk of developing SLE. Later, in 2015, Kuo et al. (149), in a large population-based study in Taiwan with 23 million participants, estimated heritability at 43.9%, shared environmental factors at 25.8%, and non-shared environmental factors at 30.3%. In this study, the risk of SLE in individuals with one or two affected first-degree relatives compared with the risk in the general population [relative risks (RRs)] was 315.9 for identical twins, 23.7 for siblings, 11.4 for parents, 14.4 for offspring, and 4.4 for spouses without genetic similarity (149). Overall, individuals with 1 and ≥2 affected first-degree relatives had an RR of 17 and 35, respectively (149). Similar results were obtained in another large study in Denmark with 5.2 million individuals. Family members with one and with ≥2 SLE-affected first-degree relatives had hazard ratios (HRs) of 9.8 and 61.1 to develop lupus, respectively. The members with an SLE-affected first-degree relative and second- or third-degree relatives were at a 10.3-fold and 3.6-fold elevated risk of SLE, respectively. In this cohort, the HR was 76.3 for the initially unaffected twin (85.7 and 49.7 for monozygotic and dizygotic twins, respectively), 8.72 for parents, and 17.0 for children of SLE patients (161). In other studies, monozygotic twins had higher SLE concordance rates (24%–69%) than dizygotic twins and non-twin siblings (2%–9% and 2%–5%, respectively) (154, 160, 162). Familial clustering has been found, with 1.3%–6% of SLE patients having a SLE-affected first-degree relative (149, 153, 154, 157, 158, 161). However, the great majority of lupus cases are sporadic, with no relatives being affected.

The 330 published SLE risk loci are anchored by 330 variants with the lowest published probability of association by chance. In addition, 760 published variants exceed the accepted probability for genome-wide significance (*p* < 5 × 10^−8^). When these are evaluated by disequilibrium expansion using *r*^2^ ≥ 0.8 (Pearson’s correlation), there are 16,318 variants to consider and evaluate for possible causation. Here, we have largely limited our analysis to what can be learned from the 330 leading variants and expanded the 760 variants. However, important additional contributions are highly likely to be made by the expanded list of 16,318 variants.

There is no doubt that some of the possible 330 loci are probably false-positive results, despite the conservative threshold that the genetics community now uses, *p* < 5 × 10^−8^. These are probably scattered through the 198 loci that are reported in only one study. The loci tagged by rs933717 at 16q24.2 in *FBXO31* and rs2714333 at 6p24.3 in *RREB1* are prime candidates for being false-positive results, in our opinion. On the other hand, replication is a foundation principle of the scientific method. Of the 330 loci, 133 have been independently confirmed in a second study, greatly reducing the possibility of an artifact of association. As almost half (88 new of 197 loci) of non-replicated loci came from the largest study done so far (26), we anticipate that a majority of these SLE loci will be verified in future larger GWASs with independently ascertained subjects.

Assignments of functional mechanisms based on genomic location become candidates for disease pathogenesis. Amino acid changes are found in 26 of the 330 lead variants, representing a 34-fold enrichment relative to their contribution to the genome (78). These are probably the most straightforward candidates to test for their consequences for gene product activity. Lead variants that make synonymous amino acid changes and those in the untranslated regions are lower and equally enriched. An intronic location for the lead variants is the least enriched (Table 2). Lead variants are depleted relative to the genome in the intergenic regions. These observations lead to the conclusion, also seen in the genetics of virtually all non-Mendelian diseases, that the vast majority of these variants would appear to have a regulatory impact on cellular activity. These possible functional consequences remain candidates until the mechanism can be directly tested with respect to disease risk. These same patterns hold as the variants considered are increased by considering all published significant variants (*n* = 760) and all variants with disequilibrium *r*^2^ > 0.8 (*n* = 16,318) (Table 2).

For at least two loci, *ITGAM* (rs1143679) and *NCF2* (rs17849502), there is evidence that the variant changing the amino acid sequence also has a regulatory function (30, 62, 79, 80).

So, which of these two consequences of the variation is probably causal? Alternatively, perhaps both consequences of these single variants influence SLE risk. Certainly, these consequences are now only candidate causal functions with the possibility remaining that now unknown functions of the variant will be later discovered that are causal by contributing to SLE risk. The discovery of a functional consequence of a variant does not mean that we have found the genetic mechanism. Rather, this important step means that we have an as-yet, unproven hypothesis for genetic mechanisms. Establishing genetic mechanisms is difficult, and we suspect that developing convincing models of genetic mechanisms, even for the loci now identified, will consume the resources available to our community for decades to come.

The most convincing result in the *NCF1* region illustrates this point from our perspective. We count no fewer than 19 independent loci in the 1.5 Mb region near the *NCF1* gene (Table 1) including the most impressive result in the entirety of published genomic experience at rs117026326 with OR = 2.14 and *p* = 2.2 × 10^−298^, which is found in East Asians and Europeans. The SLE risk allele at rs201802880 (*NCF1* p.R90H), associated with reduced expression of NCF1, a negative regulator of TLR signaling, leads to decreased ROS and neutrophil extracellular trap (NET) formation, increased IFN-I detected in peripheral blood, the presence of antiphospholipid autoantibodies, and increased potentially autoreactive double-negative B cells (ABCs) (163, 164).

The change of arginine to histidine in *NCF1*, a subunit of the NADPH protein complex, decreases the phospholipid-binding affinity of NCF1 protein impairing its endosomal localization that results in decreased functionality of NADPH, further acidification of endosomes, and greater cleavage of the endosomal TLRs, TLR7 and TLR9, that facilitates downstream TLR signaling and the excessive activation of plasmacytoid dendritic cells, which are a major subset of IFN-I-producing cells, as touted by Meng et al. (163). They also suggest that hydroxychloroquine would be efficacious for SLE patients with these risk alleles, given its known therapeutic action of raising the pH of endosomes. Olsson et al. (164) showed that *NCF1* p.R90H is associated with decreased extracellular ROS production in neutrophils and an increased expression of type 1 IFN-regulated genes. We are also suspicious that some of the other loci in this genomic neighborhood would also impact a critical NCF1 activity. The importance of NADPH oxidase complex and ROS production in lupus pathogenesis is also supported by the *NCF2* locus at 1q25.3 that encodes p67^*phox*^, another core component of the multi-protein NADPH oxidase where the lupus risk variant is also associated with decreased ROS production.

The SLE risk association at 8p23.1 near the *BLK* gene, which encodes non-receptor tyrosine-kinase of the src family involved in B-lymphocyte development, differentiation, and signaling, is another fascinating and complex association involving multiple variants that appear to focus their effects on the promoters of *BLK* and *FAM167*. The *BLK* gene is involved in the largest 4.5 Mb genomic inversion commonly present in the human species that affects the expression of many genes and is likely inversely associated with SLE (protective) (165, 166). There are also four independent loci associated with lupus in GWASs in the BLK region (8p23.1) covering over 3.6 Mb (Table 1, Supplementary Material Table S1) and mostly correlated with non-inverted status (165). We have shown that distal enhancers influence the coordinated inverse expression of *BLK* and *FAM167A*: the SLE risk haplotype causes lower expression of *BLK* and higher expression of *FAM167A* (166), thereby providing multiple actions to consider that may or may not be responsible for altering SLE risk. We have identified almost 800 differential haplotype– chromatin interactions at 8p23.1, including the “risk-dosage”-dependent influence of variants in enhancers E1, E2, and E3 and promoter on *BLK* expression (166). As shown earlier, two lupus-associated BLK promoter variants, namely, rs922483 and rs1382568, control BLK expression in cell type- and developmental-stage-specific manner (167) adding even more complexity. The interplay between multiple variants influencing risk, haplotypes of diverse composition, the inversion, and multiple functional consequences of variation, suggest that a complete understanding of the genetic architecture will be lost in the complexity for the foreseeable future.

There are numerous other examples of loci that we now have hints about their genetic mechanisms beyond *NCF1* and *BLK*. None rivals the anticipated complexity beyond the HLA region. The probably overly conservative rules we developed for this project were disequilibrium of *r*^2^ > 0.2 coalescing significant (*p* < 5 × 10^−8^) associations together into a locus and disequilibrium at *r*^2^ < 0.2 separating significant associations into separate loci. These rules result in approximately 37 and 41 risk loci in classical and extended HLA regions (168, 169) (loci 110–146 and 106–146 in Table 1 and Supplementary Material Table S1) covering 3.6 Mb or 7.6 Mb of the genome, correspondingly. MHC is the most polymorphic region of the human genome with over 38,000 allele sequences for HLA genes collected in the IPD-IMGT/HLA Database (170) and hundreds of thousands of SNPs and structural genomic polymorphisms, including copy number variants, indels, segmental duplications, inversions, and translocations (171). In SLE, well-established and consistently replicated associations are observed with alleles HLA-DRB1*03:01 and HLA-DRB1*15:01 (24, 25, 31, 45, 47, 48, 52, 59, 172-177). To study this region, more precisely we need to consider not only the individual variants and structural variants but also the alleles for many HLA and non-HLA genes. Moreover, some HLA molecules will develop unique activities when heterodimers form with variants from the different haplotypes (compound risk allele heterozygosity) found on the two chromosomes of each individual (24, 175). In the absence of major technical and analytic breakthroughs, identifying the causal variants and separating the many competing influences in the HLA region will require work for decades to come.

Finding a mechanism attributable to a plausibly causal variant does not establish that mechanism as causal. At best, these relationships become candidates for causation. Unknown or undetected mechanisms remain possible. Additional direct evidence from therapeutics or from animal or *in vitro* models is needed to increase suspicion that the identified mechanism is causal for SLE.

The stringent criterion of requiring the association to obtain a low probability, at present set by the community at *p* < 5 × 10^−8^, is not foolproof. Indeed, those loci achieving this level of causation in only one study have not yet met the replication requirement of the empirical scientific method. On the other hand, *p* < 5 × 10^−8^ is a stringent requirement, suggesting that a small proportion of those loci awaiting will not be confirmed.

Clinical implications of the genetic findings are complex and not direct. At this point, the GWAS genetics we discuss here provide a foundation for a subsequent understanding of pathogenesis; however, direct applications for diagnosis or treatment in clinical practice are not yet available. In general, the relationships, while statistically significant, are not sufficiently discriminating. The rare cases of single-gene defects with very large effect sizes (2) are an exception to this conclusion but would require exome or whole genome sequencing to identify these SLE cases.

Despite there being no epidemiological data on SLE for 80% of the countries in the world, incidence estimates in the same ancestry may vary by >10-fold. Nevertheless, Europeans consistently have a 1.5–5 times lower incidence of SLE compared with non-Europeans (178-182). AA, EAS, and MA/Mestizo patients exhibit a larger number of manifestations characteristic of SLE, accumulate damage from SLE more rapidly, and have more severe symptoms (whether hematological, cardiovascular, serosal, neurological, or renal), higher morbidity, and a younger average age of onset compared with Europeans. For example, in AA patients, end-stage renal disease is linked to the presence of the *APOL1* nephropathy risk genotype, which is more common in the AA general population (183, 184). In contrast, Europeans have a higher prevalence of photosensitivity. Furthermore, different ethnic groups respond differentially to standard therapy, including cyclophosphamide, mycophenolate, rituximab, and belimumab. Some of these variations may be accounted for by environmental and/or socioeconomic factors; however, ancestry remains a key determinant of outcome (180, 182, 185-190). Genetic differences are widely thought to be at least part of the explanation for these differences.

Nevertheless, at this point in our effort to understand the genetic architecture of SLE and despite having 330 risk loci, there are no convincing examples that support their ancestry-specific genetic mechanisms. While the consequences of full development of African ancestry in SLE are awaited and may change this observation, what we conclude to this point is that while the variants may change in frequency across ancestries, the predominance of the results now available favors the genetic architecture being shared across ancestries.

The genetic loci generally contribute to SLE risk independently in an additive fashion without positive or negative synergy (24, 191). Thus, the polygenic additive model of inheritance with many small-magnitude, independent genetic effects altering SLE risk has proven to be the most robust model for the interrelations between alleles of risk loci. While a comprehensive analysis of dominance genetic effects (including multiplicative and fully recessive and dominant models) has not been undertaken in SLE, a recent analysis of the UK Biobank strongly suggests that these models fit the data better for the rare risk loci, which may or may not be detected by the additive model (192). Epistatic interactions are also not ordinarily modeled in SLE, consistent with the many failed efforts to identify epistasis, which is only rarely established (193). Perhaps, epistasis is very important, but because of the poor statistical power to establish its presence, which dooms attempts for confirmation, we fail to detect the specific instances of epistasis. Indeed, the sample sizes needed to confirm dominance models and epistasis are beyond current capabilities and are not practical. In contrast, pleiotropy for SLE-associated variants is broadly known, where a single SLE variant influences multiple genes (see Supplementary Material Table S1) and/or phenotypic traits (shared loci between SLE and type 1 diabetes, rheumatoid arthritis, Crohn’s disease, ulcerative colitis, multiple sclerosis, and other disorders) (see Supplementary Material Table S7) (194, 195).

For all complex phenotypes, the effect sizes are small for the risk loci discovered by genetic association. This does not reflect their importance to pathogenesis. Indeed, fundamental processes for SLE pathogenesis that do not vary among human beings in ways that change risk would not be detected by genetic association studies. Critical genes for the phenotype are sometimes captured by rare variants that have large effect sizes. Indeed, many genes induce a lupus phenotype with probably few if any other variants contributing to a lupus phenotype. [Please refer to ref. (2) for a discussion of monogenic lupus and their integration with SLE genetic association results].

**FIGURE 5:**
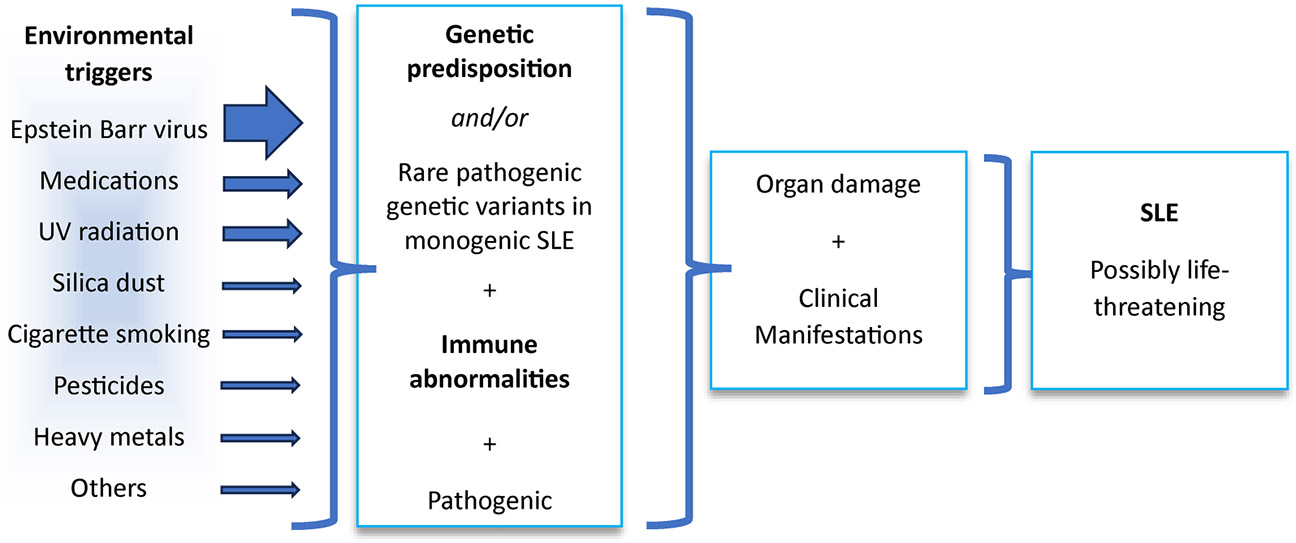
Model of systemic lupus erythematosus (SLE) pathogenesis. Plausible etiology of SLE with their relative proportional importance estimated by the thickness of the arrows followed by the processes contributing, including genetics and other experiments, culminating in organ injury and the clinical manifestations of the disease recognized as SLE.

The 330 now known are all germline variations; however, there are important additional considerations. The interplay of genetic, epigenetic, and environmental factors is assumed to hold the secrets for a complete understanding of disease mechanisms. While we are well on the path toward a comprehensive understanding, a greater part remains unknown. Environmental exposure may cause epigenetic changes, encourage somatic mutations, trigger activation of innate and adaptive immune responses, or provoke loss of immune homeostasis with autoantibody production and inflammatory cytokine dysregulation, all of which may induce or accelerate the development of SLE in susceptible individuals (196-198). Environment exposures that may trigger SLE have been reviewed recently (196, 198-201) and include crystalline silica dust, air pollution, cigarette smoking, other respiratory exposures, pesticides, chemicals in household products, polycyclic aromatic hydrocarbons, heavy metals, UV radiation, uranium processing, diet, alcohol use, sleep quality, vaccinations, medications, exogenous hormones, and infections, in particular, with EBV (1, 97, 146). Our simplistic model of the progression from plausible etiology to disease is presented in Figure 5.

The possible environmental contribution from EBV has been bolstered from a different perspective. In 2018, Harley et al. (97) showed the DNA of about half of the lupus risk loci are bound by a group of transcription factors that are enriched at the risk loci of SLE and six other largely idiopathic inflammatory diseases, suggesting shared disease risk mechanisms. The most closely associated top 10 human transcription factors with the 53 EU ancestry lupus risk loci included in the study are RELA, NFATC1, PML, BCL3, NFIC, NFKB2, RELB, TBP, STAT5A, and TBLIXR1, with RRs from 5.54 to 25.22 (10^−26^ > Pc > 10^−53^, where Pc is the Bonferroni-corrected probability) (97). The possible environmental interaction is that approximately half of the SLE risk loci are bound by EBV-encoded transcription cofactors EBNA2, EBNA3C, and EBNA-LP together with human TFs such as POLAR2A, RELA, RELB, NFKB1, NFKB2, EP300, and others, forming super-enhancers (26, 97). The role of EBV in SLE pathogenesis is supported by association and the possible role of anti-EBNA1 as the foundation for molecular mimicry (1).

TF binding changes may range from having a large impact on specific gene expression to not having any impact. TF motifs appear to occur in clusters with some built-in redundancy that may buffer, thereby reducing the impact of the genetic regulatory perturbations in one instance of a cluster of TF motifs (98). Unfortunately, the power of GWAS is poor in identifying epistatic interactions between SLE loci, relegating the work done to date to largely additive intergenic mechanisms.

Meanwhile, only a few examples of somatic mutation possibly contributing to SLE pathogenesis are known (202); however, somatic mutation also may be a part of another process, such as clonal hematopoiesis. Clonal hematopoiesis of indeterminate potential (CHIP) with mutations in cell clones was identified in 10.7% of SLE patients, which was relatively high, compared with unaffected individuals and conditioning on age (203). Most variants (62.5%) were located in the *DNMT3A* gene, and other mutations were reported in *TET2, GNAS, ASLX1, TP53, SH2B3, SETBP1, CBL, JAK2, PPM1D, ETV6, KDM6A, NFE2,* and *SMC3* (203). Another example of clonal hematopoiesis in SLE is RAS-associated autoimmune leukoproliferative disease (RALD) manifested with an SLE-like syndrome or SLE (204, 205). RALD is characterized by persistent monocytosis; often associated with leukocytosis, lymphoproliferation, and autoimmune phenomena, early onset (mostly at the age <5 years old), and resistance to IL2 depletion-dependent apoptosis; and caused by somatic mutations in RAS genes (NRAS and KRAS), which plays an important role in intracellular signaling and control proliferation and apoptosis (204, 206, 207). Whether somatic mutation in RAS genes precedes SLE or appears later in patients having RALD with SLE remains unknown.

The mechanisms suggested by our error-prone effort to identify the gene products with an agency to alter SLE risk suggest many cellular functions by gene set analysis (Supplementary Material Table S8). This is the effort to understand the consequences of possible causal variants to identify their intermediate targets that then act to change risk. At this point, this is an inexact process. The causal variants are not unambiguously identified in the vast majority of the 330 loci. Furthermore, what they do is only partially known. Many plausible causal activities and relationships likely remain unknown to us. The multiple activities and consequences of a candidate gene target are usually left without establishing the particular activity that alters risk. Nevertheless, the number and variety of cellular processes that can be implicated in SLE pathogenesis is overwhelming (Supplementary Material Table S8). Clearly, there is a strong immune response component, which serves as the organizing principle when considering these results. The potential of other processes to influence the discretion of immune responsiveness, as influenced by apoptosis, for example, can provide context to these considerations.

Nevertheless, themes involving impaired mechanisms of apoptosis, autophagy, DNA degradation, and clearance of cellular debris appear to be particularly prominent from the gene set analysis (Supplementary Material Table S8). In SLE both excessive cell death via apoptosis and NETosis and a decrease in debris clearance are responsible for extracellular nuclear material (free or in microvesicles or microparticles with DNA and RNA, proteins, and nucleic acids-protein complexes) that initiate the autoimmune response and production of autoantibodies. Autoimmune complexes engage the activating receptors including the Fcγ receptor, FcgRIIA on plasmacytoid dendritic cells, and other immune cells leading to internalization and downstream activation of intracellular TLRs and other nucleic acid sensors in the cytoplasm. They also stimulate the production of production of proinflammatory cytokines, especially type I IFN (208-212). These multilayer processes in SLE pathogenesis involve genes participating in apoptosis (*TNFRSF21, IKBKG, IKBKB, BCL2L11, BAK1, TRAF3, IRF1, IRF3, IRF4, IRF5, IRF7, CCND2, PYCARD*), regulation of ribonuclease activity (*OAS1, OAS2, OAS3, OASL*), complement activation (*C1QB, C2, C3, C4B, CFB, ITGAM, ITGAX*), and Fcγ receptor-mediated phagocytosis (*FCGR2A, FCGR3A, FCGR3B, PTPRC, LYN, NCF1*).

Upon internalization, in the cytoplasm, DNA from cell debris or autoimmune complexes interacts with endosomal toll-like receptor 9 (TLR9) or the cyclic GMP–AMP synthase (cGAS), stimulator of IFN genes (STING) system, whereas RNA–protein complexes interact with internal RNA sensors such as TLR3 and TLR7, the retinoic acid-inducible gene 1 (RIG-I), and melanoma differentiation-associated protein 5 (MDA5) pathways. Downstream signaling from this nucleic sensing triggers the production of type I IFN and other cytokines (212-216). In turn, the overproduction of type I IFN can stimulate the maturation of dendritic cells, the major producers of type I IFNs, and the expression of TLR7 and TLR9 and other IFN-dependent genes in different immune cells. It can also reinforce the synthesis of proinflammatory cytokines and chemokines, leading to activation of autoreactive B cells and Th1 cells, the production of autoantibodies, and loss of self-tolerance and other effects. In contrast to normal immune response, for example to viral infections, where TLRs can discriminate self-derived DNA from microbe-derived DNA, in patients with SLE the regulation of nucleic sensing and pathways triggering activation of type I IFN production is disturbed and SLE patients exhibit abnormally high levels of INF-α in their blood correlating with more severe disease manifestations (217, 218). Nucleic acid sensing, including all three main sensing pathways, namely, cGAS-STING, RLR-MAVS, and TLRs, that activate IFN production and regulation of type I IFN production by antigen-presenting cells including dendritic cells would appear to involve many of the SLE loci, including *TLR7, IRF3, IRF4, IRF5, IRF7, IRF8, STAT4, IFIH1, ITGAM, ITGAX, TRAF3, TNFAIP3, IL10, UBE2L3, IKBKB, IKBKG, IKBKE, IRAK1, IRAK4, IL12A, IL12B, PTPN11, PTPN22, USP18, RELA, JAK2, MAPKAPK2, STAT1, ATG5,* and *TYK2* (Supplementary Material Table S7).

Many of the risk genes would appear to be unified under the concept of B- and T-cell activation and signaling, leading to the loss of central and peripheral immunological tolerance, aberrant adaptive immune responses, and autoantibody production. Loci involved include HLA genes, such as *PTPN22, TNFSF4, PPP2CA, CD40, CD44, CD80, ELF1, BANK1, BLK, LYN, KIT, RASGRP3, IKZF3, ETS1, CDKN1A,* and *CDKN1B*. These changes in gene pathways mediate the decrease of the activation threshold for CD4+ T and B cells upon autoantigen encounter and stimulate their proliferation and cytokine production (219-222). As a complex disease with variable manifestations, SLE affects many overlapping pathways and cells.

Despite the incredible advances that 330 purported risk loci imply, what we know of the genetics of SLE now, however, remains woefully incomplete. There are variant types that are poorly incorporated into genome-wide association studies (GWASs) for technical reasons (e.g., CNVs, endogenous retroviruses, extended structural variations such as large insertions, deletions, and inversions). In addition, the study of some ancestries, especially African ancestry, is embarrassingly inadequate, rendering this a partial analysis. Nevertheless, the 330 published SLE risk loci represent an important accomplishment toward understanding these disease differences.

Beyond the desperately needed studies in African ancestry, the next horizons in these studies of SLE genetic architecture include the genetic evaluation of SLE subsets (e.g., nephritis, cytopenias, serologies, IFN, and cytokines), the genetic correlations with other disorders (e.g., Sjogren’s syndrome, rheumatoid arthritis), DNA methylation, genomic structure, assigning variant mechanisms to cell types, the role of transcription factors and other regulatory elements in candidate mechanisms, the interaction of SLE genetics with candidate environmental etiologies (e.g., EBV), using the advanced understanding of plausible mechanism to identify and develop new therapies and preventive measures, and establishing that variation in candidate mechanisms do alter disease risk.

In summary, we have come a long way in the half century of SLE genetic association studies. The identified 330 risk loci represent a host of human biological processes and many potentially and possibly environmental processes involved in SLE pathogenesis. Once these and their subsequent congeners are fully understood, we hope that strategies for highly efficacious therapies and simple preventive strategies will become available.

## Supplementary Material

Image 1 SUPPLEMENTARY FIGURE 1
Minor allele frequency versus association effect size.

Image 2 SUPPLEMENTARY FIGURE 2
Number of loci influencing expression or function of plausible target genes. Of the Group 1 & 2 genes (*n* = 971) related to the 760 published variants associated with SLE at *p* < 5 × 10^−8^, one to 9 loci are related to individual genes, as indicated by the color coding in the legend.

Image 3 SUPPLEMENTARY FIGURE 3
Genes implicated by the overlap of both Groups 1 and 2.

Table 8 SUPPLEMENTARY TABLE 8
Pathways associated with lupus loci.

Table 6 SUPPLEMENTARY TABLE 6
Ancestry bias at 36 confirmed SLE risk loci (*p* < 5 × 10^−8^, ≥3 times) and summary statistics for these loci in EUR from (^33^) or EAS from (^30^).

Table 4 SUPPLEMENTARY TABLE 4
Genes whose expression is influenced by one or more SLE loci.

Table 5 SUPPLEMENTARY TABLE 5
Transcriptional regulators and DNA-interacting proteins that are among the plausibly causal gene candidates in SLE.

Table 2 SUPPLEMENTARY TABLE 2
The distribution of loci according to minor allele frequency (MAF) or odds ratio (OR) and the number of times the locus has been published with *p* < 5 × 10^−8^ (Summarized in Figure 4).

Table 3 SUPPLEMENTARY TABLE 3
The 16,318 variants in disequilibrium at *r*^2^ > 0.8 with 760 published SLE risk variants. The variants in the expanded set of 16,318 are taken from the population in which the published SLE risk variant was found.

Table 1 SUPPLEMENTARY TABLE 1
The published 760 SLE risk variants at *p* < 5 × 10^−8^.

Table 7 SUPPLEMENTARY TABLE 7
Traits (pathways, cells, drugs, etc.) influenced by SLE-associated genes.

## Figures and Tables

**TABLE 1:** The 330 systemic lupus erythematosus (SLE) published risk loci.

N	Location	Leading variant	Candidate gene	p-value	Population	Minor allele	Global MAF	OR	Ref.
1	1p36.33	rs12093154	*SDF4*	2.51E-09	TA	A	0.13	0.84	(22)
2	1p36.23	rs3795310	*RERE*	3.36E-08	TA	C	0.48	0.88	(22)
3	1p36.11	rs4649203	*IFNLR1*	9.9E-09	EAS, TA	G	0.43	1.16	(23)
4	1p34.3	rs28411034	*MTF1*	1.50E-10	TA	A	0.21	0.86	(22)
5	1p31.3	rs6702599	*IL12RB2*	3.18E-09	TA, EU	A	0.23	0.84	(22)
6	1p31.3	rs3828069	*IL12RB2*	1.77E-09	TA	C	0.19	0.85	(24)
7	1p13.2	rs2476601	*PTPN22*	1.1E-28	EU, TA	A	0.027	1.43	(25)
8	1p13.1	rs9651076	*CD58*	3.26E-13	EAS, TA	G	0.42	1.12	(26)
9	1q23.1	rs116785379	*ETV3*	6.68E-16	EAS	C	0.043	1.21	(26)
10	1q23.1	rs112806509	*FCRL5*	1.76E-15	EAS, TA	(T)9	0.13	0.81	(26)
11	1q23.3	rs12120358	*FCGR2A*	2.96E-11	EAS	T	0.17	0.84	(26)
12	1q23.3	rs1801274	*FCGR2A*	5.05E-15	TA, EU	G	0.44	1.17	(22)
13	1q23.3	rs111994823	*FCGR2A*	3.41E-11	EAS	C	0.032	1.41	(26)
14	1q23.3	rs76107698	*FCGR2C*	1.85E-30	EAS	C	0.082	0.79	(26)
15	1q23.3	FCGR3B CN (<2)	*FCGR3B*	<1E-09	TA, EU	-	-	1.80	(27)
16	1q23.3	rs75773410	*FCGR2B*	3.84E-15	EAS	G	0.17	0.77	(26)
17	1q25.1	rs1234314	*TNFSF4*	6.52E-28	TA, EAS, EU, MA	G	0.39	-	(28)
18	1q25.1	rs2205960	*TNFSF4*	3.16E-90	EAS, EU, MA, TA	T	0.18	1.37	(26)
19	1q25.1	rs117278480	*PRDX6-AS1*	2.23E-52	EAS	G	0.014	0.65	(26)
20	1q25.1	rs2039982	*PRDX6*	1.85E-37	EAS, EU, MA, TA	C	0.37	1.24	(26)
21	1q25.1	rs549669428	*RABGAP1L*	4.53E-08	TA	G	0.32	0.84	(22)
22	1q25.3	rs17849501	*NCF2*	3.45E-88	EU, EU, MA, TA	T	0.018	2.10	(25)
23	1q25.3	rs10911363	*NCF2*	2.52E-17	TA, EAS, EU	T	0.37	1.17	(24)
24	1q25.3	1:183524640:I (b37)	*NCF2*	4.21E-10	EU	-	0.11	1.37	(29)
25	1q25.3	rs13306575	*NCF2*	2.28E-14	EAS, MA	A	0.018	1.31	(26)
26	1q25.3	rs35937854	*NCF2*	1.49E-09	AA	G	0.014	2.34	(30)
27	1q25.3	rs41263646	*NCF2*	1.54E-08	EU	T	0.11	0.78	(24)
28	1q25.3	rs10911628	*EDEM3*	2.30E-13	EU	A	0.091	1.95	(31)
29	1q31.2	rs1547624	*RGS1*	4.55E-08	EAS	A	0.23	1.17	(22)
30	1q31.3	rs34889541	*PTPRC*	2.44E-12	TA, EAS	A	0.077	0.81	(29)
31	1q32.1	rs3806357	*RNPEP*	4.25E-09	EAS	A	0.088	1.11	(26)
32	1q32.1	rs2297550	*IKBKE*	6.22E-13	TA, EAS	G	0.24	1.19	(22)
33	1q32.1	rs529561493	*RASSF5*	9.8E-09	EAS	C	0.0004	3.66	(32)
34	1q32.1	rs3024493	*IL10*	2.35E-13	TA, EU	A	0.082	1.25	(24)
35	1q42.2	rs6586391	*TARBP1*	4.13E-08	TA	C	0.21	1.13	(33)
36	1q42.3	rs9782955	*LYST*	1.25E-09	EU	T	0.16	1.16	(25)
37	1q44	rs1780813	*SMYD3*	3.50E-08	EU	T	0.057	0.55	(34)
38	2p25.1	rs75362385	*ID2*	8.40E-13	EAS	T	0.17	0.89	(26)
39	2p23.1	rs7579944	*LBH*	1.02E-20	EAS, TA	C	0.44	0.88	(26)
40	2p23.1	rs17321999	*LBH*	2.22E-16	TA, EAS, EU	A	0.15	0.83	(29)
41	2p22.3	rs13385731	*RASGRP3*	1.29E-33	EAS, TA	C	0.077	1.29	(26)
42	2p16.1	rs1432296	*REL*	1.34E-08	TA	T	0.058	1.18	(24)
43	2p14	rs11126034	*SPRED2*	2.60E-10	EAS	C	0.44	1.12	(26)
44	2p14	rs268134	*SPRED2*	1.14E-10	EU	A	0.19	1.21	(25)
45	2p13.1	rs4852324	*DGUOK-AS1*	5.7E-14	EAS	C	0.20	0.79	(35)
46	2p13.1	rs6705628	*DGUOK*	6.9E-17	EAS, TA	T	0.15	0.75	(35)
47	2p13	rs73954925	*BCL2L11*	5.11E-11	EAS	G	0.11	1.17	(26)
48	2q21.3	rs218174	*MCM6*	1.83E-13	EAS	A	0.31	1.12	(26)
49	2q22.2	rs2381401	*ARHGAP15*	1.73E-09	TA	T	0.30	1.15	(22)
50	2q24.2	rs2111485	*IFIH1*	4.55E-12	TA, EAS, EU	G	0.34	0.88	(24)
51	2q24.2	rs10930046	*IFIH1*	1.16E-08	AA	C	0.19	0.70	(36)
52	2q24.2	rs13023380	*IFIH1*	5.20E-14	TA, EU	A	0.22	0.82	(36)
53	2q32.2	rs9630991	*NEMP2*	1.08E-13	TA, EU	A	0.33	0.85	(22)
54	2q32.2	rs11889341	*STAT4*	5.89E-137	TA, EAS, EU, MA	T	0.24	1.59	(22)
55	2q32.3	rs71030321	*STAT4*	3.16E-49	EAS, EU	(A)15	0.098	1.46	(26)
56	2q33.1	rs7572733	*PLCL1*	1.25E-14	EAS	T	0.39	1.14	(26)
57	2q33.2	rs3087243	*CTLA4*	7.02E-11	EAS, TA	A	0.42	1.19	(37)
58	2q34	rs7565158	*ENSG00000273118*	2.88E-10	EAS	T	0.47	1.10	(26)
59	2q34	rs3768792	*IKZF2*	1.21E-13	EU, EAS	G	0.26	1.24	(25)
60	2q36.3	rs5839171	*MIR5702*	1.98E-08	Egyptian	-	0.44	0.63	(38)
61	3p24.1	rs438613	*LINC01967*	7.52E-09	EAS, TA	C	0.36	0.92	(26)
62	3p14.3	rs180977001	*PXK*	2.47E-08	EU	C	0.018	1.27	(24)
63	3p14.3	rs9311676	*PXK*	3.06E-14	EU, TA	T	0.22	1.17	(39)
64	3p13	rs7637844	*LINC00877*	1.28E-08	EAS	C	0.24	0.88	(26)
65	3q13.33	rs79498479	*CD80*	1.89E-30	EAS, EU, TA	AAACAAACA	0.21	0.83	(26)
66	3q25.33	rs77583790	*IL12A*	6.49E-11	EU	A	0.003	2.15	(25)
67	3q25.33	rs564976	*IL12A*	2.2E-10	EU	A	0.20	0.87	(24)
68	3q26.2	rs10936599	*ACTRT3*	1.92E-13	TA, EAS	T	0.27	1.14	(40)
69	3q28	rs6762714	*LPP*	4.00E-15	TA, EU	C	0.35	1.16	(29)
70	4p16.3	rs3733345	*DGKQ*	2E-11	TA, EAS	G	0.47	0.89	(24)
71	4p16.3	rs231694	*FAM193A*	9.71E-09	EAS	T	0.21	1.11	(26)
72	4p16.1	rs13116227	*GPR78*	3.05E-11	EAS	T	0.17	1.34	(41)
73	4p14	rs71196850	*RHOH*	1.35E-16	EAS	CT	0.37	1.13	(26)
74	4q12	rs2855772	*KIT*	1.21E-15	EAS	C	0.20	1.40	(41)
75	4q21.21	rs6533951	*BMP2K*	1.25E-10	EAS	G	0.47	1.11	(26)
76	4q21.23	rs6841907	*COQ2*	1.4E-09	EAS	A	0.34	1.10	(26)
77	4q21.3	rs144261754	*AFF1*	3.45E-10	EAS	AAA	0.21	0.90	(26)
78	4q21.3	rs116940334	*AFF1*	3.15E-10	EAS	T	0.034	0.83	(26)
79	4q24	rs4643809	*BANK1*	3.53E-24	EAS, AA, EU, TA	T	0.45	0.85	(26)
80	4q25	rs956237	*LEF1*	4.47E-11	EAS	A	0.39	1.11	(26)
81	4q25	rs58107865	*LEF1*	6.57E-25	EAS	C	0.058	0.80	(26)
82	4q27	rs11724582	*IL2*	1.71E-08	TA	G	0.17	0.88	(24)
83	4q35.1	rs10018951	*TRAPPC11*	1.18E-14	EAS	T	0.15	1.31	(41)
84	5p15.33	rs7725218	*TERT*	2.47E-17	EAS, TA	A	0.41	1.13	(26)
85	5p13.2	rs6871748	*IL7R*	3.96E-08	TA	C	0.17	0.89	(22)
86	5q21.1	rs12153670	*ST8SIA4*	7.65E-10	EAS, EU, TA	G	0.24	1.15	(42)
87	5q23.3	rs74989671	*FBN2*	1.61E-08	EAS	G	0.091	1.54	(43)
88	5q31.1	rs370449198	*FNIP1*	4.41E-08	EAS	(C)8	0.020	0.72	(26)
89	5q31.1	rs2549002	*RAD50*	2.40E-10	EAS	C	0.43	0.91	(26)
90	5q31.1	rs115267018	*SKP1*	9.08E-11	EAS	C	0.082	0.85	(26)
91	5q31.1	rs244689	*TCF7*	1.21E-20	EAS, EU	A	0.28	1.29	(44)
92	5q31.1	rs7726414	*SKP1*	3.17E-16	TA, EAS, EU	T	0.18	1.32	(22)
93	5q31.1	rs138305363	*UBE2B*	4.07E-08	EAS	G	0.002	0.62	(26)
94	5q31.1	rs707149	*GPX3*	3.58E-08	EAS	A	0.40	0.81	(45)
95	5q31.1	rs2233302	*GPX3*	1.17E-08	EAS	G	0.099	0.76	(45)
96	5q33.1	rs10036748	*TNIP1*	1.27E-45	EU, EAS, MA, TA	C	0.44	1.38	(25)
97	5q33.3	rs2421184	*IL12B*	4.67E-12	EAS	A	0.35	0.84	(45)
98	5q33.3	rs2431697	*MIR146A*	5.98E-29	TA, AA, EAS, EU	C	0.38	0.78	(22)
99	5q33.3	rs57095329	*MIR146A*	2.74E-08	EAS	G	0.14	1.29	(46)
100	6p25.3	rs9503037	*DUSP22*	1.36E-15	EAS	G	0.20	0.88	(26)
101	6p24.3	rs2714333	*RREB1*	1E-08	EAS	T	0.022	3.11	(32)
102	6p22.3	rs17603856	*ATXN1*	3.27E-12	TA, EU	G	0.23	0.88	(29)
103	6p22.3	rs10807602	*ATXN1*	1.99E-08	TA	T	0.42	1.11	(33)
104	6p22.3	rs10498722	*CARMIL1*	2.87E-10	EU	T	0.074	1.30	(24)
105	6p22.2	rs79774308	*CARMIL1*	5.76E-10	EAS	G	0.008	1.45	(26)
106	6p22.2	rs36014129	*BTN3A2*	1.21E-24	EU	A	0.021	1.50	(24)
107	6p22.2	rs9295676	*TRIM38*	7.94E-11	EAS	T	0.22	1.11	(26)
108	6p22.1	rs10946940	*ZSCAN26*	8.20E-09	EU	G	0.50	0.69	(31)
109	6p22.1	rs77285596	*ZSCAN23*	2.20E-19	EAS	G	0.020	1.36	(26)
110	6p21.33	rs1270942	*C4A*	2.25E-165	EU, AA, TA	G	0.031	2.28	(25)
111	6p21.33	rs2263318	*DDX39B*	1.05E-19	EAS	A	0.081	0.60	(26)
112	6p21.33	rs2524117	*HLA-C*	8.20E-21	EU	C	0.32	1.34	(47)
113	6p21.33	rs9265604	*HLA-C*	1.3E-08	EU	C	0.38	0.83	(47)
114	6p21.33	rs4458721	*MICA*	2.73E-10	EAS	T	0.31	1.18	(26)
115	6p21.33	rs2246618	*MICB*	1.76E-10	EU	T	0.28	1.49	(47)
116	6p21.33	rs2256974	*LST1*	2.23E-25	EAS	A	0.29	0.84	(26)
117	6p21.33	rs9378200	*MICA*	8.2E-17	EU	C	0.14	0.59	(47)
118	6p21.33	rs3115674	*LY6G6F*	1.43E-08	EAS	G	0.043	0.65	(26)
119	6p21.33	rs117217736	*LY6G6F*	1.06E-12	EAS	C	0.001	0.48	(26)
120	6p21.33	rs74290525	*SKIC2 (SKIV2L)*	1.12E-12	EU	A	0.058	2.06	(25)
121	6p21.32	rs9268807	*HLA-DRB5*	4.90E-117	EAS, EU	C	0.16	1.60	(26)
122	6p21.33	rs554383943	*CYP21A1P*	2.47E-13	EAS	G	0.003	3.63	(26)
123	6p21.33	rs406658	*SKIC2 (SKIV2L)*	4.95E-08	EAS	A	0.22	0.71	(48)
124	6p21.33	rs1150755	*MICB*	6.11E-117	TA, EU, MA	T	0.052	1.30	(24)
125	6p21.32	rs200283861	*ATF6B*	2.51E-23	EAS	A	0.22	1.20	(26)
126	6p21.32	rs9281656	*PRRT1*	1.23E-11	EAS	(A)18	0.18	1.19	(26)
127	6p21.32	rs8192591	*NOTCH4*	4.2E-21	EU	T	0.031	1.34	(47)
128	6p21.32	rs368529276	*TSBP1*	3.52E-19	EAS	G	0.003	2.52	(26)
129	6p21.32	rs3129941	*TSBP1*	9.65E-09	EAS	A	0.20	-	(49)
130	6p21.32	rs72548051	*HLA-DRA*	1.3E-29	EAS	AC	<0.001	1.67	(50)
131	6p21.32	rs9275572	*HLA-DQA2*	7.00E-48	EU, AA, EAS, MA, TA	A	0.34	1.69	(20)
132	6p21.32	rs28529717	*HLA-DQA1*	4.3E-21	EAS	A	0.32	2.19	(32)
133	6p21.32	rs9268989	*HLA-DRB5*	6.49E-14	EAS	A	0.42	0.86	(26)
134	6p21.32	rs34452045	*HLA-DRA*	3.39E-31	EAS	-	0.18	0.69	(26)
135	6p21.32	6:32449301:T:<INS:ME:ALU>	*HLA-DRA*	7.84E-37	EAS	-	0.13	0.62	(26)
136	6p21.32	rs2647078	*HLA-DRB1*	8.51E-21	EAS	G	0.16	0.80	(26)
137	6p21.32	rs660895	*HLA-DQA2*	4.77E-31	EAS	G	0.20	0.62	(51)
138	6p21.32	rs1966002	*HLA-DRB1*	8.43E-31	EAS	T	0.17	1.34	(26)
139	6p21.32	rs9273349	*HLA-DQA1*	3.24E-38	EAS	C	0.37	1.58	(51)
140	6p21.32	rs9273371	*HLA-DQA1*	1.18E-09	EAS	T	0.21	1.61	(48)
141	6p21.32	rs17412833	*HLA-DQB1*	7.81E-17	EAS	T	0.29	1.79	(52)
142	6p21.32	rs189311301	*HLA-DQB1*	2.92E-10	EAS	G	0.015	2.23	(26)
143	6p21.32	rs17206287	*HLA-DRB5*	2.04E-12	EAS	G	0.17	1.20	(26)
144	6p21.32	rs7753017	*HLA-DQB2*	5.33E-43	EAS, EU	A	0.34	0.75	(26)
145	6p21.32	rs115910061	*HLA-DPA1*	4.82E-12	EAS	T	0.019	0.72	(26)
146	6p21.32	rs1431403	*HLA-DPB1*	1.14E-22	EAS	C	0.46	0.85	(26)
147	6p21.31	rs3748079	*ITPR3*	1.78E-08	EAS	T	0.17	1.88	(53)
148	6p21.31	rs3734266	*BLTP3A (UHRF1BP1)*	9.40E-24	TA, EAS, EU	C	0.16	1.29	(22)
149	6p21.31	rs11755393	*BLTP3A (UHRF1BP1)*	1.56E-12	TA, EU	G	0.47	0.87	(42)
150	6p21.31	rs820077	*ANKS1A*	1.31E-08	EU	G	0.25	1.19	(25)
151	6p21.32	rs2762340	*ANKS1A*	4.93E-15	TA, EAS	G	0.32	0.87	(40)
152	6p21.31	rs10807150	*DEF6*	6.06E-16	EAS, TA	C	0.38	1.25	(45)
153	6p21.2	rs34868004	*CPNE5*	4.46E-09	EAS	A	0.23	1.10	(26)
154	6q15	rs3857496	*BACH2*	7.66E-09	EAS	C	0.33	1.12	(26)
155	6q15	rs597325	*BACH2*	4.03E-12	TA, EAS	A	0.30	0.89	(29)
156	6q21	rs6923608	*PRDM1*	6.13E-09	EU	A	0.19	1.20	(24)
157	6q21	rs548234	*ATG5*	2.39E-28	TA, EAS, EU	C	0.20	0.81	(22)
158	6q21	rs9373839	*ATG5*	4.18E-15	TA, EU	C	0.11	1.19	(24)
159	6q22.1	rs9488914	*DSE*	1.14E-08	EAS	C	0.38	0.86	(26)
160	6q23.3	rs2327832	*IFNGR1*	1.76E-13	EU	G	0.095	1.22	(24)
161	6q23.3	rs148314165	*TNFAIP3*	3.48E-84	EAS, EU, TA	(T)4	0.023	1.71	(26)
162	6q25.2	rs9322454	*IPCEF1*	2.42E-08	EAS	A	0.26	1.09	(26)
163	7p15.1	rs702814	*JAZF1*	1.97E-11	TA, EU	T	0.22	1.15	(24)
164	7p12.2	rs2366293	*IKZF1*	2.33E-09	EU	G	0.15	1.23	(54)
165	7p12.2	rs4598207	*IKZF1*	4.12E-60	EAS, EU, TA	T	0.34	1.33	(26)
166	7p12.2	rs10239000	*IKZF1*	1.51E-25	EAS	A	0.20	1.19	(26)
167	7q11.22	rs13238909	*SPDYE21*	4.40E-08	TA	A	0.088	0.85	(55)
168	7q11.23	rs150518861	*METTL27*	4.10E-08	EU	A	0.005	1.66	(34)
169	7q11.23	rs10716716	*EIF4H*	8.49E-10	EAS	(T)13	0.19	1.15	(26)
170	7q11.23	rs372942110	*LAT2*	5.62E-09	EAS	(AATATATATA)2AA	0.006	2.29	(26)
171	7q11.23	rs530634980	*GTF2IRD1*	5.1E-18	EAS	T	0.001	2.02	(26)
172	7q11.23	rs73135369	*GTF2IRD1*	8.77E-14	TA, EAS	C	0.031	1.32	(29)
173	7q11.23	rs13244581	*GTF2IRD1*	1.59E-20	EAS	C	0.17	0.67	(26)
174	7q11.23	rs80346167	*GTF2IRD1*	3.26E-29	EAS	A	0.11	-	(45)
175	7q11.23	rs116991837	*GTF2IRD1*	3.82E-15	EAS	A	0.002	2.65	(26)
176	7q11.23	rs150724213	*GTF2I*	2.51E-15	EAS	A	0.005	3.88	(26)
177	7q11.23	rs117026326	*NCF1*	2.20E-298	EAS, EU	T	0.019	2.14	(26)
178	7q11.23	rs67955681	*GTF2IRD2*	3.68E-10	EAS	(A)15	0.45	0.85	(26)
179	7q11.23	rs79171842	*GTF2I*	3.02E-23	EAS	T	0.002	0.41	(26)
180	7q11.23	rs143176121	*GTF2I*	1.58E-66	EAS	C	0.002	0.29	(26)
181	7q11.23	rs587680541	*NCF1*	4.24E-62	EAS	CC	0.001	4.14	(26)
182	7q11.23	rs794368	*HIP1*	1.54E-26	EAS	A	0.46	1.19	(45)
183	7q11.23	rs145931380	*HIP1*	2.25E-15	EAS	(T)14	0.15	1.38	(56)
184	7q11.23	rs77009341	*HIP1*	6.39E-62	EAS	C	0.004	2.09	(26)
185	7q11.23	rs4573208	*HIP1*	1.53E-15	EAS	A	0.075	1.17	(26)
186	7q11.23	rs146063533	*HIP1*	9.44E-16	EAS	T	0.001	1.61	(26)
187	7q32.1	rs41298401	*IRF5*	5.04E-45	EAS, MA, TA	G	0.085	1.29	(26)
188	7q32.1	rs4728142	*IRF5*	3.38E-84	TA, AA, EAS, EU, MA	A	0.30	1.44	(24)
189	7q32.1	rs10488631	*IRF5*	9.00E-110	EU, AA, MA, TA	C	0.059	1.92	(25)
190	7q32.1	rs28364822	*TNPO3*	1.19E-17	EAS	A	0.001	1.67	(26)
191	8p23.1	rs6985109	*BLK*	2.51E-11	EU, TA	A	0.44	1.23	(20)
192	8p23.1	rs2736332	*BLK*	1.57E-70	EAS, AA, EAS, EU, TA	G	0.49	1.36	(26)
193	8p23.1	rs5889367	*ENSG00000284957*	1.34E-17	EAS	A	0.00001	0.69	(45)
194	8p23.1	rs880632	*FDFT1*	4.87E-08	EU	A	0.19	0.86	(57)
195	8p11.21	rs1804182	*PLAT*	3.48E-08	AA	A	0.005	1.94	(24)
196	8p11.21	rs2272736	*IKBKB*	6.37E-11	EAS	A	0.024	0.82	(26)
197	8q12.1	rs7829816	*LYN*	5.40E-09	EU	G	0.23	0.77	(20)
198	8q12.1	rs2953898	*RPS20*	4.43E-08	TA	T	0.087	0.84	(24)
199	8q13.3	rs142937720	*NCOA2*	2.27E-12	EAS	AG	0.19	0.89	(26)
200	8q13.3	rs17374162	*MSC*	3.02E-09	EAS	A	0.33	0.92	(26)
201	8q21.13	rs117821148	*PEX2*	4.8E-08	EAS	T	0.005	1.46	(33)
202	8q21.12	rs4739134	*ZC2HC1A*	3.47E-08	TA	T	0.25	1.12	(24)
203	8q22.3	rs13259960	*FLJ42969 (SLEAR)*	1.03E-11	EAS	G	0.14	1.35	(58)
204	8q24.21	rs16902895	*LINC00824*	1.48E-13	EAS	G	0.18	1.12	(26)
205	9p24.1	rs1887428	*JAK2*	2.19E-17	TA, EAS	G	0.31	1.16	(29)
206	9p21.3	rs7858766	*KLHL9*	2.25E-15	EAS	C	0.37	1.14	(26)
207	9q22.33	rs11788118	*NR4A3*	1.53E-08	TA	A	0.14	0.88	(24)
208	9q22.33	rs1405209	*NR4A3*	2.42E-08	TA	C	0.26	1.11	(22)
209	10p15.1	rs77448389	*GDI2*	7.30E-10	EAS	G	0.081	0.86	(26)
210	10p13	rs10795956	*CAMK1D*	2.87E-08	TA	A	0.31	1.11	(33)
211	10p13	rs7911501	*FAM107B*	2.0E-09	EAS	A	0.081	2.10	(32)
212	10q11.23	rs7072606	*WDFY4*	2.22E-12	EAS	C	0.14	0.88	(26)
213	10q11.23	rs7097397	*WDFY4*	2.23E-40	EAS, EU, TA	A	0.36	0.81	(26)
214	10q21.2	rs7902146	*ARID5B*	3.34E-12	EAS, EU, TA	C	0.35	0.90	(26)
215	10q21.2	rs10995261	*ADO*	2.57E-08	EAS, TA	T	0.18	0.91	(26)
216	10q22.1	rs780669	*SLC29A3*	4.83E-09	EAS, TA	C	0.34	1.16	(33)
217	10q22.1	rs10823829	*CDH23*	1.05E-09	EAS, TA	C	0.21	0.91	(26)
218	10q24.33	rs4917385	*NT5C2*	1.39E-08	MA	T	0.31	0.72	(59)
219	10q24.33	rs111447985	*STN1*	1.72E-08	EAS	A	0.027	1.17	(26)
220	10q25.2	rs58164562	*PDCD4*	3.14E-12	EAS	C	0.17	0.89	(26)
221	11p15.5	rs1131665	*IRF7*	9.36E-21	TA, EU	C	0.28	0.84	(24)
222	11p15.5	rs6598011	*TMEM80*	1.12E-09	EU	T	0.31	1.15	(24)
223	11p15.4	rs3750996	*STIM1*	1.89E-12	EAS	G	0.060	1.17	(26)
224	11p15.1	rs77885959	*GTF2H1*	3.16E-17	EAS	G	0.004	1.69	(26)
225	11p13	rs2732549	*CD44*	1.2E-23	EU, EAS, TA	G	0.22	1.24	(25)
226	11p13	rs11032994	*CD44*	1.12E-17	EAS	A	0.17	1.15	(26)
227	11q13.1	rs10896045	*RNASEH2C*	6.59E-26	EAS, EU, TA	A	0.48	1.17	(26)
228	11q13.1	rs1308020	*MAP3K11*	2.96E-19	TA, EAS	A	0.25	0.84	(40)
229	11q13.3	rs4930642	*TPCN2*	6.16E-13	EAS	A	0.19	1.15	(26)
230	11q13.4	rs3794060	*NADSYN1*	1.32E-20	EU	T	0.35	1.23	(25)
231	11q13.4	rs77971648	*FCHSD2*	3.16E-23	EAS	C	0.021	1.29	(26)
232	11q14.3	rs372605131	*NAALAD2*	1.2E-08	EAS	C	0.17	1.78	(32)
233	11q23.3	rs377392985	*DDX6*	2.86E-19	EAS	(A)9	0.31	1.16	(26)
234	11q23.3	rs4936441	*DDX6*	5.71E-16	EAS	C	0.20	0.82	(26)
235	11q24.3	rs9736939	*ETS1*	1.23E-58	EAS, EU, TA	A	0.33	1.27	(26)
236	11q24.3	rs7941765	*ETS1*	1.35E-10	EU, TA	T	0.28	1.14	(25)
237	11q24.3	rs684150	*FLI1*	4.32E-10	EAS	T	0.39	0.91	(26)
238	12p13.32	rs2540119	*CCND2*	3.51E-08	EAS	T	0.30	1.09	(26)
239	12p13.2	rs12822507	*CREBL2*	2.20E-08	EAS	G	0.36	0.86	(35)
240	12p13.1	rs4251697	*CDKN1B*	1.17E-43	EAS, TA	A	0.030	0.64	(26)
241	12p13.1	rs34330	*CDKN1B*	5.29E-22	TA, EAS	T	0.34	0.84	(60)
242	12q12	rs10506216	*PRICKLE1*	3.08E-08	EU	A	0.17	-	(61)
243	12q23.2	rs4622329	*DRAM1*	4.00E-15	EAS	G	0.48	1.12	(26)
244	12q23.3	rs6539078	*STAB2*	9.49E-14	EAS, TA	C	0.39	0.89	(26)
245	12q24.12	rs10774625	*SH2B3*	7.28E-12	TA, EU	A	0.15	0.83	(22)
246	12q24.12	rs77465633	*ATXN2*	6.99E-18	EAS	A	0.013	1.34	(26)
247	12q24.13	rs1131476	*OAS1*	1.25E-09	EAS	G	0.21	1.11	(26)
248	12q24.23	rs428073	*TAOK3*	2.52E-08	TA	C	0.27	1.12	(33)
249	12q24.31	rs3999421	*SPPL3*	1.29E-09	EAS	T	0.40	0.91	(26)
250	12q24.32	rs1059312	*SLC15A4*	1.48E-13	EU, EAS, TA	G	0.47	1.17	(25)
251	12q24.33	rs11059928	*SLC15A4*	1.61E-30	EAS, EU, TA	T	0.13	0.82	(26)
252	12q24.33	rs67438707	*ENSG00000256875*	5.66E-09	EAS	(CATCAC)3CATCA	0.37	0.88	(26)
253	13q14.11	rs57141708	*ELF1*	6.84E-22	EAS	A	0.11	1.18	(26)
254	13q14.2	rs76725306	*RCBTB1*	3.60E-08	TA	A	0.19	1.16	(22)
255	13q32.3	rs1885889	*TM9SF2*	2.05E-13	TA, EAS	A	0.23	0.87	(22)
256	13q33.3	rs145720245	*MYO16*	2.8E-10	EAS	A	0.002	4.00	(32)
257	14q13.2	rs8016947	*PPP2R3C*	1.08E-13	EAS	T	0.39	0.83	(23)
258	14q24.1	rs4902562	*RAD51B*	6.15E-10	EU, TA	A	0.41	1.14	(25)
259	14q31.3	rs11845506	*GALC*	5.00E-10	MA	A	0.043	0.20	(24)
260	14q32.32	rs12148050	*TRAF3*	2.57E-08	TA	G	0.49	0.91	(22)
261	14q32.33	rs2819426	*PLD4*	2.51E-30	EAS, TA	C	0.43	0.82	(26)
262	15q14	rs11073328	*FAM98B*	9.90E-15	EU	T	0.097	1.94	(31)
263	15q14	rs7170151	*RASGRP1*	3.20E-12	EAS, TA	T	0.48	1.11	(26)
264	15q14	rs12900339	*RASGRP1*	4.73E-10	EAS, TA	G	0.44	0.85	(45)
265	15q14	rs12900640	*RASGRP1*	2.42E-11	EAS	A	0.30	1.10	(26)
266	15q24.2	rs2289583	*ULK3*	6.22E-15	EU	A	0.18	1.19	(25)
267	15q24.1	rs11553760	*ULK3*	7.32E-10	EAS	T	0.089	1.11	(26)
268	15q24.3	rs869310	*TSPAN3*	1.90E-08	TA	G	0.25	0.88	(22)
269	15q26.2	rs8023715	*LINC02253*	1.20E-08	EU	A	0.074	1.81	(31)
270	15q26.3	rs35985016	*LRRK1*	1.95E-08	EAS	G	0.010	0.84	(26)
271	16p13.13	rs8054198	*CLEC16A*	1.79E-08	MA	T	0.069	0.36	(24)
272	16p13.13	rs12599402	*CLEC16A*	2.74E-22	TA, EAS, EU	T	0.50	0.84	(22)
273	16p13.13	rs35032408	*CLEC16A*	2.84E-08	EAS	G	0.14	0.69	(45)
274	16p12.2	rs79401250	*PRKCB*	1.48E-12	EAS	G	0.058	1.17	(26)
275	16p11.2	rs534645300	*ZNF629*	2.68E-09	EAS	(T)15	0.23	0.81	(26)
276	16p11.2	rs1143679	*ITGAM*	3.60E-90	TA, AA, EU, MA	A	0.085	1.76	(62)
277	16p11.2	rs2359661	*ITGAX*	4.47E-08	EU	G	0.43	1.37	(63)
278	16q12.1	rs11288784	*HEATR3*	2.38E-10	EAS	(T)12	0.26	0.90	(26)
279	16q12.2	rs9934578	*CHD9*	4.86E-08	TA	T	0.24	1.15	(33)
280	16q13	rs223881	*COQ9*	5.87E-16	TA, EAS	T	0.45	0.87	(40)
281	16q13	rs2731783	*CSNK2A2*	1.08E-09	TA	A	0.21	1.12	(55)
282	16q22.1	rs1749792	*ZFP90*	4.00E-11	TA, EAS	T	0.23	1.14	(24)
283	16q23.2	rs11376510	*LINC01229*	2.23E-10	EAS	(T)14	0.25	0.90	(26)
284	16q24.1	rs13332649	*IRF8*	2.12E-18	EU, TA	G	0.11	1.34	(25)
285	16q24.1	rs447632	*IRF8*	7.23E-28	EAS, TA	G	0.47	0.85	(26)
286	16q24.1	rs11117432	*IRF8*	1.27E-32	EAS, EU, TA	A	0.087	0.73	(26)
287	16q24.2	rs933717	*FBXO31*	2.36E-10	EAS	T	0.41	0.13	(64)
288	17p13.2	rs2286672	*PLD2*	2.93E-09	EU	T	0.19	1.25	(25)
289	17p13.1	rs61759532	*ACAP1*	2.79E-11	EAS, EU	T	0.099	1.24	(26)
290	17p11.2	rs35966917	*TNFRSF13B*	4.66E-09	EAS	G	0.41	0.91	(26)
291	17q12	rs4252665	*IKZF3*	1.96E-12	TA, EU	T	0.016	1.46	(24)
292	17q21.31	rs12952708	*ARHGAP27*	3.70E-08	EU	T	0.39	1.16	(34)
293	17q21.33	rs2671655	*ZNF652*	4.60E-08	EAS	C	0.12	1.09	(26)
294	17q25.1	rs8072449	*GRB2*	1.19E-11	TA, EU	G	0.40	0.84	(24)
295	17q25.3	rs113417153	*PGS1*	1.90E-08	EAS	T	0.12	0.89	(26)
296	18q22.2	rs763361	*CD226*	3.03E-12	TA, EAS	C	0.47	0.88	(22)
297	18q23	rs118075465	*NFATC1*	1.16E-10	EAS	A	0.11	1.14	(26)
298	19p13.3	rs2238577	*CFD*	1.83E-14	EAS	T	0.30	0.89	(26)
299	19p13.3	rs4807205	*DOT1L*	8.17E-09	TA	G	0.45	1.12	(42)
300	19p13.3	rs5826945	*C3*	9.67E-11	EAS	T	0.13	0.84	(26)
301	19p13.2	rs3093030	*ICAM1*	4.88E-08	TA	T	0.32	1.16	(65)
302	19p13.2	rs74908652	*ICAM1*	2.28E-09	EU	C	0.090	0.83	(24)
303	19p13.2	rs34725611	*TYK2*	4.93E-23	EU, TA	G	0.27	0.78	(24)
304	19p13.2	rs34536443	*TYK2*	2.43E-25	TA, EU	C	0.010	0.47	(24)
305	19p13.2	rs55882956	*TYK2*	1.23E-16	EAS	A	0.008	0.67	(26)
306	19p13.11	rs2362475	*KLF2*	2.00E-09	EAS	A	0.28	0.85	(42)
307	19p13.11	rs2384991	*IQCN*	4.95E-08	TA	C	0.31	1.11	(22)
308	19p13.11	rs11673604	*SSBP4*	4.21E-12	EAS, TA	C	0.48	1.14	(26)
309	19p13.11	rs12461589	*ANKRD27*	5.00E-10	EAS, TA	T	0.062	0.90	(26)
310	19p13.33	rs33974425	*TEAD2*	4.40E-12	EAS, TA	CA	0.32	1.12	(26)
311	19p13.33	rs7251	*IRF3*	4.40E-08	TA	G	0.49	0.88	(66)
312	19q13.41	rs3794986	*SIGLEC6*	1.46E-14	EAS	G	0.44	0.89	(26)
313	19q13.41	rs4801882	*SIGLEC14*	1.86E-18	EAS	A	0.37	0.88	(26)
314	19q13.42	rs56154925	*PPP6R1*	4.93E-23	TA	T	0.17	0.88	(24)
315	20p13	rs6074813	*SIRPB1*	3.23E-08	TA	G	0.43	1.12	(22)
316	20q13.12	rs4810485	*CD40*	9.95E-09	TA, EU	T	0.24	1.43	(24)
317	20q13.13	rs11697848	*RNF114*	1.40E-11	EU	T	0.039	2.12	(31)
318	22q11.21	rs4819670	*USP18*	5.53E-11	EAS	T	0.36	1.15	(26)
319	22q11.21	rs4821116	*UBE2L3*	8.86E-46	EAS, EU, TA	T	0.23	1.24	(26)
320	22q13.1	rs61616683	*SYNGR1*	5.73E-10	EAS	T	0.34	0.79	(45)
321	22q13.1	rs137956	*GRAP2*	5.00E-08	TA	C	0.28	0.88	(24)
322	Xp22.2	rs6641111	*PRPS2*	3.27E-25	EAS	G	0.25	1.19	(26)
323	Xp22.2	rs3853839	*TLR7*	2E-19	TA, EAS	G	0.40	1.25	(67)
324	Xp21.2	rs887369	*TASL*	5.26E-10	EU	A	0.10	1.15	(25)
325	Xp11.22	rs13440883	*GPR173*	7.53E-09	TA	C	0.33	1.16	(68)
326	Xp11.21	rs5914778	*NBDY*	5.26E-12	EAS	A	0.30	1.35	(69)
327	Xq28	rs143181706	*MAMLD1*	3.7E-08	EAS	T	0.11	1.50	(50)
328	Xq28	rs5987175	*SSR4*	1.21E-09	TA	C	0.33	0.85	(68)
329	Xq28	rs1059702	*IRAK1*	1.30E-27	TA, EAS, EU	A	0.37	1.43	(70)
330	Xq26	rs5945199	*G6PD*	2.90E-12	EAS	A	0.21	0.78	(26)

The most statistically significant (lead) variant with p<5×10^−8^ in at least one published study is presented with its odds ratio (OR) for minor allele in the ancestry (or population) with the lowest published probability and global minor allele frequency (MAF) from the 1000 Genomes Project (78). The ancestry with the lowest published probability is listed first when significant in multiple ancestries. Each locus is labeled with the closest expressed gene or a gene nominated by functional studies or computational prediction. Separate loci for neighboring variants are designated if there is evidence in the literature that they are separable or if their alleles have r^2^<0.2 in the subject population. TA, transancestral or mixed across ancestries; EAS, East Asian; EU, European or European-American; AA, African-American; MA, mixed American (including Native American ancestry). (Supplementary Material Table S1 presents the 760 published variants associated with SLE at p <5 × 10^−8^, which have been expanded from the 330 leading variants that define the 330 risk loci. Supplementary Material Table S3 presents the 16,318 variants from the r^2^>0.8 expansion of 760 variants in those populations in which the significant association at 5 × 10^−8^ was found).

**TABLE 2:** Systemic lupus erythematosus (SLE) risk loci variant distribution compared with genome composition.

	Global genome (EU + EAS)	SLE leading variants (n = 330)			SLE all published variants (n = 760)			SLE LD expanded all published variants (n = 16,318)		
Variant	%	%	OR	p-value	%	OR	p-value	%	OR	p-value
Non-synonymous	0.25%	7.90%	34.31	5.6E-170	6.59%	28.19	4.2E-267	1.15%	4.63	1.5E-114
Synonymous	0.27%	2.13%	7.91	1.3E-10	1.71%	6.34	3.5E-14	0.69%	2.54	2.5E-24
Intron	41.09%	52.28%	1.57	3.7E-05	56.65%	1.87	3.0E-18	52.13%	1.56	6.9E-180
UTR	0.73%	5.17%	7.37	4.5E-21	4.22%	5.95	2.8E-29	2.24%	3.10	9.6E-111
Intergenic and others	57.65%	32.52%	0.35	2.9E-20	30.83%	0.33	1.5E-50	43.79%	0.57	7.7E-280

The comparison of the physical genomic distribution of DNA sequence function from the entire human genome (78), as at present understood to the positions of the 360 lead variants, the 760 published variants at p <5× 10–8, and the 16,318 variants after expansion of the 760 variants to all variants in disequilibrium at r2 > 0.8. To calculate the odds ratios (OR) and probabilities (p), a denominator of 1 million was used for the variants in the genome.

**TABLE 3:** Ancestry biased systemic lupus erythematosus (SLE) risk loci comparing EAS and EU.

Locus	Leading SNP	Population	Minor allele frequency (MAF)		
			EU	EAS	Ratio
1p13.2	rs2476601	EU	0.094	0	-
7q11.23	rs77009341	EAS	0	0.018	-
11q13.4	rs77971648	EAS	0	0.104	-
17q12	rs4252665	EU	0.04	0	-
3p14.3	rs9311676	EU	0.414	0.001	414
12q24.12	rs10774625	EU	0.477	0.003	159
12p13.1	rs4251697	EAS	0.001	0.125	125
7p15.1	rs702814	EU	0.508	0.015	33.9
8p23.1	rs6985109	EU	0.528	0.021	25.1
2p13.1	rs6705628	EAS	0.009	0.156	17.3
11p15.5	rs1131665	EU	0.267	0.021	12.7
1q32.1	rs3024493	EU	0.166	0.028	5.9
1q32.1	rs2297550	EAS	0.113	0.519	4.6
Xp22.2	rs3853839	EAS	0.171	0.777	4.5
17p11.2	rs35966917	EAS	0.16	0.592	3.7
2p22.3	rs13385731	EAS	0.058	0.173	3
2p14	rs268134	EU	0.253	0.088	2.9
16q24.1	rs13332649	EU	0.198	0.073	2.7
3q26.2	rs10936599	EAS	0.243	0.578	2.4
15q14	rs7170151	EAS	0.251	0.561	2.2
16q13	rs223881	EAS	0.209	0.467	2.2
19p13.2	rs34725611	EU	0.263	0.529	2
11q23.3	rs377392985	EAS	0.22	0.433	2
1q23.3	rs1801274	EU	0.511	0.278	1.8
2p23.1	rs7579944	EAS	0.639	0.355	1.8
15q24.2	rs2289583	EU	0.313	0.179	1.7
19p13.11	rs11673604	EAS	0.354	0.225	1.6
7q11.23	rs794368	EAS	0.394	0.55	1.4
11q13.1	rs1308020	EAS	0.343	0.249	1.4
14q32.33	rs2819426	EAS	0.265	0.355	1.3
6p21.31	rs11755393	EU	0.354	0.458	1.3
16q24.1	rs447632	EAS	0.408	0.49	1.2
18q22.2	rs763361	EAS	0.527	0.612	1.2
16q22.1	rs1749792	EAS	0.209	0.187	1.1
6p21.32	rs660895	EAS	0.218	0.24	1.1
5p15.33	rs7725218	EAS	0.367	0.382	1.04

Minor allele frequency (MAF) from 1000 Genomes data (78) in European (EU) and East Asian (EAS) ancestral populations. The ratio is the MAF of EU/EAS if >1; otherwise, it is EAS/EU when >1. The population in which the genome-wide significant result was obtained is underlined and with a bold font.

**TABLE 4:** Pathways influenced by systemic lupus erythematosus (SLE)- associated genes.

Term	Overlap	Adjusted P-value	Odds ratio
Interleukin (IL)-12 complex	70/504	2.1E-29	7.2
IL23 complex	57/456	2.3E-21	6.2
IL35 complex	55/281	5.1E-21	6.5
B-cell receptor complex	60/566	3.5E-19	5.2
Interferon (IFN) regulatory factor 5 complex	22/59	2.7E-18	24.5
IFN-y signaling pathway	31/97	6.2E-18	12.3
Immune system	157/1943	4.0E-17	2.5
Immune system signaling by IFNs, interleukins, prolactin, and growth hormones	46/280	6.9E-15	5.2
Cytokine signaling in the immune system	76/702	7.8E-14	3.3
Th1- and Th2-cell differentiation	25/92	1.5E-13	9.7
Cell adhesion molecules	31/148	2.0E-13	6.9
MHC protein complex	14/21	3.0E-13	51.3
Intestinal immune network for IgA production	18/48	1.5E-12	15.4
IFN-a/p signaling	20/64	1.1E-11	11.7
NF-kB complex	61/864	2.6E-11	3.3
Adaptive immune system	71/733	1.3E-10	2.9
Fc receptor complex	25/185	8.4E-10	6.4
Antigen processing and presentation	19/78	1.1E-09	8.3
Th17-cell differentiation	22/107	1.2E-09	6.7
Positive regulation of T-cell activation	23/107	7.5E-09	7.1

Top 20 pathways based on p-value. For almost 700 traits associated with different pathways influenced by SLE locus-associated genes, see Supplementary Material Table S7 and S8.

## Key findings from the genome-wide genetic studies (GWASs) of SLE

1. Lupus is a complex genetic disease involving alleles with hundreds of variants. Approximately 330 lupus-predisposing loci have been discovered.
2. Lupus genetic loci are found to be associated with transcribed genes substantially more often than expected.
3. Genetic analysis of the available genetic association data implies that the mechanisms underlying lupus development are similar across both the European and East Asian ancestries.
4. There are a myriad of pathways and inflammatory components contributing to lupus from a genetic perspective.
