# Supplementary figures and images for: The 330 risk loci known for systemic lupus erythematosus (SLE): a review
Source: Front Lupus. Author manuscript; Available in PMC 2024 Dec 2. (PMC11609870; doi:10.3389/flupu.2024.1398035)

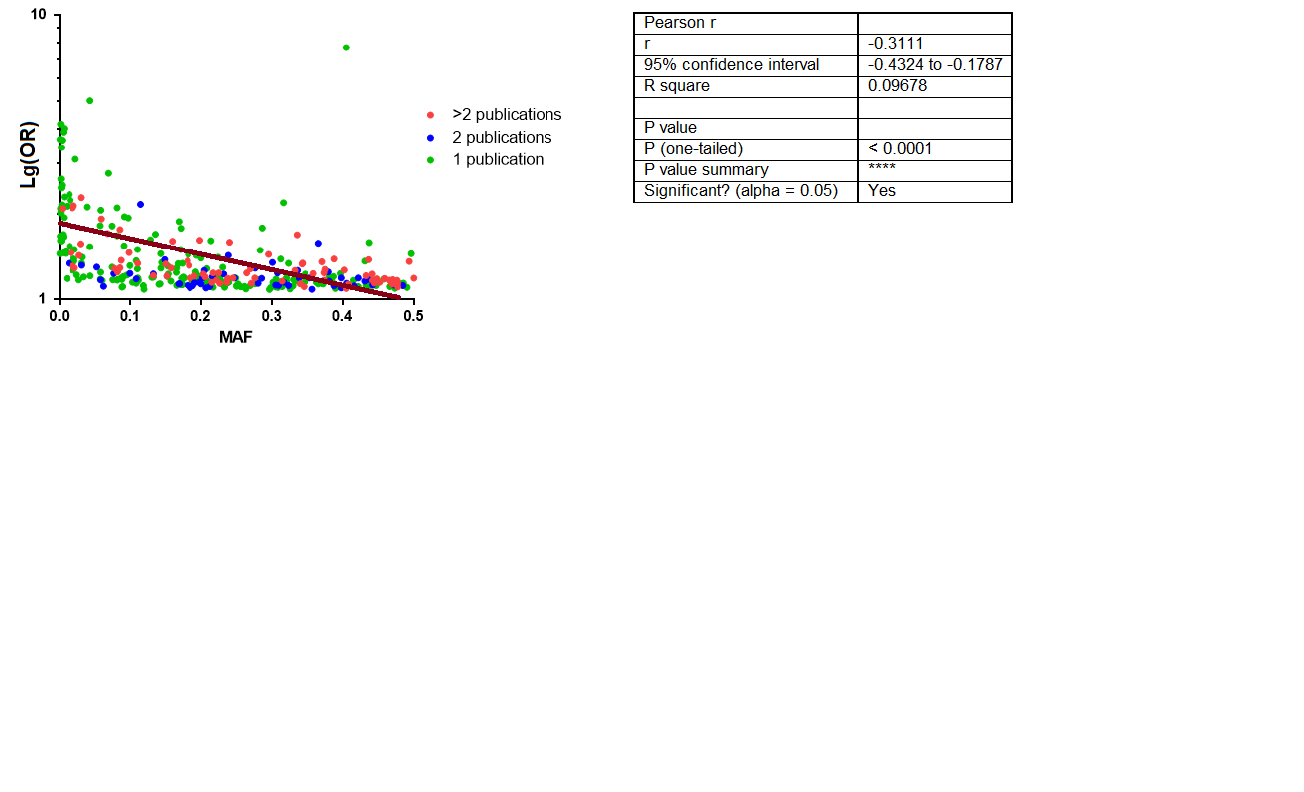

Supplement: Image 1 — SUPPLEMENTARY FIGURE 1 Minor allele frequency versus association effect size. [file NIHMS2037203-supplement-Image_1.png]

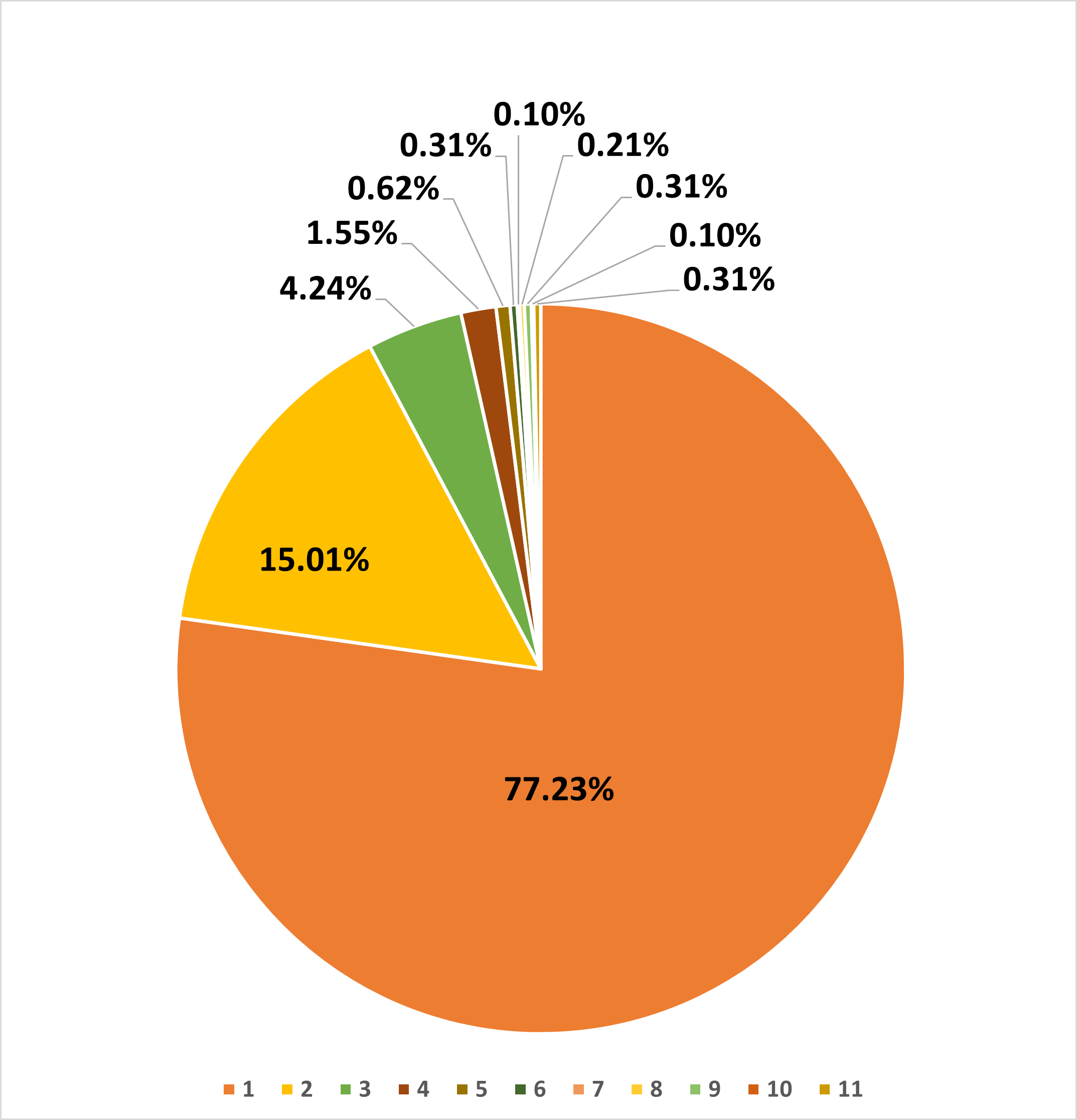

Supplement: Image 2 — SUPPLEMENTARY FIGURE 2 Number of loci influencing expression or function of plausible target genes. Of the Group 1 & 2 genes (n = 971) related to the 760 published variants associated with SLE at p < 5 × 10^−8^, one to 9 loci are related to individual genes, as indicated by the color coding in the legend. [file NIHMS2037203-supplement-Image_2.png]

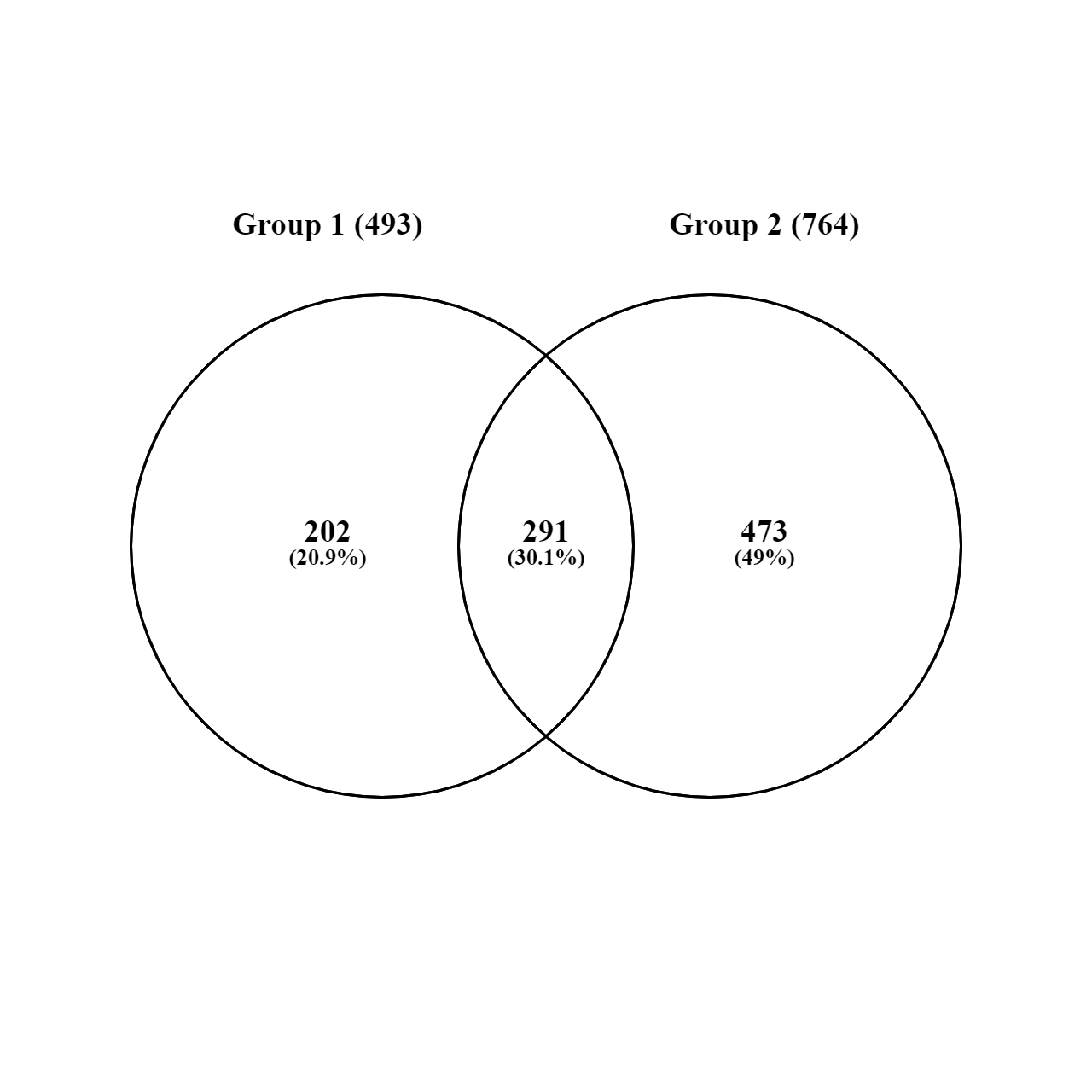

Supplement: Image 3 — SUPPLEMENTARY FIGURE 3 Genes implicated by the overlap of both Groups 1 and 2. [file NIHMS2037203-supplement-Image_3.png]
