# Supplementary material for: The 330 risk loci known for systemic lupus erythematosus (SLE): a review
Source: Front Lupus. Author manuscript; Available in PMC 2024 Dec 2. (PMC11609870; doi:10.3389/flupu.2024.1398035)
Supplement: Table 8 — SUPPLEMENTARY TABLE 8 Pathways associated with lupus loci. [file NIHMS2037203-supplement-Table_8.docx]

# **Supplementary Table 8. Pathways influenced by SLE-associated genes.** Almost 700 traits (Supplementary Table 7) associated with different but mostly related pathways influenced by SLE-loci associated genes are grouped below. Relative order is based on enrichment p value for one of the pathways of the group tested.

| Pathways influenced by SLE-associated genes |
| --- |
| *Cytokine-Mediated Signaling Pathways (IFN-γ in particular, IFN α/β, IL-12, IL-23, IL-35, IL-2, IL-4, IL-27, IL-20, IL-17, IL-3, IL-5, IL-6, IL-1, IL-10, IFN type III, IL-11, IL-13, TNF; thymic stromal lymphopoietin (TSLP) pathway; oncostatin M signaling) and related processes (interferon regulatory factor (IRF) complex; IRF3, IRF5, IRF7 complex; type I interferon receptor complex; IL3R complex; IL23R complex; IL2R complex; IL4R complex; IL28R complex; IL1R complex; TNF receptor superfamily complex; cytokine-cytokine receptor interaction; IL-12/STAT4; IL12R -> STAT; IL12R -> NF-kB/NFAT; IL12R -> NF-kB/NFATC; IL23R -> STAT3/NF-kB; IFNLR1-> STAT/NFKB; IFNG -> ARRB1/STAT1; IL-2/STAT5; IL2/PI3K; IL2R -> ELK/SRF/MYC; IL-6/JAK/STAT3; IL6R -> CEBP/ELK/SRF; IL6R -> STAT; IL6ST -> STAT5B; IL10R -> STAT; IL27R -> STAT; TLR4/5/7/9 -> NF-kB signaling; IL-22 soluble receptor signaling; NO2-dependent IL 12 pathway in NK cells; IL12 and Stat4 dependent signaling in Th1 development; TNFR2 signaling; TNF-α effects on cytokine activity, cell motility, and apoptosis; cellular response to TNF; eosinophil survival by cytokine signaling)  *B Cell Receptor (BCR) signaling (BCR complex; BCR activation by antigen; pre-B cell receptor complex; IgM B cell receptor complex; IgG B cell receptor complex; IgE B cell receptor complex)  *Immune and other cell differentiation (Th1, Th2, Th17, CD4+ and CD8+ α/β T Cell, Treg, memory T cell; hematopoietic progenitor cell; hematopoietic stem cell; vascular associated smooth muscle cell; erythrocyte; development of pulmonary dendritic cells and macrophage subsets; dendritic cells in regulating Th1 and Th2 development; selective expression of chemokine receptors during T-cell polarization; keratinocyte)  *Cell adhesion and migration (including leukocyte adhesion to endothelial cell; Integrin beta-2 pathway; Beta2 integrin cell surface interactions; Alpha-M beta-2 Integrin signaling; SELE (E selectin)-> ELK-SRF signaling; leukocyte transendothelial migration; regulation of blood vessel endothelial cell migration; integrin alpha4-beta1 complex; integrin alpha1-beta1 complex; integrin alphaX-beta2 complex; integrin alpha6-beta1 complex)  *Antigen processing and presentation by MHC class II in particular and MHC Class I (including antigen processing and presentation of endogenous peptide antigen; exogenous peptide antigen via MHC class I; cross presentation; MHC protein complex; MHC class II protein complex; MHC class I protein complex)  *Immunoglobulin production (including intestinal IgA production; circulating immunoglobulin complex; IgE immunoglobulin complex)  *NF-kB Signaling (including NF-kB Canonical Signaling; atypical NF-kappaB pathway; TNF-α signaling via NF-kB; NFKB signaling activation by blocking of tumor suppressors; NF-kappaB complex; IkappaB kinase complex)  *Immune response and inflammation (adaptive; Ig-mediated; Th1 type; T cell mediated; innate immune response; inflammatory response; immunoregulatory interactions between a lymphoid and a non-lymphoid cell; regulation of NK cell mediated immunity; anti-inflammatory function of macrophage M2 lineage; HMGB1 and IL1B in neuroinflammation; MC1R in anti-Inflammatory signaling; plasmin effects in inflammation; neutrophil recruitment and priming; response to lipopolysaccharide; acute phase proteins synthesis; lipopolysaccharide receptor complex)  *Fc Receptor mediated signaling (neutrophil activation via FCGR3B/adherence on endothelial cells; Fc-epsilon receptor I signaling in mast cells; Fc gamma R-mediated phagocytosis; FCERI Mediated Ca+2 Mobilization; Fc receptor complex; Fc-gamma receptor I complex; Fc-epsilon receptor I complex; Fc-gamma receptor III complex)  *Immune and other cell activation (T cell; B cell; T-cell independent B-cell; bystander B cell; proinflammatory monocyte, NK cells, NK activation through ITAM-containing receptors/through integrins and non-ITAM-containing receptors/through C-Type Lectin-like Receptors; basophil activation; mast-cell activation via IgE signaling; mast-cell activation without degranulation; mast-cell activation without degranulation through KIT/F2R signaling; NK T Cell; vascular endothelial cell activation by cytokines/by blood coagulation factors/ by growth factors ; neutrophil; platelet activation, signaling and aggregation)  *Fatty acid synthase complex  *PD-1 signaling *(PD-1 is an immune checkpoint that guards against autoimmunity via apoptosis)*  *Cytokine Production (IFN type I & II; IL-17; IL10; CHRM1 -> IL2; P2RY11/13/14 -> IL8/10; TNF production; regulation of NK cell cytokine production)  *T Cell Receptor (TCR) and co-stimulatory signaling (translocation of ZAP-70 to Immunological synapse; phosphorylation of CD3 And TCR zeta chains; TCR signaling in naive CD8+ and CD4+ T cells; CD28 co-stimulation; TCR -> NF-kB; TCR-> CREBBP; TCR -> NFATC; TCR -> AP-1 signaling; inhibition of TCR signaling by activated Csk; Lck and Fyn tyrosine kinases in initiation of TCR activation; gamma-delta TCR complex; pre-TCR complex; alpha-beta T cell receptor complex)  *Regulation of cytotoxicity (including T and NK cell mediated cytotoxicity; NK cell inhibitory receptor Signaling)  *Toll-like Receptor (TLR) signaling (including MyD88-dependent and independent; TLR4; TLR4 -> IRF; TLR2; TLR7; TLR3; TLR3 -> IRF; TLR9; TLR7/8; TRIF-dependent; endolysosomal TLR signaling; TRAF6-mediated IRF7 activation in TLR7/8 or 9 signaling; TRAF6 mediated induction of NFkB and MAPK upon TLR7/8 or 9 activation)  *JAK-STAT signaling pathway (receptor signaling via JAK-STAT; tyrosine phosphorylation of STAT; regulation of receptor signaling via STAT; STAT5 activation; regulatory circuits of the STAT3 signaling pathway)  *Immune cell&other proliferation and hemopoiesis (B cells; T cell; NK Cell, lymphocyte; vascular smooth muscle cell/pericyte migration and proliferation)  *Osteoclast differentiation  *Regulation of apoptosis (regulation of B cell apoptosis; regulation of apoptosis by IL-5, IL-4 and TCR; MEF2D role in T-cell apoptosis; endothelial cell apoptotic process; BCL-2 complex; Bcl-2 family protein complex)  *Vitamin D Receptor Pathway  *Complement (including activation of C3 and C5; classical; lectin pathway; MPB-related complement activation; complement cascade activation by pentraxins) and coagulation cascades (hemostasis)  *C-type lectin receptor (CLR) signaling  *Mitogen-Activated Protein Kinase (MAPK) signaling pathway (MAPK1 (ERK2) activation; regulation of MAP kinase activity; regulation of ERK1 (MAPK3) and ERK2 cascade)  *Pattern Recognition Receptor (PRP) Signaling Pathway (cytosolic PRP; RIG-I-like receptor signaling; RIG-I/MDA5 (DDX58/IFIH1) signaling; RIG-I/MDA5-mediated induction of interferon-alpha/beta pathways; RIG-I Signaling; Dectin-1 (CLEC7A) Signaling; NOD-like receptor (NLR) signaling)  *AHR Signaling (in Th17 cells; M1 macrophages; Tr1 cells; AHR in intestinal cell antimicrobial barrier maintenance)  *Protein phosphorylation and dephosphorylation, signaling by receptor tyrosine kinases (regulation of peptidyl-tyrosine phosphorylation; protein-tyrosine phosphatase 1B (PTP1B, PTPN1) signaling; activation of Src by protein tyrosine phosphatase alpha; protein tyrosine kinase activity; protein kinase binding; effects of calcineurin in keratinocyte differentiation; protein tyrosine phosphatase complex)  *Stem cell factor receptor (c-Kit) signaling (SCF-KIT; Kit receptor signaling; KIT -> STAT signaling; SCF/SKP2 Complex)  *Control of immune tolerance by vasoactive intestinal peptide  *PDGF signaling pathway (PDGFR -> AP-1/MYC; PDGFR -> STAT signaling; PDGFR-beta signaling)  *STING complex  *Ubiquitinilation (LUBAC complex; ubiqubiquinol and ubiquinoine biosynthesis; uitinilation of target proteins by E3 ubiquitin ligases)  *BAT3 complex  *Ire1 complex  *Regulation of apoptotic cell clearance  *Endosomal/vacuolar pathway (MyD88 dependent cascade initiated on endosome)  *TRAF3-dependent IRF activation pathway  *Regulation of nuclease activity (regulation of ribonuclease activity)  *CD40L signaling pathway (CD40/CD40L signaling)  *Cellular senescence and autophagy (autophagosome organization)  *Antigen Receptor-Mediated Signaling Pathway  *Fibrin Complement Receptor 3 Signaling Pathway  *FLT3 signaling  *G alpha q pathway; G alpha 13 pathway  *Activation of IRF3/IRF7 (mediated by TBK1/IKK epsilon; by TICAM1; TRAF6-mediated IRF7 activation)  *Protein processing in endoplasmic reticulum  *Mitophagy (receptor mediated mitophagy)  *CXCR4 signaling pathway  *CSF3 (G-CSF) signaling  *PI3K-Akt signaling (PI3K/AKT/mTOR - VitD3 signaling; PI5P, PP2A And IER3 regulate PI3K/AKT signaling)  *Signal regulatory protein (Endosomal/vacuolar) family interactions  *GM-CSF-mediated signaling (CSF2 (GM-CSF) -> STAT signaling)  *AP-1 transcription factor network  *Regulation of phagocytosis (role of phospholipids in phagocytosis)  *Ras signaling (Ras-GAP signaling; regulation of Ras family activation; Ras protein signal transduction; activation of RAS in B cells; erythropoietin activates RAS)  *Regulation of transcription (regulation of DNA/nucleic acid-templated transcription; regulation of NFAT transcription factors; calcineurin-regulated NFAT-dependent transcription in lymphocytes; regulation of nuclear beta catenin signaling and target gene transcription)  *Phosphatidylglycerol biosynthesis  *Pathways of nucleic acid metabolism and innate immune sensing (cytosolic DNA-sensing pathway)  *Gastrin signaling  *RANKL/RANK signaling (RANKL regulation of apoptosis and immune response)  *FoxO family signaling pathway  *Mucin production in goblet and mucous cells (mucin production in goblet airway epithelial cells)  *Neutrophil degranulation (neutrophil degranulation via FPR1/IL8)  *TYROBP causal network in microglia  *miR-517 relationship with ARCN1 and USP1  *Angiopoietin receptor Tie2-mediated signaling  *Erythropoietin (EPO) signaling  *V(D)J recombination activation  *Cell cycle (cyclin D associated events in G1; mitotic G2/M transition checkpoint)  *SHP2 (PTPN11) signaling  *UrokinaseR signaling (UrokinaseR -> ELK/SRF signaling; UrokinaseR -> STAT signaling)  *Growth hormone receptor signaling (Growth Factor Receptor Binding  *Synthesis of prostaglandin (PG)  *Regulation of glucose transmembrane transport  *Synapse pruning  *CTLA4 inhibitory signaling  *Peripheral T-cell tolerance  *Oxidative damage and DNA damage response (ATM-dependent DNA damage response)  *Leptin signaling (SOCS3 and PTPN1 in hypothalamic neuron insensitivity to insulin and leptin)  *ERBB2/3 (HER2/3)-> EP300/ETS/ETV/SP1 signaling  *PTPRC (CD45) -> BCL6 Signaling  *Rho GTPase cycle  *AXL Receptor Tyrosine Kinase  *Protein transport (regulation of protein transport; intracellular protein transmembrane transport)  *Resolution of D-Loop structures (resolution of D-loop structures thru Holliday junction intermediates)  *Neurotrophin signaling (B Brain-derived neurotrophic factor (BDNF) signaling; p75 neurotrophin receptor signaling via NF-kB)  *Adenylyltransferase activity  *ROS in angiotensin mediated cardiovascular remodeling and hypertrophy  *Wnt signaling  *VEGFR signaling (VEGFA-VEGFR2 signaling; VEGFR -> STAT; VEGFR -> NFATC; VEGFR -> ATF/CREB/ELK-SRF signaling)  *AGE/RAGE pathway  *Cellular response to mechanical stimulus  *BRCA2 binding to PALB2  *Lipid and lipoprotein metabolism  *Prolactin receptor signaling  *RAC1/PAK1/p38/MMP2 pathway  *ER-phagosome pathway  *Nicotinamide nucleotide biosynthetic process  *Regulation of JNK cascade  *Alpha-synuclein signaling  *Peptidyl-Serine modification  *Lipoxin A4/FPR2-related neutrophil depression  *IKK complex recruitment by IRAK1  *Platelet-mediated interactions with vascular and circulating cells |

IFN – Interferon (type I IFN-α and β, type II IFN-γ); IL – Interleukin; natural killer (NK)
