# Supplementary material for: The 330 risk loci known for systemic lupus erythematosus (SLE): a review
Source: Front Lupus. Author manuscript; Available in PMC 2024 Dec 2. (PMC11609870; doi:10.3389/flupu.2024.1398035)
Supplement: Table 1 — SUPPLEMENTARY TABLE 1 The published 760 SLE risk variants at p < 5 × 10^−8^. [file NIHMS2037203-supplement-Table_1.docx]

Supplementary table 1. SLE associated variants identified in GWAS and candidate gene studies.

| N | Location | Causal variant (ref);  presence of TE | Variant | Position GRCh38.p13 | Variant type | Gene by position (Closets gene) | Aminoacid change | Candidate gene for loci (reference) | Open Target Genetics prediction (top 3 genes) | Major allele | Minor allele | Global MAF | Lowest P-value | Population | OR | OR Allele | Risk allele | References, best p value (others) |
| --- | --- | --- | --- | --- | --- | --- | --- | --- | --- | --- | --- | --- | --- | --- | --- | --- | --- | --- |
| 1 | 1p36.33 |  | **rs12093154** | 1243545 | missense | C1QTNF12(p) | A/V | C1QTNF12 (p):c^1^  SDF4 (p):e,d^1^  B3GALT6 (p):e,d^1^ | SDF4>C1QTNF12>B3GALT6 | G | A | 0.132 | 2.51E-09 | Mix | 0.835±0.03 | A | G | ^1^ |
| 2 | 1p36.23 |  | **rs3795310** | 8371547 | intron | RERE (p) |  | RERE (p):e,d^1^ | RERE>ENO1>SLC45A1 | T | C | 0.481 | 3.36E-08 | Mix | 0.881±0.023 | T | C | ^1^ |
| 3 | 1p36.11 | *TE* | rs113333331 | 24189898-24189905 | intergenic | IFNLR1 (p) |  | IFNLR1 (p):e↑^2^ | IFNLR1>GRHL3>NIPAL3 | (A)_8_ | (A)_7_ | 0.125 | 4.32E-08 | Mix | 1.13±0.02 | T (vs TA) | (A)_7_ | ^2^ |
|  |  |  | **rs4649203** | 24193430 | intergenic | IFNLR1 (p) |  |  | IFNLR1>GRHL3>STPG1 | A | G | 0.4313 | 9.9E-09 | EAS | 1.16 | G | G | ^3^ |
| 4 | 1p34.3 |  | **rs28411034** | 37811325 | 3’ UTR | MTF1 (p) |  | MTF1 (p):e,d^1^  INPP5B (p):e,d^1^ | FHL3>INPP5B>MTF1 | G | A | 0.209 | 1.50E-10 | Mix | 0.863±0.023 | A | G | ^1^ |
| 5 | 1p31.3 |  | **rs6702599** | 67359716 | intron | IL12RB2 (p) |  | IL12RB2 (p):e,d^1^ | IL12RB2>SERBP1>C1orf141 | C | A | 0.226 | 1.78E-08 | EU | 0.821±0.035 | C | A | ^1^ |
|  |  |  |  |  |  |  |  |  |  |  |  |  | 3.18E-09 | Mix | 0.836±0.03 | C | A | ^1^ |
| 6 | 1p31.3 |  | **rs3828069** | 67373890 | intron | IL12RB2 (p) |  |  | IL12RB2>SERBP1>C1orf141 | T | C | 0.1927 | 1.77E-09 | Mix | 0.85 (0.79-0.90) | G | T | ^4^ |
| 7 | 1p13.2 |  | rs6679677 | 113761186 | intergenic | RSBN1 (p) |  | PTPN22 (p):c^5^,e^1,6,7^,3D^6^,d^1^  BCL2L15 (p):d^1^  AP4B1 (p):e^6^,3D^6^  HIPK1 (p):e^6^,3D^6^  PHTF1 (p):e^4^ | DCLRE1B>PTPN22>PHTF1 | C | A | 0.0258 | 2.02E-23 | EU | 1.41 (1.32-1.51) | A | A | ^4^ (^5^) |
|  |  |  | **rs2476601** | 113834946 | missense | PTPN22 (p) | W/R |  | DCLRE1B>PTPN22>AP4B1 | G | A | 0.0274 | 1.1E-28 | EU | 1.43 (1.34-1.53) | A | A | ^5^ (^1,4,7–13^) |
|  |  |  |  |  |  |  |  |  |  |  |  |  | 2.93E-11 | Mix | 1.511 (1.338-1.706) | T | A | ^12^ (^13^) |
| 8 | 1p13.1 |  | **rs9651076** | 116500680 | intron | ENSG00000286276 (lncRNA) |  | CD58 (p):d^1^  ENSG00000224950 (AL390066.1) (lncRNA):d^1^ | CD58>ATP1A1>IGSF3 | A | G | 0.416 | 3.26E-13 | EAS | 1.117±0.015 | A | A | ^14^ |
|  |  |  | rs1016140 | 116533925 | intron | CD58 (p) |  |  |  | G | T | 0.292 | 2.95E-10 | EAS | 0.87±0.022 | T | G | ^1^ |
|  |  |  |  |  |  |  |  |  |  |  |  |  | 1.26E-08 | Mix | 0.893±0.02 | T | G | ^1^ |
| 9 | 1q23.1 |  | **rs116785379** | 157138367 | 5’ UTR | ETV3 (p) |  |  | ETV3>ETV3L>ARHGEF11 | G | C | 0.043 | 6.68E-16 | EAS | 1.211±0.024 | C | C | ^14^ |
| 10 | 1q23.1 |  | rs11264750 | 157527370 | intron | FCRL5 (p) |  | FCRL5 (p):d^1^  FCRL4 (p):d^1^ | FCRL5>FCRL2>FCRL4 | A | G | 0.101 | 8.70E-13 | Mix | 0.755±0.039 | G | A | ^1^ |
|  |  |  |  |  |  |  |  |  |  |  |  |  | 1.95E-12 | EAS | 0.751±0.041 | G | A | ^1^ |
|  |  |  | **rs112806509** | 157532226-157532235 | 3’ UTR | FCRL5 (p) |  |  | FCRL5>FCRL4>FCRL3 | (T)_10_ | (T)_9_ | 0.132 | 1.76E-15 | EAS | 0.812±0.026 | A (vs AT) | (T)_10_ | ^14^ |
| 11 | 1q23.3 |  | **rs12120358** | 161474579 | intergenic | ENSG00000283360 (lncRNA) |  |  | FCGR2A>FCGR2B>HSPA6 | A | T | 0.172 | 2.96E-11 | EAS | 0.842±0.026 | A | T | ^14^ |
| 12 | 1q23.3 | rs1801274 (^15^) | rs7551957 | 161500252 | intergenic | FCGR2A (p) |  | FCGR2A (p):i^5^,c^5^,e^5^,d^1^,f(rs1801274-G(R): buinding of human IgG↓)^15^  FCGR2B (p):i,c,e^5^  FCGR3B (p):i^5^,c^5^,e^1,5^,d^1^ | FCGR2B>FCGR2A>ATF6 | T | C | 0.3632 | 4.27E-13 | Mix | 1.20 (1.14-1.26) | C | C | ^11^ |
|  |  |  |  |  |  |  |  |  |  |  |  |  | 2.07E-11 | EU | 1.21 | C | C | ^11^ |
|  |  |  | rs6671847 | 161509020 | intron | FCGR2A (p) |  |  | FCGR2B>FCGR2A>FCGR2C | G | A | 0.384 | 1.33E-12 | EU | 1.2 | A | A | ^5^ |
|  |  |  | **rs1801274** | 161509955 | missense | FCGR2A (p) | H/R ^(H131R)^ |  | FCGR2A>FCGR2B>FCGR2C | A | G | 0.4417 | 5.05E-15 | Mix | 1.167±0.02 | G | G | ^1^(^4^) |
|  |  |  |  |  |  |  |  |  |  |  |  |  | 1.19E-11 | EU | 1.209±0.028 | G | G | ^1^ (^4,5,9,11,16,17^) |
|  |  |  | rs12129787 | 161522797 | splice region | FCGR2A (p) |  |  | FCGR2A>FCGR2B>FCGR3B | C | T | 0.3287 | 1.37E-09 | EU | 1.22 (1.14-1.29) | A | T | ^4^ |
| 13 | 1q23.3 |  | **rs111994823** | 161500484 | intergenic | FCGR2A (p) |  |  | FCGR2A>FCGR2C>FCRLB | T | C | 0.032 | 3.41E-11 | EAS | 1.409±0.052 | T | T | ^14^ |
| 14 | 1q23.3 |  | **rs76107698** | 161600039 | intron | FCGR2C (p) |  |  | FCGR2C>FCGR3A>FCGR2B | G | C | 0.082 | 1.85E-30 | EAS | 0.788±0.021 | C | G | ^14^ |
| 15 | 1q23.3 |  | **low FCGR3B CN (<2)** | 82.5-kb repeat unit | repeat region | FCGR3B (p) |  |  |  |  |  |  | <1E-09 | Mix | 1.797 (1.562-2.068) | <2 | <2 | ^18^ |
|  |  |  |  |  |  |  |  |  |  |  |  |  | 2.70E-08 | EU | - | <2 | <2 | ^19^ |
| 16 | 1q23.3 |  | **rs75773410** | 161656008 | intergenic | FCGR2B (p) |  |  | FCGR2B>FCGR2C>HSPA6 | A | G | 0.165 | 3.84E-15 | EAS | 0.774±0.033 | A | G | ^14^ |
| 17 | 1q25.1 |  | rs1234313 | 173197108 | intron | TNFSF4 (p) |  |  | TNFSF4>TNFSF18>PRDX6 | G | A | 0.3684 | 4.37E-08 | EAS | 1.38 (1.32-1.44) | C | G | ^20^ |
|  |  |  | **rs1234314** ^[1]^ | 173208253 | intergenic | TNFSF4 (p) |  |  |  | C | G | 0.388 | 6.52E-28 | Mix | - | C | G | ^20^ |
|  |  |  |  |  |  |  |  |  |  |  |  |  | 5.84E-11 | EAS | 0.72 (0.67-0.87) | C | G | ^20^ |
|  |  |  |  |  |  |  |  |  |  |  |  |  | 2.43E-09 | EU | 0.83 (0.78-0.87 | C | G | ^20^ |
|  |  |  |  |  |  |  |  |  |  |  |  |  | 3.67E-09 | MA | 0.68 (0.55-0.81) | C | G | ^20^ |
|  |  |  | rs1234315 ^[2]^ | 173209324 | intergenic | TNFSF4 (p) |  |  |  | T | C | 0.4325 | 2.34E-26 | EAS | 1.37 (1.29-1.45) | A | T | ^21^ (^22^) |
|  |  |  | rs2205959 | 173222232 | intergenic | TNFSF4 (p) |  |  |  | A | G | 0.359 | 3.4E-12 | EAS | 1.39 (1.27-1.52) | G | G | ^23^ |
| 18 | 1q25.1 |  | rs1234317^[1]^ | 173218636 | intergenic | TNFSF4 (p) |  | TNFSF4 (p):i^5^,e^1^,^20^,d^1^ | TNFSF4>TNFSF18>PRDX6 | C | T | 0.1995 | 1.16E-28 | EAS | 1.37 (1.26-1.48) | T | T | ^20^ |
|  |  |  |  |  |  |  |  |  |  |  |  |  | 1.16E-28 | Mix | 1.38 (1.24-1.54) | T | T | ^20^ |
|  |  |  |  |  |  |  |  |  |  |  |  |  | 1.77E-11 | EU | 1.27 (1.20-1.34) | T | T | ^20^ |
|  |  |  |  |  |  |  |  |  |  |  |  |  | 3.65E-09 | MA | 1.5 (1.36-1.63) | T | T | ^20^ |
|  |  |  | **rs2205960** ^[1,2]^ | 173222336 | intergenic | PRDX6-AS1 (lncRNA) |  |  |  | G | T | 0.1809 | 2.52E-57 | Mix | 1.368±0.02 | T | T | ^1^ (^4,20,22^) |
|  |  |  |  |  |  |  |  |  |  |  |  |  | 3.16E-90 | EAS | 1.368±0.016 | T | T | ^14^ (^1,21,22,24–31^) |
|  |  |  |  |  |  |  |  |  |  |  |  |  | 3.84E-23 | EU | 1.29 (1.23-1.36) | A | T | ^4^ (^1,8,20^) |
|  |  |  |  |  |  |  |  |  |  |  |  |  | 1.7E-10 | MA | 1.56 (1.42-1.69) | T | T | ^20^ |
|  |  |  | rs76413021 | 173237158 | non coding transcript exon | PRDX6-AS1 (lncRNA) |  |  |  | G | A | 0.07129 | 3.26E-13 | EAS | 1.52 (1.36-1.71) | A | A | ^32^ |
|  |  |  | rs12039904 | 173243134 | intron | PRDX6-AS1 (lncRNA) |  |  |  | C | T | 0.1841 | 3.13-23 | Mix | 1.31 (1.24-1.38) | T | T | ^11^ |
|  |  |  |  |  |  |  |  |  |  |  |  |  | 7.20E-13 | EU | 1.27 | T | T | ^11^ |
|  |  |  |  |  |  |  |  |  |  |  |  |  | 1.47E-12 | EAS | 1.41 | T | T | ^11^ |
|  |  |  | rs844648 | 173254724 | intron | PRDX6-AS1 (lncRNA) |  |  | TNFSF4>DARS2>TNFSF18 | G | A | 0.4535 | 3.06E-08 | EAS | 0.46 (0.54-0.74) | G | A | ^29^ |
|  |  |  | rs844649 | 173255204 | intron | PRDX6-AS1 (lncRNA) |  |  |  | T | C | 0.291 | 1.24E-13 | EAS | 1.50 (1.34-1.67) | C | C | ^33^ |
|  |  |  | rs704840 | 173257056 | intron | PRDX6-AS1 (lncRNA) |  |  |  | T | G | 0.2907 | 3.12E-19 | EU | 1.22 (1.17-1.27) | G | G | ^5^ (^9^) |
|  |  |  | rs10489265 | 173266926 | intron | PRDX6-AS1 (lncRNA) |  |  | TNFSF4>TNFSF18>PRDX6 | A | C | 0.1997 | 1.00E-10 | EU | - | C | C | ^16^ |
|  |  |  | rs10912578 | 173282717 | intron | PRDX6-AS1 (lncRNA) |  |  | TNFSF4>PRDX6>TNFSF18 | G | A | 0.383 | 4.16E-19 | EU | 1.27 | A | A | ^5^ (^11^) |
|  |  |  |  |  |  |  |  |  |  |  |  |  | 1.98E-08 | EAS | 1.28 | - | A | ^11^ |
| 19 | 1q25.1 |  | **rs117278480** | 173241650 | intron | PRDX6-AS1 (lncRNA) |  |  | TNFSF4>TNFSF18>PRDX6 | A | G | 0.014 | 2.23E-52 | EAS | 0.649±0.028 | A | G | ^14^ |
| 20 | 1q25.1 |  | rs12736195 | 173338762 | intron | PRDX6-AS1 (lncRNA) |  | PRDX6 (p):e↓^34^  TNFSF4(OX40L) (p): e^34^ | PRDX6>TNFSF4>SLC9C2 | T | C | 0.1727 | 7.31E-11 | MA | 0.66 (0.59-0.74) | C | T | ^35^ |
|  |  |  | rs10798269 | 173340574 | intron | PRDX6-AS1 (lncRNA) |  |  |  | G | A | 0.3774 | 4.04E-10 | EU | 0.83 (0.78-0.88) | A | G | ^36^ |
|  |  |  | rs17346550 | 173346486 | intron | PRDX6-AS1 (lncRNA) |  |  |  | T | C | 0.1677 | 5.59E-10 | MA | 0.66 (0.58-0.74) | C | T | ^35^ |
|  |  |  | rs1539255 | 173353521 | intron | PRDX6-AS1 (lncRNA) |  |  |  | C | T | 0.3802 | 1.60E-19 | Mix | 0.84 (0.81-0.87) | T | C | ^4^ |
|  |  |  |  |  |  |  |  |  |  |  |  |  | 2.37E-12 | EU | 0.85 (0.81-0.89) | T | C | ^4^ |
|  |  |  | rs10753074 | 173377204 | intron | PRDX6-AS1 (lncRNA) |  |  |  | T | C | 0.3714 | 9.54E-13 | EU | 0.84 (0.81-0.88) | C | T | ^4^ (^5^) |
|  |  |  | rs4916342 | 173378698 | intron | PRDX6-AS1 (lncRNA) |  |  |  | A | G | 0.2997 | 8.22E-09 | EAS | 0.75 (0.67-0.82) | G | A | ^32^ |
|  |  |  | rs10798275 | 173381232 | intron | PRDX6-AS1 (lncRNA) |  |  |  | A | G | 0.299 | 2.39E-13 | EAS | 0.75 (0.7-0.81) | G | A | ^27^ |
|  |  |  | **rs2039982** | 173390048 | intron | PRDX6-AS1 (lncRNA) |  |  |  | T | C | 0.374 | 1.85E-37 | EAS | 1.242±0.017 | T | T | ^14^ |
|  |  |  | rs1418190 | 173392840 | intron | PRDX6-AS1 (lncRNA) |  |  |  | T | C | 0.3361 | 2.14E-22 | Mix | 1.223±0.021 | T | T | ^1^ |
|  |  |  |  |  |  |  |  |  |  |  |  |  | 1.13E-15 | EAS | 1.268±0.03 | T | T | ^1^ (^31^) |
|  |  |  |  |  |  |  |  |  |  |  |  |  | 7.08E-09 | EU | 1.182±0.029 | T | T | ^1^ |
|  |  |  | rs4916219 | 173404044 | intron | PRDX6-AS1 (lncRNA) |  |  | PRDX6>DARS2>RABGAP1L | C | T | 0.3197 | 7.77E-09 | EAS | 0.80 (0.7-0.86) | A | C | ^31^ |
| 21 | 1q25.1 |  | **rs549669428** | 174925885 | intron | RABGAP1L (p) |  | KIAA0040 (p):d^1^  RABGAP1L (p):d^1^ | MRPS14>RABGAP1L>CACYBP | T | G | 0.320 | 4.53E-08 | Mix | 0.839±0.032 | G | T | ^1^ |
| 22 | 1q25.3 | rs17849501^(37)^  rs17849502^(38)^ | rs17484292 | 183330915 | intron | NMNAT2 (p) |  | NCF2 (p):c^4,5^,d^1^,m^38^,f (rs17849502-T: ROS production↓)^38^  SMG7 (p):e^5,37^ | SMG7>NCF2>NMNAT2 | T | A | 0.0188 | 1.48E-39 | EU | 1.77 (1.63-1.93) | T | A | ^4^ |
|  |  |  |  |  |  |  |  |  |  |  |  |  | 9.97E-38 | Mix | 1.59 (1.40-1.79) | T | A | ^4^ |
|  |  |  | rs12146097 | 183360126 | intron | NMNAT2 (p) |  |  | SMG7>NMNAT2>NCF2 | C | T | 0.0551 | 6.40E-13 | Mix | 1.40 (1.58-3.08) | T | T | ^39^ |
|  |  |  |  |  |  |  |  |  |  |  |  |  | 1.50E-10 | EU | 1.38 (1.25-1.53) | T | T | ^39^ (^40^) |
|  |  |  | rs17849502 | 183563445 | missense | NCF2 (p) | H/Q |  | NCF2>SMG7>ARPC5 | G | T | 0.0182 | 1.15E-57 | EU | 2.11 | T | T | ^5^ (^37,38,41^) |
|  |  |  |  |  |  |  |  |  |  |  |  |  | 5.87E-11 | MA | 1.95 (1.59-2.38) | T | T | ^4^ (^37^) |
|  |  |  | **rs17849501** | 183573188 | synonymous | NCF2 (p) |  |  |  | C | T | 0.0182 | 3.45E-88 | EU | 2.10 (1.95-2.26) | T | T | ^5^ (^9,11^) |
|  |  |  |  |  |  |  |  |  |  |  |  |  | 3.34E-10 | MA | 2.02 | A | T | ^37^ (^4^) |
| 23 | 1q25.3 | rs2275675^(39)^ | rs2702178 | 183483410 | intron | SMG7 (p) |  | SMG7 (p):e↓^39^ | NCF2>NMNAT2>SMG7 | G | A | 0.3872 | 2.4E-08 | EU | 1.23 (1.14-1.32) | A | A | ^39^ |
|  |  |  | rs66977652 | 183571081-183571082 | intron | NCF2 (p) |  |  | NCF2>SMG7>NMNAT2 | AA | A | 0.486 | 7.76E-11 | EAS | 0.907±0.015 | T (vs TA) | A | ^14^ |
|  |  |  | **rs10911363** | 183580622 | intron | NCF2 (p) |  |  | NCF2>SMG7>ARPC5 | G | T | 0.3744 | 2.52E-17 | Mix | 1.17 (1.13-1.22) | A | T | ^4^ |
|  |  |  |  |  |  |  |  |  |  |  |  |  | 1.18E-13 | EU | 1.19 (1.14-1.25) | A | T | ^4^ (^42^) |
| 24 | 1q25.3 |  | 1:183524640:I (b37)*** *(****rs2308167?)*** | 183555505 | intergenic | NCF2 (p) |  |  |  | *(TGTT?)* | *(TGTTTGTT?)* | EU: 0.108; AS:0.104^11^ | 4.21E-10 | EU | 1.37 | I | *(TGTTTGTT?)* | ^11^ |
| 25 | 1q25.3 |  | **rs13306575** | 183563302 | missense | NCF2 (p) | R/W | NCF2 (p):m^37^ | NCF2>SMG7>ARPC5 | G | A | 0.0180 | 1.00E-09 | MA | 1.71 (1.44-2.03) | A | A | ^4^ (^35^) |
|  |  |  |  |  |  |  |  |  |  |  |  |  | 2.28E-14 | EAS | 1.311±0.035 | A | A | ^14^ |
| 26 | 1q25.3 |  | **rs35937854** | 183566954 | missense | NCF2 (p) | V/A | NCF2 (p):m^37^ | NCF2>SMG7>ARPC5 | A | G | 0.0136 | 1.49E-09 | AA | 2.34 | G | G | ^37^ |
| 27 | 1q25.3 |  | **rs41263646** | 183597997 | intergenic | NCF2 (p) |  |  | NCF2>RGL1>SMG7 | C | T | 0.1096 | 1.54E-08 | EU | 0.78 (0.71-0.85) | A | C | ^4^ |
| 28 | 1q25.3 | *TE* | **rs10911628** | 184680369 | intergenic | EDEM3 (p) |  |  | EDEM3>NIBAN1>C1orf21 | C | A | 0.0913 | 2.30E-13 | EU | 1.954 (2.16-1.770) | A | A | ^43^ |
| 29 | 1q31.2 |  | **rs1547624** | 192574707 | intron | ENSG00000285280 (lncRNA) |  | RGS1 (p):e,d^1^ | RGS1>RGS2>UCHL5 | T | A | 0.227 | 4.55E-08 | EAS | 1.172±0.029 | T | T | ^1^ |
| 30 | 1q31.3 |  | **rs34889541** | 198625639 | intergenic | PTPRC (p) |  | PTPRC (p):e,d^1^ | ATP6V1G3>PTPRC>NEK7 | G | A | 0.0765 | 2.44E-12 | Mix | 0.81 (0.76-0.86) | A | G | ^11^ |
|  |  |  |  |  |  |  |  |  |  |  |  |  | 2.96E-10 | EAS | 0.78 (0.72-0.84) | A | G | ^11^ |
|  |  |  | rs4143303 | 198701340 | intron | PTPRC (p) |  |  | PTPRC>ATP6V1G3>NEK7 | G | A | 0.074 | 3.70E-09 | EAS | 0.88±0.022 | A | G | ^14^ |
| 31 | 1q32.1 |  | **rs3806357** | 202010327 | 5’ UTR | ELF3 (p); ELF3-AS1 (lncRNA) |  |  | RNPEP>TIMM17A>GPR37L1 | G | A | 0.088 | 4.25E-09 | EAS | 1.106±0.017 | A | A | ^14^ |
| 32 | 1q32.1 |  | rs4844538 | 206469377 | intergenic | IKBKE (p) |  | IKBKE (p):i^11^,e↓^1,11^d^1^  MAPKAPK2 (p): e,d^1^ | IKBKE>SRGAP2>DYRK3 | A | T | 0.391 | 2.11E-10 | EAS | 1.109±0.016 | A | A | ^14^ |
|  |  |  | **rs2297550** | 206470429 | intergenic | IKBKE (p) |  |  | IKBKE>DYRK3>EIF2D | C | G | 0.2376 | 6.22E-13 | Mix | 1.187±0.024 | G | G | ^1^ (^11^) |
|  |  |  |  |  |  |  |  |  |  |  |  |  | 1.57E-08 | EAS | 1.179±0.029 | G | G | ^1^ |
| 33 | 1q32.1 |  | **rs529561493** | 206561820 | intron | RASSF5 (p) |  |  | IL19>FCMR>RASSF5 | A | C | 0.0004 | 9.8E-09 | EAS | 3.66 (2.35-5.71) | A | A | ^24^ |
| 34 | 1q32.1 | rs3122605^(44)^ | rs3024505 | 206766559 | intergenic | IL10 (p) |  | IL10 (p):i^5^,d^1^,e↑^44^ | IL19>FCMR>IL24 | G | A | 0.0863 | 4.64E-09 | EU | 1.17 (1.11-1.24) | T | A | ^5^ (^8,42^) |
|  |  |  | **rs3024493** | 206770623 | intron | IL10 (p) |  |  | FCMR>IL24>IL19 | C | A | 0.0815 | 2.35E-13 | Mix | 1.25 (1.16-1.35) | T | A | ^4^ |
|  |  |  |  |  |  |  |  |  |  |  |  |  | 3.39E-10 | EU | 1.20 (1.14-1.28) | T | A | ^4^ |
|  |  |  | rs3122605 | 206781696 | intron | IL19 (p) |  |  | IL10>IL24>FCMR | A | G | 0.0813 | 1.22E-11 | EU | 1.23 (1.16-1.30) | G | G | ^4^ (^44^) |
| 35 | 1q42.2 |  | **rs6586391** | 234498457 | intergenic | TARBP1 (p) |  | TARBP1 (p):e^2^ | TARBP1>IRF2BP2>TOMM20 | T | C | 0.206 | 4.13E-08 | Mix | 1.13±0.02 | C | C | ^2^ |
| 36 | 1q42.3 |  | **rs9782955** | 235876577 | intron | LYST (p) |  | LYST (p):i^5^,e^1,5,34^,d^1^ | LYST>NID1>GNG4 | C | T | 0.1567 | 1.25E-09 | EU | 1.16 (1.11-1.22) | C | C | ^5^ |
| 37 | 1q44 |  | **rs1780813** | 246280780 | intron | SMYD3 (p) |  |  | SMYD3>KIF26B>TFB2M | C | T | 0.0573 | 3.50E-08 | EU | 0.55 (0.31-0.79) | C | T | ^9^ |
|  |  |  |  |  |  |  |  |  |  |  |  |  |  |  |  |  |  |  |
| 38 | 2p25.1 |  | **rs75362385** | 7432948 | intron | ENSG00000229727 (lncRNA) |  |  | ID2>RNF144A | G | T | 0.171 | 8.40E-13 | EAS | 0.887±0.017 | T | G | ^14^ |
| 39 | 2p23.1 |  | **rs7579944** | 30222160 | intergenic | LBH (p) |  | YPEL5 (p):e,d^1^ | LBH>YPEL5>LCLAT1 | T | C | 0.4445 | 1.17E-15 | Mix | 1.155±0.018 | C | C | ^1 (11)^ |
|  |  |  |  |  |  |  |  |  |  |  |  |  | 1.02E-20 | EAS | 0.876±0.014 | T | C | ^14 (1,45)^ |
|  |  |  | rs906868 | 30225478 | intergenic | LBH (p) |  |  |  | G | T | 0.4625 | 7.71E-10 | EAS | 1.139 (1.079-1.203) | A | T | ^45^ |
| 40 | 2p23.1 |  | **rs17321999** | 30256991 | intron | LBH (p) |  | LBH (p):e↓^11^ | LBH>YPEL5>LCLAT1 | C | A | 0.1516 | 2.22E-16 | Mix | 0.83 (0.79-0.87) | A | C | ^11^ |
|  |  |  |  |  |  |  |  |  |  |  |  |  | 2.26E-09 | EU | 0.84 (0.79-0.89) | A | C | ^11^ |
|  |  |  |  |  |  |  |  |  |  |  |  |  | 9.55E-09 | EAS | 0.82 (0.77-0.88) | A | C | ^11^ |
| 41 | 2p22.3 | rs13385731^(46)^ | **rs13385731** | 33476823 | intron | RASGRP3 (p) |  | RASGRP3 (p):e,d^1^ | RASGRP3>FAM98A>TTC27 | T | C | 0.0773 | 1.29E-33 | EAS | 1.287±0.021 | T | T | ^14 (1,21)^ |
|  |  |  |  |  |  |  |  |  |  |  |  |  | 5.09E-23 | Mix | 0.744±0.003 | C | T | ^1^ |
|  |  |  | rs13425999 | 33477136 | intron | RASGRP3 (p) |  |  |  | C | T | 0.0647 | 8.68E-10 | EAS | 0.71 (0.64-0.79) | T | C | ^27^ |
| 42 | 2p16.1 |  | **rs1432296** | 60841032 | intron | REL-DT (lncRNA) |  |  | REL>SANBR>PUS10 | C | T | 0.0575 | 1.34E-08 | Mix | 1.18 (1.10-1.26) | A | T | ^4^ |
| 43 | 2p14 |  | **rs11126034** | 65353087 | intron | SPRED2 (p) |  |  | SPRED2>ACTR2>RAB1A | T | C | 0.439 | 2.60E-10 | EAS | 1.121±0.018 | T | T | ^14^ |
|  |  |  | rs11684155 | 65354166 | intron | SPRED2 (p) |  |  |  | C | T | 0.4377 | 2.61E-10 | EAS | 0.892±0.018 | T | C | ^14^ |
| 44 | 2p14 | rs6740462^(46)^ | **rs268134** | 65381229 | intron | SPRED2 (p) |  | SPRED2 (p):e^1^ | SPRED2>ACTR2>RAB1A | G | A | 0.1903 | 1.14E-10 | EU | 1.21 | G | G | ^5^ |
|  |  |  | rs268124 | 65427230 | intron | SPRED2 (p) |  |  |  | T | C | 0.3097 | 8.6E-09 | EU | 1.21 | - | T | ^11^ |
|  |  |  | rs6740462 | 65440138 | intron | LINC02934 (lncRNA) |  |  |  | A | C | 0.1777 | 1.76E-08 | EU | 0.83 (0.78-0.88) | C | A | ^9^ (^5^) |
| 45 | 2p13.1 | *TE* | rs940296 ^[3]^ | 73973706 | intron | DGUOK-AS1 (lncRNA) |  |  | MTHFD2>BOLA3>TET3 | T | C | 0.248 | 2.10E-08 | EAS | - | - | T | ^26^ |
|  |  |  | **rs4852324** ^[3]^ | 73975451 | intron | DGUOK-AS1 (lncRNA) |  |  | TET3>MTHFD2>BOLA3 | T | C | 0.2047 | 5.7E-14 | EAS | 0.79 | C | T | ^28^ (^26^) |
| 46 | 2p13.1 | rs146710425^(46)^  rs6705628^(47)^  rs2272165^(47)^  *TEx2* | **rs6705628** ^[3]^ | 73981235 | non coding transcript exon | DGUOK-AS1 (lncRNA) |  | DGUOK (p):e↓,3D^47^  DGUOK-AS1 (lncRNA):e,3D^47^  TET3 (p):e,d^1^ | DGUOK>STAMBP>BOLA3 | C | T | 0.1496 | 6.9E-17 | EAS | 0.75 | T | C | ^28^ (^1,11,47^) |
|  |  |  |  |  |  |  |  |  |  |  |  |  | 4.61E-09 | Mix | 0.845±0.029 | T | C | ^1^ |
|  |  |  | rs2272165 | 73986218 | 5’ UTR | TET3 (p); ENSG00000235499 (lncRNA) |  |  |  | G | A | 0.136 | 3.48E-10 | EAS | 0.79 (0.71-0.86) | A | G | ^47^ |
|  |  |  | rs10207954 | 73989388 | intron | TET3 (p) |  |  | DGUOK>BOLA3>MTHFD2 | A | T | 0.068 | 1.91E-12 | EAS | 1.145±0.019 | A | A | ^14^ |
|  |  |  | rs28421442 | 73992488 | intron | TET3 (p) |  |  |  | T | A | 0.1462 | 4.18E-09 | EAS | 1.44 | - | T | ^11^ |
| 47 | 2p13 |  | **rs73954925** | 111119597 | 3’ UTR | ACOXL (p) |  |  | BCL2L11>ACOXL>BUB1 | C | G | 0.111 | 5.11E-11 | EAS | 1.169±0.024 | C | C | ^14^ |
| 48 | 2q21.3 |  | rs2322659 | 135798089 | missense | LCT (p) | H/R | MCM6 (p):e^1^,^30^,d^1^ LCT-AS1 (LOC100507600) (lncRNA):d^1^ RAB3GAP1 (p):e,d^1^ | LCT>MCM6>UBXN4 | T | C | 0.4427 | 4.29E-13 | EAS | 1.153±0.020 | G | C | ^30^ |
|  |  |  | rs3754689 | 135833176 | missense | LCT (p) | A/T |  | DARS1>LCT>MCM6 | C | T | 0.355 | 3.17E-08 | EAS | 0.896±0.020 | A | C | ^30^ |
|  |  |  | **rs218174** | 135900775 | intergenic | DARS1 (p) |  |  | MCM6>DARS1>LCT | G | A | 0.309 | 1.83E-13 | EAS | 1.121±0.015 | A | A | ^14^ |
| 49 | 2q22.2 |  | **rs2381401** | 143263405 | intron | ARHGAP15 (p) |  | ARHGAP15(p):e,d^1^ | ARHGAP15>KYNU | C | T | 0.301 | 1.73E-09 | Mix | 1.149±0.023 | T | T | ^1^ |
| 50 | 2q24.2 | rs1990760^(48)^ | **rs2111485** | 162254026 | intergenic | FAP (p) |  | IFIH1 (p):c^5^,i^5^,e^5^,d^1^, f(changes in inflammation-related gene expression)^48^  GCA (p):e,d^1^ | FAP>GCA>IFIH1 | A | G | 0.3393 | 4.55E-12 | Mix | 0.88 (0.85-0.92) | A | G | ^4^ |
|  |  |  |  |  |  |  |  |  |  |  |  |  | 1.27E-11 | EU | 1.15 (1.11-1.20) | G | G | ^5^ (^4^) |
|  |  |  | rs11679244 | 162225885 | intron | FAP (p) |  |  |  | C | A | 0.250 | 3.04E-08 | EAS | 1.119±0.02 | A | A | ^14^ |
|  |  |  | rs1990760 ^[4]^ | 162267541 | missense | IFIH1 (p) | N/S |  | IFIH1>FAP>GCA | C | T | 0.3566 | 1.63E-08 | EU | 1.17 | T | T | ^42^ (^4,5^) |
| 51 | 2q24.2 | rs10930046^(48)^ | **rs10930046 ^[4]^** | 162281473 | missense | IFIH1 (p) | V/L | IFIH1 (p):f(apoptosis↑, changes in inflammation-related gene expression)^48^ | IFIH1>GCA>FAP | T | C | 0.1937 | 1.16E-08 (dominant model) | AA | 0.70 (0.62-0.79) | G | T | ^48^ |
| 52 | 2q24.2 | rs13023380^(48^**^)^** | **rs13023380 ^[4]^** | 162297853 | intron | IFIH1 (p) |  |  | GCG>IFIH1>GCA | G | A | 0.2216 | 5.20E-14 | Mix | 0.82 (0.78-0.87) | G | A | ^48^ |
|  |  |  |  |  |  |  |  |  |  |  |  |  | 9.52E-11 | EU | 0.84 (0.79-0.88) | G | A | ^48^ |
| 53 | 2q32.2 |  | **rs9630991** | 190567413 | intron | NEMP2-DT (lncRNA) |  | NAB1 (p):e,d^1^  NEMP2(TMEM194B) (p):e,d^1^  MFSD6 (p):e,d^1^ | NEMP2>NAB1>MFSD6 | G | A | 0.330 | 1.08E-13 | Mix | 0.851±0.022 | A | G | ^1^ |
|  |  |  |  |  |  |  |  |  |  |  |  |  | 6.73E-09 | EU | 0.851±0.028 | A | G | ^1^ |
| 54 | 2q32.2 | rs7574865^(46)^  rs11889341^(49)^ | rs3821236 | 191038032 | intron | STAT4 (p) |  | STAT4 (p):i^5^,e↑^1,50,51^, d^1^  STAT1: e↑^49,51^ | STAT4>GLS>STAT1 | G | A | 0.3031 | 5.96E-20 | Mix | 1.77 | A | A | ^50^ |
|  |  |  |  |  |  |  |  |  |  |  |  |  | 9.85E-13 | EAS | 1.45 | A | A | ^52^ |
|  |  |  |  |  |  |  |  |  |  |  |  |  | 8.49E-11 | EU | 1.49 | - | A | ^53^ (^50^) |
|  |  |  | rs3024866 | 191058115 | splice region | STAT4 (p); ENSG00000288064 (lncRNA) |  |  | STAT4>GLS>MFSD6 | G | A | 0.4964 | 2.31E-12 | Mix | 1.51 | C | G | ^50^ |
|  |  |  | rs10168266 | 191071078 | Intron | STAT4 (p) |  |  | STAT4>GLS>STAT1 | C | T | 0.2256 | 1.00E-16 | Mix | 1.56 (1.46-1.67) | T | T | ^54^ |
|  |  |  |  |  |  |  |  |  |  |  |  |  | 2.70E-16 | EAS | 1.59 (1.42-1.78) | T | T | ^55^ (^56,57^) |
|  |  |  |  |  |  |  |  |  |  |  |  |  | 1.38E-15 | EU | 1.49 (1.35-1.65) | A | T | ^56^ |
|  |  |  | **rs11889341** | 191079016 | Intron | STAT4 (p) |  |  |  | C | T | 0.24 | 5.89E-137 | Mix | 1.591±0.019 | T | T | ^1^ (^49^) |
|  |  |  |  |  |  |  |  |  |  |  |  |  | 5.59E-122 | EU | 1.73 (1.65-1.81) | T | T | ^1^ (^5,9,11,49,58^) |
|  |  |  |  |  |  |  |  |  |  |  |  |  | 4.64E-123 | EAS | 1.407±0.014 | T | T | ^14^ (^1,11,23,24,27,32,49^) |
|  |  |  |  |  |  |  |  |  |  |  |  |  | 5.85E-16 | MA | 1.48 (1.34-1.63) | A | T | ^4^ (^35^) |
|  |  |  | rs6736175 | 191081596 | intron | STAT4 (p) |  |  |  | C | T | 0.4163 | 9.17E-17 | EU | 1.24 | C | C | ^5^ |
|  |  |  | rs12612769 | 191089272 | intron | STAT4 (p) |  |  | STAT4>STAT1>MYO1B | A | C | 0.2093 | 2.37E-19 | EAS | 1.59 (1.43-1.75) | C | C | ^32^ |
|  |  |  | rs10931481 | 191090126 | intron | STAT4 (p) |  |  |  | A | G | 0.3924 | 1.74E-08 | EU | 1.312 (1.194-1.442) | T^#^ | G | ^59^ |
|  |  |  | rs4274624 | 191093930 | intron | STAT4 (p) |  |  | STAT4>GLS>STAT1 | T | C | 0.2608 | 8.48E-85 | Mix | 1.69 (1.60-1.78) | C | C | ^11^ |
|  |  |  |  |  |  |  |  |  |  |  |  |  | 9.73E-66 | EU | 1.75 | C | C | ^11^ (^49^) |
|  |  |  |  |  |  |  |  |  |  |  |  |  | 1.19E-22 | EAS | 1.57 | C | C | ^11^ |
|  |  |  | rs4853458 | 191094763 | intron | STAT4 (p) |  |  |  | G | A | 0.260 | 2.77E-12 | MA | 1.499 | - | A | ^49^ |
|  |  |  | rs7574865 | 191099907 | intron | STAT4 (p) |  |  | STAT4>STAT1>GLS | G | T | 0.2554 | 5.17E-42 | EAS | 1.51 (1.43-1.61) | A | T | ^21^ (^26,28,29,57,60,61^) |
|  |  |  |  |  |  |  |  |  |  |  |  |  | 1.40E-41 | EU | 1.57 (1.49-1.69) | T | T | ^8^ (^16,41,43,50,59,62–69^) |
|  |  |  |  |  |  |  |  |  |  |  |  |  | 4.44E-23 | Mix | 1.82 | T | T | ^50^ (^54^) |
|  |  |  |  |  |  |  |  |  |  |  |  |  | 2.41E-08 | MA | 2.1 | T | T | ^50^ |
|  |  |  | rs7568275 | 191101726 | intron | STAT4 (p) |  |  | STAT4>GLS>STAT1 | C | G | 0.2786 | 1.13E-77 | Mix | 1.42 (1.36-1.48) | C | G | ^4^ (^54^) |
|  |  |  |  |  |  |  |  |  |  |  |  |  | 4.51E-68 | EU | 1.55 (1.48-1.63) | C | G | ^4^ (^49^) |
|  |  |  |  |  |  |  |  |  |  |  |  |  | 4.00E-15 | MA | 1.46 (1.33-1.61) | C | G | ^4^ |
|  |  |  | rs10181656 | 191105153 | intron | STAT4 (p) |  |  |  | C | G | 0.2612 | 1.00E-16 | Mix | 1.49 (1.39-1.60) | - | G | ^54^ |
|  |  |  | rs7582694 | 191105394 | intron | STAT4 (p) |  |  |  | G | C | 0.273 | 4.30E-69 | EU | 1.56 (1.48-1.64) | C | C | ^4^ |
|  |  |  |  |  |  |  |  |  |  |  |  |  | 1.00E-16 | Mix | 1.49 (1.40-1.59) | C | C | ^54^ |
|  |  |  |  |  |  |  |  |  |  |  |  |  | 7.72E-16 | EAS | 1.5675 | C | C | ^70^ |
|  |  |  |  |  |  |  |  |  |  |  |  |  | 9.11E-16 | MA | 1.47 (1.34-1.61) | C | C | ^4^ |
|  |  |  | rs10174238 | 191108308 | intron | STAT4 (p) |  |  | STAT4>STAT1>NABP1 | A | G | 0.3407 | 1.00E-16 | Mix | 1.43 (1.36-1.54) | - | A | ^54^ |
| 55 | 2q32.3 |  | rs6715106 | 191048308 | intron | STAT4 (p); ENSG00000288064 (lncRNA) |  |  | STAT4>STAT1>NABP1 | A | G | 0.0759 | 8.33E-15 | EU | 0.67 (0.60-0.74) | G | A | ^4^ |
|  |  |  | rs7601754 | 191075725 | intron | STAT4 (p) |  |  |  | A | G | 0.3417 | 2.43E-10 | EAS | - | - | A | ^26^ (^28,29,60^) |
|  |  |  | **rs71030321** (rs71403211) | 191084261-191084274 | intron | STAT4 (p) |  |  | STAT4>STAT1>MYO1B | (A)_14_ | (A)_15_ | 0.098 | 3.16E-49 | EAS | 1.462±0.026 | T (vs TA) | (A)_14_ | ^14^ |
|  |  |  | rs56668168 | 191086636-191086655 | intron | STAT4 (p) |  |  |  | AGCCTTGTCTATTCT | - | 0.273 | 2.84E-18 | EAS | 0.61 (0.55-0.69) | D | AGCCTTGTCTATTCT | ^27^ |
| 56 | 2q33.1 |  | **rs7572733** | 198065082 | intron | PLCL1 (p) |  |  | PLCL1>BOLL>MARS2 | C | T | 0.386 | 1.25E-14 | EAS | 1.143±0.017 | T | T | ^14^ |
|  |  |  | rs11684176 | 198090050 | intron | PLCL1 (p) |  |  |  | C | T | 0.348 | 1.5E-14 | EAS | 1.146±0.018 | T | T | ^14^ |
| 57 | 2q33.2 | rs17268364^(71)^ | rs3087243 | 203874196 | intergenic | CTLA4 (p) |  | CTLA4 (p):e↓^1,71^,d^1^ | CTLA4>ICOS>CD28 | G | A | 0.369 | 2.29E-08 | Mix | 0.895±0.02 | A | G | ^1^ |
|  |  |  | **rs17268364** | 203913095 | intergenic | CTLA4 (p) |  |  |  | G | A | 0.422 | 7.02E-11 | EAS | 1.19 (1.13-1.26) | G | G | ^71^ |
| 58 | 2q34 |  | **rs7565158** | 212729246 | intron | ENSG00000273118 (lncRNA) |  |  | ERBB4>IKZF2 | G | T | 0.473 | 2.88E-10 | EAS | 1.096±0.015 | T | T | ^14^ |
| 59 | 2q34 |  | **rs3768792** | 213006985 | 3’ UTR | IKZF2 (p); ENSG00000273118 (lncRNA) |  | IKZF2 (p):i^5^,e^1^,d^1^ | IKZF2>SPAG16>ERBB4 | A | G | 0.2642 | 1.21E-13 | EU | 1.24 (1.17-1.31) | C | G | ^5^ (^9^) |
|  |  |  | rs2371790 | 213019942 | intron | IKZF2 (p) |  |  |  | G | A | 0.418 | 6.48E-09 | EAS | 1.107±0.018 | A | A | ^14^ |
|  |  |  | rs10048743 | 213025508 | intron | IKZF2 (p) |  |  | IKZF2>SPAG16>BARD1 | T | G | 0.2598 | 2.45E-10 | EU | 1.25 | G | G | ^5^ (^11^) |
| 60 | 2q36.3 |  | **rs5839171** | 226540919-226540928 | intergenic | MIR5702 (miRNA) |  |  | IRS1>RHBDD1 | A | - | 0.437 | 1.98E-08 | Egyptian | 0.63 (0.53-0.75) | G (vs GA) | A | ^72^ |
|  |  |  |  |  |  |  |  |  |  |  |  |  |  |  |  |  |  |  |
| 61 | 3p24.1 |  | **rs438613** | 28030595 | intron | LINC01967 (lncRNA) |  | LINC01980 (lncRNA):d^1^ | EOMES>CMC1>AZI2 | T | C | 0.357 | 1.32E-08 | Mix | 1.102±0.017 | C | C | ^1^ |
|  |  |  |  |  |  |  |  |  |  |  |  |  | 7.52E-09 | EAS | 0.920±0.014 | T | C | ^14^ |
| 62 | 3p14.3 |  | **rs180977001** | 58332737 | intergenic | PXK (p) |  |  | PXK>FLNB>PDHB | A | C | 0.0184 | 2.47E-08 | EU | 1.27 (1.17-1.39) | C | C | ^4^ |
| 63 | 3p14.3 |  | rs6445972 | 58335980 | intron | PXK (p) |  | ABHD6 (p):e↑^5,73^  PXK (p):e^1,5,34^,d^1^  BCR internalization↓^74^ | PXK>KCTD6>ACOX2 | T | C | 0.1528 | 4.62E-10 | EU | 0.81 (0.75-0.86) | C | T | ^74^ |
|  |  |  | *3:58363342:D (b37)*** (rs536644701?)* | 58377615 | intron | PXK (p) |  |  |  | ? | ? | 0.003^5^ | 1.59E-08 | EU | 1.17 | I | ? | ^5^ |
|  |  |  | rs6445975 | 58384450 | intron | PXK (p) |  |  | PXK>PDHB>RPP14 | T | G | 0.3427 | 5.27E-09 | EU | 1.20 (1.13-1.27) | G | G | ^36^ (^16,67^) |
|  |  |  | 3:58416772:I (b37)***  *(rs34834843?)* | 58431045 | intron | PDHB (p) |  |  |  | *(TT?)* | *(TTATT?)* | EU: 0.352; AS:0.003^11^ | 2.78E-08 | EU | 1.19 | I  (TTATT?) | (TTATT?) | ^11^ |
|  |  |  | rs9852465 | 58479456 | intergenic | KCTD6 (p) |  |  | PXK>KCTD6>RPP14 | A | G | 0.4056 | 1.97E-08 | Mix | 1.10 (1.06-1.15) | G | G | ^4^ |
|  |  |  | **rs9311676** | 58484624 | intergenic | KCTD6 (p) |  |  | PXK>KCTD6>ACOX2 | C | T | 0.2218 | 3.06E-14 | EU | 1.17 (1.13-1.22) | C | C | ^5^ |
| 64 | 3p13 |  | **rs7637844** | 72176765 | intron | LINC00877 (lncRNA); LINC00870 (lncRNA) |  |  | RYBP>GPR27>EIF4E3 | A | C | 0.236 | 1.28E-08 | EAS | 0.877±0.023 | A | C | ^14^ |
| 65 | 3q13.33 | rs61400421^(46)^  *TE* | rs12494314 | 119403973 | intron | ARHGAP31 (p) |  | CD80 (p):d^1^  ARHGAP31 (p):e,d^1^  POGLUT1 (p): e^1,27^,d^1^  B4GALT4 (p):e^27^ | TIMMDC1>POGLUT1>ARHGAP31 | T | C | 0.1899 | 1.01E-09 | EAS | 0.84 (0.80, 0.89) | G | T | ^31^ |
|  |  |  | rs2305249 | 119409551 | synonymous | ARHGAP31 (p) |  |  |  | G | A | 0.1877 | 1.64E-09 | EAS | 0.8 (0.74-0.86) | A | G | ^27^ |
|  |  |  | rs1132200 | 119431989 | missense | TMEM39A (p) | A/T |  | TIMMDC1>TMEM39A>POGLUT1 | C | T | 0.0887 | 8.62E-09 | Mix | 0.73 (0.59-0.89) | A | C | ^75^ |
|  |  |  | rs12636784 | 119455536 | Intron | TMEM39A (p) |  |  | TIMMDC1>POGLUT1>ARHGAP31 | A | G | 0.1653 | 1.01E-11 | Mix | 0.80 (0.74-0.86) | C | A | ^4^ |
|  |  |  |  |  |  |  |  |  |  |  |  |  | 6.24E-09 | EU | 0.82 (0.77-0.88) | C | A | ^4^ |
|  |  |  | rs1131265 | 119503609 | stop lost | TIMMDC1 (p) | */S |  |  | G | C | 0.2073 | 8.35E-16 | Mix | 0.843±0.021 | C | G | ^1^ |
|  |  |  |  |  |  |  |  |  |  |  |  |  | 1.37E-14 | EAS | 0.827±0.025 | C | G | ^1^ |
|  |  |  |  |  |  |  |  |  |  |  |  |  | 1.42E-09 | EU | 0.81 (0.76-0.87) | C | G | ^4^ |
|  |  |  | **rs79498479** (rs144104218) | 119518880-119518892 | intron | TIMMDC1 (p) |  |  | CD80>POPDC2>TIMMDC1 | AAACAAACAAACA | AAACAAACA | 0.206 | 1.89E-30 | EAS | 0.833±0.016 | A (vs AAAAC) | AAACAAACAAACA | ^14^ |
|  |  |  | rs6804441 | 119542097 | intron | CD80 (p) |  |  | TIMMDC1>POGLUT1>NR1I2 | A | G | 0.1867 | 2.5E-16 | EAS | 0.79 | G | A | ^28^ |
|  |  |  | rs2222631 | 119553544 | intron | CD80 (p) |  |  | NR1I2>ADPRH>PLA1A | A | G | 0.4401 | 4.5E-08 | EAS | 0.86 (0.81-0.91) | G | A | ^29^ |
| 66 | 3q25.33 |  | **rs77583790** | 159976265 | intron | IL12A-AS1 (lncRNA) |  |  | IL12A>SCHIP1>C3orf80 | G | A | 0.0026 | 6.49E-11 | EU | 2.15 | A | A | ^5^ |
| 67 | 3q25.33 | rs2936303^(46)^ | rs564799 | 160011200 | intron | IL12A-AS1 (lncRNA) |  | IL12A (p):i,e^5^ | IL12A>SCHIP1>C3orf80 | C | T | 0.2115 | 1.54E-09 | EU | 1.14 (1.09-1.18) | C | C | ^5^ |
|  |  |  | **rs564976** | 160011272 | intron | IL12A-AS1 (lncRNA) |  |  |  | G | A | 0.1975 | 2.2E-10 | EU | 0.87 (0.83-0.91) | T | G | ^4^ |
| 68 | 3q26.2 |  | **rs10936599** | 169774313 | synonymous | MYNN (p) |  | ACTRT3 (p):d^1^ | MYNN>ACTRT3>LRRC34 | C | T | 0.2706 | 1.92E-13 | Mix | 1.14 (1.10-1.18) | C | C | ^76^ (^1^) |
|  |  |  |  |  |  |  |  |  |  |  |  |  | 1.73E-09 | EAS | 1.124±0.019 | G | C | ^30^ (^27,76^) |
|  |  |  | rs1317082 | 169779797 | intron | MYNN (p) |  |  | ACTRT3>MYNN>LRRC34 | A | G | 0.267 | 1.62E-11 | EAS | 1.102±0.014 | A | A | ^14^ |
|  |  |  | rs6793295 | 169800667 | missense | LRRC34 (p) | S/G |  | LRRC34>ACTRT3>SEC62 | T | C | 0.4213 | 2.99E-09 | EAS | 1.130±0.021 | A | T | ^30^ |
| 69 | 3q28 |  | **rs6762714** | 188752450 | intron | LPP (p) |  | LPP (p):e^1^ | LPP>BCL6>TPRG1 | T | C | 0.3468 | 4.00E-15 | Mix | 1.16 (1.12-1.20) | T | T | ^11^ |
|  |  |  |  |  |  |  |  |  |  |  |  |  | 7.97E-10 | EU | 1.14 (1.09-1.19) | T | T | ^11^ |
|  |  |  |  |  |  |  |  |  |  |  |  |  |  |  |  |  |  |  |
| 70 | 4p16.3 |  | **rs3733345** | 960459 | 3’ UTR | DGKQ (p) |  | DGKQ (p):e↑^6^  GAK (p):e,d^1^  IDUA (p):e,d^1^ | IDUA>DGKQ>TMEM175 | T | G | 0.4722 | 2E-11 | Mix | 0.89 (0.85-0.92) | G | T | ^4^ |
|  |  |  | rs13101828 | 971932 | Intron | DGKQ (p) |  |  |  | A | G | 0.406 | 7.78E-10 | EAS | 0.91±0.015 | A | G | ^14^ |
|  |  |  | rs4690229 | 976936 | intron | DGKQ (p) |  |  | IDUA>DGKQ>SLC26A1 | A | T | 0.3604 | 1.62E-08 | EU | 1.13 (1.09-1.19) | T | A | ^4^ |
| 71 | 4p16.3 |  | **rs231694** | 2699117 | intron | FAM193A (p) |  |  | ADD1>NOP14>GRK4 | C | T | 0.207 | 9.71E-09 | EAS | 1.111±0.018 | T | T | ^14^ |
| 72 | 4p16.1 |  | **rs13116227** | 8556539 | intergenic | GPR78 (p) |  |  | GPR78>CPZ>ACOX3 | C | T | 0.173 | 3.05E-11 | EAS | 1.34 | T | T | ^77^ |
| 73 | 4p14 |  | **rs71196850** (rs113284964) | 40305571-40305572 | intergenic | GPR78 (p) |  |  | RHOH>N4BP2>CHRNA9 | CTTCCT | CT | 0.366 | 1.35E-16 | EAS | 1.134±0.015 | G (vs GCTTC) | CT | ^14^ |
| 74 | 4q12 |  | **rs2855772** | 54682309 | Intron | KIT (p) |  |  | KIT>SRD5A3>KDR | T | C | 0.200 | 1.21E-15 | EAS | 1.4 | C | C | ^77^ |
| 75 | 4q21.21 |  | **rs6533951** | 78723125 | intergenic | BMP2K (p) |  |  | BMP2K>ANXA3>PAQR3 | A | G | 0.466 | 1.25E-10 | EAS | 1.111±0.016 | A | A | ^14^ |
| 76 | 4q21.23 |  | rs11099582 | 83221591 | intergenic | COQ2 (p) |  |  | COQ2>PLAC8>HPSE | G | A | 0.3367 | 1.4E-09 | EAS | 1.103±0.016 | A | A | ^14^ |
|  |  |  | rs6841907 | 83225843 | intergenic | COQ2 (p) |  |  | COQ2>PLAC8>HPSE | T | C | 0.308 | 1.10E-09 | EAS | 0.906±0.016 | T | C | ^14^ |
| 77 | 4q21.3 | rs340626^(46)^ | **rs144261754** | 86983537-86983543 | intron | AFF1 (p) |  | AFF1 (p):e↑^55^ |  | (A)_7_ | AAA | 0.2125 | 3.45E-10 | EAS | 0.899±0.017 | T (vs TAAAA) | (A)_7_ | ^14^ |
|  |  |  | rs340630 | 87037243 | intron | AFF1 (p) |  |  | C4orf36>AFF1>KLHL8 | G | A | 0.4317 | 8.3E-09 | EAS | 1.21 (1.14-1.30) | A | A | ^55^ |
| 78 | 4q21.3 |  | **rs116940334** | 87023100 | intron | AFF1 (p) |  |  | AFF1>C4orf36>SLC10A6 | G | T | 0.034 | 3.15E-10 | EAS | 0.833±0.029 | T | G | ^14^ |
| 79 | 4q24 | *TE* | rs6856202 | 101795552 | Intron | BANK1 (p) |  | BANK1 (p):i^5^,e↑^1,5,34,78^,d^1^ BANK1 (p):isophorm expression (full-length↑, ∆2↓)^79^ | BANK1>PPP3CA | G | A | 0.383 | 3.76E-09 | EU | 1.19 | - | A | ^11^ |
|  |  |  | 4:102721293:D (b37)***  (rs11280314?) | 101800136 | Intron | BANK1 (p) |  |  |  | (GGGGGGAGGG/del(GGGGGGA)?) | (GGGGGGAGGGGGGAGGG?) | del EU:0.446; AS:0.390^11^ | 4.5E-10 | EU | 1.20 | del | (GGGGGGAGGG?) | ^11^ (^5^) |
|  |  |  | rs10028805 | 101816093 | intron | BANK1 (p) |  |  | BANK1>PPP3CA | G | A | 0.4441 | 2.67E-17 | Mix | 0.854±0.019 | A | C | ^1^ |
|  |  |  |  |  |  |  |  |  |  |  |  |  | 4.31E-17 | EU | 1.20 (1.15-1.25) | G | G | ^5^ (^9,78^) |
|  |  |  |  |  |  |  |  |  |  |  |  |  | 8.41E-11 | EAS | 0.854±0.024 | A | C | ^1^ |
|  |  |  | rs71597109 | 101819845 | intron | BANK1 (p) |  |  |  | C | T | 0.220 | 6.86E-14 | Mix | 0.790 (0.742-0.840) | T | C | ^78^ |
|  |  |  |  |  |  |  |  |  |  |  |  |  | 1.02E-08 | AA | 0.6786 | T | C | ^78^ |
|  |  |  | rs17266594 | 101829765 | intron | BANK1 (p) |  |  |  | T | C | 0.2186 | 4.74E-11 | Mix | 1.42 (1.28-1.58) | T | T | ^79^ (^80^) |
|  |  |  |  |  |  |  |  |  |  |  |  |  | 4.67E-09 | EAS | 0.606 (0.51-0.72) | C | T | ^81^ |
|  |  |  | rs10516487 | 101829919 | missense | BANK1 (p) | R/H |  |  | G | A | 0.2184 | 2.64E-13 | Mix | 1.317 (1.223-1.417) | G | G | ^80^ (^79,82^) |
|  |  |  |  |  |  |  |  |  |  |  |  |  | 3.58E-11 | EU | 0.85 (0.81-0.89) | T | G | ^4^ (^16^) |
|  |  |  | rs17200824 | 101831432 | intron | BANK1 (p) |  |  |  | A | G | 0.2202 | 9.24E-09 | AA | 0.679 (0.595-0.775) | G | A | ^78^ |
|  |  |  | rs4637409 | 101832251 | intron | BANK1 (p) |  |  |  | A | G | 0.2179 | 1.45E-17 | Mix | 0.84 (0.80-0.87) | C | A | ^4^ |
|  |  |  |  |  |  |  |  |  |  |  |  |  | 2.41E-11 | EU | 0.85 (0.81-0.89) | C | A | ^4^ |
|  |  |  | **rs4643809** | 101834942 | intron | BANK1 (p) |  |  |  | C | T | 0.448 | 3.53E-24 | EAS | 0.846±0.016 | T | C | ^14^ |
|  |  |  | rs7682827 | 101835908 | intron | BANK1 (p) |  |  |  | G | A | 0.4964 | 1.29E-09 | EAS | 0.83 (0.77-0.89) | A | G | ^27^ |
|  |  |  | rs4426778 | 101859567 | intron | BANK1 (p) |  |  |  | A | G | 0.4269 | 1.03E-12 | EU | 0.79 (0.74-0.84) | T | G | ^4^ |
| 80 | 4q25 |  | **rs956237** | 108125804 | intron | LEF1 (p) |  |  | LEF1>CYP2U1>PAPSS1 | G | A | 0.391 | 4.47E-11 | EAS | 1.107±0.015 | A | A | ^14^ |
| 81 | 4q25 |  | **rs58107865** | 108140462 | intron | LEF1 (p) |  |  | LEF1>RPL34>HADH | G | C | 0.058 | 6.57E-25 | EAS | 0.802±0.021 | C | G | ^14^ |
| 82 | 4q27 |  | **rs11724582** | 122470309 | intergenic | IL2 (p) |  |  | IL2>KIAA1109>ADAD1 | A | G | 0.168 | 1.71E-08 | Mix | 0.88 (0.84-0.93) | C | A | ^4^ |
|  |  |  | rs907715 | 122613898 | intron | IL21 (p) |  |  | IL21>KIAA1109>BBS12 | C | T | 0.366 | 2.17E-08 | Mix | 1.16 (1.10-1.22) | G | C | ^83^ |
| 83 | 4q35.1 |  | **rs10018951** | 183688220 | Intron | TRAPPC11 (p) |  |  | TRAPPC11>RWDD4>STOX2 | C | T | 0.1526 | 1.18E-14 | EAS | 1.31 | T | T | ^77^ |
|  |  |  |  |  |  |  |  |  |  |  |  |  |  |  |  |  |  |  |
| 84 | 5p15.33 |  | rs7726159 | 1282204 | intron | TERT (p) |  | TERT (p):e^1^ | TERT>CLPTM1L>SLC6A18 | C | A | 0.3235 | 2.11E-11 | EAS | 1.21 (1.14-1.28) | A | A | ^27^ (^1^) |
|  |  |  |  |  |  |  |  |  |  |  |  |  | 6.04E-11 | Mix | 1.134±0.019 | A | A | ^1^ |
|  |  |  | **rs7725218** | 1282299 | intron | TERT (p) |  |  |  | G | A | 0.408 | 2.47E-17 | EAS | 1.132±0.015 | A | A | ^14^ |
| 85 | 5p13.2 |  | **rs6871748** | 35885880 | intergenic | IL7R (p) |  | IL7R (p):e^1^,d^1^  CAPSL (p):e^1^,d^1^  CAPSL-DT (RP11-79C6.3) (lncRNA):d^1^  SPEF2 (p):d^1^ | IL7R>LMBRD2>SPEF2 | T | C | 0.173 | 3.96E-08 | Mix | 0.886±0.022 | C | T | ^1^ |
| 86 | 5q21.1 |  | rs6886392 | 100800161 | intergenic | ST8SIA4 (p) |  | ST8SIA4 (p):e^9^ | ST8SIA4>FAM174A | G | C | 0.233 | 4.08E-09 | Mix | 1.13 (1.08-1.18) | C | C | ^4^ |
|  |  |  | rs2544920 | 100805670 | intergenic | ST8SIA4 (p) |  |  |  | T | A | 0.389 | 8.74E-10 | EAS | 1.122±0.019 | A | A | ^14^ |
|  |  |  | **rs12153670** | 100836660 | intron | ST8SIA4 (p) |  |  | ST8SIA4>FAM174A>CHD1 | A | G | 0.236 | 7.65E-10 | EAS | 1.15±0.022 | G | G | ^84^ |
|  |  |  | rs55849330 | 100848943 | intron | ST8SIA4 (p) |  |  |  | C | A | 0.1807 | 4.9E-08 | EU | 1.16 (1.11-1.21) | A | A | ^9^ |
| 87 | 5q23.3 |  | **rs74989671** | 128398268 | intron | FBN2 (p) |  |  | FBN2>SLC27A6>SLC12A2 | A | G | 0.091 | 1.61E-08 | EAS | 1.5447 | G | G | ^70^ |
| 88 | 5q31.1 |  | **rs370449198** | 131784646-131784652 | intron | FNIP1 (p) |  |  | RAPGEF6>FNIP1>MEIKIN | (C)_7_ | (C)_8_ | 0.020 | 4.41E-08 | EAS | 0.721±0.060 | A (A vs AC) | (C)_8_ | ^14^ |
| 89 | 5q31.1 |  | **rs2549002** | 132493886 | intron | ENSG00000283782 (p) |  |  | RAD50>IRF1>P4HA2 | A | C | 0.434 | 2.40E-10 | EAS | 0.905±0.016 | A | C | ^14^ |
| 90 | 5q31.1 |  | **rs115267018** | 134085418 | intergenic | VDAC1 (p) |  |  | SKP1>TCF7>VDAC1 | G | C | 0.082 | 9.08E-11 | EAS | 0.85±0.025 | C | G | ^14^ |
| 91 | 5q31.1 |  | **rs244689** | 134087125 | intergenic | VDAC1 (p) |  |  | TCF7>UBE2B>SKP1 | G | A | 0.276 | 1.21E-20 | EAS | 1.29 (1.22-1.35) | A | A | ^85^ |
|  |  |  | rs244687 | 134087925 | intergenic | VDAC1 (p) |  |  | UBE2B>TCF7>SKP1 | G | A | 0.346 | 1.31E-08 | EU | 1.25 | - | A | ^11^ |
| 92 | 5q31.1 |  | rs4388254 | 134092910 | intergenic | VDAC1 (p) |  | TCF7 (p):i^5^,e^1^,d^1^  SKP1 (p):e^5^  UBE2B (p):e^1^,d^1^  CDKN2AIPNL (p):d^1^ | SKP1>TCF7>UBE2B | C | T | 0.238 | 1.50E-11 | Mix | 1.39 (1.26-1.53) | T | T | ^11^ |
|  |  |  |  |  |  |  |  |  |  |  |  |  | 3.71E-10 | EU | 1.47 | T | T | ^11^ (^5^) |
|  |  |  | rs6874758 | 134093501 | intergenic | VDAC1 (p) |  |  |  | G | C | 0.199 | 3.88E-14 | EAS | 1.238±0.028 | C | C | ^14^ |
|  |  |  | **rs7726414** | 134096143 | intergenic | VDAC1 (p) |  |  |  | C | T | 0.183 | 3.17E-16 | Mix | 1.317±0.034 | T | T | ^1^ |
|  |  |  |  |  |  |  |  |  |  |  |  |  | 4.44E-16 | EU | 1.45 (1.32-1.58) | T | T | ^5^ (^9^) |
|  |  |  |  |  |  |  |  |  |  |  |  |  | 1.66E-13 | EAS | 1.42 (1.28-1.53) | T | T | ^85^ (^1,27,86^) |
| 93 | 5q31.1 |  | **rs138305363** | 134379182 | intron | UBE2B |  |  | JADE2>PPP2CA>UBE2B | A | G | 0.002 | 4.07E-08 | EAS | 0.622±0.087 | A | G | ^14^ |
| 94 | 5q31.1 |  | **rs707149** | 151010955 | intergenic | GPX3 (p) |  |  | GPX3>ZNF300>ANXA6 | G | A | 0.397 | 3.58E-08 | EAS | 0.81 (0.75-0.88) | A | G | ^27^ |
| 95 | 5q31.1 |  | **rs2233302** | 151035537 | intron | TNIP1 (p) |  |  | GPX3>TNIP1>GM2A | C | G | 0.099 | 1.17E-08 | EAS | 0.76 (0.69-0.83) | G | C | ^27^ |
| 96 | 5q33.1 | rs10036748^(46)^ | rs7708392 | 151077924 | intron | TNIP1 (p) |  | TNIP1(ABIN1) (p):e↓^1,4,87^,i^5^,d^1^ | TNIP1>GPX3>ANXA6 | C | G | 0.4355 | 2.00E-24 | EU | 1.29 (1.23-1.35) | C | C | ^4^ (^8,88^) |
|  |  |  |  |  |  |  |  |  |  |  |  |  | 2.23E-11 | MA | 1.76 (1.50-2.07) | C | C | ^35^ (^4^) |
|  |  |  |  |  |  |  |  |  |  |  |  |  | 4.08E-09 | Mix | 1.13 (1.08-1.18) | C | C | ^4^ |
|  |  |  | rs6889239 | 151078210 | Intron | TNIP1 (p) |  |  |  | C | T | 0.4623 | 6.73E-25 | EU | 1.29 (1.23-1.35) | C | C | ^4^ (^5,11,42,87^) |
|  |  |  | **rs10036748** | 151078585 | intron | TNIP1 (p) |  |  |  | T | C | 0.4363 | 1.27E-45 | EU | 1.38 (1.32-1.45) | T | T | ^5^ (^1,4,9,11,89^) |
|  |  |  |  |  |  |  |  |  |  |  |  |  | 1.55E-34 | Mix | 1.46 (1.35-1.59) | A | T | ^4^ (^1,11^) |
|  |  |  |  |  |  |  |  |  |  |  |  |  | 5.98E-26 | EAS | 1.194±0.017 | T | T | ^14^ (^1,21,26,30^) |
|  |  |  |  |  |  |  |  |  |  |  |  |  | 9.56E-11 | MA | 1.72 (1.46-2.02) | T | T | ^35^ (^4^) |
|  |  |  | rs960709 | 151081488 | intron | TNIP1 (p) |  |  |  | G | A | 0.4343 | 1.15E-10 | EAS | 0.75 (0.69-0.82) | A | G | ^27^ |
| 97 | 5q33.3 |  | **rs2421184** | 159459931 | intron | LOC285626 (lncRNA); LINC01845 (lncRNA) |  |  | IL12B>ADRA1B>RNF145 | G | A | 0.353 | 4.67E-12 | EAS | 0.84 (0.80-0.88) | G | A | ^27^ (^14^) |
| 98 | 5q33.3 | rs2431697^(46)^ | rs4921283 | 160443604 | intergenic | PTTG1 (p) |  | MIR146A (MIR3142HG) (miRNA):e↓^4^,d^1^ | PTTG1>SLU7>ZBED8 | G | A | 0.499 | 6.76E-09 | EU | 0.85 | G | A | ^10^ |
|  |  |  | **rs2431697** | 160452971 | intron | MIR3142HG (lncRNA) |  |  | SLU7>ATP10B>PTTG1 | T | C | 0.3754 | 5.98E-29 | Mix | 0.784±0.022 | C | T | ^1^ (^11^) |
|  |  |  |  |  |  |  |  |  |  |  |  |  | 8.01E-28 | EU | 1.26 (1.21-1.31) | T | T | ^5^ (^1,4,9,11,16,67^) |
|  |  |  |  |  |  |  |  |  |  |  |  |  | 1.39E-28 | EAS | 1.241±0.019 | T | T | ^14^ (^1,11,31^) |
|  |  |  |  |  |  |  |  |  |  |  |  |  | 1.27E-12 | AA | 0.76 (0.70-0.82) | C | T | ^4^ |
|  |  |  | rs2431098 | 160460329 | intron | MIR3142HG (lncRNA) |  |  | PTTG1>ZBED8>SLU7 | A | G | 0.4681 | 3.29E-21 | Mix | 1.19 (1.14-1.23) | C | G | ^4^ |
|  |  |  |  |  |  |  |  |  |  |  |  |  | 4.03E-18 | EU | 1.25 | G | G | ^5^ (^4,11^) |
|  |  |  |  |  |  |  |  |  |  |  |  |  | 1.49E-08 | AA | 1.25 (1.16-1.36) | C | G | ^4^ |
| 99 | 5q33.3 | rs57095329^(90)^  rs2277920^(46)^ | **rs57095329** | 160467840 | intron | MIR3142HG (lncRNA) |  | MIR146A (MIR3142HG) (miRNA):e^90^ | ZBED8>PTTG1>SLU7 | A | G | 0.1428 | 2.74E-08 | EAS | 1.29 (1.18-1.40) | G | G | ^90^ |
|  |  |  |  |  |  |  |  |  |  |  |  |  |  |  |  |  |  |  |
| 100 | 6p25.3 |  | **rs9503037** | 243302 | intergenic | DUSP22 (p) |  | DUSP22 (p):e,d^1^ | DUSP22>IRF4>HUS1B | A | G | 0.200 | 1.36E-15 | EAS | 0.881±0.016 | A | G | ^14^ |
|  |  |  | rs6927090 | 252145 | intergenic | DUSP22 (p) |  |  | IRF4>DUSP22>HUS1B | C | T | 0.092 | 3.48E-10 | EAS | 1.233±0.033 | T | T | ^1^ |
| 101 | 6p24.3 |  | **rs2714333** | 7236387 | intergenic | RREB1 (p) |  |  | RREB1>SSR1>CAGE1 | C | T | 0.022 | 1E-08 | EAS | 3.11 (2.11–4.59) | T | T | ^24^ |
| 102 | 6p22.3 |  | **rs17603856** | 16630667 | intron | ATXN1 (p) |  |  | ATXN1>GMPR>STMND1 | T | G | 0.2288 | 3.27E-12 | Mix | 0.88 (0.85-0.91) | G | T | ^11^ |
|  |  |  |  |  |  |  |  |  |  |  |  |  | 3.34E-08 | EU | 0.89 (0.85-0.93) | G | T | ^11^ |
| 103 | 6p22.3 |  | **rs10807602** | [16968557](https://useast.ensembl.org/Homo_sapiens/Location/View?contigviewbottom=variation_feature_variation%3Dnormal%2Cseq%3Dnormal;db=core;r=6:16968507-16968607;source=dbSNP;v=rs10807602;vdb=variation;vf=172676291) | intergenic | STMND1 (p) |  |  | ATXN1>STMND1>RBM24 | C | T | 0.422 | 1.99E-08 | Mix | 1.11±0.02 | C | C | ^2^ |
| 104 | 6p22.3 |  | **rs10498722** | 25186284 | intron | CMAHP (pseudogene) |  |  | CARMIL1>H2BC5>RIPOR2 | C | T | 0.0741 | 2.87E-10 | EU | 1.30 (1.20-1.41) | A | T | ^4^ |
| 105 | 6p22.2 |  | **rs79774308** | 25410794 | intron | CARMIL1 (p) |  |  | CARMIL1>SCGN>H2BC1 | A | G | 0.008 | 5.76E-10 | EAS | 1.452±0.06 | A | A | ^14^ |
| 106 | 6p22.2 |  | rs35789010 | 25513951 | intron | CARMIL1 (p) |  | SLC17A4 (p):e^4^ | BTN3A2>H2BC5>BTN3A3 | G | A | 0.0186 | 4.59E-19 | EU | 1.46 (1.35-1.59) | A | A | ^4^ |
|  |  |  | rs4712969 | 25763964 | intron | SLC17A4 (p) |  |  |  | G | A | 0.0715 | 1.83E-22 | EU | 1.42 (1.32-1.52) | A | A | ^4^ |
|  |  |  | **rs36014129** | 25884291 | intergenic | SLC17A3 (p) |  |  | BTN3A2>BTN3A1>H2BC5 | G | A | 0.021 | 1.21E-24 | EU | 1.50 (1.39-1.62) | A | A | ^4^ |
| 107 | 6p22.2 |  | **rs9295676** | 25928148 | intron | SLC17A2 (p) |  |  | TRIM38>SLC17A3>CARMIL1 | G | T | 0.217 | 7.94E-11 | EAS | 1.105±0.015 | T | T | ^14^ |
| 108 | 6p22.1 |  | **rs10946940** | 27592808 | intergenic | TRNA_Met (tRNA) |  |  | ZSCAN26>ZSCAN31>ZSCAN9 | A | G | 0.496 | 8.20E-09 | EU | 0.690 (0.73-0.649) | G | A | ^43^ |
| 109 | 6p22.1 |  | **rs77285596** | 28442366 | intron | ZSCAN23 (p) |  |  | ZSCAN23>ZSCAN12>ZSCAN31 | T | G | 0.020 | 2.20E-19 | EAS | 1.362±0.034 | T | T | ^14^ |
| 110 | 6p22.1-6p21.32 | rs9267544^(46)^  rs3101018^(46)^  rs9267574^(46)^  rs3129950^(46)^ | rs1233491 | 29493953 | intergenic | MAS1L (p) |  |  | HLA-A>HLA-G>HLA-F | G | C | 0.018 | 7.70E-10 | EU | 2.2 (1.58-3.08) | C | C | ^91^ |
|  |  |  | rs3094067 | 30331468 | intron | TRIM39 (p); TRIM39-RPP21 (p) |  |  | HLA-C>FLOT1>HLA-A | T | G | 0.024 | 2.3E-11 | EU | 2.32 (1.68-3.20) | G | G | ^91^ |
|  |  |  | rs3131060 | 30795514 | intergenic | HCG20 (lncRNA) |  |  | FLOT1>HLA-C>CCHCR1 | G | A | 0.042 | 1.2E-13 | EU | 2.3 (1.71-3.11) | A | A | ^91^ |
|  |  |  | rs3132579 (rs114090659) ^[5]^ | 30973212 | intergenic | MUC21 (p) |  |  | HLA-C>MICB>FLOT1 | T | C | 0.043 | 5.81E-92 | EU | 1.99 | C | C | ^5^ |
|  |  |  | rs3094084 | 30979732 | intergenic | MUC21 (p) |  |  | FLOT1>HLA-C>MICB | A | T | 0.041 | 1.5E-11 | EU | 2.30 (1.76-3.01) | T | T | ^91^ |
|  |  |  | rs3130564 | 31133897 | intron | PSORS1C1 (p) |  |  | HLA-C>MICA>CCHCR1 | C | T | 0.0575 | 4E-10 | EU | 2.05 (1.56-2.70) | T | T | ^91^ |
|  |  |  | rs2844559 | 31372298 | intron | ENSG00000285647 (lncRNA) |  |  | MICB>HLA-C>HLA-B | C | T | 0.042 | 9.1E-15 | EU | 2.53 (1.89-3.39) | T | T | ^91^ |
|  |  |  | rs3099844 | 31481199 | non coding transcript exon | MICB-DT (lncRNA) |  |  | MICB>HLA-C>C4A | C | A | 0.0823 | 4.0E-10 | EU | 2.44 (1.81-3.29) | A | A | ^91^ (^92^) |
|  |  |  | rs2857595 | 31600692 | intergenic | NCR3 (p) |  |  | MICA>HLA-C>HLA-B | G | A | 0.321 | 1.96E-09 | EU | 2.37 (1.79-3.14) | A | A | ^92^ (^26^) |
|  |  |  | rs9267531 | 31668965 | intron | CSNK2B (p); ENSG00000263020 (p) |  |  | MICB>HLA-C>C4A | A | G | 0.0298 | 3.30E-14 | EU | 2.62 (1.93-3.54) | G | G | ^91^ |
|  |  |  | rs3131379 ^[6]^ | 31753256 | intron | MSH5 (p); MSH5-SAPCD1 (lncRNA) |  |  |  | G | A | 0.0338 | 1.7E-52 | EU | 2.36 (2.11-2.64) | A | A | ^67^ (^62,91^) |
|  |  |  |  |  |  |  |  |  |  |  |  |  | 4.1E-08 | AA | 1.65 (1.38-1.98) | A | A | ^93^ |
|  |  |  | rs558702 ^[6]^ | 31902549 | intron | C2 (p) |  |  |  | G | A | 0.0336 | 7.8E-21 | EU | 2.276 (2.47-2.097) | A | A | ^43^ |
|  |  |  | **rs1270942** | 31951083 | intron | CFB (p); ENSG00000244255 (p) |  |  | C4A>MICB>HLA-C | A | G | 0.0308 | 2.25E-165 | EU | 2.28 (2.15-2.42) | C | G | ^5^ (^9,67,94^) |
|  |  |  | rs1150757 | 32061428 | synonymous | TNXB (p) |  |  | C4A>CYP21A2>MICB | G | A | 0.0190 | 6.30E-107 | EU | 2.33 | A | A | ^5^ (^11^) |
|  |  |  | rs1150753 ^[7]^ | 32092090 | intron | TNXB (p) |  |  | C4A>MICB>CYP21A2 | A | G | 0.0188 | 1.00E-71 | EU | 2.20 (2.01-2.39) | G | G | ^95^ (^96^) |
|  |  |  | rs1269852 ^[6]^ | 32112414 | non coding transcript exon | ENSG00000284829 (lncRNA) |  |  | CYP21A2>HLA-DMA>MICB | G | C | 0.0190 | 5.63E-29 | Mix | 2.4 | C | C | ^97^ (^98^) |
|  |  |  | rs7775397 | 32293475 | missense | TSBP1 (p); TSBP1-AS1 (lncRNA) | K/Q |  | C4A>TSBP1>MICB | T | G | 0.0194 | 8.00E-47 | EU | 2.28 | G | G | ^67^ (^92^) |
|  |  |  | rs3117103 ^[8]^ | 32381780 | intron | TSBP1-AS1 (lncRNA) |  |  | MICB>HLA-DQA1>C4A | A | T | 0.0501 | 2.42E-16 | EU | 2.35 (1.89-2.92) | T | T | ^99^ |
|  |  |  | rs3135394 | 32440720 | intron | HLA-DRA (p) |  |  | HLA-DQB1>C4A>HLA-DMA | A | G | 0.0196 | 2.0E-60 | EU | 1.98 (1.84-2.14) | G | G | ^8^ |
|  |  |  | rs9272219 | 32634492 | intron | HLA-DQA1 (p) |  |  |  | G | T | 0.2734 | 2.00E-08 | EU | 1.91 (1.49-2.44) | T | T | ^91^ |
|  |  |  | rs2187668 ^[9]^ | 32638107 | intron | HLA-DQA1 (p) |  |  |  | C | T | 0.0777 | 9.4E-34 | EU | 1.94 (1.75-2.16) | T | T | ^69^ (^16,62,66,68,91,96^) |
|  |  |  | rs3129716 | 32689659 | intergenic | HLA-DQB1 (p) |  |  | HLA-DQB1>HLA-DQA1>HLA-DQA2 | T | C | 0.065 | 4.2E-09 | EU | - | - | C | ^96^ |
|  |  |  | rs1794282 | 32698749 | intergenic | HLA-DQB1 (p) |  |  | C4A>HLA-DMA>HLA-DQB1 | C | T | 0.020 | 2.60E-46 | EU | 2.26 | T | T | ^67^ |
|  |  |  | rs3957147 (rs114092478) ^[5]^ | 32714358 | intergenic | ENSG00000232080 (lncRNA) |  |  | HLA-DQB1>HLA-DQB2>HLA-DRB5 | C | T | 0.081 | 2.90E-93 | EU | 2 | T | T | ^5^ |
| 111 | 6p22.1-6p21.23 |  | rs111508444 | 29635735 | intergenic | LOC102725019 (lncRNA) |  |  | HLA-F>HLA-G>GABBR1 | A | G | 0.0761 | 2.41E-19 | EAS | 1.522±0.047 | A | A | ^14^ |
|  |  |  | rs566731348 | 31027112 | missense | MUC22 (p) | I/V |  |  | A | G | 0.0040 | 3.65E-12 | EAS | 1.889±0.092 | A | A | ^14^ |
|  |  |  | **rs2263318** | 31464229 | non coding transcript exon | HCP5 (lncRNA) |  |  | DDX39B>MICA>SKIV2L | G | A | 0.0811 | 1.05E-19 | EAS | 0.597±0.057 | A | G | ^14^ |
| 112 | 6p21.33 |  | **rs2524117 ^[7]^** | 31263092 | intergenic | HLA-C (p) |  |  | HLA-C>CCHCR1>HLA-B | T | C | 0.323 | 8.20E-21 | EU | 1.34 (1.26-1.43) | G | C | ^95^ |
|  |  |  | rs3906272 | 31295147 | intron | LINC02571 (lncRNA) |  |  | HLA-C>HLA-B>MICA | C | T | 0.150 | 2.95E-10 | EU | 2.84 (2.05-3.92) | A | T | ^98^ |
| 113 | 6p21.33 |  | **rs9265604 ^[7]^** | 31331673 | intergenic | HLA-B (p) |  |  | HLA-C>CCHCR1>HLA-B | T | C | 0.381 | 1.3E-08 | EU | 0.83 (0.78-0.89) | G | T | ^95^ |
| 114 | 6p21.33 |  | **rs4458721** | 31448075 | intron | ENSG00000288587 (lncRNA) |  |  | MICA>HLA-C>MICB | C | T | 0.3055 | 2.73E-10 | EAS | 1.181±0.026 | T | T | ^14^ |
| 115 | 6p21.33 |  | **rs2246618 ^[7]^** | 31511209 | intergenic | MICB (p) |  | HLA-C (p):e↓^95^ | MICB>HLA-C>FLOT1 | C | T | 0.283 | 1.76E-10 | EU | 1.49 (1.40-1.58) | A | T | ^95^ |
| 116 | 6p21.33 |  | rs6929796 | 31554892 | intron | NFKBIL1 (p) |  |  | LST1>DDX39B>HLA-C | G | A | 0.2692 | 6.68E-13 | EAS | - | - | - | ^26^ |
|  |  |  | **rs2256974** | 31587615 | intron | LST1 (p) |  |  |  | C | A | 0.2851 | 2.23E-25 | EAS | 0.842±0.017 | A | C | ^14^ |
| 117 | 6p21.33 |  | **rs9378200** ^[7]^ | 31605150 | intergenic | AIF1 (p) |  |  | MICA>MICB>LST1 | T | C | 0.135 | 8.2E-17 | EU | 0.59 (0.52-0.67) | C | T | ^95^ |
| 118 | 6p21.33 |  | **rs3115674** | 31831299 | intron | ENSG00000285565 (lncRNA) |  |  | LY6G6F>LY6G6F-LY6G6D>EHMT2 | T | G | 0.0429 | 1.43E-08 | EAS | 0.652±0.075 | T | G | ^14^ |
| 119 | 6p21.33 |  | **rs117217736** | 31840951 | intergenic | SNHG32 (lncRNA), ENSG00000285565 (lncRNA) |  |  | LY6G5B>CSNK2B>GPANK1 | T | C | 0.0010 | 1.06E-12 | EAS | 0.479±0.103 | T | C | ^14^ |
| 120 | 6p21.33 |  | **rs74290525** ^[5]^ | 31867385 | intron | SLC44A4 (p) |  |  | SKIV2L (SKIC2)>LY6G5C>LY6G5B | G | A | 0.0583 | 1.12E-12 | EU | 2.06 | G | G | ^5^ |
| 121 | 6p21.33-6p21.32 |  | rs419788 ^[10]^ | 31961022 | intron | SKIV2L (p) |  |  | HLA-DQA2>HLA-C>HLA-DRB5 | C | T | 0.1837 | 4.3E-08 | EU | 2.0 (1.6-2.6) | T | T | ^100^ |
|  |  |  | rs113164910* | 32427005 | intergenic | HLA-DRA (p) |  |  |  | AAC | A | - | 2.48E-37 | EAS | 1.65 (1.53-1.78) | D | A | ^27^ |
|  |  |  | rs9501626 | 32432567 | intergenic | HLA-DRA (p) |  |  | HLA-DRB5>HLA-DQB1>HLA-DQB2 | C | A | 0.195 | 1.00E-18 | EAS | 1.86 (1.62-2.13) | A | A | ^55^ (^26^) |
|  |  |  | rs3129895 (rs116727542) ^[11]^ | 32453450 | intergenic | HLA-DRA (p) |  |  | HLA-DRB5>HLA-DRB1>HLA-DQB1 | G | A | 0.159 | 6.15E-24 | EAS | 0.53 (0.47-0.60) | G | A | ^32^ |
|  |  |  | **rs9268807** | 32456138 | intergenic | HLA-DRA (p) |  |  |  | G | C | 0.159 | 4.90E-117 | EAS | 1.604±0.021 | C | C | ^14^ |
|  |  |  | rs7763262 | 32457105 | intergenic | HLA-DRA (p) |  |  | HLA-DQA2>HLA-DRB5>HLA-DRB1 | C | T | 0.251 | 3.96E-21 | EAS | - | - | T | ^26^ |
|  |  |  | rs1548306 (rs114883138) | 32459402 | intergenic | HLA-DRA (p) |  |  |  | T | A | 0.231 | 2.16E-78 | EU | 1.78 | - | A | ^11^ |
|  |  |  |  |  |  |  |  |  |  |  |  |  | 1.02E-20 | EAS | 1.6 | - | A | ^11^ |
|  |  |  | rs6903608 | 32460508 | intron | HLA-DRB9 (pseudogene) |  |  | HLA-DRB5>HLA-DRB1>HLA-DQA2 | T | C | 0.344 | 4.63E-14 | EAS | 1.282±0.033 | G | C | ^30^ |
|  |  |  | rs9269207 | 32482119 | intergenic | HLA-DRB5 (p) |  |  |  | T | C | 0.3293 | 6E-48 | EAS | 0.754±0.019 | T | C | ^14^ |
|  |  |  | rs9269233 | 32483985 | intergenic | HLA-DRB5 (p) |  |  |  | C | A | 0.2268 | 3.4E-11 | EAS | 1.17±0.024 | A | A | ^14^ |
|  |  |  | rs9270970 | 32605797 | intergenic | HLA-DRB1 (p) |  |  | HLA-DRB1>HLA-DQB1>HLA-DRB5 | T | C | 0.243 | 3.62E-26 | EAS | 1.8293 | G | G | ^70^ |
|  |  |  | rs9270984 | 32606214 | intergenic | HLA-DRB1 (p) |  |  | HLA-DRB1>HLA-DRB5>HLA-DQB1 | G | T | 0.173 | 4.37E-71 | EAS | 0.543±0.034 | G | T | ^1^ (^28^) |
|  |  |  |  |  |  |  |  |  |  |  |  |  | 6.51E-66 | Mix | 0.634±0.027 | G | T | ^1^ |
|  |  |  | rs9271055 | 32607592 | intergenic | HLA-DRB1 (p) |  |  |  | T | G | 0.174 | 4.32E-25 | EAS | - | - | G | ^26^ |
|  |  |  | rs9271100 ^[12]^ | 32608701 | intergenic | HLA-DRB1 (p) |  |  |  | C | T | 0.238 | 1.42E-12 | EAS | 1.90 (1.59-2.27) | A | T | ^21^ |
|  |  |  | rs9271366 ^[13]^ | 32619077 | intergenic | HLA-DQA1 (p) |  |  |  | A | G | 0.150 | 4.78E-38 | EAS | 1.696±0.041 | G | G | ^30^ (^29,98^) |
|  |  |  |  |  |  |  |  |  |  |  |  |  | 6.46E-12 | MA | 2.06 (1.71-2.50) | G | G | ^35^ |
|  |  |  | rs9271731 ^[7]^ | 32625835 | intergenic | HLA-DQA1 (p) |  |  |  | G | A | 0.319 | 1.3E-29 | EU | 1.59 (1.49-2.44) | A | A | ^95^ |
|  |  |  | rs9273076 ^[5]^ | 32644524 | non coding transcript exon | HLA-DQA1 (p) |  |  | HLA-DRB1>HLA-DRB5>HLA-DQB1 | G | T | 0.1625 | 7.54E-13 | EU | 1.3 | T | T | ^5^ |
| 122 | 6p21.33 |  | **rs554383943** | 32007481 | intron | CYP21A1P (pseudogene) |  |  |  | C | G | 0.0032 | 2.47E-13 | EAS | 3.634±0.176 | C | C | ^14^ |
| 123 | 6p21.33 |  | **rs406658** | 32028747 | synonymous | C4B (p) |  |  | SKIV2L>HLA-DRB5>CFB | C | A | 0.224 | 4.95E-08 | EAS | 0.71 (0.63-0.80) | A | C | ^32^ |
| 124 | 6p21.33 |  | **rs1150755** | 32070773 | intron | TNXB (p) |  |  | MICB>CYP21A2>HLA-C | C | T | 0.0523 | 6.11E-117 | Mix | 1.30 (1.19-1.41) | T | T | ^4^ |
|  |  |  |  |  |  |  |  |  |  |  |  |  | 5.00E-114 | EU | 1.33 (1.20-1.48) | T | T | ^4^ |
|  |  |  |  |  |  |  |  |  |  |  |  |  | 1.28E-08 | MA | 1.30 (1.19-1.41) | T | T | ^4^ |
|  |  |  | rs1150754 ^[9]^ | 32082981 | intron | TNXB (p) |  |  | MICB>C4A>CYP21A2 | C | T | 0.0497 | 6.40E-29 | EU | 2.21 (1.93-2.53) | A | T | ^66^ |
| 125 | 6p21.32 |  | **rs200283861** | 32026946 | intron | C4B (p) |  |  | ATF6B>TNXB>BAG6 | G | A | 0.2161 | 2.51E-23 | EAS | 1.203±0.019 | A | A | ^14^ |
|  |  |  | rs4713505 | 32137224 | intergenic | FKBPL (p) |  |  | CYP21A2>NOTCH4>TNXB | G | T | 0.174 | 1.01E-08 | EAS | - | - | - | ^26^ |
| 126 | 6p21.32 |  | **rs9281656 (rs565981183)** | 32143247-32143265 | intergenic | PRRT1 (p) |  |  | EGFL8>ATF6B>FKBPL | (A)_19_ | (A)_18_ | 0.176 | 1.23E-11 | EAS | 1.192±0.026 | CA (vs C) | (A)_19_ | ^14^ |
| 127 | 6p21.32 |  | **rs8192591 ^[7]^** | 32218019 | missense | NOTCH4 (p) | G/S |  | MICA>HLA-DRB5>MICB | C | T | 0.0312 | 4.2E-21 | EU | 1.34 (1.26-1.43) | G | C | ^95^ |
| 128 | 6p21.32 |  | **rs368529276** | 32353696 | intron | TSBP1 (p) |  |  |  | A | G | 0.0030 | 3.52E-19 | EAS | 2.522±0.103 | A | A | ^14^ |
| 129 | 6p21.32 |  | **rs3129941** | 32369909 | missense | TSBP1 (p) | C/R |  | HLA-DRB5>HLA-DRB1>HLA-DRA | G | A | 0.1967 | 9.65E-09 | EAS | - | - | - | ^26^ |
| 130 | 6p21.32 |  | **rs72548051** (rs796780915) | 32435620-32435621 | intergenic | HLA-DRA (p) |  |  |  | A or AA** | AC or A** | <0.001 | 1.3E-29 | EAS | 1.67 (1.52-1.82) | AC | AC | ^23^ |
| 131 | 6p21.32 |  | rs7192 | 32443869 | missense | HLA-DRA (p) | L/V |  | HLA-DRA>HLA-DQA2>HLA-DRB1 | G | T | 0.3405 | 6.2E-40 | EU | 1.61 | - | T | ^67^ |
|  |  |  | rs4410767 | 32480352 | intergenic | HLA-DRA (p) |  |  |  | T | C | 0.354 | 1E-09 | EU | 0.39 (0.28-0.52) | C | T | ^91^ |
|  |  |  | rs1049225 (rs9273448) | 32659970 | 3’ UTR | HLA-DQB1 (p); HLA-DQB1-AS1 (lncRNA) |  |  | HLA-DQB1>HLA-DRB1>HLA-DRB5 | G | A | 0.1695 | 3.68E-32 | Mix | - | A | A | ^4^ |
|  |  |  |  |  |  |  |  |  |  |  |  |  | 9.08E-19 | EU | - | A | A | ^4^ |
|  |  |  |  |  |  |  |  |  |  |  |  |  | 6.00E-10 | AA | - | A | A | ^4^ |
|  |  |  | rs9469220 ^[7]^ | 32690533 | intergenic | HLA-DQB1 (p) |  |  | HLA-DRB5>HLA-DOB>TAP2 | A | G | 0.398 | 1.1E-47 | EU | 0.65 (0.61-0.68) | T | G | ^95^ |
|  |  |  | **rs9275572** | 32711222 | intergenic | LOC102725019 (lncRNA) |  |  | HLA-DQA2>HLA-DQA1>HLA-DRA | G | A | 0.335 | 7.00E-48 | EU | 1.69 | A | A | ^67^ |
|  |  |  |  |  |  |  |  |  |  |  |  |  | 1.11E-16 | MA | 1.62 (1.46-1.80) | A | A | ^35^ (^43^) |
|  |  |  | rs3135461 | 32712345 | intergenic | LOC102725019 (lncRNA) |  |  | HLA-DQA2>HLA-DRB1>HLA-DRB5 | A | G | 0.1855 | 1.88E-39 | EAS | 0.726±0.024 | A | G | ^14^ |
| 132 | 6p21.32 |  | **rs28529717** | 32647186 | intergenic | HLA-DQA1 (p) |  |  | HLA-DQA1>HLA-DQB1>HLA-DRB1 | G | A | 0.316 | 4.3E-21 | EAS | 2.19 (1.86-2.58) | A | A | ^24^ |
| 133 | 6p21.32 |  | **rs9268989** | 32467772 | intergenic | HLA-DRA (p) |  |  | HLA-DRB5>HLA-DRB1>HLA-DQB1 | G | A | 0.4151 | 6.49E-14 | EAS | 0.856±0.021 | A | G | ^14^ |
|  |  |  | rs9269029 | 32469767 | intergenic | HLA-DRA (p) |  |  | HLA-DRB5>HLA-DRB1>HLA-DQA1 | C | A | 0.2526 | 2.33E-08 | EAS | 0.866±0.026 | A | C | ^14^ |
| 134 | 6p21.32 |  | **rs34452045** | 32474342 | intergenic | HLA-DRA (p) |  |  |  | C | - | 0.1817 | 3.39E-31 | EAS | 0.694±0.031 | G (vs GC) | C | ^14^ |
| 135 | 6p21.32 |  | **6:32449301:T:<INS:ME:ALU>** | 32481524 | intergenic | HLA-DRA (p) |  |  |  | T | <INS:ME:ALU> | 0.128 (^14^) | 7.84E-37 | EAS | 0.616±0.038 | <INS:ME:ALU> | T | ^14^ |
| 136 | 6p21.32 |  | **rs2647078** | 32598319 | intergenic | HLA-DRB1 (p) |  |  |  | C | G | 0.1645 | 8.51E-21 | EAS | 0.803±0.023 | C | G | ^14^ |
| 137 | 6p21.32 |  | **rs660895** | 32609603 | intergenic | HLA-DRB1 (p) |  |  | HLA-DQA2>HLA-DRB1>HLA-DRB5 | A | G | 0.198 | 4.77E-31 | EAS | 0.619±0.041 | G | A | ^30^ |
|  |  |  | rs9275312 | 32697951 | intergenic | HLA-DRB1 (p) |  |  | HLA-DQA2>HLA-DQA1>HLA-DRB1 | A | G | 0.186 | 2.79E-14 | EAS | - | - | A | ^26^ |
|  |  |  | rs9275383 (rs114653103) | 32701069 | intergenic | LOC102725019 (lncRNA) |  |  |  | G | T | 0.123 | 7.31E-15 | EAS | 0.57 (0.49-0.66) | T | G | ^32^ |
|  |  |  | rs9275428 | 32703201 | intergenic | LOC102725019 (lncRNA) |  |  | HLA-DQA2>HLA-DRB5>HLA-DRB1 | A | G | 0.306 | 4.99E-11 | EAS | 0.50 (0.41-0.62) | G | A | ^52^ |
| 138 | 6p21.32 |  | **rs1966002** | 32613907 | intergenic | HLA-DQA1 (p) |  |  | HLA-DRB1>HLA-DRB5>HLA-DRA | G | T | 0.167 (ALFA) | 8.43E-31 | EAS | 1.337±0.025 | T | T | ^14^ |
| 139 | 6p21.32 |  | **rs9273349** | 32658092 | intergenic | HLA-DQB1 (p) |  |  | HLA-DQA1>HLA-DRA>C2 | T | C | 0.366 ^(26)^ | 3.24E-38 | EAS | 1.577±0.035 | A | T | ^30^ (^26^) |
| 140 | 6p21.32 |  | **rs9273371** | 32658788 | intergenic | HLA-DQB1 (p) |  |  | HLA-DQA1>HLA-DQA2>HLA-DQB1 | C | T | 0.214 | 1.18E-09 | EAS | 1.61 (1.38-1.87) | T | T | ^32^ |
| 141 | 6p21.32 |  | **rs17412833** | 32664821 | missense | HLA-DQB1 (p) | F/Y |  | HLA-DQB1>HLA-DQA2>HLA-DQA1 | A | T | 0.286 | 7.81E-17 | EAS | 1.785 | T | T | ^101^ |
| 142 | 6p21.32 |  | **rs189311301** | 32688139 | intergenic | HLA-DQB1 (p) |  |  |  | C | G | 0.0148 | 2.92E-10 | EAS | 2.233±0.127 | C | C | ^14^ |
| 143 | 6p21.32 |  | **rs17206287** | 32706948 | intergenic | LOC102725019 (lncRNA) |  |  | HLA-DRB5>HLA-DQA1>HLA-DQB2 | A | G | 0.1743 | 2.04E-12 | EAS | 1.2±0.026 | A | A | ^14^ |
| 144 | 6p21.32 |  | rs3997854 ^[12]^ | 32715138 | intergenic | LOC102725019 (lncRNA) |  |  | TAP2>HLA-DOB>HLA-DQA2 | T | G | 0.163 | 2.85E-08 | EAS | 0.44(0.32-0.58) | C | T | ^21^ |
|  |  |  | rs7769979 | 32755795 | intergenic | HLA-DQB2 (p) |  |  | HLA-DOB>TAP2>HLA-DRB5 | A | G | 0.425 | 5.64E-14 | EU | 0.58 (0.50-0.67) | A | G | ^99^ |
|  |  |  | rs2301271 | 32757416 | intron | HLA-DQB2 (p) |  |  | HLA-DOB>TAP2>HLA-DRA | G | A | 0.2813 | 2E-12 | EU | 1.47 (1.32-1.63) | T | A | ^66^ |
|  |  |  | **rs7753017** | 32759548 | intron | HLA-DQB2 (p) |  |  | HLA-DOB>TAP2>HLA-DRB5 | G | A | 0.3429 | 5.33E-43 | EAS | 0.745±0.021 | A | G | ^14^ |
|  |  |  | rs2051549 | 32762309 | intron | HLA-DQB2 (p) |  |  | HLA-DOB>TAP2>HLA-DRA | A | G | 0.2574 | 3.36E-22 | EU | - | - | G | ^59^ |
| 145 | 6p21.32 |  | **rs115910061** | 33076238 | intron | HLA-DPB1 (p); HLA-DPA1 (p) |  |  | HLA-DPA1>HLA-DOA>HLA-DPB1 | G | T | 0.0190 | 4.82E-12 | EAS | 0.721±0.047 | T | G | ^14^ |
| 146 | 6p21.32 |  | **rs1431403** | 33079254 | intron | HLA-DPB1 (p); HLA-DPA1 (p) |  |  | HLA-DPB1>HLA-DPA1>HLA-DOA | T | C | 0.4577 | 1.14E-22 | EAS | 0.848±0.017 | T | C | ^14^ |
| 147 | 6p21.31 | rs3748079^(102)^ | **rs3748079** | 33620370 | 5’ UTR | ITPR3 (p) |  | ITPR3 (p):e↓^102^ | ITPR3>BAK1>IP6K3 | C | T | 0.1685 | 1.78E-08 | EAS | 1.88 (1.51–2.35) | C | C | ^102^ |
| 148 | 6p21.31 |  | rs205284 | 34584135 | intergenic | ILRUN (p) |  | BLTP3A (UHRF1BP1) (p): c^4^,e↓^1,5,103^,d^1^  ZNF76 (p): c,e^4^ | UHRF1BP1>SNRPC>TAF11 | C | T | 0.103 | 1.37E-12 | EAS | - | - | T | ^26^ |
|  |  |  | 6:34651199:I (b37)***  (rs201879119?) | 34683422 | intron | ILRUN (p) |  |  |  | (T?) | (TT?) | EU:0.110; AS:0.113 (^11^) | 9.68E-09 | EU | 1.29 | I | (TT?) | ^11^ |
|  |  |  |  |  |  |  |  |  |  |  |  |  | 4.69E-12 | EAS | 1.60 | I | (TT?) | ^11^ |
|  |  |  | rs6908462 | 34705372 | intergenic | ILRUN-AS1 (lncRNA) |  |  | SNRPC>TAF11>ILRUN | C | T | 0.187 | 6.11E-12 | EAS | - | - | T | ^26^ |
|  |  |  | rs9462027 | 34829464 | intron | BLTP3A (p) |  |  | SNRPC>ILRUN>UHRF1BP1 | G | A | 0.3313 | 1.01E-10 | EAS | 1.46 | - | A | ^11^ |
|  |  |  |  |  |  |  |  |  |  |  |  |  | 7.55E-09 | EU | 1.14 (1.09-1.19) | A | A | ^5^ (^4^) |
|  |  |  | rs34840245 | 34844924 | intron | BLTP3A (p) |  |  | SNRPC>TAF11>UHRF1BP1 | T | G | 0.1532 | 2E-11 | Mix | 1.20 (1.14-1.27) | G | G | ^4^ |
|  |  |  | **rs3734266** ^[14]^ | 34855410 | intron | BLTP3A (p) |  |  | SNRPC>TAF11>UHRF1BP1 | T | C | 0.1573 | 9.40E-24 | Mix | 1.289±0.025 | C | C | ^1^ |
|  |  |  |  |  |  |  |  |  |  |  |  |  | 1.96E-18 | EAS | 1.339±0.033 | C | C | ^1^ (^26,28^) |
|  |  |  | rs6457796 | 34860776 | intron | BLTP3A (p) |  |  | ILRUN>SNRPC>UHRF1BP1 | T | C | 0.356 | 2.68E-22 | EAS | 0.808±0.022 | T | C | ^14^ |
|  |  |  | rs13205210 ^[14]^ | 34864079 | missense | BLTP3A (p) | M/T |  | UHRF1BP1>SNRPC>TAF11 | T | C | 0.1438 | 2.26E‐17 | EAS | 1.41 | C | C | ^103^ (^26,29,30,104^) |
|  |  |  | rs2234044 | 35122320 | synonymous | TCP11 (p) |  |  | TCP11>DEF6>ZNF76 | T | C | 0.159 | 8.27E-09 | EAS | - | - | C | ^26^ |
|  |  |  | rs3756856 | 35142928 | intron | TCP11 (p) |  |  |  | A | G | 0.136 | 3.23E-09 | EAS | - | - | G | ^26^ |
| 149 | 6p21.31 |  | rs9462015 | 34771949 | intron | SNRPC (p) |  | BLTP3A (UHRF1BP1) (p):c^4^ | SNRPC>ILRUN>UHRF1BP1 | T | G | 0.4525 | 5.30E-09 | EU | 1.28 (1.18-1.38) | - | G | ^69^ |
|  |  |  | **rs11755393** ^[14]^ | 34856859 | missense | TCP11 (p) | Q/R |  |  | T | G | 0.4677 | 2.20E-08 | EU | 1.17 (1.10-1.24) | G | G | ^8^ |
|  |  |  |  |  |  |  |  |  |  |  |  |  | 1.56E-12 | Mix | 0.87±0.020 | A | G | ^84^ |
| 150 | 6p21.31 |  | **rs820077** | 35066077 | intron | ANKS1A (p) |  |  | TCP11>ANKS1A>DEF6 | A | G | 0.2488 | 1.31E-08 | EU | 1.19 | G | G | ^5^ |
| 151 | 6p21.32 |  | **rs2762340** | 35084858 | intron | ANKS1A (p) |  | DEF6 (p):e^76^ | ANKS1A>TCP11>DEF6 | A | G | 0.3163 | 4.93E-15 | Mix | 0.87 (0.84-0.90) | G | A | ^76^ |
|  |  |  | rs10484578 | 35278542 | intron | ZNF76 (p) |  |  | TCP11>DEF6>ZNF76 | A | G | 0.4069 | 3.73E-14 | EAS | 1.131±0.016 | A | A | ^14^ |
| 152 | 6p21.31 | rs6940375^(46)^  rs4711411^(46)^  rs3800392^(46)^  rs78199774^(46)^  rs6938946^(46)^  rs77904818^(46)^ | **rs10807150** | 35304497 | intron | DEF6 (p) |  | ZNF76 (p):e^27^  DEF6 (p):e^27^ | TCP11>ZNF76>DEF6 | T | C | 0.3798 | 6.06E-16 | EAS | 1.25 (1.19-1.32) | C | C | ^27^ |
|  |  |  | rs4713853 | 35359578 | Intron | PPARD (p) |  |  | DEF6>ZNF76>RPL10A | T | C | 0.366 | 3.04E-12 | Mix | 1.18±0.023 | A^#^ | T | ^84^ |
|  |  |  |  |  |  |  |  |  |  |  |  |  | 2.42E-08 | EAS | 1.17±0.028 | A^#^ | T | ^84^ |
| 153 | 6p21.2 |  | **rs34868004** | between 36747254 and 36747255 | intron | CPNE5 (p) |  |  | CPNE5>RAB44>CDKN1A | - | A | 0.233 | 4.46E-09 | EAS | 1.104±0.017 | CA | A | ^14^ |
| 154 | 6q15 |  | **rs3857496** | 90009445 | intron | BACH2 (p) |  |  | BACH2>GJA10>CASP8AP2 | T | C | 0.3293 | 7.66E-09 | EAS | 1.116±0.019 | T | T | ^14^ |
| 155 | 6q15 |  | **rs597325** | 90292775 | intron | BACH2 (p) |  | BACH2 (p):i^11^,e^1^,d^1^ | BACH2>MDN1>PNRC1 | G | A | 0.3049 | 4.03E-12 | Mix | 0.89 (0.86-0.92) | A | G | ^11^ |
|  |  |  |  |  |  |  |  |  |  |  |  |  | 4.60E-11 | EAS | 0.906±0.015 | A | G | ^14^ (^11^) |
| 156 | 6q21 |  | **rs6923608** | 106089915 | intron | ATG5 (p); PRDM1 (p) |  |  | PRDM1>ATG5>CRYBG1 | G | A | 0.1949 | 6.13E-09 | EU | 1.20 (1.13-1.28) | T | A | ^4^ |
| 157 | 6q21 |  | rs533733 | 106116361 | intron | ATG5 (p) |  | ATG5 (p):e↑^105^,i^5^  ATG3 (p):e↑^105^  PRDM1 (p):i^5^,e^1^,d^1^ | PRDM1>ATG5>CRYBG1 | G | A | 0.204 | 1.7E-10 | EAS | 1.47 (1.3-1.65) | A | A | ^24^ |
|  |  |  | **rs548234** | 106120159 | intron | ATG5 (p) |  |  |  | T | C | 0.2029 | 2.39E-28 | Mix | 0.814±0.019 | T | C | ^1^ |
|  |  |  |  |  |  |  |  |  |  |  |  |  | 5.54E-39 | EAS | 0.819±0.015 | T | C | ^14^ (^1,21,30,105^) |
|  |  |  |  |  |  |  |  |  |  |  |  |  | 8.29E-10 | EU | 0.835±0.029 | T | C | ^1^ (^4^) |
|  |  |  | rs7768653 | 106126919 | intron | ATG5 (p) |  |  |  | T | C | 0.2706 | 3.11E-12 | EU | 1.23 | - | C | ^11^ |
|  |  |  | rs10581116 | 106131232 -106131254 | intron | ATG5 (p) |  |  |  | - | TTT | 0.238 | 1.33E-09 | EAS | 1.27 (1.18-1.37) | I | TTT | ^27^ |
|  |  |  | rs6568431 | 106140931 | intron | ATG5 (p) |  |  | PRDM1>ATG5>CRYBG1 | C | A | 0.3788 | 5.04E-14 | EU | 1.21 (1.15 -1.27) | A | A | ^5^ (^8,9,11,16,67^) |
| 158 | 6q21 |  | **rs9373839** | 106207742 | intron | ATG5 (p) |  | ATG5 (p):e^34^ | ATG5>PRDM1>CRYBG1 | T | C | 0.1102 | 4.18E-15 | Mix | 1.19 (1.12-1.26) | G | C | ^4^ |
|  |  |  |  |  |  |  |  |  |  |  |  |  | 3.84E-14 | EU | 1.22 (1.16-1.29) | G | C | ^4^ |
|  |  |  | rs2299864 | 106220119 | intron | ATG5 (p) |  |  | ATG5>PRDM1>PREP | C | T | 0.11 | 5.77E-15 | EU | 1.24 (1.17-1.30) | T | T | ^4^ |
| 159 | 6q22.1 |  | **rs9488914** | 116369686 | intron | DSE (p) |  |  | DSE>TSPYL1>TSPYL4 | T | C | 0.381 | 1.14E-08 | EAS | 0.862±0.026 | T | C | ^14^ |
|  |  |  | rs9387400 | 116372957 | intron | DSE (p) |  |  |  | A | C | 0.429 | 3.14E-08 | EAS | 1.346±0.054 | C | C | ^1^ |
| 160 | 6q23.3 | *TEx2* | **rs2327832** | 137651931 | intergenic | LINC03004 (lncRNA) |  | OLIG3 (p):e^1^ | OLIG3>IFNGR1>TNFAIP3 | A | G | 0.095 | 1.76E-13 | EU | 1.22 (1.15-1.28) | C | G | ^4^ |
| 161 | 6q23.3 | chr6:137908902-137908903 TT>A^(106)^  rs148314165^(46)^ rs200820567^(46)^  *TE* | rs75163761 | 137836352 | intergenic | ILRUN-AS1 (lncRNA) |  | TNFAIP3 (p) :c^4,5^,i^5^,e^1^,d^1^  WAKMAR2 (lnc-PERP-2, ENSG00000235842) (lncRNA):d^1^ |  | C | T | 0.0030 | 5.23E-24 | EAS | 1.631±0.048 | T | T | ^14^ |
|  |  |  | rs5029924 | 137866361 | intron | WAKMAR2 (lncRNA) |  |  | TNFAIP3>HEBP2>TXLNB | C | T | 0.131 | 9.54E-17 | EU | 2.09 | T | T | ^10^ |
|  |  |  | rs3757173 | 137869017 | intron | TNFAIP3 (p) |  |  | HEBP2>TNFAIP3>TXLNB | A | G | 0.2055 | 1.75E-15 | EAS | 1.952 (1.656-2.302) | G | G | ^107^ |
|  |  |  | rs7750604 | 137869396 | intron | TNFAIP3 (p) |  |  |  | C | T | 0.207 | 1.3E-12 | EAS | 1.93 (1.61-2.32) | T | T | ^24^ |
|  |  |  | rs5029937 | 137874014 | intron | TNFAIP3 (p) |  |  | TNFAIP3>HEBP2>IFNGR1 | G | T | 0.1396 | 5.91E-24 | Mix | 1.899 (1.677-2.151) | T | T | ^107^ |
|  |  |  |  |  |  |  |  |  |  |  |  |  | 1.14E-15 | EAS | 2.100 (1.751-2.518) | T | T | ^107^ (^32^) |
|  |  |  |  |  |  |  |  |  |  |  |  |  | 5.3E-13 | EU | 1.71 (1.51-1.95) | T | T | ^8^ (^107^) |
|  |  |  | rs5029939 | 137874586 | intron | TNFAIP3 (p) |  |  |  | C | G | 0.1396 | 2.39E-29 | Mix | 1.81 (1.63-2.01) | C | G | ^4^ |
|  |  |  |  |  |  |  |  |  |  |  |  |  | 5E-29 | EU | 1.81 (1.63-2.01) | C | G | ^4^ (^36,53,108^) |
|  |  |  | rs2230926 | 137874929 | missense | TNFAIP3 (p) | F/C |  |  | T | G | 0.1396 | 2.79E-29 | EU | 1.81 (1.63-2.01) | C | G | ^4^ (^1,5,11,62,107^) |
|  |  |  |  |  |  |  |  |  |  |  |  |  | 7.34E-59 | Mix | 1.881±0.039 | G | G | ^1^ (^80,107^) |
|  |  |  |  |  |  |  |  |  |  |  |  |  | 5.76E-46 | EAS | 2.001±0.049 | G | G | ^1^ (^11,21,26,28,29,55,107,109,110^) |
|  |  |  | rs9494894 | 137907383 | intergenic | ENSG00000287393 (lncRNA) |  |  |  | T | C | 0.0675 | 3.48E-84 | EAS | 0.586±0.027 | T | C | ^14^ (^27^) |
|  |  |  | **rs148314165** | 137908902-137908906 | intergenic | ENSG00000287393 (lncRNA) |  |  | TNFAIP3>PERP>ARFGEF3 | (T)_5_ | (T)_4_ | 0.023 | 3.48E-84 | EAS | 1.711±0.028 | G (vs GT) | (T)_4_ | ^14^ |
|  |  |  | 6:137908902-137908903 | 137908902-137908903(137908906) | intergenic | ENSG00000287393 (lncRNA) |  |  |  | TT or (T)_5_ | A or A(T)_3_ | 0.039 (^106^) | 8.33E-10 | EAS | 2.54(1.89-3.42) | A (vs TT) | A(T)_3_ | ^106^ |
|  |  |  |  |  |  |  |  |  |  |  |  |  | 1.58E-08 | EU | 1.70 (1.41-2.04) | A (vs TT) | A(T)_3_ | ^106^ |
|  |  |  | rs4896303 | 137912948 | intergenic | ENSG00000287393 (lncRNA) |  |  | TNFAIP3>IFNGR1>PERP | G | C | 0.472 | 6.84E-11 | EAS | 2.35 (1.82-3.03) | C | C | ^106^ |
|  |  |  | rs9494895 | 137913641 | intergenic | ENSG00000287393 (lncRNA) |  |  |  | C | T | 0.065 | 6.0E-14 | EAS | 1.74 (1.51-2.01) | T | T | ^23^ |
|  |  |  | rs77000060 | 137916852 | intergenic | ENSG00000287393 (lncRNA) |  |  |  | C | T | 0.038 | 1.84E-29 | EU | 1.89 (1.69-2.11) | T | T | ^4^ (^11^) |
|  |  |  |  |  |  |  |  |  |  |  |  |  | 4.74E-13 | EAS | 2.08 | - | T | ^11^ |
|  |  |  | rs6932056 | 137921300 | intergenic | ENSG00000287393 (lncRNA) |  |  | TNFAIP3>PERP>IFNGR1 | T | C | 0.041 | 1.97E-31 | EU | 1.83 (1.65-2.02) | C | C | ^5^ (^9,106^) |
|  |  |  | rs58721818 | 137922602 | intergenic | ENSG00000287393 (lncRNA) |  |  |  | C | T | 0.035 | 3.19E-19 | EU | 1.82 | T | T | ^5^ |
| 162 | 6q25.2 |  | **rs9322454** | 154249517 | intron | IPCEF1 (p) |  |  | IPCEF1>SCAF8>CNKSR3 | G | A | 0.263 | 2.42E-08 | EAS | 1.090±0.015 | A | A | ^14^ |
|  |  |  |  |  |  |  |  |  |  |  |  |  |  |  |  |  |  |  |
| 163 | 7p15.1 |  | rs10245867 | 28102567 | intron | JAZF1 (p) |  | JAZF1 (p):e^1,5^,d^1^ | JAZF1>CREB5>TAX1BP1 | T | G | 0.4533 | 4.31E-08 | EU | 1.14 (1.09-1.19) | T | T | ^4^ |
|  |  |  | rs12531540 | 28123055 | intron | JAZF1 (p) |  |  |  | C | T | 0.2101 | 3.35E-08 | EU | 1.15 | C | C | ^5^ |
|  |  |  | **rs702814** | 28133113 | intron | JAZF1 (p) |  |  |  | C | T | 0.2151 | 1.97E-11 | Mix | 1.15 (1.10-1.20) | C | C | ^4^ |
|  |  |  |  |  |  |  |  |  |  |  |  |  | 4.67E-08 | EU | 1.13 (1.08-1.18) | C | C | ^4^ |
|  |  |  | rs849142 | 28146272 | intron | JAZF1 (p) |  |  |  | T | C | 0.2354 | 8.61E-11 | EU | 1.14 (1.10-1.19) | A | T | ^5^ (^4,8,42^) |
| 164 | 7p12.2 |  | **rs2366293** | 50188232 | intergenic | ENSG00000231681 (lncRNA) |  |  | IKZF1>FIGNL1>ZPBP | C | G | 0.153 | 2.33E-09 | EU | 1.23 | G | G | ^42^ |
| 165 | 7p12.2 |  | **rs4598207** | 50218883 | intron | ENSG00000231681 (lncRNA |  | IKZF1 (p):i^5^,e*↑*^1,111^,d^1^  CLEC4C (p):e↑^111^  HERC5 (p):e↑^111^  IFI6 (p):e↑^111^  IFIT1 (p):e↑^111^  MX1 (p):e↑^111^  TNFRSF21 (p):e↑^111^  CLEC10A (p):e*↓*^111^  C1QB (p):e*↓*^111^ | IKZF1>FIGNL1>ZPBP | A | T | 0.342 | 4.12E-60 | EAS | 1.333±0.018 | A | A | ^14^ |
|  |  |  | rs11185602 | 50259481 | intron | ENSG00000231681 (lncRNA |  |  | IKZF1>ZPBP>SPATA48 | A | G | 0.3381 | 1.53E-16 | EAS | 0.66 (0.60-0.73) | G | A | ^32^ (^23^) |
|  |  |  | rs4917014 | 50266267 | intergenic | ENSG00000231681 (lncRNA) |  |  |  | T | G | 0.278 | 5.18E-29 | EAS | 1.33±0.03 | C | T | ^1^ (^21,30^) |
|  |  |  |  |  |  |  |  |  |  |  |  |  | 2.17E-31 | Mix | 0.798±0.019 | G | T | ^1^ (^4^) |
|  |  |  |  |  |  |  |  |  |  |  |  |  | 6.39E-14 | EU | 1.18 (1.13-1.24) | T | T | ^5^ (^4,112^) |
|  |  |  | rs4385425 | 50267738 | intergenic | ENSG00000231681 (lncRNA) |  |  | IKZF1>FIGNL1>ZPBP | A | G | 0.480 | 1.51E-09 | EU | 0.872 (0.832-0.914) | G | A | ^112^ |
|  |  |  |  |  |  |  |  |  |  |  |  |  | 1.1E-11 | EAS | 1.49 (1.33-1.67) | A | A | ^24^ |
|  |  |  | rs876037 | 50269096 | intergenic | ENSG00000235620 (lncRNA) |  |  | IKZF1>ZPBP>SPATA48 | T | A | 0.265 | 9.36E-23 | EAS | 0.68 (0.63-0.74) | A | T | ^27^ |
|  |  |  | rs62447171 | 50270294 | intergenic | ENSG00000235620 (lncRNA) |  |  |  | G | A | 0.3107 | 8.97E-60 | EAS | 0.767±0.016 | A | G | ^14^ |
| 166 | 7p12.2 |  | **rs10239000** | 50292600 | intergenic | IKZF1 (p) |  |  | IKZF1>ZPBP>SPATA48 | G | A | 0.2021 | 1.51E-25 | EAS | 1.189±0.017 | A | A | ^14^ |
|  |  |  | rs62447178 | 50299441 | intergenic | IKZF1 (p) |  |  | IKZF1>FIGNL1>ZPBP | T | C | 0.080 | 7.23E-11 | EAS | 1.31 (1.20-1.41) | C | C | ^27^ |
| 167 | 7q11.22 |  | **rs13238909** | 67611386 | intergenic | ENSG00000233423 (lncRNA) |  |  | SPDYE21>KCTD7 | G | A | 0.088 | 4.40E-08 | Mix | 0.85 | A | G | ^113^ |
| 168 | 7q11.23 |  | **rs150518861** | 74152347 | intergenic | EIF4H (p) |  |  | METTL27>DNAJC30>EIF4H | G | A | 0.005 | 4.10E-08 | EU | 1.66 (1.49-1.84) | A | A | ^9^ |
| 169 | 7q11.23 |  | **rs10716716** | 74193613-74193626 | non coding transcript exon | EIF4H (p) |  |  |  | (T)14 | (T)13 | 0.1879 | 8.49E-10 | EAS | 1.151±0.023 | G (vs GT) | (T)14 | ^14^ |
| 170 | 7q11.23 |  | **rs372942110** | 74214690-74214721 | intron | LAT2 (p) |  |  | LAT2 | (AATATATATA)3AA | (AATATATATA)2AA | 0.0062 | 5.62E-09 | EAS | 2.294±0.142 | T (vs TAATATATATA) | (AATATATATA)3AA | ^14^ |
| 171 | 7q11.23 |  | **rs530634980** | 74495911 | intron | GTF2IRD1 (p) |  |  | GTF2IRD1>CCL24>GTF2I | C | T | 0.0010 | 5.1E-18 | EAS | 2.016±0.081 | T | T | ^14^ |
| 172 | 7q11.23 |  | **rs73135369** | 74526648 | intron | GTF2IRD1 (p) |  | NCF1 (p):e,d^1^ | GTF2IRD1>GTF2I>CLIP2 | T | C | 0.0312 | 8.77E-14 | Mix | 1.32 (1.23-1.42) | C | C | ^11 (1)^ |
|  |  |  |  |  |  |  |  |  |  |  |  |  | 7.33E-13 | EAS | 1.38 (1.26-1.51) | C | C | ^11 (1)^ |
| 173 | 7q11.23 |  | **rs13244581** | 74580705 | intron | GTF2IRD1 (p) |  |  | GTF2IRD1>GTF2I>NCF1 | G | C | 0.1705 | 1.59E-20 | EAS | 0.667±0.044 | C | G | ^14^ |
| 174 | 7q11.23 | rs4717901^(46)^ | **rs80346167** ^[15]^ | 74586373 | intron | GTF2IRD1 (p) |  |  | GTF2IRD1>GTF2I>NCF1 | G | A | 0.114 | 3.26E-29 | EAS | - | - | A | ^27^ |
|  |  |  | rs4717901 | 74602653 | intergenic | GTF2IRD1 (p) |  |  |  | A | C | 0.071 | 1.5E-24 | EAS | 2.16 (1.86-2.50) | C | C | ^114^ |
| 175 | 7q11.23 |  | **rs116991837** | 74589381 | intron | GTF2IRD1 (p) |  |  | GTF2IRD1>GTF2I>NCF1 | G | A | 0.0016 | 3.82E-15 | EAS | 2.654±0.124 | A | A | ^14^ |
| 176 | 7q11.23 |  | **rs150724213** | 74606017 | intergenic | ENSG00000273069 (lncRNA) |  |  | GTF2I>NCF1>GTF2IRD1 | G | A | 0.0048 | 2.51E-15 | EAS | 3.882±0.171 | A | A | ^14^ |
| 177 | 7q11.23 | rs201802880^(115–118)^ | rs7800325 | 74618332 | intergenic | ENSG00000273069 (lncRNA) |  | NCF1 (p) rs201802880: c^115,117^, e^34^, f(ROS production↓)^117,118^  GTF2I (p):3D^27^  VGF (p):3D^27^ | SPDYE12>GTF2IRD1>GTF2I | T | C | 0.116 | 1.04E-280 | EAS | 0.449±0.022 | T | C | ^14^ |
|  |  |  | rs73366469 **^[15]^** | 74619286 | intergenic | ENSG00000273069 (lncRNA) |  |  |  | T | C | 0.112 | 3.8E-117 | EAS | 2.38 (2.22-2.56) | C | C | ^27^ (^70,84,116^) |
|  |  |  |  |  |  |  |  |  |  |  |  |  | 2.68E-13 | EU | 1.29 (1.21-1.38) | C | C | ^4^ |
|  |  |  | **rs117026326** ^[15]^ | 74711703 | intron | GTF2I-AS1 (lncRNA); GTF2I (p) |  |  |  | C | T | 0.0194 | 2.20E-298 | EAS | 2.137±0.021 | T | T | ^14^ (^1,114,116^) |
|  |  |  | rs201802880 ^[15]^ | 74779296 | missense | NCF1 (p) | R/H |  |  | G | A | 0.0010 | 3.1E−104 | EAS | 3.47 | A | A | ^116^ (^115^) |
|  |  |  |  |  |  |  |  |  |  |  |  |  | 7.0E-20 | EU | 3.0 (2.4-3.9) | T | A | ^117^ |
|  |  |  | rs199789198 | 74781840 | intron | NCF1 (p) |  |  | NCF1>GTF2IRD2>SPDYE12 | C | A | 0.008 | 2.57E-194 | EAS | 3.098±0.038 | A | A | ^14^ (^24^) |
| 178 | 7q11.23 |  | **rs67955681** | 74625354-74625367 | intergenic | ENSG00000279005 (novel transcript) |  |  | GTF2IRD2>GTF2I>GTF2IRD1 | (A)14 | (A)15 | 0.4475 (ALFA) | 3.68E-10 | EAS | 0.849±0.026 | T (vs TA) | (A)15 | ^14^ |
| 179 | 7q11.23 |  | rs68008267 | 74673141 | intron | GTF2I (p) |  |  | NCF1>SPDYE12>GTF2IRD2 | C | T | 0.099 | 2.65E-12 | EAS | 1.406±0.049 | T | T | ^14^ |
|  |  |  | **rs79171842** | 74684288 | intron | GTF2I (p) |  |  | GTF2I>NCF1>GTF2IRD2 | A | T | 0.002 | 3.02E-23 | EAS | 0.405±0.091 | A | T | ^14^ |
| 180 | 7q11.23 |  | **rs143176121** | 74694107 | intron | GTF2I (p) |  |  | GTF2IRD1>GTF2I>NCF1 | T | C | 0.002 | 1.58E-66 | EAS | 0.292±0.071 | T | C | ^14^ |
| 181 | 7q11.23 |  | **rs587680541** | 74781289-74781291 | intron | NCF1 (p) |  |  | NCF1>GTF2IRD2>SPDYE12 | CCC | CC | 0.001 | 4.24E-62 | EAS | 4.14±0.085 | T (vs TC) | CC | ^14^ |
| 182 | 7q11.23 |  | rs1167796 | 75543861 | intron | HIP1 (p) |  | HIP1 (p):e^1^ |  | G | A | 0.2977 | 2.21E-08 | EAS | 0.83 (0.78-0.89) | A | G | ^21^ |
|  |  |  | rs11773745 | 75542119 | intron | HIP1 (p) |  |  | SPDYE5>TRIM73>HIP1 | G | A | 0.355 | 2.52E-11 | EAS | 1.239±0.032 | A | A | ^1^ |
|  |  |  | **rs794368** | 75556681 | intron | HIP1 (p) |  |  | TRIM73>POM121C>SPDYE5 | G | A | 0.459 | 1.54E-26 | EAS | 1.185±0.016 | A | A | ^27^ |
|  |  |  | rs794356 | 75567227 | intron | HIP1 (p) |  |  | POM121C>TRIM73>HIP1 | G | A | 0.346 | 2.54E-08 | EAS | 0.78 (0.72-0.84) | A | G | ^32^ (^55^) |
| 183 | 7q11.23 |  | rs6964720 | 75551049 | intron | HIP1 (p) |  |  | POM121C>TRIM73>HIP1 | A | G | 0.1328 | 2E-10 | EAS | 1.40 (1.26-1.56) | G | G | ^27^ |
|  |  |  | **rs145931380** | 75552865 | intron | HIP1 (p) |  |  | CCL24>CCL26>HIP1 | (T)_13_ | (T)_14_/(T)_15_ | dupT=0.148 | 2.25E-15 | EAS | 1.38 (1.27-1.49) | I | dupT | ^21^ |
| 184 | 7q11.23 |  | **rs77009341** | 75559377 | intron | HIP1 (p) |  |  | CCL24>HIP1>SPDYE5 | G | C | 0.0036 | 6.39E-62 | EAS | 2.089±0.044 | C | C | ^14^ (^23^) |
|  |  |  | rs139110493 | 75580635 | intron | HIP1 (p) |  |  |  | G | C | 0.003 | 1.21E-12 | EAS | 2.48 (1.93-3.19) | C | C | ^32^ |
| 185 | 7q11.23 |  | **rs4573208** | 75649229 | intron | HIP1 (p) |  |  | HIP1>CCL24>SPDYE5 | G | A | 0.075 | 1.53E-15 | EAS | 1.173±0.02 | A | A | ^14^ |
| 186 | 7q11.23 |  | **rs146063533** | 75692143 | intron | HIP1 (p) |  |  | HIP1>CCL24>CCL26 | C | T | 0.001 | 9.44E-16 | EAS | 1.612±0.059 | T | T | ^14^ |
| 187 | 7q32.1 | rs729302^(119)^  rs12706860^(119)^  rs13245639^(119)^ | rs729302 ^[16]^ | 128928906 | intergenic | IRF5 (p) |  | IRF5 (p):e↑^1,120^,d^1^ | IRF5>KCP>ATP6V1F | A | C | 0.262 | 1.36E-14 | EU | 0.79±0.031 | C | A | ^1^ (^67, 59, 121^) |
|  |  |  |  |  |  |  |  |  |  |  |  |  | 2.80E-41 | Mix | 0.774±0.019 | C | A | ^1^ |
|  |  |  |  |  |  |  |  |  |  |  |  |  | 1.74E-28 | EAS | 0.763±0.024 | C | A | ^1^ (^26, 28^) |
|  |  |  | rs12706860 | 128929972 | intergenic | IRF5 (p) |  |  |  | C | G | 0.305 | 1.98E-12 | MA | 0.66 (0.59-0.74) | C | C | ^4^ |
|  |  |  | rs77090378 | 128932054 | intergenic | IRF5 (p) |  |  |  | G | A | 0.086 | 1.22E-11 | EAS | 0.76 (0.70-0.83) | A | G | ^27^ |
|  |  |  | **rs41298401** | 128938253 | splice region | IRF5 (p) |  |  | IRF5>TNPO3>FLNC | C | G | 0.085 | 5.04E-45 | EAS | 1.294±0.018 | C | C | ^14^ |
| 188 | 7q32.1 | rs2004640^(122)^  rs77571059^(123,124)^  rs3778754^(119)^  rs3807307^(119)^  rs11269962^(119)^  rs4728142^124,125)^ | rs4731532 | 128932712 | intergenic | IRF5 (p) |  | IRF5 (p): e(total;LCLs;PBMCs):e↑^1,119,122,123,125–133^,e(exon1A,1C)↓^122,134^,e(exon 1B;splicing;exon 1B transcripts are expressed only in risk rs2004640-T)↑^122,134^,d^1^ | IRF5>TNPO3>KCP | G | A | 0.335 | 2.57E-13 | AA | 0.74 (0.68-0.80) | C | A | ^4^ |
|  |  |  | rs11269962 (rs113478424) | 128935744-128935758 | intergenic | IRF5 (p) |  |  | IRF5>TNPO3>ATP6V1F | CTTAGCTATTGCTCC | C | 0.368 (TOPMED) | 3.97E-12 | EAS | 1.59 (1.39-1.81) | del | C | ^32^ (^24^) |
|  |  |  | **rs4728142 ^[16]^** | 128933913 | intergenic | IRF5 (p) |  |  | IRF5>TNPO3>KCP | G | A | 0.295 | 3.38E-84 | Mix | 1.44 (1.39-1.50) | T | A | ^4^ (^1,125,^) |
|  |  |  |  |  |  |  |  |  |  |  |  |  | 6.21E-51 | EU | 1.40 (1.34-1.46) | T | A | ^4^ (^1,41,43,121,125^) |
|  |  |  |  |  |  |  |  |  |  |  |  |  | 2.61E-29 | MA | 2.12 (1.88-2.39) | T | A | ^4^ (^35,121^) |
|  |  |  |  |  |  |  |  |  |  |  |  |  | 2.55E-45 | EAS | 1.562±0.032 | A | A | ^1^ (^21,26,29,30,32^) |
|  |  |  |  |  |  |  |  |  |  |  |  |  | 1.16E-12 | AA | 1.35 (1.24-1.47) | T | A | ^4^ (^125^) |
|  |  |  | rs3757387 | 128936032 | intergenic | IRF5 (p) |  |  |  | T | C | 0.295 | 1.14E-48 | EU | 1.45 | C | C | ^5^ (^11^) |
|  |  |  |  |  |  |  |  |  |  |  |  |  | 7.94E-33 | MA | 1.71 (1.56-1.87) | C | C | ^4^ |
|  |  |  |  |  |  |  |  |  |  |  |  |  | 8.73E-79 | EAS | 0.693±0.02 | T | C | ^14^ (^11,27^) |
|  |  |  |  |  |  |  |  |  |  |  |  |  | 1.01E-17 | AA | 1.44 (1.33-1.57) | C | C | ^4^ |
|  |  |  | rs3807135 | 128937563 | 5’ UTR | IRF5 (p) |  |  |  | C | T | 0.4936 | 2E-09 | EU | 1.29 | G | C | ^121^ |
|  |  |  | rs77571059 ^[17]^ | 128937861-128937877 | intron | IRF5 (p) |  |  |  | (GCGGG)_3_GC | (GCGGG)_4_GC | 0.290 | 4.6E-09 | EU | 1.69 (1.42-2.02) | (GCGGG)_4_GC | (GCGGG)_4_GC | ^123^ |
|  |  |  |  |  |  |  |  |  |  |  |  |  | 3.35E-24 | EAS | 1.59 (1.45-1.75) | I | GGGGGG/GAGGGG | ^27^ |
|  |  |  |  |  |  |  |  |  |  |  |  |  | 6.79E-09 | MA | 2.25 (1.70-3.00) | CGGGG | (GCGGG)_4_GC | ^130^ |
|  |  |  |  |  |  |  |  |  |  |  |  |  | 1.58E-19 | Mix | 1.60 (1.44-1.80) | CGGGG | (GCGGG)_4_GC | ^130^ |
|  |  |  | rs2004640 | 128938247 | splice donor | IRF5 (p) |  |  | IRF5>TNPO3>FLNC | G | T | 0.4135 | 1.85E-23 | Mix | 1.44 (1.34–1.55) | T | T | ^135^ (^122,129,130^) |
|  |  |  |  |  |  |  |  |  |  |  |  |  | 6.07E-13 | MA | - | T | T | ^136^ (^130^) |
|  |  |  |  |  |  |  |  |  |  |  |  |  | 2.11E-37 | EU | 1.44 (1.36-1.52) | T | T | ^134^ (^121,122,137^) |
|  |  |  | rs3807307 | 128939148 | intron | IRF5 (p) |  |  | IRF5>TNPO3>KCP | T | C | 0.3365 | 3.75E-62 | EU | 1.46 (1.39-1.52) | C | C | ^4^ |
|  |  |  |  |  |  |  |  |  |  |  |  |  | 6.57E-29 | MA | 1.68 (1.53-1.84) | C | C | ^4^ |
|  |  |  | rs752637 | 128939366 | intron | IRF5 (p) |  |  |  | C | T | 0.491 | 4.09E-12 | EU | 1.34 | ­ | C | ^121^ |
|  |  |  | rs3807306 | 128940626 | intron | IRF5 (p) |  |  |  | G | T | 0.346 | 4.13E-30 | Mix | - | - | T | ^121^ |
|  |  |  |  |  |  |  |  |  |  |  |  |  | 2.7E-19 | EU | 0.70 | - | T | ^121^ (^137^) |
|  |  |  |  |  |  |  |  |  |  |  |  |  | 1.03E-09 | MA | 0.51 | - | T | ^121^ |
|  |  |  | rs7808907 | 128944030 | intron | IRF5 (p) |  |  |  | T | C | 0.4603 | 1.44E-31 | EU | 0.77 (0.74-0.80) | T | C | ^4^ |
|  |  |  | rs2280714 | 128954671 | 3’ UTR | TNPO3 (p) |  |  | TNPO3>IRF5>KCP | T | C | 0.3992 | 1.05E-10 | EU | 0.76 | - | T | ^121^ |
|  |  |  | rs78724056 | 129013997 | intron | TNPO3 (p) |  |  |  | G | T | 0.012 | 1.69E-32 | EAS | 1.596±0.039 | T | T | ^14^ |
|  |  |  | rs10239340 | 129028456 | intron | TNPO3 (p) |  |  | IRF5>TNPO3>KCP | G | T | 0.4499 | 6.98E-16 | EU | 0.77 (0.73-0.82) | T | G | ^67^ |
|  |  |  | rs10279821 ^[16]^ | 129043493 | intron | TNPO3 (p) |  |  |  | C | T | 0.398 | 6.05E-09 | EU | 0.80 (0.74-0.86) | T | C | ^67^ |
| 189 | 7q32.1 | rs34288126^(46)^ | rs35000415 | 128945562 | intron | IRF5 (p) |  | IRF5 (p):i^5^,e^1,5,132^,d^1^  IFNα ↑ and IFN-inducible chemokine expression ↑^132^  circulating type I IFN levels↑^138^ | IRF5>TNPO3>TSPAN33 | C | T | 0.0587 | 1.2E-99 | Mix | 1.82 (1.69-1.96) | T | T | ^4^ |
|  |  |  |  |  |  |  |  |  |  |  |  |  | 5.67E-70 | EU | 1.73 (1.63-1.84) | T | T | ^4^ (^5,11^) |
|  |  |  |  |  |  |  |  |  |  |  |  |  | 7.11E-33 | MA | 1.98 (1.77-2.22) | T | T | ^4^ |
|  |  |  |  |  |  |  |  |  |  |  |  |  | 4.39E-08 | AA | 1.95 (1.53-2.48) | T | T | ^4^ |
|  |  |  | rs2070197 | 128948946 | 3’ UTR | IRF5 (p) |  |  |  | T | C | 0.0591 | 1.98E-60 | EU | 1.82 | C | C | ^5^ (^8^) |
|  |  |  |  |  |  |  |  |  |  |  |  |  | 1.26E-21 | MA | - | C | C | ^136^ (^130,139^) |
|  |  |  |  |  |  |  |  |  |  |  |  |  | 2.24E-23 | Mix | 2.11 (1.81-2.45) | C | C | ^130^ (^129^) |
|  |  |  | **rs10488631** ^[17]^ | 128954129 | intergenic | TNPO3 (p) |  |  |  | T | C | 0.059 | 9.00E-110 | EU | 1.92 (1.81-2.03) | C | C | ^5^ (^9,16,36,43,62,66,68,69,123,140^) |
|  |  |  |  |  |  |  |  |  |  |  |  |  | 2.61E-29 | MA | 2.12 (1.88-2.39) | C | C | ^35^ |
|  |  |  | rs12539741 | 128956751 | intron | TNPO3 (p) |  |  | TNPO3>IRF5>TSPAN33 | C | T | 0.0591 | 6.24E-31 | MA | 2.11 (1.88-2.37) | T | T | ^35^ |
|  |  |  | rs12706861 | 128976528 | intron | TNPO3 (p) |  |  |  | C | T | 0.0589 | 3.85E-71 | EU | 1.76 (1.65-1.87) | T | T | ^4^ |
|  |  |  | rs12531711 | 128977412 | intron | TNPO3 (p) |  |  |  | A | G | 0.0593 | 7.38E-41 | EU | 1.718±0.04 | G | G | ^1^ (^10,59,66^) |
|  |  |  | rs12534421 | [128984019](https://useast.ensembl.org/Homo_sapiens/Location/View?contigviewbottom=variation_feature_variation%3Dnormal%2Cseq%3Dnormal;db=core;r=7:128983969-128984069;source=dbSNP;v=rs12534421;vdb=variation;vf=732507988) | intron | TNPO3 (p) |  |  |  | C | A | 0.059 | 3.9E-44 | Mix | 1.66–2.44 | - | A | ^125^ |
|  |  |  |  |  |  |  |  |  |  |  |  |  | 4.675E-35 | EU | 1.701 | C^#^ | A | ^125^ |
|  |  |  | rs13239597 | 129055929 | non coding transcript exon | TPI1P2 (pseudogene) |  |  |  | C | A | 0.0589 | 9.01E-17 | EU | 1.848 | A | A | ^17^ (^41^) |
|  |  |  | rs12537284 ^[16]^ | 129077852 | intergenic | TNPO3 (p) |  |  |  | G | A | 0.071 | 3.61E-19 | EU | 1.54 (1.40-1.70) | A | A | ^67^ (^10^) |
|  |  |  | rs17340646 | 129082460 | intergenic | TNPO3 (p) |  |  |  | T | G | 0.154 | 8.65E-20 | MA | 1.83 (1.61-2.08) | G | G | ^4^ |
| 190 | 7q32.1 |  | **rs28364822** | 129055928 | non coding transcript exon | TPI1P2 (pseudogene) |  |  | TNPO3>IRF5>TSPAN33 | T | A | 0.001 | 1.19E-17 | EAS | 1.672±0.06 | A | A | ^14^ |
|  |  |  |  |  |  |  |  |  |  |  |  |  |  |  |  |  |  |  |
| 191 | 8p23.1 | rs6985109^(46)^ | rs2955587 | 8240557 | splice region | FAM86B3P (pseudogene) |  | MFHAS1 (p):e^1,141^,d^1^  ERI1 (p):e,d^1^  CLDN23 (p):e^141^  FAM167A (C8orf13):e^141^  FDFT1 (p):e^141^  BLK (p):e^141^  SGK223 (p):e^141^ | ERI1>PRAG1>CLDN23 | A | G | 0.4305 | 5.55E-10 | Mix | 1.11 (1.06-1.17) | C | G | ^4^ |
|  |  |  |  |  |  |  |  |  |  |  |  |  | 7.91E-10 | EU | 1.15 (1.10-1.20) | C | G | ^4^ |
|  |  |  | rs2980512 | 8283379 | intergenic | PRAG1 (p) |  |  |  | T | C | 0.445 | 3.54E-10 | EU | 1.15 (1.10-1.20) | C | C | ^4^ |
|  |  |  | rs2428 | 8783635 | 3’ UTR | MFHAS1 (p) |  |  |  | T | C | 0.4193 | 1.17E-08 | Mix | 1.13 | T | T | ^113^ |
|  |  |  | rs1567398 | 8869294 | intron | MFHAS1 (p) |  |  | ERI1>MFHAS1>CLDN23 | T | G | 0.3466 | 6.06E-09 | EU | 1.17 | A | T | ^141^ |
|  |  |  | rs7831557 | 10422718 | intron | MSRA (p) |  |  | MSRA>SLC35G5>PRSS55 | G | A | 0.279 | 1.22E-09 | EU | 1.14 (1.10-1.20) | G | G | ^4^ |
|  |  |  | rs7819602 | 10869332 | intergenic | ENSG00000280294 (uncategorized) |  |  | BLK>PINX1>FAM167A | G | C | 0.424 | 9.54E-10 | EU | 1.15 (1.10-1.20) | C | C | ^4^ |
|  |  |  | **rs6985109** | 10904075 | intron | XKR6 (p) |  |  | BLK>FAM167A>CTSB | G | A | 0.4413 | 2.51E-11 | EU | 1.23 (1.16-1.3) | G | G | ^67^ (^4^) |
|  |  |  | rs4240671 | 10910238 | intron | XKR6 (p) |  |  | FAM167A>CTSB>PINX1 | G | A | 0.4052 | 6.6E-09 | EU | 0.75 (0.68-0.83) | A | G | ^67^ |
|  |  |  | rs11783247 | 10931365 | intron | XKR6 (p) |  |  | XKR6>FAM167A>BLK | C | T | 0.4503 | 8.00E-10 | EU | 1.21 (1.14-1.28) | C | C | ^67^ |
|  |  |  | rs6984496 | 10938583 | intron | XKR6 (p) |  |  | BLK>FAM167A>CTSB | G | T | 0.4661 | 2.00E-10 | EU | 1.22 (1.14-1.29) | C | G | ^67^ |
|  |  |  | rs4841498 | 11127922 | intron | XKR6 (p) |  |  |  | C | T | 0.4457 | 6.57E-10 | EU | 1.173 | C | C | ^141^ |
|  |  |  | rs4412337 | 11214511 | intergenic | ENSG00000270076 (lncRNA) |  |  | FAM167A>SLC35G5>BLK | A | G | 0.168 | 2.97E-09 | EU | 0.857 | C | A | ^141^ |
|  |  |  | rs7836059 | 11414655 | intron | FAM167A-AS1 (lncRNA) |  |  | BLK>FAM167A>NEIL2 | G | A | 0.4339 | 4.00E-10 | EU | 0.82 (0.78-0.88) | T | G | ^67^ |
|  |  |  | rs17807624 | 11605506 | intergenic | LINC00208 (lncRNA) |  |  | FAM167A>BLK>FDFT1 | C | T | 0.451 | 9.96E-09 | EU | 1.156 | A | T | ^141^ |
| 192 | 8p23.1 | rs922483-T^(142)^  rs1382568-G^(142)^  *TE* | rs12680762 | 11474517 | intron | FAM167A (p) |  | BLK (p):e^2,6,35,141^/↑^143^/↓^68,144^,d^2^  LINC00208 (lncRNA):d^2^  FAM167A-AS1 (C8orf12) (lncRNA): d^2^  CLDN23 (p):e^141^  FAM167A (C8orf13) (p):e↑^68,141,144^  FDFT1 (p):e^141^ | FAM167A>BLK>FDFT1 | C | T | 0.3355 | 1.45E-08 | EU | 1.335 (1.208-1.475) | A | T | ^59^ (^1^) |
|  |  |  | rs2618444 | 11480861 | intron | AF131216.5 |  |  |  | A | C | 0.3642 | 7E-09 | AA | 1.36 (1.22-1.51) | C | C | ^4^ |
|  |  |  | rs2061831 | 11482373 | intergenic | ENSG00000284957 (lncRNA) |  |  |  | T | C | 0.363 | 1.46E-40 | Mix | 1.31 (1.25-1.37) | G | C | ^4^ |
|  |  |  |  |  |  |  |  |  |  |  |  |  | 3.61E-31 | EU | 1.33 (1.27-1.40) | G | C | ^4^ |
|  |  |  |  |  |  |  |  |  |  |  |  |  | 1.8E-08 | AA | 1.36 (1.22-1.51) | G | C | ^4^ |
|  |  |  | **rs2736332** | 11482456 | intergenic | ENSG00000284957 (lncRNA) |  |  |  | C | G | 0.493 | 1.64E-22 | EU | 1.31 | C | C | ^5^ (^11^) |
|  |  |  |  |  |  |  |  |  |  |  |  |  | 1.57E-70 | EAS | 1.36±0.017 | - | C | ^14^ (^11^) |
|  |  |  | rs7812879 | 11482672 | intergenic | ENSG00000284957 (lncRNA) |  |  | BLK>FAM167A>FDFT1 | C | T | 0.185 | 2.09E-24 | EAS | 0.69 (0.64-0.74) | A | C | ^21^ (^25,26^) |
|  |  |  | rs2736336 | 11484361 | intergenic | ENSG00000284957 (lncRNA) |  |  | BLK>FAM167A>GATA4 | G | T | 0.372 | 6.46E-32 | EU | 1.34 (1.28-1.41) | T | T | ^4^ |
|  |  |  | rs978803 | 11485966 | intergenic | ENSG00000284957 (lncRNA) |  |  | FAM167A>BLK>CTSB | A | G | 0.271 | 1.16E-08 | EU | 1.157 | T | A | ^141^ |
|  |  |  | rs2254546 | 11486171 | intergenic | ENSG00000284957 (lncRNA) |  |  | BLK>FAM167A>FDFT1 | G | A | 0.160 | 1.38E-13 | EAS | - | - | G | ^26^ (^28,55^) |
|  |  |  | rs2736340 | 11486464 | intergenic | ENSG00000284957 (lncRNA) |  |  |  | C | T | 0.361 | 6.28E-20 | EU | 1.29 (1.22-1.37) | T | T | ^5^ (^1,8–10,141,144^) |
|  |  |  |  |  |  |  |  |  |  |  |  |  | 2.01E-50 | Mix | 1.363±0.021 | T | T | ^1^ (^144^) |
|  |  |  |  |  |  |  |  |  |  |  |  |  | 1.29E-38 | EAS | 1.403±0.026 | T | T | ^1^ (^29,144^) |
|  |  |  |  |  |  |  |  |  |  |  |  |  | 1.12E-08 | AA | 1.36 (1.22-1.51) | T | T | ^4^ |
|  |  |  | rs1478901 | 11490324 | intron | BLK (p) |  |  | FAM167A>BLK>FDFT1 | G | C | 0.360 | 1.32E-11 | EAS | 0.64 (0.56-0.73 | C^#^ | C | ^142^ |
|  |  |  | rs13277113 | 11491677 | intron | BLK (p) |  |  |  | G | A | 0.3576 | 2.19E-31 | EU | 1.34 (1.27-1.40) | T | A | ^4^ (^16,59,68,144^) |
|  |  |  |  |  |  |  |  |  |  |  |  |  | 2.58E-13 | EAS | 0.69 | G | A | ^29^ (^52,70,144^) |
|  |  |  |  |  |  |  |  |  |  |  |  |  | <1.00E-08 | Mix | 1.359 (1.292-1.429) | A | A | ^144^ |
|  |  |  |  |  |  |  |  |  |  |  |  |  | <1.00E-08 | AA | 1.415 (1.287-1.555) | A | A | ^144^ |
|  |  |  | rs4840568 | 11493510 | intron | BLK (p) |  |  |  | G | A | 0.4377 | 5.18E-18 | EU | 1.31 | - | A | ^11^ |
|  |  |  |  |  |  |  |  |  |  |  |  |  | 1.45E-13 | EAS | 1.47 | - | A | ^11^ |
|  |  |  | rs2736345 | 11494976 | intron | BLK (p) |  |  |  | A | G | 0.4643 | 7.88E-11 | EAS | 0.70 (0.63-0.78) | A | G | ^32^ |
|  |  |  | rs2618476 | 11495032 | intron | BLK (p) |  |  |  | T | C | 0.3648 | 2E-08 | EU | 1.29 | - | C | ^53^ |
|  |  |  | rs998683 | 11495491 | intron | BLK (p) |  |  |  | G | A | 0.362 | 5.22E-14 | EU | 0.76 (0.71-0.82) | G | A | ^142^ |
|  |  |  | rs2618481 | 11496588 | intron | BLK (p) |  |  |  | T | C | 0.355 | 7.8E-10 | EAS | 1.55 (1.35–1.78) | T | T | ^24^ |
|  |  |  | rs2618480 | 11496598 | intron | BLK (p) |  |  | BLK>FAM167A>FDFT1 | C | T | 0.1617 | 1.6E-12 | EAS | 0.7 (0.63-0.77) | T | C | ^23^ |
|  |  |  | rs2248932 | 11534141 | intron | BLK (p) |  |  |  | A | G | 0.4934 | 1.63E-14 | EAS | 0.76 (0.71-0.82) | G | A | ^21^ (^143,144^) |
|  |  |  |  |  |  |  |  |  |  |  |  |  | 7E-10 | EU | 1.22 (1.14-1.3) | T | A | ^67^ |
|  |  |  |  |  |  |  |  |  |  |  |  |  | <1.00E-08 | Mix | 1.285 (1.228-1.345) | T | A | ^144^ |
|  |  |  | rs17153419 | 11536724 | intron | BLK (p) |  |  |  | A | G | 0.368 | 1.28E-10 | EU | 1.191 | C | G | ^141^ |
|  |  |  | rs2618479 | 11498312 | intron | BLK (p) |  |  |  | G | A | 0.155 | 5.26E-21 | EAS | 0.72 (0.67-0.77) | A | G | ^21^ |
| 193 | 8p23.1 |  | **rs5889367** | 11480303-11480304 | intron | ENSG00000284957 (lncRNA) |  |  |  | - | A | 0.00001 | 1.34E-17 | EAS | 0.69 (0.63-0.75) | D | - | ^27^ |
| 194 | 8p23.1 |  | **rs880632** | 11878430 | intergenic | CTSB (p) |  | CLDN23 (p):e^141^  FAM167A (C8orf13) (p):e^141^  FDFT1 (p):e^141^  CTSB (p):e^141^ | FDFT1>CTSB>BLK | C | A | 0.188 | 4.87E-08 | EU | 0.857 | A | C | ^141^ |
| 195 | 8p11.21 | rs1804182^(46)^ | **rs1804182** | 42176001 | stop gain | PLAT (p) | R/* |  | PLAT>DKK4>POLB | G | A | 0.00539 | 3.48E-08 | AA | 1.94 (1.53-2.45) | A | A | ^4^ |
| 196 | 8p11.21 |  | rs117627999 | 42246722 | intron | IKBKB-DT (lncRNA) |  |  | IKBKB>PLAT>AP3M2 | C | T | 0.019 | 1.62E-08 | EAS | 0.827±0.034 | T | C | ^14^ |
|  |  |  | **rs2272736** | 42319645 | missense | IKBKB (p) | R/Q |  | IKBKB>AP3M2>POLB | G | A | 0.024 | 6.37E-11 | EAS | 0.819±0.03 | A | G | ^14^ |
| 197 | 8q12.1 |  | **rs7829816** | 55936827 | intron | LYN (p) |  |  | LYN>TGS1>TMEM68 | A | G | 0.2304 | 5.40E-09 | EU | 0.77 (0.70-0.84) | C | A | ^67^ |
| 198 | 8q12.1 |  | **rs2953898** | 56068244 | 3’ UTR | RPS20 (p) |  |  | LYN>RPS20>PLAG1 | C | T | 0.0867 | 4.43E-08 | Mix | 0.84 (0.79-0.90) | A | C | ^4^ |
| 199 | 8q13.3 |  | rs13260060 | 70306125 | intron | NCOA2 (p) |  | NCOA2 (p):d^1^ | NCOA2>XKR9>TRAM1 | G | A | 0.188 | 1.92E-08 | EAS | 0.889±0.021 | A | G | ^1^ |
|  |  |  | rs11347613 | 70397462-70397478 | intron | NCOA2 (p) |  |  |  | (A)_17_ | (A)_16_ | 0.279 | 2.34E-12 | EAS | 0.888±0.017 | G (vs GA) | (A)_17_ | ^14^ |
|  |  |  | **rs142937720** | 70417932-70417940 | intergenic | NCOA2 (p) |  |  |  | AGTGGCCAG | AG | 0.188 | 2.27E-12 | EAS | 0.894±0.016 | A | AGTGGCCAG | ^14^ |
| 200 | 8q13.3 |  | rs10111413 | 71979513 | intron | MSC-AS1 (lncRNA) |  |  |  | G | A | 0.318 | 3.05E-09 | EAS | 0.918±0.014 | A | G | ^14^ |
|  |  |  | rs17374162 | 71982724 | intron | MSC-AS1 (lncRNA) |  |  | MSC>TRPA1>EYA1 | G | A | 0.325 | 3.02E-09 | EAS | 0.917±0.015 | A | G | ^14^ |
| 201 | 8q21.13 |  | **rs117821148** | 77195989 | intergenic | ENSG00000288966 (lncRNA) |  |  | PEX2 | C | T | 0.005 | 4.8E-08 | EAS | 1.46±0.07 | T | T | ^2^ |
| 202 | 8q21.12 |  | **rs4739134** | 78643913 | intron | ENSG00000286675 (lncRNA) |  |  | ZC2HC1A>IL7>PKIA | C | T | 0.2498 | 3.47E-08 | Mix | 1.12 (1.07-1.17) | T | T | ^4^ |
| 203 | 8q22.3 | **rs13259960**^(145)^ | **rs13259960** | 101073779 | intron | FLJ42969 (lncRNA) |  | FLJ42969 (SLEAR) (lncRNA):e↑,3C^145^ | YWHAZ>ZNF706>PABPC1 | A | G | 0.144 | 1.03E-11 | EAS | 1.35 (1.22-1.45) | G | G | ^145^ |
| 204 | 8q24.21 |  | **rs16902895** | 128413347 | intron | LINC00824 (lncRNA) |  | LINC00824 (lncRNA):d^1^ |  | A | G | 0.181 | 1.48E-13 | EAS | 1.122±0.016 | A | A | ^14^ |
|  |  |  | rs7815944 | 128415272 | intron | LINC00824 (lncRNA) |  |  |  | A | G | 0.195 | 1.78E-09 | EAS | 0.862±0.025 | G | A | ^1^ |
|  |  |  |  |  |  |  |  |  |  |  |  |  |  |  |  |  |  |  |
| 205 | 9p24.1 |  | **rs1887428** | 4984530 | 5’ UTR | JAK2 (p) |  | JAK2 (p):i^11^ | JAK2>RCL1>AK3 | C | G | 0.3073 | 2.19E-17 | Mix | 1.16 (1.12-1.20) | G | G | ^11^ |
|  |  |  |  |  |  |  |  |  |  |  |  |  | 4.49E-14 | EAS | 1.24 (1.17-1.31) | G | G | ^11^ (^14^) |
| 206 | 9p21.3 |  | **rs7858766** | 21267088 | intergenic | IFNA22P (pseudogene) |  |  | KLHL9>HACD4>IFNA10 | T | C | 0.372 | 2.25E-15 | EAS | 1.139±0.016 | T | T | ^14^ |
| 207 | 9q22.33 |  | **rs11788118** | 99575049 | intron | LOC101928438 (lncRNA); STX17-DT (lncRNA); ENSG00000234860 (lncRNA) |  |  | NR4A3>STX17>SEC61B | G | A | 0.1416 | 1.53E-08 | Mix | 0.88 (0.84-0.92) | A | G | ^4^ |
| 208 | 9q22.33 |  | **rs1405209** | 99823263 | intron | NR4A3 (p); STX17-DT (lncRNA) |  | NR4A3 (p):d^1^ | STX17>NR4A3>INVS | T | C | 0.261 | 2.42E-08 | Mix | 1.112±0.019 | C | C | ^1^ |
|  |  |  |  |  |  |  |  |  |  |  |  |  |  |  |  |  |  |  |
| 209 | 10p15.1 |  | **rs77448389** | 5868783 | intron | ANKRD16 (p) |  |  | GDI2>FBH1>ANKRD1 | A | G | 0.081 | 7.30E-10 | EAS | 0.855±0.025 | A | G | ^14^ |
| 210 | 10p13 |  | **rs10795956** | 12446137 | intron | CAMK1D (p) |  |  | CAMK1D>CDC123>NUDT5 | G | A | 0.314 | 2.87E-08 | Mix | 1.11±0.02 | A | A | ^2^ |
| 211 | 10p13 |  | **rs7911501** | 14394837 | intron | FRMD4A (p) |  |  | FAM107B>FRMD4A>CDNF | G | A | 0.081 | 2.0E-09 | EAS | 2.1 (1.65-2.67) | A | A | ^24^ |
| 212 | 10q11.23 |  | **rs7072606** | 48725929 | missense | WDFY4 (p) | S/P |  | WDFY4>ARHGAP22>LRRC18 | T | C | 0.140 | 2.22E-12 | EAS | 0.884±0.018 | T | C | ^14^ |
| 213 | 10q11.23 | rs877819^(146)^  *TE* | rs10857635 | 48766611 | intron | WDFY4 (p) |  | WDFY4 (p):c^5^,e↓^1,5,146^,d^1^ | ARHGAP22>WDFY4>LRRC18 | T | G | 0.3209 | 6.87E-10 | Mix | 0.86 (0.82-0.91) | C | T | ^4^ |
|  |  |  |  |  |  |  |  |  |  |  |  |  | 3.38E-08 | EU | 0.84 (0.79-0.89) | C | T | ^4^ |
|  |  |  | **rs7097397** | 48817351 | missense | WDFY4 (p) | R/Q |  | WDFY4>ARHGAP22>LRRC18 | G | A | 0.3586 | 2.23E-40 | EAS | 0.81±0.016 | A | G | ^14^ (^1,27,32,57^) |
|  |  |  |  |  |  |  |  |  |  |  |  |  | 1.82E-32 | Mix | 0.804±0.018 | A | G | ^1^ |
|  |  |  |  |  |  |  |  |  |  |  |  |  | 8.69E-12 | EU | 1.2 | G | G | ^5^ (^1,11^) |
|  |  |  | rs877819 | 48834906 | intron | WDFY4 (p) |  |  |  | G | A | 0.4653 | 2.45E-09 | EAS | - | - | A | ^29^ (^26,28,57^) |
|  |  |  | rs2663052 | 48861350 | intron | WDFY4 (p) |  |  | WDFY4>LRRC18>ARHGAP22 | A | G | 0.3694 | 5.25E-09 | EU | 1.16 (1.10 - 1.22) | C | G | ^5^ (^9^) |
|  |  |  | rs11101537 | 48868280 | intron | WDFY4 (p) |  |  |  | T | G | 0.2145 | 4.2E-9 | EAS | 1.3 (1.19-1.42) | T | T | ^23^ (^24^) |
|  |  |  | rs2663054 | 48893932 | intron | ENSG00000241577 (lncRNA); WDFY4 (p) |  |  | TMEM273>WDFY4>LRRC18 | A | G | 0.3472 | 1.49E-08 | EU | 0.83 (0.78-0.88) | A | G | ^4^ |
|  |  |  | rs1913517 | 48911009 | intron | ENSG00000241577 (lncRNA); WDFY4 (p); LRRC18 (p) |  |  | TMEM273>LRRC18>ARHGAP22 | G | A | 0.3838 | 7.22E-12 | EAS | 1.24 (1.17-1.32) | A | A | ^21^ (^30^) |
| 214 | 10q21.2 |  | **rs7902146** | 62041271 | intron | ARID5B (p) |  | ARID5B (p):i^5^,e^1^,d^1^ | ARID5B>RTKN2>ZNF365 | T | C | 0.345 | 3.34E-12 | EAS | 0.9±0.015 | T | C | ^14^ |
|  |  |  | rs4948496 | 62045858 | intron | ARID5B (p) |  |  |  | C | T | 0.3848 | 5.1E-11 | EAS | 0.85 | T | C | ^28^ |
|  |  |  |  |  |  |  |  |  |  |  |  |  | 1.29E-10 | Mix | 1.139±0.02 | C | C | ^1^ |
|  |  |  |  |  |  |  |  |  |  |  |  |  | 1.04E-10 | EU | 1.14 (1.10-1.19) | C | C | ^5^ |
| 215 | 10q21.2 |  | rs10995254 | 62642076 | intron | [ENSG00000285551](https://useast.ensembl.org/Homo_sapiens/Gene/Variation_Gene/Table?db=core;g=ENSG00000285551;source=dbSNP;v=rs10995254;vdb=variation;vf=168963944) (lncRNA); [ENSG00000285837](https://useast.ensembl.org/Homo_sapiens/Gene/Variation_Gene/Table?db=core;g=ENSG00000285837;source=dbSNP;v=rs10995254;vdb=variation;vf=168963944) (p) |  | ZNF365 (p):e^1^ | ADO>ZNF365>EGR2 | T | G | 0.259 | 4.47E-08 | EAS | 1.092±0.016 | T | T | ^14^ |
|  |  |  | **rs10995261** | 62651528 | intron | ENSG00000285551 (lncRNA) |  |  |  | C | T | 0.184 | 2.57E-08 | EAS | 0.909±0.017 | T | C | ^14^ |
|  |  |  | rs4745876 | 62665366 | intron | ENSG00000285551 (lncRNA) |  |  |  | G | A | 0.189 | 3.66E-08 | Mix | 0.882±0.023 | A | G | ^1^ |
| 216 | 10q22.1 |  | rs780669 | 71352484 | non coding transcript exon | SLC29A3 (p) |  | SLC29A3 (p):e↓^2^ | SLC29A3>CDH23>UNC5B | T | C | 0.336 | 4.83E-09 | EAS | 1.16±0.03 | T | T | ^2^ |
|  |  |  |  |  |  |  |  |  |  |  |  |  | 3.86E-08 | Mix | 1.13±0.02 | T | T | ^2^ |
| 217 | 10q22.1 |  | **rs10823829** | 71706952 | synonymous | CDH23 (p) |  | CDH23 (p):e,d^1^ | CDH23>C10orf105>VSIR | T | C | 0.207 | 1.05E-09 | EAS | 0.910±0.016 | T | C | ^14^ |
|  |  |  | rs10999979 | 71741309 | intron | CDH23 (p) |  |  | VSIR>C10orf105>PSAP | C | A | 0.057 | 4.65E-08 | Mix | 1.165±0.028 | A | A | ^1^ |
| 218 | 10q24.33 |  | **rs4917385** | 103243964 | intron | NT5C2 (p) |  |  | ATP5MK>NT5C2>INA | G | T | 0.312 | 1.39E-08 | MA | 0.72 (0.65-0.80) | T | G | ^35^ |
| 219 | 10q24.33 |  | **rs111447985** | 103918153 | 5’ UTR | STN1 (p) |  |  | STN1>SFR1>CFAP43 | C | A | 0.027 | 1.72E-08 | EAS | 1.172±0.028 | A | A | ^14^ |
| 220 | 10q25.2 |  | **rs58164562** | 110904356 | non coding transcript exon | BBIP1 (p) |  |  | PDCD4>BBIP1>DUSP5 | T | C | 0.165 | 3.14E-12 | EAS | 0.892±0.016 | T | C | ^14^ |
|  |  |  |  |  |  |  |  |  |  |  |  |  |  |  |  |  |  |  |
| 221 | 11p15.5 |  | rs12802200 | 566936 | non coding transcript exon | MIR210HG (lncRNA) |  | IRF7 (p):i^5^,c^4,5^,e^1,5^,d^1^ | IRF7>ANO9>LRRC56 | C | A | 0.155 | 8.81E-10 | EU | 1.23 (1.15-1.31) | C | C | ^5^ (^1,9^) |
|  |  |  |  |  |  |  |  |  |  |  |  |  | 1.59E-09 | Mix | 0.82±0.033 | A | C | ^1^ |
|  |  |  | rs4963128 | 589564 | intron | PHRF1 (p) |  |  | IRF7>LRRC56>PTDSS2 | C | T | 0.2947 | 1.89E-15 | EU | 0.83 (0.79-0.87) | T | C | ^4^ (^8,16,67^) |
|  |  |  | **rs1131665** | 613208 | missense | IRF7 (p) | Q/R |  | IRF7>DRD4>PTDSS2 | T | C | 0.2756 | 9.36E-21 | Mix | 0.84 (0.81-0.87) | G | T | ^4^ |
|  |  |  |  |  |  |  |  |  |  |  |  |  | 1.11E-16 | EU | 0.81 (0.77-0.85) | G | T | ^4^ |
|  |  |  | rs1061502 | 614318 | missense | IRF7 (p) | K/E |  | IRF7>DRD4>ANO9 | T | C | 0.2756 | 3.47E-12 | EU | 1.22 | T | T | ^5^ (^1,11^) |
|  |  |  |  |  |  |  |  |  |  |  |  |  | 1.11E-09 | Mix | 0.835±0.03 | C | T | ^1^ |
|  |  |  | rs112006329 | 617228 | 3’ UTR | CDHR5 (p) |  |  | IRF7>DRD4>LRRC56 | G | A | 0.2762 | 1.06E-16 | EU | 0.81 (0.77-0.85) | A | G | ^4^ |
|  |  |  | rs11246217 | 623765 | intron | CDHR5 (p) |  |  | IRF7>DRD4>ANO9 | A | G | 0.2354 | 1.11E-16 | EU | 0.81 (0.77-0.85) | C | A | ^4^ |
|  |  |  | rs58688157 | 625085 | 5’ UTR | CDHR5 (p) |  |  |  | A | G | 0.2867 | 4.95E-13 | EU | 1.24 | A | A | ^5^ |
|  |  |  | rs12418883 | 634083 | intergenic | DRD4 (p) |  |  | IRF7>LRRC56>TMEM80 | G | C | 0.273 | 3.51E-11 | EU | 1.24 | - | G | ^11^ |
| 222 | 11p15.5 |  | **rs6598011** | 577809 | intron | PHRF1 (p) |  |  | TMEM80>EPS8L2>IRF7 | C | T | 0.3089 | 1.12E-09 | EU | 1.15 (1.10-1.21) | A | T | ^4^ |
| 223 | 11p15.4 |  | rs3750996 | 4091970 | 3’ UTR | STIM1 (p) |  |  | STIM1>RHOG>PGAP2 | A | G | 0.060 | 1.89E-12 | EAS | 1.167±0.022 | A | G | ^14^ |
| 224 | 11p15.1 |  | rs77885959 | 18340835 | intron | GTF2H1 (p) |  |  | GTF2H1>LDHA>SPTY2D1 | T | G | 0.004 | 3.16E-17 | EAS | 1.694±0.062 | T | T | ^14^ |
| 225 | 11p13 |  | rs2732552 | 35063045 | intron | ENSG00000289526 (lncRNA) |  | CD44 (p):i^5^,e^34^  PDHX (p):e^1^ | APIP>CD44>PDHX | C | T | 0.427 | 1.13E-16 | Mix | 1.174±0.019 | C | C | ^1^ (^147^) |
|  |  |  |  |  |  |  |  |  |  |  |  |  | 1.74E-08 | EAS | 1.162±0.027 | C | C | ^1^ |
|  |  |  |  |  |  |  |  |  |  |  |  |  | 1.02E-09 | EU | 1.188±0.028 | C | C | ^1^ (^147^) |
|  |  |  | **rs2732549** | 35066852 | non coding transcript exon | ENSG00000289526 (lncRNA) |  |  |  | A | G | 0.217 | 1.2E-23 | EU | 1.24 (1.19-1.29) | T | A | ^5^ (^9^) |
|  |  |  | rs2732547 | 35067136 | non coding transcript exon | ENSG00000289526 (lncRNA) |  |  |  | C | T | 0.413 | 1.55E-11 | EAS | 0.82 (0.77-0.87) | A | C | ^31^ |
|  |  |  | rs387619 | 35063045 | intergenic | ENSG00000289526 (lncRNA) |  |  |  | C | T | 0.242 | 2.84E-13 | EU | 1.21 | C | C | ^5^ (^11,147^) |
|  |  |  | rs2785198 | 35071482 | intergenic | ENSG00000289526 (lncRNA) |  |  |  | A | G | 0.237 | 1.30E-20 | EAS | 1.181±0.018 | A | A | ^14^ |
|  |  |  | rs353592 | 35076646 | intergenic | ENSG00000289526 (lncRNA) |  |  |  | A | G | 0.292 | 1E-08 | Mix | 0.89 (0.85-0.93) | T | G | ^4^ |
|  |  |  | rs507230 | 35107625 | intergenic | CD44-DT (lncRNA) |  |  | CD44>PDHX>APIP | A | G | 0.292 | 3.98E-12 | EU | 0.72 (0.61-0.86) | G | A | ^94^ |
| 226 | 11p13 |  | **rs11032994** | 35089610 | intergenic | ENSG00000289526 (lncRNA) |  |  | CD44>PDHX>APIP | G | A | 0.172 | 1.12E-17 | EAS | 1.147±0.016 | A | A | ^14^ |
| 227 | 11q13.1 |  | rs2009453 | 65632057 | intron | PCNX3 (p) |  | RNASEH2C (p):e↓^11^  SIPA1 (p):e^1^,^27^,d^1^  RELA (p): e^1^,^27^,d^1^  MAP3K11 (p):e,d^1^  PCNX3 (PCNXL3) (p):e^1,27^,d^1^ | RNASEH2C>KAT5>OVOL1 | C | T | 0.4445 | 1.96E-11 | EAS | 0.859±0.023 | T | C | ^1^ (^27^) |
|  |  |  |  |  |  |  |  |  |  |  |  |  | 4.22E-12 | Mix | 0.885±0.018 | T | C | ^1^ |
|  |  |  | **rs10896045** | 65788053 | intron | OVOL1 (p) |  |  | CFL1>MUS81>OVOL1 | G | A | 0.476 | 6.59E-26 | EAS | 1.168±0.015 | A | A | ^14^ |
|  |  |  | rs494003 | 65774827 | 3’ UTR | AP5B1 (p) |  |  | RNASEH2C>KAT5>MUS81 | G | A | 0.1554 | 5.81E-09 | Mix | 1.14 (1.09-1.19) | A | A | ^11^ (^1^) |
|  |  |  |  |  |  |  |  |  |  |  |  |  | 4.92E-08 | EU | 1.204±0.034 | A | A | ^1^ |
| 228 | 11q13.1 |  | **rs1308020** | 65730087 | intergenic | RNASEH2C (p) |  | SIPA1 (p):e,d^1^  RELA (p):e,d^1^  MAP3K11 (p):e^1,76^,d^1^  PCNXL3 (p):e,d^1^  SCYL1 (p):e^76^  SIPA1 (p):e^76^  FIBP (p):e^76^  FAM89B (p):e^76^  EFEMP2 (p):e^76^  CTSW (p):e^76^ | BANF1>MAP3K11>KAT5 | G | A | 0.245 | 2.96E-19 | Mix | 0.84 (0.81-0.88) | T | G | ^76^ (^1^) |
|  |  |  |  |  |  |  |  |  |  |  |  |  | 1.77E-09 | EAS | 0.78 (0.73-0.83) | T | G | ^76^ (^1,27^) |
| 229 | 11q13.3 | rs10750836^((30)^ | rs10750836 | 69048055 | intergenic | TPCN2 (p) |  | TPCN2 (p):e↓^30^ | TPCN2>MRPL21>MRGPRD | T | C | 0.369 | 2.17E-08 | EAS | 1.115±0.019 | G | C | ^30^ |
|  |  |  | **rs4930642** | 69048902 | intergenic | TPCN2 (p) |  |  | TPCN2>MRPL21>IGHMBP2 | G | A | 0.191 | 6.16E-13 | EAS | 1.145±0.019 | A | A | ^14^ |
| 230 | 11q13.4 | rs12792306^(46)^  rs732934^(46)^ | **rs3794060** | 71476633 | 3’ UTR | NADSYN1 (p) |  | DHCR7 (p):e^5^  NADSYN1 (p):e^5,34^ | NADSYN1>DHCR7>KRTAP5-7 | C | T | 0.3466 | 1.32E-20 | EU | 1.23 (1.18-1.29) | C | C | ^5^ |
| 231 | 11q13.4 |  | rs11235604 | 72822491 | missense | ATG16L2 (p) | R/W | ATG16L2 (p):d^1^ | ATG16L2>FCHSD2>STARD10 | C | T | 0.0202 | 8.87E-12 | Mix | 0.78 (0.71-0.85) | T | C | ^76^ |
|  |  |  |  |  |  |  |  |  |  |  |  |  | 1.90E-09 | EAS | 0.76 (0.69-0.84) | T | C | ^27^ (^76^) |
|  |  |  | **rs77971648** | 72929435 | intron | FCHSD2 (p) |  |  | FCHSD2>ATG16L2>ARAP1 | T | C | 0.021 | 3.16E-23 | EAS | 1.291±0.026 | T | T | ^14^ |
|  |  |  | rs11235667 | 73152652 | intergenic | IUR1 (lncRNA) |  |  | FCHSD2>ARAP1>ATG16L2 | A | G | 0.022 | 6.67E-11 | EAS | 0.63 (0.55-0.72) | G | A | ^32^ (^2,27^) |
| 232 | 11q14.3 |  | **rs372605131** | 89805517 | intron | TRIM49 (p) |  | NAALAD2 (p):e↑^24^ |  | T | C | 0.171 | 1.2E-08 | EAS | 1.78 (1.46–2.16) | C | C | ^24^ |
| 233 | 11q23.3 |  | rs11603023^[18]^ | 118615352 | intron | PHLDB1 (p) |  | DDX6 (p):e^1^ | PHLDB1>ARCN1>TREH | C | T | 0.3994 | 1.25E-08 | EAS | 1.20 (1.12-1.27) | T | T | ^148^ |
|  |  |  | rs4639966^[18]^ | 118702810 | intron | ENSG00000255422 (lncRNA) |  |  | PHLDB1>DDX6>IFT46 | T | C | 0.2756 | 1.25E-16 | EAS | 1.29 (1.22-1.37) | G | C | ^21^ (^1^) |
|  |  |  | rs480958 | 118707281 | intron | ENSG00000255422 (lncRNA) |  |  | PHLDB1>DDX6>MPZL2 | A | G | 0.451 | 3.42E-19 | EAS | 0.871±0.015 | A | G | ^14^ |
|  |  |  | **rs377392985** | between 118780114&118780115 | intron | DDX6 (p) |  |  |  | - | (A)_9_ | 0.314 | 2.86E-19 | EAS | 1.161±0.017 | C(A)_9_ (vs C) | (A)_9_ | ^14^ |
|  |  |  | rs10892301^[18]^ | 118864767 | intergenic | CXCR5 (p) |  |  | PHLDB1>DDX6>MPZL2 | G | A | 0.452 | 2.51E-08 | EAS | 0.85 (0.80-0.90) | G | A | ^148^ |
| 234 | 11q23.3 |  | **rs4936441** | 118854951 | intergenic | CXCR5 (p) |  |  | TREH>DDX6>PHLDB1 | G | C | 0.195 | 5.71E-16 | EAS | 0.823±0.024 | C | G | ^14^ |
| 235 | 11q24.3 | rs12576753^(46)^  *TEx2* | rs12576753 | 128434246 | intergenic | ETS1 (p) |  | ETS1 (p):i^5^,e↓^1,57^,d^1^  FLI1 (p):i^5^ | ETS1>FLI1>KCNJ5 | C | A | 0.170 | 1.8E-12 | EAS | 1.36 (1.25-1.48) | A | A | ^23^ |
|  |  |  | **rs9736939** | 128435976 | intergenic | ETS1 (p) |  |  |  | G | A | 0.332 | 1.23E-58 | EAS | 1.265±0.015 | A | A | ^14^ |
|  |  |  | rs6590330 | 128441164 | intergenic | ETS1 (p) |  |  |  | G | A | 0.221 | 1.77E-25 | EAS | 1.37 (1.29-1.45) | A | A | ^21^ (^28,29,55^) |
|  |  |  | rs11501246 | 128444529 | intergenic | ETS1 (p) |  |  | ETS1>FLI1>KCNJ1 | A | G | 0.234 | 1.58E-58 | EAS | 0.791±0.015 | A | G | ^14^ |
|  |  |  | rs12574073 | 128449583 | intergenic | ETS1 (p) |  |  |  | C | T | 0.173 | 2.68E-14 | Mix | 1.24 (1.17-1.32) | A | T | ^4^ |
|  |  |  |  |  |  |  |  |  |  |  |  |  | 1.45E-09 | EU | 1.24 (1.16-1.33) | A | T | ^4^ |
|  |  |  | rs61432431 | 128452727 | intergenic | ETS1 (p) |  |  |  | T | C | 0.145 | 4.56E-28 | EAS | 1.46 (1.37-1.56) | C | C | ^27^ (^11^) |
|  |  |  | rs12575600 | 128454974 | intergenic | ETS1 (p) |  |  |  | C | G | 0.172 | 5.96E-10 | EU | 1.24 (1.16-1.33) | G | G | ^4^ |
|  |  |  | rs1128334 | 128459064 | 3’ UTR | ETS1 (p) |  |  |  | C | T | 0.1713 | 6.41E-49 | Mix | 1.355±0.021 | T | T | ^1^ |
|  |  |  |  |  |  |  |  |  |  |  |  |  | 2.52E-08 | EU | 1.287±0.045 | T | T | ^1^ |
|  |  |  |  |  |  |  |  |  |  |  |  |  | 1.70E-42 | EAS | 1.373±0.023 | T | T | ^1^ (^32,57^) |
| 236 | 11q24.3 |  | **rs7941765** | 128629105 | non coding transcript exon | ENSG00000288975 (lncRNA), LOC101929538 (lncRNA) |  | ETS1 (p):e^1^,d^1^ | ETS1>FLI1>KCNJ5 | C | T | 0.2802 | 1.35E-10 | EU | 1.14 (1.10-1.19) | C | C | ^5^ |
|  |  |  |  |  |  |  |  |  |  |  |  |  | 6.37E-09 | Mix | 1.134±0.022 | C | C | ^1^ |
| 237 | 11q24.3 |  | **rs684150** | 128724266 | intron | FLI1 (p) |  |  | FLI1>ETS1>KCNJ5 | C | T | 0.390 | 4.32E-10 | EAS | 0.914±0.014 | T | C | ^14^ |
|  |  |  |  |  |  |  |  |  |  |  |  |  |  |  |  |  |  |  |
| 238 | 12p13.32 |  | **rs2540119** | 4031710 | intergenic | ENSG00000256969 (lncRNA) |  |  | CCND2>PARP11>CRACR2A | C | T | 0.296 | 3.51E-08 | EAS | 1.086±0.015 | T | T | ^14^ |
| 239 | 12p13.2 | *TE* | **rs12822507 ^[19]^** | 12620587 | intron | CREBL2 (p) |  |  | CREBL2>APOLD1>GPR19 | A | G | 0.3636 | 2.20E-08 | EAS | 0.86 | G | A | ^28^ |
| 240 | 12p13.1 |  | rs11055009 | 12665031 | intron | GPR19 (p) |  | GPR19 (p):e^1^ | APOLD1>GPR19>CDKN1B | T | G | 0.155 | 8.8E-09 | EAS | 0.69 (0.61-0.79) | G | T | ^23^ |
|  |  |  | rs10845602 | 12672303 | intron | GPR19 (p) |  |  | GPR19>APOLD1>CDKN1B | A | G | 0.273 | 5.77E-31 | EAS | 1.21±0.016 | A | A | ^14^ |
|  |  |  | rs10845606^[19]^ | 12681960 | intron | ENSG00000257004 (lncRNA); GPR19 (p) |  |  |  | C | A | 0.1583 | 3.80E-17 | EAS | 0.79 | A | C | ^28^ (^1^) |
|  |  |  |  |  |  |  |  |  |  |  |  |  | 1.90E-08 | Mix | 0.878±0.023 | A | C | ^1^ |
|  |  |  | **rs4251697** | 12721528 | 3’ UTR | CDKN1B (p) |  |  | CDKN1B>BORCS5>MANSC1 | G | A | 0.030 | 1.17E-43 | EAS | 0.637±0.033 | A | G | ^14^ (^24^) |
| 241 | 12p13.1 | **rs34330(**^149^) | **rs34330^[19]^** | 12717761 | 3’ UTR | CDKN1B (p) |  | CDKN1B (p):e↓^28^/↑^149^,f(CRISPR-based)  APOLD1 (p):e,3D,f(CRISPR-based)^149^  DDX47 (p):e,3D,f(CRISPR-based)^149^  RPL37AP9 (ENSG0000024232, RP11-59H1.4) (pseudogene):e^149^ GPR19 (p):3D^149^,f(CRISPR-based)^149^ Proliferation, apoptosis^149^ | APOLD1>CDKN1B>BORCS5 | C | T | 0.338 | 4.8E-12 | EAS | 0.84 | T | C | ^28^ |
|  |  |  |  |  |  |  |  |  |  |  |  |  | 5.29E-22 | Mix | 0.84 (0.81-0.87) | T | C | ^149^ |
| 242 | 12q12 |  | **rs10506216** | 42737083 | intergenic | LINC02450 (lncRNA) |  | PRICKLE1 (p):e^96^  PPHLN1 (p):e^96^  IRAK4 (p):e^96^ | PRICKLE1>ZCRB1>PPHLN1 | G | A | 0.166 | 3.08E-08 | EU | - | T | T | ^96^ |
| 243 | 12q23.2 |  | **rs4622329** | 101928157 | intron | DRAM1 (p) |  |  | DRAM1>CHPT1>WASHC3 | A | G | 0.4842 | 4.00E-15 | EAS | 1.119±0.014 | G | G | ^14^ (^28^) |
| 244 | 12q23.3 |  | rs10082873 | 103511597 | intergenic | ENSG00000286197 (lncRNA) |  | STAB2 (p):e↓^84^ | ASCL1>PAH>STAB2 | A | G | 0.467 | 4.08E-08 | Mix | 0.89±0.021 | G | A | ^84^ |
|  |  |  | **rs6539078** | 103522302 | intron | ENSG00000286197 (lncRNA) |  |  | PAH>STAB2>ASCL1 | T | C | 0.388 | 9.49E-14 | EAS | 0.894±0.015 | T | C | ^14^ |
| 245 | 12q24.12 |  | **rs10774625** | 111472415 | intron | ATXN2 (p) |  | SH2B3 (p):i^5^,e^1^,d^1^  TRAFD1 (p):e^1^,d^1^  PTPN11 (p):e^1^,d^1^ | SH2B3>PHETA1>ALDH2 | G | A | 0.1512 | 7.28E-12 | Mix | 0.827±0.028 | G | A | ^1^ (^5^) |
|  |  |  |  |  |  |  |  |  |  |  |  |  | 4.09E-09 | EU | 1.13 (1.08–1.18) | A | A | ^5^ |
|  |  |  | rs597808 | 111535554 | intron | ATXN2 (p) |  |  | SH2B3>PHETA1>ALDH2 | G | A | 0.148 | 6.47E-10 | EU | 1.18 | A | A | ^5^ |
|  |  |  | rs653178 | 111569952 | intron | ATXN2 (p) |  |  | SH2B3>ALDH2>PHETA1 | T | C | 0.1474 | 7.39E-09 | Mix | 1.14 (1.08-1.20) | C | C | ^4^ |
| 246 | 12q24.12 |  | **rs77465633** | 111495741 | intron | ATXN2 (p) |  |  | ATXN2>SH2B3>PHETA1 | C | A | 0.013 | 6.99E-18 | EAS | 1.34±0.034 | A | A | ^14^ |
|  |  |  | rs76596471 | 111974714 | intron | TMEM116 (p) |  |  | TMEM116>ERP29>NAA25 | T | C | 0.010 | 6.60E-12 | EAS | 1.45±0.05 | C | C | ^2^ |
| 247 | 12q24.13 |  | **rs1131476** | 112919404 | missense | OAS1 (p) | A/T |  | OAS1>OAS2>OAS3 | A | G | 0.212 | 1.25E-09 | EAS | 1.11±0.017 | A | A | ^14^ |
| 248 | 12q24.23 |  | **rs428073** | 118244946 | missense | TAOK3 (p) | S/N |  | TAOK3>PEBP1>VSIG10 | T | C | 0.269 | 2.52E-08 | Mix | 1.12±0.02 | T | T | ^2^ |
| 249 | 12q24.31 |  | rs7975703 | 120661499 | non coding transcript exon | CABP1 (p) |  | UNC119B (p):e^1^  COQ5 (p):e^1^  MLEC (p):e^1^  CABP1 (p):e^1^ | UNC119B>MLEC>CABP1 | C | T | 0.352 | 2.99E-08 | Mix | 0.884±0.022 | T | C | ^1^ |
|  |  |  | rs35743748 | 120790827-120790839 | intron | SPPL3 (p) |  |  | SPPL3>ACADS>MLEC | (T)_13_ | (T)_14_ | 0.456 | 2.45E-09 | EAS | 1.101±0.016 | CT (vs C) | (T)_14_ | ^14^ |
|  |  |  | **rs3999421** | 120930715 | intergenic | HNF1A-AS1 (lncRNA) |  |  | SPPL3>OASL>C12orf43 | A | T | 0.398 | 1.29E-09 | EAS | 0.910±0.016 | A | T | ^14^ |
| 250 | 12q24.32-12q24.33 |  | 12:129276658:I (b37)*** (rs41508750?) | 128792113 | intergenic | SLC15A4 (p) |  | SLC15A4 (p):i^5^,e^4,5^ |  | ((C)_8_?) | ((C)_7_?) | EU:0.108; AS:0.185 (^11^) | 3.00E-08 | EAS | 1.34 | I ((C)_8_?) | ? | ^11^ |
|  |  |  | rs12370194 | 128792705 | intron | SLC15A4 (p) |  |  | SLC15A4>GLT1D1 | A | T | 0.487 | 3.08E-09 | EAS | 0.82 (0.76-0.88) | T | A | ^27^ |
|  |  |  | **rs1059312** | 128794319 | synonymous | SLC15A4 (p) |  |  |  | A | G | 0.4655 | 1.48E-13 | EU | 1.17 (1.12-1.21) | C | G | ^5^ |
|  |  |  |  |  |  |  |  |  |  |  |  |  | 6.53E-10 | Mix | 1.12 (1.07-1.16) | G | G | ^4^ |
|  |  |  | rs4760589 | 128799837 | intron | SLC15A4 (p) |  |  |  | T | C | 0.483 | 3.17E-09 | EAS | 0.82 (0.76-0.87) | C | T | ^27^ |
|  |  |  | rs11059919 | 128804645 | intron | SLC15A4 (p) |  |  |  | G | A | 0.479 | 8.17E-09 | EU | 1.16 | G | G | ^5^ |
| 251 | 12q24.33 |  | rs11059927 | 128809788 | intron | SLC15A4 (p) |  | SLC15A4 (p):c^4^,e^1,4,34^,d^1^ | SLC15A4>GLT1D1 | T | C | 0.1312 | 2.44E-08 | EU | 1.21 (1.13-1.30) | C | C | ^4^ |
|  |  |  | **rs11059928** | 128811558 | intron | SLC15A4 (p) |  |  |  | A | T | 0.132 | 1.61E-30 | EAS | 0.823±0.017 | A | T | ^14^ |
|  |  |  | rs10847697 | 128814840 | synonymous | SLC15A4 (p) |  |  |  | G | A | 0.131 | 3.54E-11 | EAS | 1.26 (1.17-1.34) | A | A | ^21^ (^29^) |
|  |  |  | rs1385374 | 128816149 | intron | SLC15A4 (p) |  |  |  | C | T | 0.1312 | 1.35E-20 | EAS | 1.29±0.027 | T | T | ^1^ (^21,25,26,29,30^) |
|  |  |  |  |  |  |  |  |  |  |  |  |  | 2.62E-27 | Mix | 1.286±0.023 | T | T | ^1^ |
|  |  |  |  |  |  |  |  |  |  |  |  |  | 3.05E-08 | EU | 1.275±0.044 | T | T | ^1^ (^4^) |
| 252 | 12q24.33 |  | **rs67438707** (rs200521476) | 132463597-132463619 | intergenic | ENSG00000256875 (lncRNA) |  |  |  | (CATCAC)_2_CATCA | (CATCAC)_3_CATCA | 0.3704 | 5.66E-09 | EAS | 0.875±0.023 | G (vs GCATCAC) | (CATCAC)_3_CATCA | ^14^ |
|  |  |  |  |  |  |  |  |  |  |  |  |  |  |  |  |  |  |  |
| 253 | 13q14.11 | rs57668933^(46)^ | rs7329174 | 40983974 | intron | ELF1 (p) |  | ELF1 (p):e,d^1^  WBP4 (p):d^1^  RGCC (C13orf15) (p):d^1^ | ELF1>WBP4>KBTBD6 | A | G | 0.1072 | 1.47E-08 | EAS | 1.26 (1.16-1.36) | G | G | ^150^ |
|  |  |  | **rs57141708** | 41001255 | intron | ELF1 (p) |  |  |  | G | A | 0.108 | 6.84E-22 | EAS | 1.183±0.018 | A | A | ^14^ |
| 254 | 13q14.2 |  | **rs76725306** | 49603317 | intergenic | RCBTB1 (p) |  | SETDB2 (p):e,d^1^  ARL11 (p):e,d^1^ | RCBTB1>PHF11>ARL11 | G | A | 0.191 | 3.60E-08 | Mix | 1.157±0.027 | A | A | ^1^ |
| 255 | 13q32.3 |  | **rs1885889** | 99439046 | intergenic | ENSG00000285448 (lncRNA) |  | TM9SF2 (p):e^1^ | CLYBL>GPR18>TM9SF2 | G | A | 0.233 | 2.05E-13 | Mix | 0.868±0.019 | G | A | ^1^ |
|  |  |  |  |  |  |  |  |  |  |  |  |  | 7.93E-10 | EAS | 0.87±0.023 | G | A | ^1^ |
| 256 | 13q33.3 |  | **rs145720245** | 108839523 | intron | MYO16 (p) |  |  | MYO16 | G | A | 0.002 | 2.8E-10 | EAS | 4 (2.6-6.15) | A | A | ^24^ |
|  |  |  |  |  |  |  |  |  |  |  |  |  |  |  |  |  |  |  |
| 257 | 14q13.2 |  | **rs8016947** | 35363460 | intron | ENSG00000258860 (lncRNA) |  |  | PPP2R3C>FAM177A1>SRP54 | G | T | 0.3872 | 1.08E-13 | EAS | 0.83 | A | G | ^3^ |
| 258 | 14q24.1 |  | **rs4902562** | 68264741 | intron | RAD51B (p) |  | RAD51B (p):e,d^1^ | ZFP36L1>RAD51B>ZFYVE26 | G | A | 0.4056 | 6.15E-10 | EU | 1.14 (1.09-1.19) | A | A | ^5^ |
|  |  |  | rs911263 | 68286876 | intron | RAD51B (p) |  |  |  | T | C | 0.4 | 2.79E-08 | Mix | 0.89 (0.85-0.93) | G | T | ^4^ |
| 259 | 14q31.3 | rs28626750^(46)^ | **rs11845506** | 87916691 | intron | GALC (p) |  |  | GALC>GPR65>KCNK10 | C | A | 0.04273 | 5.00E-10 | MA | 0.20 (0.12-0.33) | A | C | ^4^ |
| 260 | 14q32.32 |  | **rs12148050** | 102797451 | intron | TRAF3 (p) |  | TRAF3 (p):e,d^1^ | TRAF3>AMN>RCOR1 | A | G | 0.490 | 2.57E-08 | Mix | 0.907±0.018 | G | A | ^1^ |
| 261 | 14q32.33 |  | rs2841280 | 104927219 | missense | PLD4 (p) | E/Q | PLD4 (p):e,d^1^, mice^23^ | PLD4>AHNAK2>CDCA4 | G | C | 0.412 | 6.12E-11 | EAS | 1.21±0.03 | C | C | ^1^ |
|  |  |  |  |  |  |  |  |  |  |  |  |  | 5.56E-09 | Mix | 1.125±0.02 | C | C | ^1^ |
|  |  |  | rs4465542 | 104941461 | missense | AHNAK2 (p) | T/A |  | AHNAK2>PLD4>CDCA4 | C | T | 0.4449 | 3.13E-08 | EAS | 0.874±0.026 | G | T | ^30^ |
|  |  |  | rs2582511 | 104949673 | synonymous | AHNAK2 (p) |  |  | PLD4>AHNAK2>CLBA1 | C | T | 0.4165 | 7.9E-11 | EAS | 0.73 (0.66-0.8) | C | T | ^23^ |
|  |  |  | **rs2819426** | 104945922 | missense | AHNAK2 (p) | L/V |  |  | G | C | 0.433 | 2.51E-30 | EAS | 0.824±0.017 | C | G | ^14^ |
|  |  |  |  |  |  |  |  |  |  |  |  |  |  |  |  |  |  |  |
| 262 | 15q14 |  | **rs11073328** | 38472642 | intron | FAM98B (p) |  |  | FAM98B>RASGRP1>FSIP1 | C | T | 0.0967 | 9.90E-15 | EU | 1.935 (2.11-1.771) | T | T | ^43^ |
| 263 | 15q14 | rs11631591^(151)^  *TE* | rs8032939 | 38541832 | intron | RASGRP1 (p) |  | RASGRP1 (p):e↑^1,151^,d^1^  LINC02694 (C15orf53) (lncRNA):e↓,3C^151^ | RASGRP1>FAM98B>SPRED1 | T | C | 0.497 | 3.2E-11 | Mix | 0.88 (0.85-0.92) | T | C | ^151^ |
|  |  |  | rs8035957 | 38546063 | intron | RASGRP1 (p) |  |  |  | T | C | 0.4946 | 5.78E-09 | EAS | 0.892±0.020 | A | C | ^30^ (^1^) |
|  |  |  |  |  |  |  |  |  |  |  |  |  | 3.23E-10 | Mix | 1.123±0.018 | C | C | ^1^ |
|  |  |  | **rs7170151** | 38554477 | intron | RASGRP1 (p) |  |  |  | C | T | 0.477 | 3.20E-12 | EAS | 1.107±0.015 | T | T | ^14^ |
| 264 | 15q14 |  | rs9920715 | 38624705 | intergenic | LINC02694 (lncRNA) |  | RASGRP1 (p):e↑^27,151^,3C^27,151^  LINC02694 (C15orf53) (lncRNA):e,3C^27^ | RASGRP1>FAM98B>SPRED1 | C | T | 0.353 | 5.1E-09 | Mix | 0.89 (0.86-0.93) | T | C | ^151^ |
|  |  |  | **rs12900339** | 38635185 | intron | LINC02694 (lncRNA) |  |  |  | A | G | 0.4367 | 4.73E-10 | EAS | 0.85 0.81-0.89 | G | A | ^27^ |
| 265 | 15q14 |  | **rs12900640** | 38682347 | intron | LINC02694 (lncRNA) |  |  | RASGRP1>FAM98B>SPRED1 | C | A | 0.298 | 2.42E-11 | EAS | 1.101±0.014 | A | A | ^14^ |
| 266 | 15q24.1-15q24.2 |  | rs34933034 | 74787133 | intron | CSK (p) |  | CSK (p):e↑^5,152^  SCAMP2 (p):e,d^1^  ULK3 (p):e,d^1^  PPCDC (p):d^1^ | ULK3>LMAN1L>CSK | G | A | 0.0901 | 1.04E-09 | EU | 1.32 (1.20-1.44) | A | A | ^152^ |
|  |  |  | **rs2289583** | 75018695 | intron | SCAMP5 (p) |  |  | ULK3>LMAN1L>MPI | C | A | 0.1841 | 6.22E-15 | EU | 1.19 (1.14-1.24) | A | A | ^5^ (^11^) |
| 267 | 15q24.1 |  | **rs11553760** | 74798906 | synonymous | CSK (p) |  |  | ULK3>CSK>SCAMP2 | C | T | 0.089 | 7.32E-10 | EAS | 1.111±0.017 | T | T | ^14^ |
| 268 | 15q24.3 |  | **rs869310** | 77537964 | intergenic | LOC101929457 (lncRNA) |  | TBC1D2B (p):e,d^1^  HMG20A (p):e,d^1^ | TSPAN3>PSTPIP1>PEAK1 | T | G | 0.252 | 1.90E-08 | Mix | 0.881±0.023 | G | T | ^1^ |
| 269 | 15q26.2 |  | **rs8023715** | 97064451 | intergenic | LINC02253 (lncRNA) |  |  |  | C | A | 0.074 | 1.20E-08 | EU | 1.812 (2.00-1.638) | A | A | ^43^ |
| 270 | 15q26.3 |  | **rs35985016** | 100988807 | missense | LRRK1 (p) | K/E |  | LRRK1>CHSY1>ALDH1A3 | A | G | 0.010 | 1.95E-08 | EAS | 0.843±0.030 | A | G | ^14^ |
|  |  |  |  |  |  |  |  |  |  |  |  |  |  |  |  |  |  |  |
| 271 | 16p13.13 |  | **rs8054198** | 10944503 | intergenic | CLEC16A (p) |  |  | CLEC16A>RMI2>PRM3 | C | T | 0.069 | 1.79E-08 | MA | 0.36 (0.25-0.51) | T | C | ^4^ |
| 272 | 16p13.13 | *TE* | rs9652601 | 11080508 | intron | CLEC16A (p) |  | CIITA (p):i^5^  SOCS1 (p):i,e^5^  CLEC16A (p):e,d^1^ | CLEC16A>DEXI>RMI2 | G | A | 0.3283 | 7.24E-17 | EU | 1.21 (1.15-1.26) | G | G | ^5^ (^4^) |
|  |  |  | rs2041670 | 11080795 | intron | CLEC16A (p) |  |  |  | G | A | 0.3762 | 2E-16 | EU | 0.84 (0.80-0.88) | T | G | ^4^ |
|  |  |  |  |  |  |  |  |  |  |  |  |  | 2.14E-16 | Mix | 0.85 (0.82-0.89) | T | G | ^4^ |
|  |  |  | rs7200786 | 11083944 | intron | CLEC16A (p) |  |  |  | G | A | 0.4629 | 2.35E-08 | EU | 1.15 | A | A | ^5^ |
|  |  |  | **rs12599402** | 11096031 | intron | CLEC16A (p) |  |  |  | C | T | 0.4996 | 5.55E-11 | EU | 0.86 (0.82-0.90) | C | T | ^4^ (^1^) |
|  |  |  |  |  |  |  |  |  |  |  |  |  | 2.74E-22 | Mix | 0.841±0.018 | C | T | ^1^ |
|  |  |  |  |  |  |  |  |  |  |  |  |  | 1.63E-13 | EAS | 0.843±0.023 | C | T | ^1^ (^153^) |
|  |  |  | rs34361002 | 1096178-11096189 | intron | CLEC16A (p) |  |  |  | (A)_12_ | (A)_14_ | 0.4944 | 1.24E-17 | EAS | 1.14±0.015 | T | (A)_12_ | ^14^ |
| 273 | 16p13.13 |  | **rs35032408** | 11121567 | intron | CLEC16A (p) |  |  | DEXI>RMI2>CLEC16A | T | G | 0.1424 | 2.84E-08 | EAS | 0.69 (0.61-0.79) | G | T | ^27^ |
| 274 | 16p12.2 |  | **rs79401250** | 23860136 | intron | PRKCB (p) |  |  | PRKCB>CHP2>ERN2 | T | G | 0.058 | 1.48E-12 | EAS | 1.172±0.022 | T | T | ^14^ |
|  |  |  | rs16972959 | 23890055 | intron | PRKCB (p) |  |  | PRKCB>PLK1>DCTN5 | G | A | 0.151 | 1.35E-09 | EAS | 0.81 (0.76-0.87) | A | G | ^154^ |
| 275 | 16p11.2 |  | rs7197475 | 30631546 | intergenic | ENSG00000288983 (lncRNA) |  |  | CFAP119>PRR14>ZNF689 | C | T | 0.496 | 2.77E-08 | EAS | 1.31 (1.20-1.46) | A | T | ^21^ |
|  |  |  | **rs534645300** | 30802134-30802147 | intergenic | ZNF629 (p) |  |  | ZNF629>CFAP119>PHKG2 | (T)_14_ | (T)_15_ | 0.232 | 2.68E-09 | EAS | 0.814±0.035 | A (A vs AT) | (T)_15_ | ^14^ |
| 276 | 16p11.2 | rs4889542^(46)^  rs1143679^(155–157)^ | rs34572943 | 31261032 | intron | ITGAM (p) |  | ITGAM (CD11b) (p):i^5^,c^4,5^,e↓^4,5,155^,m^158^,d^1^,f(mutant protein CD11b (rs1143679 A): ↓ binding to fibrinogen, vitronectin, iC3b, DC-SIGN, ICAM-1, ICAM-2; polarization of Mac-1 in the membrane instead of even distribution; ↓phagocytosis; ↓Toll-like receptor 7/8 (TLR7/8)-induced cytokine release)^155–157^  ITGAX (p):e,d^1^ | ITGAM>ITGAX>PYCARD | G | A | 0.0851 | 9.08E-85 | Mix | 1.68 (1.59-1.78) | A | A | ^4^ |
|  |  |  |  |  |  |  |  |  |  |  |  |  | 3.39E-76 | EU | 1.71 (1.61-1.81) | A | A | ^5^ (^4,9^) |
|  |  |  |  |  |  |  |  |  |  |  |  |  | 1.2E-20 | MA | 2.01 (1.73-2.33) | A | A | ^4^ (^35^) |
|  |  |  |  |  |  |  |  |  |  |  |  |  | 5.36E-11 | AA | 1.47 (1.31-1.65) | A | A | ^4^ |
|  |  |  | **rs1143679** | 31265490 | missense | ITGAM (p) | R/H |  |  | G | A | 0.0851 | 2.78E-62 | EU | 1.72 (1.61-1.83) | A | A | ^4^ (^1,5,11,62,64,158–162^) |
|  |  |  |  |  |  |  |  |  |  |  |  |  | 3.60E-90 | Mix | 1.76 | A | A | ^155^ (^158,159,162,163^) |
|  |  |  |  |  |  |  |  |  |  |  |  |  | 3.26E-21 | MA | 2.30 (1.97-2.70) | A | A | ^35^ (^159^) |
|  |  |  |  |  |  |  |  |  |  |  |  |  | 1.62E-13 | AA | 1.54 (1.37-1.73) | A | A | ^4^ (^93,159^) |
|  |  |  | rs35472514 | 31272002 | intron | ITGAM (p) |  |  |  | C | G | 0.121 | 4.24E-53 | EU | 1.7 | G | G | ^5^ |
|  |  |  | rs12928725 | 31272675 | intron | ITGAM (p) |  |  |  | G | T | 0.1378 | 6.61E-22 | MA | 1.94 (1.70-2.23) | T | T | ^4^ |
|  |  |  | rs13338069 | 31288153 | intron | ITGAM (p) |  |  | ITGAX>ITGAM>BCKDK | A | G | 0.1474 | 1.94E-21 | MA | 1.98 (1.72-2.28) | C | G | ^4^ |
|  |  |  | rs7190018 | 31290157 | intron | ITGAM (p) |  |  |  | G | A | 0.1829 | 3.35E-11 | EU | 1.615 | A | A | ^17^ |
|  |  |  | rs9888739 | 31301932 | intron | ITGAM (p) |  |  | ITGAX>ITGAM>STX4 | C | T | 0.2718 | 1.61E-23 | EU | 1.62 (1.47-1.78) | T | T | ^67^ (^16,43,66,69^) |
|  |  |  | rs11860650 | 31315385 | intron | ITGAM (p) |  |  | ITGAX>ITGAM>PYCARD | C | T | 0.1468 | 1.90E-20 | EU | 1.43 (1.32-1.54) | T | T | ^8^ (^17,41^) |
|  |  |  | rs1143678 | 31331684 | missense | ITGAM (p) | P/S |  |  | C | T | 0.1695 | 1.05E-21 | EU | 1.55 | T | T | ^10^ (^67^) |
|  |  |  | rs4548893 | 31353172 | intergenic | ENSG00000289930 (lncRNA) |  |  | ITGAX>FUS>STX4 | C | T | 0.183 | 2.36E-12 | EU | 1.34 (1.24—1.46) | A | T | ^67^ |
|  |  |  | rs11574637 | 31357553 | missense | ITGAX (p) | F/L |  | ITGAX>ITGAM>FUS | T | C | 0.2007 | 3.00E-11 | EU | 1.33 (1.22-1.46) | C | C | ^68^ |
| 277 | 16p11.2 |  | **rs2359661** | 31269826 | intron | ITGAM (p) |  |  | ITGAX>TRIM72>PYDC1 | A | G | 0.4313 | 4.47E-08 | EU | 1.365 | A | A | ^17^ |
| 278 | 16q12.1 |  | **rs11288784** | 50055297-50055309 | intron | HEATR3-AS1 (lncRNA) |  |  | HEATR3>TENT4B>BRD7 | (T)_13_ | (T)_12_ | 0.256 | 2.38E-10 | EAS | 0.902±0.016 | G (G vs GT) | (T)_13_ | ^14^ |
| 279 | 16q12.2 |  | **rs9934578** | 53095122 | intron | CHD9 (p) |  | CHD9 (p):e^2^ | CHD9>RBL2>AKTIP | C | T | 0.239 | 4.86E-08 | Mix | 1.15±0.03 | C | C | ^2^ |
| 280 | 16q13 |  | **rs223881** | 57352654 | intergenic | CCL22 (p) |  | CCL22 (p):d^1^  CCL17 (p):e,d^1^  POLR2C (p):e^76^  COQ9 (p):e^76^ | COQ9>CX3CL1>POLR2C | C | T | 0.452 | 5.87E-16 | Mix | 0.87 (0.84-0.90) | C | T | ^76^ (^1^) |
|  |  |  |  |  |  |  |  |  |  |  |  |  | 6.97E-10 | EAS | 0.869±0.023 | C | T | ^1^ (^27,76^) |
|  |  |  | rs669763 | 57356566 | intergenic | CCL22 (p) |  |  | CX3CL1>DOK4>CCL17 | C | G | 0.441 | 2.89E-15 | EAS | 1.123±0.015 | C | C | ^14^ |
|  |  |  | rs223889 | 57358329 | intergenic | CCL22 (p) |  |  |  | C | T | 0.490 | 1.08E-08 | Mix | 1.21 (1.13-1.29) | T | T | ^4^ |
| 281 | 16q13 |  | **rs2731783** | 58219556 | intergenic | ENSG00000260867 (lncRNA) |  | CSNK2A2 (p):e↓^1,113^ | CCDC113>CSNK2A2>PRSS54 | G | A | 0.214 | 1.08E-09 | Mix | 1.12 | A | A | ^113^ |
| 282 | 16q22.1 |  | rs28410471 | 68520852 | intergenic | ZFP90 (p) |  | ZFP90(FIK) (p): e↓^1,4,11,34^ | ZFP90>SMPD3>SLC7A6 | T | A | 0.168 | 3.14E-08 | EAS | 1.128±0.022 | A | A | ^14^ |
|  |  |  | **rs1749792** | 68535537 | non coding transcript exon | ZFP90 (p) |  |  | ZFP90>SLC7A6>SMPD3 | G | T | 0.2262 | 4.00E-11 | Mix | 1.14 (1.10-1.19) | T | T | ^4^ |
|  |  |  | rs1170426 | 68569895 | intron | ZFP90 (p) |  |  | ZFP90>SMPD3>SLC7A6 | T | C | 0.226 | 2.24E-08 | Mix | 1.12 (1.08-1.17) | C | C | ^11^ |
|  |  |  |  |  |  |  |  |  |  |  |  |  | 4.36E-08 | EAS | 1.20 (1.12-1.28) | C | C | ^11^ |
| 283 | 16q23.2 |  | **rs11376510** | 79711776-79711788 | intron | LINC01229 (lncRNA) |  |  | MAF | (T)_13_ | (T)_14_ | 0.250 | 2.23E-10 | EAS | 0.898±0.017 | (T)_13_ | (T)_14_ | ^14^ |
| 284 | 16q24.1 | rs13335265^(46)^ | **rs13332649** | 85933077 | intron | ENSG00000285163 (lncRNA) |  | IRF8 (p):i^5^,e^1^,d^1^ | IRF8>EMC8>COX4I1 | A | G | 0.11 | 2.12E-18 | EU | 1.34 | A | A | ^5^ (^11^) |
|  |  |  | rs11648084 | 85938992 | intron | ENSG00000285163 (lncRNA) |  |  |  | C | T | 0.3478 | 2.34E-09 | EU | 0.83 (0.77-0.89) | A | C | ^75^ |
|  |  |  | rs11644034 | 85939006 | intron | ENSG00000285163 (lncRNA) |  |  |  | G | A | 0.1198 | 9.58E-18 | EU | 1.25 (1.19-1.32) | G | G | ^5^ (^1,9,75^) |
|  |  |  |  |  |  |  |  |  |  |  |  |  | 5.30E-17 | Mix | 0.777±0.03 | A | G | ^1^ (^75^) |
|  |  |  | rs4843869 | 85941535 | intron | ENSG00000285163 (lncRNA) |  |  |  | G | A | 0.1052 | 7.61E-10 | EU | 0.76 (0.70-0.83) | A | G | ^75^ |
| 285 | 16q24.1 |  | **rs447632** | 85934034 | intron | ENSG00000285163 (lncRNA) |  | IRF8 (p):e^1^,d^1^ | IRF8>C16orf74>EMC8 | A | G | 0.470 | 7.23E-28 | EAS | 0.851±0.015 | A | G | ^14^ |
|  |  |  | rs2934498 | 85934676 | intron | ENSG00000285163 (lncRNA) |  |  |  | A | G | 0.3786 | 2.56E-11 | Mix | 1.155±0.022 | G | G | ^1^ |
|  |  |  |  |  |  |  |  |  |  |  |  |  | 4.97E-09 | EAS | 1.25 (1.16-1.34) | G | G | ^27^ |
| 286 | 16q24.1 |  | rs34912238 | 85968297 | intron | ENSG00000285040 (lncRNA) |  | IRF8 (p):↑e^1,164^,d^1^ | IRF8>C16orf74>EMC8 | C | T | 0.0803 | 6.95E-11 | EAS | 0.65 (0.57-0.74) | T | C | ^4^ |
|  |  |  | rs11117431 | 85981710 | intron | ENSG00000285040 (lncRNA) |  |  | IRF8>EMC8>COX4I1 | A | G | 0.1312 | 6.25E-11 | EU | 0.82 (0.78-0.87) | G | A | ^23^ |
|  |  |  |  |  |  |  |  |  |  |  |  |  | 1.30E-08 | EAS | 0.62 (0.52-0.73) | G | A | ^27^ |
|  |  |  | rs2280381 | 85985027 | intron | ENSG00000285040 (lncRNA) |  |  |  | T | C | 0.2684 | 3.94E-19 | Mix | 1.231±0.023 | T | T | ^1^ |
|  |  |  |  |  |  |  |  |  |  |  |  |  | 6.75E-15 | EAS | 1.323±0.036 | T | T | ^1^ |
|  |  |  |  |  |  |  |  |  |  |  |  |  | 1.24E-08 | EU | 1.16 | A | T | ^42^ |
|  |  |  | **rs11117432** | 85985665 | intergenic | ENSG00000285040 (lncRNA) |  |  |  | G | A | 0.087 | 1.27E-32 | EAS | 0.728±0.027 | A | G | ^14^ |
|  |  |  | rs11117433 | 85985910 | intergenic | ENSG00000285040 (lncRNA) |  |  |  | G | C | 0.104 | 1.84E-11 | Mix | 0.85 (0.80-0.90) | C | G | ^4^ |
|  |  |  |  |  |  |  |  |  |  |  |  |  | 3.19E-10 | EU | 0.84 (0.80-0.89) | C | G | ^4^ |
| 287 | 16q24.2 | **rs933717**^(165)^ | **rs933717** | 87381644 | intron | FBXO31 (p) |  | MAP1LC3B (p):e↑^165^ | FBXO31>MAP1LC3B>ZCCHC14 | C | T | 0.405 | 2.36E-10 | EAS | 0.13 (0.07–0.24) | T | C | ^165^ |
|  |  |  |  |  |  |  |  |  |  |  |  |  |  |  |  |  |  |  |
| 288 | 17p13.2 |  | **rs2286672** | 4809322 | missense | PLD2 (p) | R/C | PLD2 (p):i,c,e^5^ | PLD2>MINK1>PSMB6 | C | T | 0.1923 | 2.93E-09 | EU | 1.25 (1.16-1.35) | T | T | ^5^ |
| 289 | 17p13.1 |  | rs9899849 | 7331664 | intron | ENSG00000224647 (pseudogene) |  | ACAP1 (p):e,d^1^ | ACAP1>KCTD11>CLDN7 | G | A | 0.294 | 1.10E-08 | EU | 1.209±0.033 | A | A | ^1^ |
|  |  |  | **rs61759532** | 7337072 | intron | ACAP1 (p) |  |  | ACAP1>CLDN7>KCTD11 | C | T | 0.099 | 2.79E-11 | EAS | 1.235±0.032 | T | T | ^14^ |
| 290 | 17p11.2 |  | **rs35966917** | 16936587 | intron | TNFRSF13B (p) |  | TNFRSF13B (p):d^1^ | COPS3>NT5M>TNFRSF13B | A | G | 0.405 | 4.66E-09 | EAS | 0.914±0.015 | A | G | ^14^ |
|  |  |  | rs34562254 | 16939677 | missense | TNFRSF13B (p) | P/L |  | TNFRSF13B>COPS3>NT5M | G | A | 0.191 | 2.88E-08 | EAS | 1.179±0.029 | A | A | ^1^ |
|  |  |  | rs4792801 | 16942153 | intron | TNFRSF13B (p) |  |  | TNFRSF13B>MPRIP>CCDC144A | C | T | 0.4922 | 1.41E-08 | EAS | 0.894±0.020 | A | C | ^30^ |
| 291 | 17q12 | rs112569955^(46)^ | **rs4252665** | 39729130 | 3’ UTR | ERBB2 (p) |  | IKZF3 (p):i^5^,e^1^,d^1^  GSDMB (p): e,d^1^  ORMDL3 (p): e,d^1^ | GSDMB>IKZF3>GSDMA | C | T | 0.0160 | 1.96E-12 | Mix | 1.46 (1.26-1.70) | A | T | ^4^ |
|  |  |  |  |  |  |  |  |  |  |  |  |  | 6.23E-11 | EU | 1.43 (1.28-1.59) | A | T | ^4^ |
|  |  |  | rs2941509 | 39764941 | 3’ UTR | IKZF3 (p) |  |  |  | C | T | 0.0869 | 7.98E-09 | EU | 1.35 (1.22 -1.49) | A | T | ^5^ |
|  |  |  | rs9913957 | 39818246 | intron | IKZF3 (p) |  |  |  | A | G | 0.0519 | 1.39E-08 | Mix | - | G | G | ^75^ |
|  |  |  | rs8076347 | 39821288 | intron | IKZF3 (p) |  |  |  | G | T | 0.0903 | 3.01E-08 | Mix | - | A | T | ^75^ |
|  |  |  | rs143123127 | 39850937 | intron | IKZF3 (p) |  |  | IKZF3>GSDMB>GSDMBA | G | A | 0.0499 | 5.68E-09 | EU | 1.51 | A | A | ^5^ |
|  |  |  | rs8079075 | 39854562 | intron | IKZF3 (p) |  |  | GSDMB>IKZF3>GSDMA | A | G | 0.0525 | 3.02E-09 | EU | 1.41 (1.26-1.57) | C | G | ^4^ (^1^) |
|  |  |  |  |  |  |  |  |  |  |  |  |  | 4.83E-09 | Mix | - | G | G | ^75^ |
|  |  |  | rs1453560 | 39867188 | intergenic | ZPBP2 (p) |  |  |  | T | G | 0.091 | 3.48E-10 | Mix | - | C | G | ^75^ |
|  |  |  |  |  |  |  |  |  |  |  |  |  | 5.14E-09 | EU | 1.40 (1.25-1.56) | C | G | ^4^ |
| 292 | 17q21.31 |  | **rs12952708 (**rs114038709) | 45379362 | intergenic | ENSG00000267446 (lncRNA) |  | ARHGAP27 (p):e^9^ | ARHGAP27>SPATA32>FMNL1 | C | T | 0.385 | 3.70E-08 | EU | 1.16 (1.11-1.22) | T | T | ^9^ |
| 293 | 17q21.33 |  | **rs2671655** | 49390658 | intron | ENSG00000262039 |  |  | PHOSPHO1>ZNF652>GNGT2 | T | C | 0.119 | 4.60E-08 | EAS | 1.087±0.015 | T | T | ^14^ |
| 294 | 17q25.1 |  | **rs8072449** | 75316103 | intergenic | GRB2 (p) |  | GRB2 (p): e^4^ | GRB2>MYO15B>MIF4GD | A | G | 0.398 | 1.19E-11 | Mix | 0.84 (0.80-0.89) | G | A | ^4^ |
|  |  |  | rs36023980 | 75345203 | intron | GRB2; ENSG00000265987 (lncRNA) |  |  |  | C | T | 0.403 | 4.7E-09 | EU | 1.18 (1.13-1.24) | C | C | ^9^ |
| 295 | 17q25.3 |  | **rs113417153** | 78377098 | intergenic | PGS1 (p) |  |  | PGS1>SOCS3>TMEM235 | C | T | 0.118 | 1.90E-08 | EAS | 0.893±0.020 | T | C | ^14^ |
|  |  |  |  |  |  |  |  |  |  |  |  |  |  |  |  |  |  |  |
| 296 | 18q22.2 |  | **rs763361** | 69864406 | missense | CD226 (p) | S/C | CD226 (p):e^1,27^,d^1^,3D^27^  ACRBP (p): e^27^  MAP3K7CL (p): e^27^ | CD226>DOK6>TMX3 | T | C | 0.4694 | 3.03E-12 | Mix | 0.883±0.018 | T | C | ^1^ (^113^) |
|  |  |  | rs1610555 | 69875911 | intron | CD226 (p) |  |  | CD226>DOK6>RTTN | G | T | 0.4718 | 4.50E-11 | EAS | 1.19 (1.13-1.26) | T | T | ^27^ |
|  |  |  | rs1788097 | 69876452 | intron | CD226 (p) |  |  |  | T | C | 0.448 | 6.28E-10 | EAS | 1.096±0.015 | T | C | ^14^ |
| 297 | 18q23 |  | **rs118075465** | 79626912 | intergenic | CTDP1-DT (lncRNA) |  |  | NFATC1>CTDP1>KCNG2 | G | A | 0.108 | 1.16E-10 | EAS | 1.140±0.020 | A | A | ^14^ |
|  |  |  |  |  |  |  |  |  |  |  |  |  |  |  |  |  |  |  |
| 298 | 19p13.3 |  | rs10414086 | 937136 | intron | ARID3A (p) |  |  | KISS1R>CFD>MED16 | C | T | 0.188 | 2.75E-14 | EAS | 0.885±0.016 | T | C | ^14^ |
|  |  |  | **rs2238577** | 948532 | intron | ARID3A (p) |  |  | CFD>ARID3A>KISS1R | C | T | 0.304 | 1.83E-14 | EAS | 0.885±0.016 | T | C | ^14^ |
| 299 | 19p13.3 |  | **rs4807205** | 2167879 | intron | DOT1L (p) |  | DOT1L (p):e↑^84^ | AP3D1>DOT1L>PEAK3 | A | G | 0.447 | 8.17E-09 | Mix | 1.12±0.019 | G | G | ^84^ |
| 300 | 19p13.3 |  | rs11085192 | 6689054 | intron | C3 (p) |  |  | TNFSF14>C3>TRIP10 | T | C | 0.316 | 1.18E-10 | EAS | 0.84±0.027 | T | C | ^14^ |
|  |  |  | **rs5826945** | 6697077 | intron | C3 (p) |  |  | C3>TRIP10>GPR108 | A | T | 0.130 | 9.67E-11 | EAS | 0.836±0.028 | A | T | ^14^ |
| 301 | 19p13.2 |  | **rs3093030** | 10286727 | non coding transcript exon | ICAM4-AS1 (lncRNA) |  | ICAM1 (p): soluble form in plasma↑^159^ | ICAM1>ICAM5>TYK2 | C | T | 0.3199 | 4.88E-08 | Mix | 1.16 (1.10-1.22) | A | T | ^159^ |
| 302 | 19p13.2 |  | **rs74908652** | 10312296 | intron | ENSG00000167807 (p); FDX2 (p) |  |  | ICAM1>ICAM4>TYK2 | T | C | 0.0901 | 2.28E-09 | EU | 0.83 (0.78-0.88) | G | T | ^4^ |
| 303 | 19p13.2 | *TE* | rs11085725 | 10351837 | intron | TYK2 (p) |  | TYK2 (p):i^5^,c^5^,e^1,5,6,34^,3D^6^,d^1^ | TYK2>ICAM3>ICAM5 | C | T | 0.2861 | 9.60E-13 | EU | 1.27 | C | C | ^11^ |
|  |  |  |  |  |  |  |  |  |  |  |  |  | 9.17E-10 | Mix | 1.18 (1.12-1.25) | C | C | ^11^ |
|  |  |  | rs11085727 | 10355447 | intron | TYK2 (p) |  |  |  | C | T | 0.2879 | 1.20E-13 | EU | 1.24 | C | C | ^5^ |
|  |  |  | rs280519 | 10362257 | splice region | TYK2 (p) |  |  | ICAM1>ICAM3>TYK2 | G | A | 0.4669 | 2.14E-12 | EU | 1.17 (1.12-1.22) | T | A | ^4^ (^42^) |
|  |  |  | rs2304256 | 10364976 | missense | TYK2 (p) | V/F |  | TYK2>ICAM3>ICAM1 | C | A | 0.2658 | 3.5E-13 | EU | 1.24 (1.17-1.31) | C | C | ^5^ (^1,9,11,166,167^) |
|  |  |  | **rs34725611** | 10366391 | intron | TYK2 (p) |  |  |  | A | G | 0.2694 | 4.93E-23 | EU | 0.78 (0.74-0.82) | G | A | ^4^ |
| 304 | 19p13.2 |  | **rs34536443** | 10352442 | missense | TYK2 (p) | P/A |  | TYK2>ICAM5>ICAM1 | G | C | 0.0102 | 2.43E-25 | Mix | 0.47 (0.38-0.57) | G | G | ^4^ |
|  |  |  |  |  |  |  |  |  |  |  |  |  | 1.24E-21 | EU | 0.52 (0.45-0.59) | G | G | ^4^ |
| 305 | 19p13.2 |  | **rs55882956** | 10359243 | missense | TYK2 (p) | R/W |  | TYK2>ICAM4>MRPL4 | G | A | 0.008 | 1.23E-16 | EAS | 0.674±0.048 | A | G | ^14^ |
| 306 | 19p13.11 |  | **rs2362475** | 16329024 | intergenic | KLF2 (p) |  | KLF2 (p):e↓^84^ | KLF2>CALR3>C19orf44 | C | A | 0.279 | 2.00E-09 | EAS | 0.85±0.028 | A | C | ^84^ |
| 307 | 19p13.11 |  | **rs2384991** | 18275824 | intergenic | IQCN (p) |  | IQCN (KIAA1683) (p):e^1^ | IQCN>JUND>MAST3 | A | C | 0.305 | 4.95E-08 | Mix | 1.108±0.019 | C | C | ^1^ |
| 308 | 19p13.11 |  | rs13344313 | 18406957 | intergenic | LRRC25 (p) |  | LRRC25 (p):e,d^1^ | ELL>SSBP4>LRRC25 | G | A | 0.277 | 1.34E-10 | Mix | 0.861±0.023 | A | G | ^1^ (^4^) |
|  |  |  | **rs11673604** | 18430178 | intron | SSBP4 (p) |  |  | ELL>SSBP4>CRLF1 | T | C | 0.477 | 4.21E-12 | EAS | 1.144±0.019 | T | T | ^14^ |
| 309 | 19p13.11 |  | **rs12461589** | 32581862 | intron | PDCD5 (p) |  | ANKRD27 (p):e^1^ | RGS9BP>ANKRD27>DPY19L3 | C | T | 0.062 | 5.00E-10 | EAS | 0.898±0.017 | T | C | ^14^ |
|  |  |  | rs405858 | 32615715 | synonymous | ANKRD27 (p) |  |  | PDCD5>ANKRD27>RGS9BP | C | T | 0.481 | 2.38E-09 | Mix | 0.894±0.019 | T | C | ^1^ |
|  |  |  |  |  |  |  |  |  |  |  |  |  | 1.70E-08 | EAS | 0.872±0.024 | T | C | ^1^ |
| 310 | 19p13.33 |  | rs3760667 | 49311575 | intron | SLC6A16 (p) |  | CD37 (p):e,d^1^  FLT3LG (p):d^1^  IRF3 (p):e,d^1^  RRAS (p):e,d^1^  FCGRT (p):e,d^1^  ALDH16A1 (p):e,d^1^ | DKKL1>KASH5>SLC6A16 | C | T | 0.300 | 2.31E-09 | Mix | 0.87 | T | C | ^1^ |
|  |  |  | rs8108669 | 49342638 | intron | TEAD2 (p); CD37 (p) |  |  | DKKL1>KASH5>TEAD2 | C | T | 0.323 | 4.68E-12 | EAS | 0.893±0.016 | T | C | ^14^ |
|  |  |  | **rs33974425** | 49348490-49348500 | intron | TEAD2 (p) |  |  |  | CAGCTGCATCA | CA | 0.323 | 4.40E-12 | EAS | 1.120±0.016 | CCAGCTGCAT | CAGCTGCATCA | ^14^ |
| 311 | 19p13.33 |  | **rs7251** | 49659652 | missense | IRF3(p) | S/T | IRF3 (p):e↑^168^ | IRF3>NOSIP>PRRG2 | C | G | 0.485 | 4.40E-08 | Mix | 0.876 | C | G | ^168^ |
| 312 | 19q13.41 |  | rs2305772 | 51530488 | missense | SIGLEC6 (p) | P/S | SIGLEC6 (p):e^1,27^,d^1^ | SIGLEC12>SIGLEC6>SIGLEC14 | A | G | 0.4195 | 2.27E-08 | EAS | 0.86 (0.81-0.9) | A | G | ^27^ |
|  |  |  | **rs3794986** | 51544061 | intergenic | SIGLEC6 (p) |  |  | SIGLEC12>SIGLEC6>ZNF175 | T | G | 0.442 | 1.46E-14 | EAS | 0.89±0.015 | T | G | ^14^ |
| 313 | 19q13.41 |  | **rs4801882** | 51623800 | intron | SIGLEC5 (p) |  |  | SIGLEC14>ZNF175>CEACAM18 | G | A | 0.373 | 1.86E-18 | EAS | 0.882±0.014 | A | G | ^14^ |
| 314 | 19q13.42 |  | **rs56154925** | 55226430 | intergenic | TMEM86B (p) |  | PPP6R1 (p):e,d^1^  TMEM86B (p):e,d^1^ | PPP6R1>TMEM86B>DNAAF3 | C | T | 0.169 | 4.93E-23 | Mix | 0.88 (0.84-0.92) | T | C | ^4^ |
|  |  |  | rs10419308 | 55228445 | splice region | TMEM86B (p) |  |  | TMEM86B>PPP6R1>HSPBP1 | G | A | 0.166 | 3.60E-08 | Mix | 0.843±0.031 | A | G | ^1^ |
|  |  |  |  |  |  |  |  |  |  |  |  |  |  |  |  |  |  |  |
| 315 | 20p13 |  | **rs6074813** | 1561106 | intron | ENSG00000260861 (p) |  | SIRPB2 (p):d^1^  SIRPG (p):e,d^1^  SIRPB1 (p):d^1^  SIRPD (p):e,d^1^ | SIRPB1>SIRPD>NSFL1C | T | G | 0.433 | 3.23E-08 | Mix | 1.12±0.021 | T | T | ^1^ |
| 316 | 20q13.12 |  | **rs4810485** | 46119308 | intron | CD40 (p) |  | CD40 (p):e↑^1,4,169^,d^1^  NCOA5 (p):e^4^,^1^,d^1^ | CD40>SLC12A5>PLTP | G | T | 0.2384 | 9.95E-09 | Mix | 1.43 (1.17-1.76) | A^#^ | ?A | ^4^ |
|  |  |  |  |  |  |  |  |  |  |  |  |  | 2.0E-08 | EU | 0.63 (0.53-0.74) | T^#^ | ?G | ^169^ |
| 317 | 20q13.13 | rs56309244^(46)^  rs117447227^(46)^  rs1884738^(46)^ | **rs11697848** | 49958778 | intergenic | RNF114 (p) |  |  | RNF114>SPATA2>SLC9A8 | C | T | 0.039 | 1.40E-11 | EU | 2.115 (2.37-1.887) | T | T | ^43^ |
|  |  |  |  |  |  |  |  |  |  |  |  |  |  |  |  |  |  |  |
| 318 | 22q11.21 |  | **rs4819670** | 18166589 | intron | USP18 (p) |  |  | USP18>TUBA8>PEX26 | C | T | 0.363 | 5.53E-11 | EAS | 1.151±0.022 | T | T | ^14^ |
| 319 | 22q11.21 | *TEx2* | rs463426 | 21454896 | intergenic | HIC2 (p) |  | UBE2L3 (p):e↑^1,5,34,170,171^,c^4^ | UBE2L3>CCDC116>HIC2 | T | C | 0.500 | 8.36E-22 | EAS | 1.30 (1.23-1.37) | A | T | ^25^ (^1,21^) |
|  |  |  |  |  |  |  |  |  |  |  |  |  | 6.57E-15 | Mix | 0.868±0.018 | C | T | ^1^ |
|  |  |  | rs131654 | 21562901 | intron | UBE2L3 (p) |  |  | UBE2L3>RIMBP3C>YDJC | T | G | 0.2416 | 2.29E-16 | EAS | 0.78 (0.74-0.83) | C | T | ^21^ (^30^) |
|  |  |  | rs131658 | 21563337 | intron | UBE2L3 (p) |  |  | UBE2L3>CCDC116>YDJC | C | G | 0.3992 | 1.03E-16 | EU | 1.25 (1.19-1.32) | G | G | ^4^ |
|  |  |  | rs140490 | 21567397 | intron | UBE2L3 (p) |  |  | UBE2L3>RIMBP3C>CCDC116 | C | T | 0.414 | 8.6E-14 | ND | 1⋅30 (1.21-1.39). | T | T | ^170^ |
|  |  |  | rs181359 | 21574352 | intron | UBE2L3 (p) |  |  | UBE2L3>CCDC116>YDJC | G | A | 0.3059 | 1.15E-09 | EU | 1.23 (1.15-1.33) | T | A | ^36^ |
|  |  |  | rs5754217 | 21585386 | intron | UBE2L3 (p) |  |  |  | G | T | 0.4141 | 7.3E-09 | EU | - | T | T | ^16^ |
|  |  |  | rs5998619 | 21591562 | intron | UBE2L3 (p) |  |  |  | G | A | 0.2929 | 1.02E-14 | EU | 1.43 | A | A | ^10^ |
|  |  |  | rs11089629 | 21604583 | intron | UBE2L3 (p) |  |  |  | T | G | 0.4217 | 1.11E-16 | EU | 1.25 (1.18-1.31) | G | G | ^4^ |
|  |  |  | rs5754344 | 21609497 | intron | UBE2L3 (p) |  |  |  | A | G | 0.226 | 1.81E-45 | EAS | 0.805±0.015 | A | G | ^14^ |
|  |  |  | rs5998672 | 21612153 | intron | UBE2L3 (p) |  |  |  | G | A | 0.4133 | 1.2E-09 | EAS | 1.3 (1.20-1.42) | A | A | ^23^ |
|  |  |  | **rs4821116** | 21619030 | intron | UBE2L3 (p) |  |  |  | C | T | 0.225 | 8.86E-46 | EAS | 1.24±0.015 | T | T | ^14^ |
|  |  |  | rs7444 | 21622645 | 3’ UTR | UBE2L3 (p) |  |  |  | T | C | 0.4291 | 1.84E-22 | EU | 1.27 (1.21 - 1.33) | C | C | ^5^ (^4,9,11^) |
|  |  |  |  |  |  |  |  |  |  |  |  |  | 8.56E-18 | EAS | 1.215±0.023 | C | C | ^1^ |
|  |  |  |  |  |  |  |  |  |  |  |  |  | 8.99E-22 | Mix | 1.22±0.021 | C | C | ^1^ (^4^) |
|  |  |  | rs4821124 | 21625000 | intergenic | UBE2L3 (p) |  |  |  | T | C | 0.238 | 7.98E-10 | EAS | 1.22 (1.14-1.32) | C | C | ^27^ |
|  |  |  | rs2298428 | 21628603 | missense | YDJC (p) | A/T |  | YDJC>UBE2L3>CCDC116 | C | T | 0.2248 | 1.94E-12 | EU | 1.28 | - | T | ^11^ |
|  |  |  |  |  |  |  |  |  |  |  |  |  | 1.31E-11 | EAS | 1.23 | T | T | ^171^ (^11^) |
|  |  |  | rs3747093 | 21630090 | intergenic | YDJC (p) |  |  | UBE2L3>CCDC116>YDJC | G | A | 0.422 | 2.67E-13 | EU | 1.26 | A | A | ^5^ |
| 320 | 22q13.1 | rs2069235^(46)^ | rs9611155 | 39343182 | intergenic | SYNGR1 (p) |  | PDGFB (p):e,d^1^  SYNGR1 (p):e^27^ | SYNGR1>MGAT3>PDGFB | T | C | 0.446 | 7.66E-09 | EAS | 1.137±0.022 | T | T | ^14^ |
|  |  |  | **rs61616683** | 39359768 | intron | SYNGR1 (p) |  |  | SYNGR1>PDGFB>MGAT3 | C | T | 0.3355 | 5.73E-10 | EAS | 0.79 (0.73-0.85) | C | T | ^27^ |
| 321 | 22q13.1 |  | **rs137956** | 39897459 | intergenic | GRAP2 (p) |  |  | GRAP2>ENTHD1>CACNA1I | T | C | 0.276 | 5.00E-08 | Mix | 0.88 (0.84-0.92) | G | T | ^4^ |
|  |  |  |  |  |  |  |  |  |  |  |  |  |  |  |  |  |  |  |
| 322 | Xp22.2 |  | rs7062536 | 12821033 | intron | PRPS2 (p) |  |  | PRPS2>TLR7>TLR8 | G | A | 0.2887 | 1E-08 | EAS | 0.84 (0.80-0.89) | A | G | ^172^ |
|  |  |  | **rs6641111** | 12821671 | intron | PRPS2 (p) |  |  |  | C | G | 0.247 | 3.27E-25 | EAS | 1.191±0.017 | C | C | ^14^ |
| 323 | Xp22.2 | **rs3853839**^(173)^ | **rs3853839** | 12889539 | 3’ UTR | TLR7 (p) |  | TLR7 (p):e↑^173,174^ | TLR7>TLR8>PRPS2 | C | G | 0.4024 | 2E-19 | Mix | 1.25 (1.20-1.32) | G | G | ^173^ |
|  |  |  |  |  |  |  |  |  |  |  |  |  | 6.5E-10 | EAS | 1.27 (1.17-1.36) | G | G | ^174^ (^173,175,176^) |
|  |  |  | rs4830478 | 12900169 | intergenic | TLR8-AS1 (lncRNA) |  |  | TLR8>TLR7>PRPS2 | G | A | 0.418 | 1.91E-09 | EAS | 1.136±0.021 | A | A | ^14^ |
| 324 | Xp21.2 |  | **rs887369** | 30559729 | synonymous | CXorf21 (p) |  |  | TASL>GK>NR0B1 | C | A | 0.1028 | 5.26E-10 | EU | 1.15 (1.10-1.21) | C | C | ^5^ |
| 325 | Xp11.22 |  | **rs13440883** | 53072295 | intron | GPR173 (p) |  |  | GPR173>TSPYL2>KANTR | A | C | 0.3327 | 7.53E-09 | Mix | 1.16 (1.11-1.23) | - | C | ^177^ |
| 326 | Xp11.21 | rs5913948^(46)^  rs5914728^(46)^  rs5960754^(46)^  rs11797562^(46)^  rs5960771^(46)^  rs1489965^(46)^  rs944916^(46)^  rs2148947^(46)^ | **rs5914778** | 56731798 | intron | NBDY (p) |  |  | NBDY>UBQLN2>SPIN3 | G | A | 0.3004 | 5.26E-12 | EAS | 1.35 | A | A | ^178^ |
|  |  |  | rs5914012 | 56882269 | intron | SPIN3 (p) |  |  | SPIN3>NBDY>SPIN2B | C | T | 0.324 | 6.65E-09 | EAS | 1.104±0.017 | T | T | ^14^ |
| 327 | Xq28 |  | **rs143181706** | 150504983 | intron | MAMLD1 (p) |  |  | MAMLD1>MTM1>MTMR1 | C | T | 0.1099 | 3.7E-08 | EAS | 1.5 (1.3-1.74) | T | T | ^23^ |
| 328 | Xq28 |  | **rs5987175** ^[20]^ | 153888385 | intron | ENSG00000284987 (p), L1CAM-AS1 (lncRNA) |  |  | SSR4>L1CAM>IRAK1 | T | C | 0.3338 | 1.21E-09 | Mix | 0.85 | - | T | ^177^ |
| 329 | Xq28 | rs3027878^(46)^  rs80208125^(46)^  rs6643807^(46)^  rs6643653^(46)^  rs1059702^(46)^  rs1059703^(179)^ | rs2269368 | 153924366 | intron | ARHGAP4 (p) |  | NAA10 (p):e^172^  MEPC2 (p):e↓^180^  IRAK1 (p):i^5^,f(risk rs1059703-G/Ser: NF-κB activation↑, phosphorylation of IRAK-1↑, interaction with TRAF6↑ and more)^179^ | HCFC1>TMEM187>PLXNB3 | C | T | 0.4932 | 1.4E-11 | EU | - | T | T | ^16^ |
|  |  |  | rs2071128 ^[20]^ | 153929940 | 3’ UTR | NAA10 (p) |  |  | PLXNB3>NAA10>ARHGAP4 | G | A | 0.346 | 2.19E-13 | EAS | 0.81 (0.77-0.86) | G | A | ^172^ |
|  |  |  | X:153214462:I (b37)*** | 153949011 | 3’ UTR | HCFC1 (p) |  |  |  | - | I | 0.157(^5^) | 1.33E-15 | EU | 1.62 | I | ? | ^5^ |
|  |  |  | rs17422 ^[20]^ | 153961975 | intron | HCFC1 (p) |  |  | HCFC1>TMEM187>PLXNB3 | G | A | 0.4151 | 1.47E-15 | EAS | 0.75 (0.71-0.80) | T | G | ^172^ |
|  |  |  |  |  |  |  |  |  |  |  |  |  | 1.66E-14 | Mix | 1.26 | - | G | ^177^ |
|  |  |  | rs5986948 | 154000721 | intergenic | IRAK1 (p) |  |  | IRAK1>RENBP>NAA10 | C | T | 0.400 | 4.36E-10 | EAS | 0.64 (0.56-0.74) | C | T | ^32^ |
|  |  |  | rs763737 | 154012856 | intron | IRAK1 (p) |  |  | IRAK1>RENBP>HCFC1 | G | A | 0.4837 | 5.04E-10 | Mix | - | G | G | ^181^ |
|  |  |  |  |  |  |  |  |  |  |  |  |  | 2.29E-08 | EAS | 1.68 | G | G | ^181^ |
|  |  |  | **rs1059702** ^[20]^ | 154018741 | missense | IRAK1 (p) | F/S |  |  | G | A | 0.3711 | 1.30E-27 | Mix | 1.43 | A | A | ^180^ (^177^) |
|  |  |  |  |  |  |  |  |  |  |  |  |  | 5.89E-54 | EAS | 1.356±0.02 | A | A | ^14^ (^27,172,180^) |
|  |  |  |  |  |  |  |  |  |  |  |  |  | 8.42E-14 | MA | 0.58 (0.49-0.68) | G | A | ^35^ (^180^) |
|  |  |  |  |  |  |  |  |  |  |  |  |  | 1.2E-09 | EU | 1.35 (1.22-1.48) | A | A | ^180^ |
|  |  |  | rs2734647 | 154026729 | 3’ UTR | MECP2 (p) |  |  | IRAK1>HCFC1>TMEM187 | C | T | 0.3738 | 5.22E-18 | EAS | 0.72 (0.67-0.76) | C | T | ^172^ (^32^) |
|  |  |  | rs17435 | 154046529 | intron | MECP2 (p) |  |  | HCFC1>TMEM187>MECP2 | T | A | 0.446 | 1.2E-08 | Mix | 1.39 (1.24-1.56) | T | T | ^182^ |
|  |  |  | rs1734787 | 154059995 | non coding transcript exon | MECP2 (p) |  |  | MECP2>IRAK1>HCFC1 | A | C | 0.3913 | 1.78E-15 | EU | 1.31 (1.22-1.40) | C | C | ^5^ (^183^) |
| 330 | Xq26 |  | **rs5945199** | 154521643 | intron | G6PD (p) |  |  | FAM3A>IKBKG>G6PD | G | A | 0.208 | 2.90E-12 | EAS | 0.778±0.036 | A | G | ^14^ |

**Comments:**

Table headers: N – locus number in the table; Location – chromosome and chromosome region; Causal variant (ref); presence of TE – candidate for causal variant with confirmed functional properties in experiments (references are given in the brakets), presence of transposable elements (TEs) in LD with SLE-associated locus with evidence of being functional/causal variants; Variant – SLE associated variant reported in literature; Position GRCh38.p13 – genomic coordinates; Gene by position (Closest gene) – gene intersected by variant or nearest gene for intergenic variants ; Aminoacid change – Aminoacid change for missense varaints; Candidate gene for loci (ref) – with information about candidate gene reported in literature; Major allele; Minor allele; Global MAF – minor allel frequency from 1000 genomes or other sourses if data from 1000 genomes (sourse <https://useast.ensembl.org/>); Lowest p–value for variant reported in literature; Population – population used in the study, every population where variant riched GWAS signifance is listed; OR – odd rartio repoted in literature that corresponds to variant with lowest P–value; OR Allele – allele in literature for wich OR was reported (if OR>1, then it is SLE risk allele, if OR<1, then it is SLE protective allele); Risk allele – SLE risk allele, all alleles were unified and correspond the current GRCh38.p13 build and DNA strand; References, best p value (others) – references,the first reference for the variant with the best/lowest p value and in brakets other publications that reported this variant that reached GWAS signifance.

Colunm “Candidate gene for loci (reference)”: e – candidate gene based on association with gene expression association including eQTL and single gene and protein expression analysis; 3D – candidate gene based on a physical interaction between genomic loci contaningt SLE risk variants and gene promoter/transciption start sites observed in chromosome conformation capture (Hi–C), Promoter Capture HiC and Chromatin interaction analysis by paired–end tag sequencing (ChIA–PET); m – computational modelling for protein structure; i – The immune phenotype designation from The Phenotypes/Alleles project in MGI http://www.informatics.jax.org/phenotypes.shtml of genes within +/−200kb of associated SNP; mice – gene mutant mice experiments ; c – coding variant with ponential advers effect; f – functional tests for protein structure altered by SLE risk variant; d – gene identified by DEPICT (^184^); (p) – protein coding gene; (lncRNA) – Long noncoding RNA; (miRNA) – MicroRNA

Colunm “Population”: Mix – mixed populations containing individuals of different ancestries reported as transancestral analysis, mixed populations; EUR – European reported as European, European American, Finnish, Swedish, Italian, Greek, Turkey, Italy; Spanish from Europe; EAS – East Asian population reported as Asian, Chinese and Han Chinese, Korean, Japanese, East Asian; AA – African American reported as Americans of African Ancestry, African-American, Gullah African–American; MA – multiracial or mixed Americans reported as Latin, Americas -enriched for Native American, Native American, Hispanic, Hispanic American, Mexican, Mestizo from Latin America

Colunm ‘Variant’:

[1] rs2205960-T (in lD block with rs1234317) and rs1234314-C were independent in conditional regression analyses (^20^)

[2] rs1234315 and rs2205960 were independent in logistic regression analysis (^31^)

[3] rs4852324, rs940296 (correlated with rs4852324) were reported as independent from rs6705628 in gene-based association analysis (^26^) and logistic regression analysis (^28^)

[4] rs13023380, rs10930046 and rs1990760 were each independently associated with SLE in pair-wise logistic regression analysis conditioned on each SNP (^48^)

[5] SNPs rs9273076, rs114092478 ([rs3957147](http://useast.ensembl.org/homo_sapiens/Variation/Summary?v=rs3957147)), rs114090659 ([rs3132579](http://useast.ensembl.org/homo_sapiens/Variation/Summary?v=rs3132579)), rs74290525 were independently associated with SLE.The HLA-DRB1*03:01 allele wre in LD with rs1150757 and rs114092478. HLA-B*08:01 is in LD with rs1150757 and rs114090659. The HLA-DRB1*15:01 allele was tagged by rs9273076 (^5^)

[6] SNPs rs558702, rs3131379 were in strong LD with rs1269852 HLA-DRB1*03:01 associated SNP (^97^)

[7] rs1150753 (assigned to “HLA-DRB1*03:01”; “HLA-B*08:01”), rs2524117, rs9265604, rs2246618, rs9469220, rs9378200, rs8192591, rs9271731 (assigned to “HLA-DRB1*15:01) were reprted as independent loci (^95^)

[8] rs3117103 was associated with HLA-DRB1*03:01 in (^99^)

[9] rs1150754 and rs2187668 are reported as independent loci (^66^). rs2187668 is a near perfect predictor of the DRB1*03:01 allele (^68^).

[10] rs419788 was reported as independent from HLA-DQB1*03:01 in conditional analysis (^100^)

[11] rs116727542 ([rs3129895](https://useast.ensembl.org/homo_sapiens/Variation/Summary?v=rs3129895)) is accounted for by HLADQB1*06:02 and HLA-DRB1*08:03 (^32^)

[12] rs9271100 and rs3997854 were independent in conditional association analysis (^21^)

[13] rs9271366 in LD with HLA-DRB1*15:02 in Filipino and with HLA-DRB1*15:01 in Europeans (^35,98^)

[14] rs13205210 and rs3734266 were reported as independent from rs11755393 in gene-based association analysis (^26^) and in conditional logistic regression and haplotype-based association tests (^104^)

[15] rs80346167 was independent from rs117026326 and rs73366469 in conditional analysis in (^27^) but the associations of rs201802880 was considered to be primary, while the associations of rs73366469, rs117026326 and rs80346167 were thought to be secondarily caused by LD with rs201802880 in conditional logistic regression test in (^115^)

[16] rs729302 were reported as independent from rs4728142 in gene-based association analysis (^26^) and rs729302, rs10279821 and rs12537284 were 3 independent loci in logistic regression model (^67^)

[17] [rs77571059](https://www.ncbi.nlm.nih.gov/snp/rs77571059) and rs10488631 were independently associated with SLE In conditional logistic regression analysis (^123^)

[18] rs11603023 and rs10892301 were independent from rs4639966 in stepwise and conditional logistic regression analysis (^148^)

[19] rs12822507, rs10845606, and rs34330 were independent in logistic regression conditional analysis (^28^)

[20] In stepwise logistic regression analysis 3 representative SNPs of these blocks, rs2071128, rs17422 and rs1059702 showed independence and consistent significance after adjusting for the effect of other individual SNPs in different LD blocks (^172^). In conditional test using logistic regression rs5987175 was found to remain significant in Asians, after adjusting for the effect of three known independent SNPs (rs1059702, rs17422, and rs2071128). However, the independence could not be replicated in the U.K. cohort, probably owing to higher LD between the blocks in Europeans (^177^)

* For rs113164910 (6:32427005, hg19) the following alleles I>D were reported in (^27^), these alleles were described as AAC>A in (^185^); allese AC>- were shown in in NCBI LDpair Tool <https://ldlink.nci.nih.gov/> but alleles A>C were contained in dbSNP https://www.ncbi.nlm.nih.gov/snp/rs113164910

** For [rs72548051](https://www.ncbi.nlm.nih.gov/snp/rs72548051) the following alleles were mentioned as A>AC in (^23^), in dbSNP https://www.ncbi.nlm.nih.gov/snp/rs72548051 these alleles was described as AA>A

*** Included in loci based on chromosome position as the rsID and alleles are unknown (no data about alleles in publication);

? – suggested rsID and alleles or no (certain) data

# – the risk allele reported in the article (column “Allele for OR”) doesn’t match to the risk alleles reported in other publications and/or to the risk allele of risk haplotype extrapolated from variant of this loci reported in other publications (column “Risk allele”)

CN – copy number

*TE* – transposable elements (TEs) reported in (^186^) in LD with SLE-associated locus; no experiments supported that TE is functional/causal variants. TE (variant in LD; locus; overlapped Enchancer ID): ALU_umary_ALU_3575 (rs10516487; 4q24), ALU_umary_ALU_629 (rs10911628; 1q25.3; E036), ALU_umary_ALU_8901 (rs1128334; 11q24.3), ALU_umary_ALU_9012(rs12822507; 12p13.2), ALU_umary_ALU_12442 (rs131654; 22q11.21), ALU_umary_ALU_5636 (rs2230926; 6q23.3), ALU_umary_ALU_5602 (rs2327832; 6q23.3), SVA_umary_SVA_309 (rs2327832; 6q23.3), ALU_umary_ALU_6582 (rs2736340; 8p23.1), ALU_umary_ALU_11882 (rs280519; 19p13.2), ALU_umary_ALU_60 (rs4649203; 1p36.11; E099), ALU_umary_ALU_1323 (rs4852324; 2p13.1), ALU_umary_ALU_12453 (rs5754217; 22q11.21; E093, E088, E097, E080), ALU_umary_ALU_8892 (rs6590330; 11q24.3), ALU_umary_ALU_1279 (rs6705628; 2p13.1), ALU_umary_ALU_1290 (rs6705628; 2p13.1), ALU_umary_ALU_2654 (rs6804441; 3q13.33), ALU_umary_ALU_7961 (rs7097397; 10q11.23), ALU_umary_ALU_10963 (rs7200786; 16p13.13; E091, E099, E103, E096, E111), L1_umary_LINE1_2630 (rs8035957)

References:

1. Wang, Y. F. *et al.* Identification of 38 novel loci for systemic lupus erythematosus and genetic heterogeneity between ancestral groups. *Nat. Commun.* **12**, 1–13 (2021).

2. Wang, Y. F. *et al.* Identification of Shared and Asian-Specific Loci for Systemic Lupus Erythematosus and Evidence for Roles of Type III Interferon Signaling and Lysosomal Function in the Disease: A Multi-Ancestral Genome-Wide Association Study. *Arthritis Rheumatol. (Hoboken, N.J.)* **74**, 840–848 (2022).

3. Li, Y. *et al.* Association analyses identifying two common susceptibility loci shared by psoriasis and systemic lupus erythematosus in the Chinese Han population. *J. Med. Genet.* **50**, 812–818 (2013).

4. Langefeld, C. D. *et al.* Transancestral mapping and genetic load in systemic lupus erythematosus. *Nat. Commun.* **8**, (2017).

5. Bentham, J. *et al.* Genetic association analyses implicate aberrant regulation of innate and adaptive immunity genes in the pathogenesis of systemic lupus erythematosus Europe PMC Funders Group. *Nat Genet* **47**, 1457–1464 (2015).

6. Acosta-Herrera, M. *et al.* Genome-wide meta-analysis reveals shared new loci in systemic seropositive rheumatic diseases. *Ann. Rheum. Dis.* **78**, 311 (2019).

7. Namjou, B. *et al.* PTPN22 Association in Systemic Lupus Erythematosus (SLE) with Respect to Individual Ancestry and Clinical Sub-Phenotypes. *PLoS One* **8**, (2013).

8. Gateva, V. *et al.* A large-scale replication study identifies TNIP1, PRDM1, JAZF1, UHRF1BP1 and IL10 as risk loci for systemic lupus erythematosus. *Nat. Genet.* **41**, 1228–1233 (2009).

9. Julià, A. *et al.* Genome-wide association study meta-analysis identifies five new loci for systemic lupus erythematosus. *Arthritis Res. Ther.* **20**, 100 (2018).

10. Márquez, A. *et al.* A combined large-scale meta-Analysis identifies COG6 as a novel shared risk locus for rheumatoid arthritis and systemic lupus erythematosus. *Ann. Rheum. Dis.* **76**, 286–294 (2017).

11. Morris, D. L. *et al.* Genome-wide association meta-analysis in Chinese and European individuals identifies ten new loci associated with systemic lupus erythematosus. *Nat. Genet.* **48**, 940–946 (2016).

12. Hu, L. Y. *et al.* Associations between PTPN22 and TLR9 polymorphisms and systemic lupus erythematosus: a comprehensive meta-analysis. *Arch. Dermatol. Res.* **309**, 461–477 (2017).

13. Lea, W. W. & Lee, Y. H. The association between the PTPN22 C1858T polymorphism and systemic lupus erythematosus: a meta-analysis update. *Lupus* **20**, 51–7 (2011).

14. Yin, X. *et al.* Meta-analysis of 208370 East Asians identifies 113 susceptibility loci for systemic lupus erythematosus. *Ann. Rheum. Dis.* **80**, 632–640 (2021).

15. Clark, M. R., Stuart, S. G., Kimberly, R. P., Ory, P. A. & Goldstein, I. M. A single amino acid distinguishes the high-responder from the low-responder form of Fc receptor II on human monocytes. *Eur. J. Immunol.* **21**, 1911–1916 (1991).

16. Graham, R. R., Hom, G., Ortmann, W. & Behrens, T. W. Review of recent genome-wide association scans in lupus. *J. Intern. Med.* **265**, 680–688 (2009).

17. Martin, J. E. *et al.* A systemic sclerosis and systemic lupus erythematosus pan-meta-GWAS reveals new shared susceptibility loci. *Hum. Mol. Genet.* **22**, 4021–4029 (2013).

18. Lee, Y. H. *et al.* Association between FCGR3B copy number variations and susceptibility to autoimmune diseases: a meta-analysis. *Inflamm. Res.* **64**, 983–91 (2015).

19. Fanciulli, M. *et al.* FCGR3B copy number variation is associated with susceptibility to systemic, but not organ-specific, autoimmunity. *Nat. Genet.* **39**, 721–3 (2007).

20. Manku, H. *et al.* Trans-Ancestral Studies Fine Map the SLE-Susceptibility Locus TNFSF4. *PLoS Genet.* **9**, (2013).

21. Han, J. W. *et al.* Genome-wide association study in a Chinese Han population identifies nine new susceptibility loci for systemic lupus erythematosus. *Nat. Genet.* **41**, 1234–1237 (2009).

22. Lee, Y. H. & Song, G. G. Associations between TNFSF4 and TRAF1-C5 gene polymorphisms and systemic lupus erythematosus: A meta-analysis. *Hum. Immunol.* **73**, 1050–1054 (2012).

23. Akizuki, S. *et al.* PLD4 is a genetic determinant to systemic lupus erythematosus and involved in murine autoimmune phenotypes. *Ann. Rheum. Dis.* **78**, 509–518 (2019).

24. Suetsugu, H. *et al.* Novel susceptibility loci for steroid-associated osteonecrosis of the femoral head in systemic lupus erythematosus. *Hum. Mol. Genet.* **31**, 1082–1095 (2022).

25. Zuo, X. B. *et al.* Variants in TNFSF4, TNFAIP3, TNIP1, BLK, SLC15A4 and UBE2L3 interact to confer risk of systemic lupus erythematosus in Chinese population. *Rheumatol. Int.* **34**, 459–464 (2014).

26. Zhang, J. *et al.* Gene-based meta-analysis of genome-wide association study data identifies independent single-nucleotide polymorphisms in ANXA6 as being associated with systemic lupus erythematosus in Asian populations. *Arthritis Rheumatol. (Hoboken, N.J.)* **67**, 2966–77 (2015).

27. Sun, C. *et al.* High-density genotyping of immune-related loci identifies new SLE risk variants in individuals with Asian ancestry. *Nat. Genet.* **48**, 323–330 (2016).

28. Yang, W. *et al.* Meta-analysis followed by replication identifies loci in or near CDKN1B, TET3, CD80, DRAM1, and ARID5B as associated with systemic lupus erythematosus in Asians. *Am. J. Hum. Genet.* **92**, 41–51 (2013).

29. Zhang, Y. *et al.* Genome-wide search followed by replication reveals genetic interaction of CD80 and ALOX5AP associated with systemic lupus erythematosus in Asian populations. *Ann. Rheum. Dis.* **75**, 891–8 (2016).

30. Wen, L. *et al.* Exome-wide association study identifies four novel loci for systemic lupus erythematosus in Han Chinese population. *Ann. Rheum. Dis.* **77**, 417 (2018).

31. Sheng, Y. *et al.* Association analyses confirm five susceptibility loci for systemic lupus erythematosus in the Han Chinese population. *Arthritis Res. Ther.* **17**, 85 (2015).

32. Lessard, C. J. *et al.* Identification of a Systemic Lupus Erythematosus Risk Locus Spanning ATG16L2, FCHSD2, and P2RY2 in Koreans. *Arthritis Rheumatol.* **68**, 1197–1209 (2016).

33. Zhang, X. X. *et al.* PRDX6 AS1 gene polymorphisms and SLE susceptibility in Chinese populations. *Front. Immunol.* **13**, (2022).

34. Takeshima, Y. *et al.* Immune cell multiomics analysis reveals contribution of oxidative phosphorylation to B-cell functions and organ damage of lupus. *Ann. Rheum. Dis.* **81**, 845–853 (2022).

35. Alarcón-Riquelme, M. E. *et al.* Genome-Wide Association Study in an Amerindian Ancestry Population Reveals Novel Systemic Lupus Erythematosus Risk Loci and the Role of European Admixture. *Arthritis Rheumatol.* **68**, 932–943 (2016).

36. Ramos, P. S. *et al.* A comprehensive analysis of shared loci between systemic lupus erythematosus (SLE) and sixteen autoimmune diseases reveals limited genetic overlap. *PLoS Genet.* **7**, (2011).

37. Kim-Howard, X. *et al.* Allelic heterogeneity in NCF2 associated with systemic lupus erythematosus (SLE) susceptibility across four ethnic populations. *Hum. Mol. Genet.* **23**, 1656–1668 (2014).

38. Jacob, C. O. *et al.* Lupus-associated causal mutation in neutrophil cytosolic factor 2 (NCF2) brings unique insights to the structure and function of NADPH oxidase. *Proc. Natl. Acad. Sci. U. S. A.* **109**, (2012).

39. Deng, Y. *et al.* Decreased SMG7 expression associates with lupus-risk variants and elevated antinuclear antibody production. *Ann. Rheum. Dis.* **75**, 2007–2013 (2016).

40. Zhao, J., Deng, Y., Grossman, J. M. & Tsao, B. P. Fine mapping and functional study of the systemic lupus erythematosus-associated NMNAT2/SMG7 locus. *Arthritis Res. Ther.* **16**, A11 (2014).

41. Leffers, H. C. B. *et al.* Established risk loci for systemic lupus erythematosus at NCF2, STAT4, TNPO3, IRF5 and ITGAM associate with distinct clinical manifestations: A Danish genome-wide association study. *Jt. bone spine* **89**, (2022).

42. Graham, D. S. *et al.* Association of NCF2, IKZF1, IRF8, IFIH1, and TYK2 with systemic lupus erythematosus. *PLoS Genet.* **7**, (2011).

43. Armstrong, D. L. *et al.* GWAS identifies novel SLE susceptibility genes and explains the association of the HLA region. *Genes Immun.* **15**, 347–354 (2014).

44. Sakurai, D. *et al.* Preferential Binding to Elk-1 by SLE-Associated IL10 Risk Allele Upregulates IL10 Expression. *PLoS Genet.* **9**, (2013).

45. Yu, Z.-Y. *et al.* One novel susceptibility locus associate with systemic lupus erythematosus in Chinese Han population. *Rheumatol. Int.* **33**, 2079–83 (2013).

46. Lu, X. *et al.* Global discovery of lupus genetic risk variant allelic enhancer activity. *Nat. Commun.* **12**, (2021).

47. Fazel-Najafabadi, M. *et al.* Discovery and Functional Characterization of Two Regulatory Variants Underlying Lupus Susceptibility at 2p13.1. *Genes (Basel).* **13**, (2022).

48. Molineros, J. E. *et al.* Admixture mapping in lupus identifies multiple functional variants within IFIH1 associated with apoptosis, inflammation, and autoantibody production. *PLoS Genet.* **9**, e1003222 (2013).

49. Patel, Z. H. *et al.* A plausibly causal functional lupus-associated risk variant in the STAT1-STAT4 locus. **27**, 2392–2404 (2018).

50. Abelson, A.-K. *et al.* STAT4 associates with systemic lupus erythematosus through two independent effects that correlate with gene expression and act additively with IRF5 to increase risk. *Ann. Rheum. Dis.* **68**, 1746–53 (2009).

51. Raj, P. *et al.* Regulatory polymorphisms modulate the expression of HLA class II molecules and promote autoimmunity. *Elife* **5**, (2016).

52. Lee, H.-S. *et al.* Ethnic specificity of lupus-associated loci identified in a genome-wide association study in Korean women. *Ann. Rheum. Dis.* **73**, 1240–5 (2014).

53. Graham, R. R. *et al.* Genetic variants near TNFAIP3 on 6q23 are associated with systemic lupus erythematosus. *Nat. Genet.* **40**, 1059–61 (2008).

54. Zheng, J., Yin, J., Huang, R., Petersen, F. & Yu, X. Meta-analysis reveals an association of STAT4 polymorphisms with systemic autoimmune disorders and anti-dsDNA antibody. *Hum. Immunol.* **74**, 986–992 (2013).

55. Okada, Y. *et al.* A genome-wide association study identified AFF1 as a susceptibility locus for systemic lupus eyrthematosus in Japanese. *PLoS Genet.* **8**, e1002455 (2012).

56. Namjou, B. *et al.* High-density genotyping of STAT4 reveals multiple haplotypic associations with systemic lupus erythematosus in different racial groups. *Arthritis Rheum.* **60**, 1085–95 (2009).

57. Yang, W. *et al.* Genome-wide association study in asian populations identifies variants in ETS1 and WDFY4 associated with systemic lupus erythematosus. *PLoS Genet.* **6**, (2010).

58. Bolin, K. *et al.* Association of STAT4 Polymorphism with Severe Renal Insufficiency in Lupus Nephritis. *PLoS One* **8**, 84450 (2013).

59. Lee, Y. H., Bae, S.-C., Choi, S. J., Ji, J. D. & Song, G. G. Genome-wide pathway analysis of genome-wide association studies on systemic lupus erythematosus and rheumatoid arthritis. *Mol. Biol. Rep.* **39**, 10627–35 (2012).

60. Yang, W. *et al.* Population differences in SLE susceptibility genes: STAT4 and BLK, but not PXK, are associated with systemic lupus erythematosus in Hong Kong Chinese. *Genes Immun.* **10**, 219–26 (2009).

61. Kobayashi, S. *et al.* Association of STAT4 with susceptibility to rheumatoid arthritis and systemic lupus erythematosus in the Japanese population. *Arthritis Rheum.* **58**, 1940–6 (2008).

62. Alonso-Perez, E. *et al.* Further evidence of subphenotype association with systemic lupus erythematosus susceptibility loci: a European cases only study. *PLoS One* **7**, e45356 (2012).

63. Remmers, E. F. *et al.* STAT4 and the risk of rheumatoid arthritis and systemic lupus erythematosus. *N. Engl. J. Med.* **357**, 977–86 (2007).

64. Suarez-Gestal, M. *et al.* Replication of recently identified systemic lupus erythematosus genetic associations: a case-control study. *Arthritis Res. Ther.* **11**, R69 (2009).

65. Taylor, K. E. *et al.* Specificity of the STAT4 genetic association for severe disease manifestations of systemic lupus erythematosus. *PLoS Genet.* **4**, e1000084 (2008).

66. Chung, S. A. *et al.* Differential genetic associations for systemic lupus erythematosus based on anti-dsDNA autoantibody production. *PLoS Genet.* **7**, e1001323 (2011).

67. Harley, J. B. *et al.* Genome-wide association scan in women with systemic lupus erythematosus identifies susceptibility variants in ITGAM, PXK, KIAA1542 and other loci. *Nat. Genet.* **40**, 204–210 (2008).

68. Hom, G. *et al.* Association of systemic lupus erythematosus with C8orf13-BLK and ITGAM-ITGAX. *N. Engl. J. Med.* **358**, 900–9 (2008).

69. Taylor, K. E. *et al.* Risk alleles for systemic lupus erythematosus in a large case-control collection and associations with clinical subphenotypes. *PLoS Genet.* **7**, e1001311 (2011).

70. Tangtanatakul, P. *et al.* Meta-analysis of genome-wide association study identifies FBN2 as a novel locus associated with systemic lupus erythematosus in Thai population. *Arthritis Res. Ther.* **22**, (2020).

71. Qi, Y. yuan *et al.* Lupus susceptibility region containing CTLA4 rs17268364 functionally reduces CTLA4 expression by binding EWSR1 and correlates IFN-α signature. *Arthritis Res. Ther.* **23**, (2021).

72. Elghzaly, A. A. *et al.* Genome-wide association study for systemic lupus erythematosus in an egyptian population. *Front. Genet.* **13**, (2022).

73. Oparina, N. Y. *et al.* PXK locus in systemic lupus erythematosus: fine mapping and functional analysis reveals novel susceptibility gene ABHD6. *Ann. Rheum. Dis.* **74**, (2015).

74. Vaughn, S. E. *et al.* Lupus risk variants in the PXK locus alter B-cell receptor internalization. *Front. Genet.* **5**, 450 (2014).

75. Lessard, C. J. *et al.* Identification of IRF8, TMEM39A, and IKZF3-ZPBP2 as susceptibility loci for systemic lupus erythematosus in a large-scale multiracial replication study. *Am. J. Hum. Genet.* **90**, 648–60 (2012).

76. Molineros, J. E. *et al.* Confirmation of five novel susceptibility loci for systemic lupus erythematosus (SLE) and integrated network analysis of 82 SLE susceptibility loci. *Hum. Mol. Genet.* **26**, 1205–1216 (2017).

77. Liu, L. *et al.* Genome-wide association study identifies three novel susceptibility loci for systemic lupus erythematosus in Han Chinese. *Br. J. Dermatol.* **179**, 506–508 (2018).

78. Martínez-Bueno, M. *et al.* Trans-Ethnic Mapping of BANK1 Identifies Two Independent SLE-Risk Linkage Groups Enriched for Co-Transcriptional Splicing Marks. *Int. J. Mol. Sci.* **19**, (2018).

79. Kozyrev, S. V *et al.* Functional variants in the B-cell gene BANK1 are associated with systemic lupus erythematosus. *Nat. Genet.* **40**, 211–6 (2008).

80. Fan, Y., Tao, J.-H., Zhang, L.-P., Li, L.-H. & Ye, D.-Q. The association between BANK1 and TNFAIP3 gene polymorphisms and systemic lupus erythematosus: a meta-analysis. *Int. J. Immunogenet.* **38**, 151–9 (2011).

81. Chang, Y. K. *et al.* Association of BANK1 and TNFSF4 with systemic lupus erythematosus in Hong Kong Chinese. *Genes Immun.* **10**, 414–20 (2009).

82. Bae, S.-C. & Lee, Y. H. Association between BANK1 polymorphisms and susceptibility to autoimmune diseases: A meta-analysis. *Cell. Mol. Biol. (Noisy-le-grand).* **63**, 29–35 (2017).

83. Hughes, T. *et al.* Fine-mapping and transethnic genotyping establish IL2/IL21 genetic association with lupus and localize this genetic effect to IL21. *Arthritis Rheum.* **63**, 1689–97 (2011).

84. Song, Q. *et al.* Genome-wide association study on Northern Chinese identifies KLF2 , DOT1L and STAB2 associated with systemic lupus erythematosus . *Rheumatology* (2021) doi:10.1093/rheumatology/keab016.

85. Wen, L. L. *et al.* Multiple variants in 5q31.1 are associated with systemic lupus erythematosus susceptibility and subphenotypes in the Han Chinese population. *Br. J. Dermatol.* **177**, 801–808 (2017).

86. Zhang, Y.-M. *et al.* Evaluation of 10 SLE susceptibility loci in Asian populations, which were initially identified in European populations. *Sci. Rep.* **7**, 41399 (2017).

87. Adrianto, I. *et al.* Association of two independent functional risk haplotypes in TNIP1 with systemic lupus erythematosus. *Arthritis Rheum.* **64**, 3695–705 (2012).

88. Caster, D. J. *et al.* ABIN1 dysfunction as a genetic basis for lupus nephritis. *J. Am. Soc. Nephrol.* **24**, 1743–1754 (2013).

89. Wang, C. *et al.* Genes identified in Asian SLE GWASs are also associated with SLE in Caucasian populations. *Eur. J. Hum. Genet.* **21**, 994–9 (2013).

90. Luo, X. *et al.* A functional variant in microRNA-146a promoter modulates its expression and confers disease risk for systemic lupus erythematosus. *PLoS Genet.* **7**, e1002128 (2011).

91. Kunz, M. *et al.* Genome-wide association study identifies new susceptibility loci for cutaneous lupus erythematosus. *Exp. Dermatol.* **24**, 510–5 (2015).

92. Clancy, R. M. *et al.* Identification of candidate loci at 6p21 and 21q22 in a genome-wide association study of cardiac manifestations of neonatal lupus. *Arthritis Rheum.* **62**, 3415–3424 (2010).

93. Sánchez, E. *et al.* Identification of novel genetic susceptibility loci in African American lupus patients in a candidate gene association study. *Arthritis Rheum.* **63**, 3493–501 (2011).

94. Ramos, P. S. *et al.* Genetic analyses of interferon pathway-related genes reveal multiple new loci associated with systemic lupus erythematosus. *Arthritis Rheum.* **63**, 2049–2057 (2011).

95. Morris, D. L. *et al.* Unraveling multiple MHC gene associations with systemic lupus erythematosus: model choice indicates a role for HLA alleles and non-HLA genes in Europeans. *Am. J. Hum. Genet.* **91**, 778–93 (2012).

96. Demirci, F. Y. *et al.* Identification of a New Susceptibility Locus for Systemic Lupus Erythematosus on Chromosome 12 in Individuals of European Ancestry. *Arthritis Rheumatol. (Hoboken, N.J.)* **68**, 174–83 (2016).

97. International MHC and Autoimmunity Genetics Network *et al.* Mapping of multiple susceptibility variants within the MHC region for 7 immune-mediated diseases. *Proc. Natl. Acad. Sci. U. S. A.* **106**, 18680–5 (2009).

98. Fernando, M. M. A. *et al.* Transancestral mapping of the MHC region in systemic lupus erythematosus identifies new independent and interacting loci at MSH5, HLA-DPB1 and HLA-G. *Ann. Rheum. Dis.* **71**, 777–84 (2012).

99. Barcellos, L. F. *et al.* High-density SNP screening of the major histocompatibility complex in systemic lupus erythematosus demonstrates strong evidence for independent susceptibility regions. *PLoS Genet.* **5**, e1000696 (2009).

100. Fernando, M. M. A. *et al.* Identification of two independent risk factors for lupus within the MHC in United Kingdom families. *PLoS Genet.* **3**, e192 (2007).

101. Sun, J. *et al.* HLA-DQβ1 amino acid position 87 and DQB1*0301 are associated with Chinese Han SLE. *Mol. Genet. Genomic Med.* **6**, 541–546 (2018).

102. Oishi, T. *et al.* A functional SNP in the NKX2.5-binding site of ITPR3 promoter is associated with susceptibility to systemic lupus erythematosus in Japanese population. *J. Hum. Genet.* **53**, 151–62 (2008).

103. Wen, L. *et al.* The association of the UHRF1BP1 gene with systemic lupus erythematosus was replicated in a Han Chinese population from mainland China. *Ann. Hum. Genet.* (2019) doi:10.1111/ahg.12362.

104. Zhang, Y. *et al.* Two missense variants in UHRF1BP1 are independently associated with systemic lupus erythematosus in Hong Kong Chinese. *Genes Immun.* **12**, 231–4 (2011).

105. Zhou, X. *et al.* Genetic association of PRDM1-ATG5 intergenic region and autophagy with systemic lupus erythematosus in a Chinese population. *Ann. Rheum. Dis.* **70**, 1330–7 (2011).

106. Adrianto, I. *et al.* *Association of a functional variant downstream of TNFAIP3 with systemic lupus erythematosus*. vol. 43 253–258 (Nat Genet, 2011).

107. Zhang, M.-Y., Yang, X.-K., Pan, H.-F. & Ye, D.-Q. Associations between TNFAIP3 gene polymorphisms and systemic lupus erythematosus risk: an updated meta-analysis. *HLA* **88**, 245–252 (2016).

108. Bates, J. S. *et al.* Meta-analysis and imputation identifies a 109 kb risk haplotype spanning TNFAIP3 associated with lupus nephritis and hematologic manifestations. *Genes Immun.* **10**, 470–7 (2009).

109. Shimane, K. *et al.* The association of a nonsynonymous single-nucleotide polymorphism in TNFAIP3 with systemic lupus erythematosus and rheumatoid arthritis in the Japanese population. *Arthritis Rheum.* **62**, 574–9 (2010).

110. Cai, L. Q. *et al.* A single-nucleotide polymorphism of the TNFAIP3 gene is associated with systemic lupus erythematosus in Chinese Han population. *Mol. Biol. Rep.* **37**, 389–394 (2010).

111. Westra, H. J. *et al.* Systematic identification of trans eQTLs as putative drivers of known disease associations. *Nat. Genet.* **45**, 1238–1243 (2013).

112. Vyse, T. J. & Cunninghame Graham, D. S. Trans-Ancestral Fine-Mapping and Epigenetic Annotation as Tools to Delineate Functionally Relevant Risk Alleles at IKZF1 and IKZF3 in Systemic Lupus Erythematosus. *Int. J. Mol. Sci.* **21**, (2020).

113. Wang, Y.-F. *et al.* Identification of ST3AGL4, MFHAS1, CSNK2A2 and CD226 as loci associated with systemic lupus erythematosus (SLE) and evaluation of SLE genetics in drug repositioning. *Ann. Rheum. Dis.* **77**, 1078–1084 (2018).

114. Li, Y. *et al.* Association of GTF2I and GTF2IRD1 polymorphisms with systemic lupus erythematosus in a Chinese Han population. *Clin. Exp. Rheumatol.* **33**, 632–8.

115. Yokoyama, N. *et al.* Association of NCF1 polymorphism with systemic lupus erythematosus and systemic sclerosis but not with ANCA-associated vasculitis in a Japanese population. *Sci. Rep.* **9**, 16366 (2019).

116. Zhao, J. *et al.* A missense variant in NCF1 is associated with susceptibility to multiple autoimmune diseases. *Nat. Genet.* **49**, 433–437 (2017).

117. Olsson, L. M. *et al.* A single nucleotide polymorphism in the NCF1 gene leading to reduced oxidative burst is associated with systemic lupus erythematosus. *Ann. Rheum. Dis.* **76**, 1607–1613 (2017).

118. Meng, Y. *et al.* The NCF1 variant p.R90H aggravates autoimmunity by facilitating the activation of plasmacytoid dendritic cells. *J. Clin. Invest.* **132**, (2022).

119. Alonso-Perez, E. *et al.* Identification of three new cis-regulatory IRF5 polymorphisms: in vitro studies. *Arthritis Res. Ther.* **15**, (2013).

120. Alonso-Perez, E. *et al.* Cis-regulation of IRF5 expression is unable to fully account for systemic lupus erythematosus association: Analysis of multiple experiments with lymphoblastoid cell lines. *Arthritis Res. Ther.* **13**, (2011).

121. Armstrong, D. L. *et al.* Identification of new SLE-associated genes with a two-step Bayesian study design. *Genes Immun.* **10**, 446–56 (2009).

122. Graham, R. R. *et al.* A common haplotype of interferon regulatory factor 5 (IRF5) regulates splicing and expression and is associated with increased risk of systemic lupus erythematosus. *Nat. Genet.* **38**, 550–5 (2006).

123. Sigurdsson, S. *et al.* Comprehensive evaluation of the genetic variants of interferon regulatory factor 5 (IRF5) reveals a novel 5 bp length polymorphism as strong risk factor for systemic lupus erythematosus. *Hum. Mol. Genet.* **17**, 872–81 (2008).

124. Kristjansdottir, G. *et al.* Interferon regulatory factor 5 (IRF5) gene variants are associated with multiple sclerosis in three distinct populations. *J. Med. Genet.* **45**, 362–369 (2008).

125. Kottyan, L. C. *et al.* The IRF5-TNPO3 association with systemic lupus erythematosus has two components that other autoimmune disorders variably share. **24**, 582–596 (2015).

126. Kawasaki, A. *et al.* Association of IRF5 polymorphisms with systemic lupus erythematosus in a Japanese population: Support for a crucial role of intron 1 polymorphisms. *Arthritis Rheum.* **58**, 826–834 (2008).

127. Graham, D. S. C. *et al.* Association of IRF5 in UK SLE families identifies a variant involved in polyadenylation. *Hum. Mol. Genet.* **16**, 579–591 (2007).

128. Martin, M. V. *et al.* Exon expression in lymphoblastoid cell lines from subjects with schizophrenia before and after glucose deprivation. *BMC Med. Genomics* **2**, (2009).

129. Kozyrev, S. V. *et al.* Structural insertion/deletion variation in IRF5 is associated with a risk haplotype and defines the precise IRF5 isoforms expressed in systemic lupus erythematosus. *Arthritis Rheum.* **56**, 1234–1241 (2007).

130. Löfgren, S. E. *et al.* Promoter insertion/deletion in the IRF5 gene is highly associated with susceptibility to systemic lupus erythematosus in distinct populations, but exerts a modest effect on gene expression in peripheral blood mononuclear cells. **37**, 574–577 (2010).

131. Feng, D. *et al.* Genetic variants and disease-associated factors contribute to enhanced interferon regulatory factor 5 expression in blood cells of patients with systemic lupus erythematosus. *Arthritis Rheum.* **62**, (2010).

132. Rullo, O. J. *et al.* Association of IRF5 polymorphisms with activation of the interferon alpha pathway. *Ann. Rheum. Dis.* **69**, 611–617 (2010).

133. Ito, I. *et al.* Association of a functional polymorphism in the IRF5 region with systemic sclerosis in a Japanese population. *Arthritis Rheum.* **60**, 1845–1850 (2009).

134. Nordang, G. B. N. *et al.* Interferon regulatory factor 5 gene polymorphism confers risk to several rheumatic diseases and correlates with expression of alternative thymic transcripts. **51**, 619–626 (2012).

135. Shin, H. D. *et al.* Replication of the genetic effects of IFN regulatory factor 5 (IRF5) on systemic lupus erythematosus in a Korean population. **9**, (2007).

136. Reddy, M. V. P. L. *et al.* Genetic association of IRF5 with SLE in Mexicans: Higher frequency of the risk haplotype and its homozygozity than Europeans. *Hum. Genet.* **121**, 721–727 (2007).

137. Kelly, J. A. *et al.* Interferon regulatory factor-5 is genetically associated with systemic lupus erythematosus in African Americans. **9**, 187–194 (2008).

138. Zervou, M. I. *et al.* Association of IRF5 polymorphisms with increased risk for systemic lupus erythematosus in population of Crete, a southern-eastern European Greek island. *Gene* **610**, 9–14 (2017).

139. Sanchez, E. *et al.* Genetically determined Amerindian ancestry correlates with increased frequency of risk alleles for systemic lupus erythematosus. *Arthritis Rheum.* **62**, 3722–9 (2010).

140. Ferreiro-Neira, I. *et al.* Opposed independent effects and epistasis in the complex association of IRF5 to SLE. *Genes Immun.* **8**, 429–38 (2007).

141. Demirci, F. Y. *et al.* Multiple signals at the extended 8p23 locus are associated with susceptibility to systemic lupus erythematosus. *J. Med. Genet.* **54**, 381–389 (2017).

142. Guthridge, J. M. *et al.* Two functional lupus-associated BLK promoter variants control cell-type- and developmental-stage-specific transcription. **94**, 586–598 (2014).

143. Zhang, Z. *et al.* The association of the BLK gene with SLE was replicated in Chinese Han. *Arch. Dermatol. Res.* **302**, 619–24 (2010).

144. Song, G. G. & Lee, Y. H. Association between BLK polymorphisms and susceptibility to SLE : A meta-analysis. *Z. Rheumatol.* **76**, 176–182 (2017).

145. Fan, Z. *et al.* Association of the Polymorphism rs13259960 in SLEAR With Predisposition to Systemic Lupus Erythematosus. **72**, 985–996 (2020).

146. Zhao, H. *et al.* An intronic variant associated with systemic lupus erythematosus changes the binding affinity of Yinyang1 to downregulate WDFY4. *Genes Immun.* **13**, 536–542 (2012).

147. Lessard, C. J. *et al.* Identification of a systemic lupus erythematosus susceptibility locus at 11p13 between PDHX and CD44 in a multiethnic study. *Am. J. Hum. Genet.* **88**, 83–91 (2011).

148. Zhang, J. *et al.* Three SNPs in chromosome 11q23.3 are independently associated with systemic lupus erythematosus in Asians. *Hum. Mol. Genet.* **23**, 524–33 (2014).

149. Singh, B. *et al.* Lupus susceptibility region containing CDKN1B rs34330 mechanistically influences expression and function of multiple target genes, also linked to proliferation and apoptosis . *Arthritis Rheumatol.* (2021) doi:10.1002/art.41799.

150. Yang, J. *et al.* ELF1 is associated with systemic lupus erythematosus in Asian populations. *Hum. Mol. Genet.* **20**, 601–607 (2011).

151. Molineros, J. E. *et al.* Mechanistic Characterization of RASGRP1 Variants Identifies an hnRNP-K-Regulated Transcriptional Enhancer Contributing to SLE Susceptibility. *Front. Immunol.* **10**, (2019).

152. Manjarrez-Orduño, N. *et al.* CSK regulatory polymorphism is associated with systemic lupus erythematosus and influences B-cell signaling and activation. *Nat. Genet.* **44**, 1227–30 (2012).

153. Zhang, Z. *et al.* Polymorphisms at 16p13 are associated with systemic lupus erythematosus in the Chinese population. *J. Med. Genet.* **48**, 69–72 (2011).

154. Sheng, Y.-J. *et al.* Follow-up study identifies two novel susceptibility loci PRKCB and 8p11.21 for systemic lupus erythematosus. *Rheumatology (Oxford).* **50**, 682–8 (2011).

155. Maiti, A. K. *et al.* Combined protein-and nucleic acid-level effects of rs1143679 (R77H), a lupus-predisposing variant within ITGAM. doi:10.1093/hmg/ddu106.

156. Rhodes, B. *et al.* The rs1143679 (R77H) lupus associated variant of ITGAM (CD11b) impairs complement receptor 3 mediated functions in human monocytes. *Ann. Rheum. Dis.* **71**, 2028–2034 (2012).

157. MacPherson, M., Lek, H. S., Prescott, A. & Fagerholm, S. C. A systemic lupus erythematosus-associated R77H substitution in the CD11b chain of the Mac-1 integrin compromises leukocyte adhesion and phagocytosis. *J. Biol. Chem.* **286**, 17303–17310 (2011).

158. Nath, S. K. *et al.* A nonsynonymous functional variant in integrin-alpha(M) (encoded by ITGAM) is associated with systemic lupus erythematosus. *Nat. Genet.* **40**, 152–4 (2008).

159. Kim, K. *et al.* Variation in the ICAM1-ICAM4-ICAM5 locus is associated with systemic lupus erythematosus susceptibility in multiple ancestries. *Ann. Rheum. Dis.* **71**, 1809–14 (2012).

160. Järvinen, T. M. *et al.* Polymorphisms of the ITGAM gene confer higher risk of discoid cutaneous than of systemic lupus erythematosus. *PLoS One* **5**, e14212 (2010).

161. Kim-Howard, X. *et al.* ITGAM coding variant (rs1143679) influences the risk of renal disease, discoid rash and immunological manifestations in patients with systemic lupus erythematosus with European ancestry. *Ann. Rheum. Dis.* **69**, 1329–32 (2010).

162. Han, S. *et al.* Evaluation of imputation-based association in and around the integrin-α-M (ITGAM) gene and replication of robust association between a non-synonymous functional variant within ITGAM and systemic lupus erythematosus (SLE). *Hum. Mol. Genet.* **18**, 1171–1180 (2009).

163. Lee, Y. H. & Bae, S.-C. Association between the functional ITGAM rs1143679 G/A polymorphism and systemic lupus erythematosus/lupus nephritis or rheumatoid arthritis: an update meta-analysis. *Rheumatol. Int.* **35**, 815–23 (2015).

164. Chrabot, B. S. *et al.* Genetic variation near IRF8 is associated with serologic and cytokine profiles in systemic lupus erythematosus and multiple sclerosis. *Genes Immun.* **14**, 471–478 (2013).

165. Qi, Y. Y. *et al.* A rare variant (rs933717) at FBXO31-MAP1LC3B in Chinese is associated with systemic lupus erythematosus. *Arthritis Rheumatol. (Hoboken, N.J.)* **70**, 287 (2018).

166. Lee, Y. H. & Bae, S.-C. Association between TYK2 polymorphisms and susceptibility to autoimmune rheumatic diseases: a meta-analysis. *Lupus* **25**, 1307–14 (2016).

167. Sigurdsson, S. *et al.* Polymorphisms in the tyrosine kinase 2 and interferon regulatory factor 5 genes are associated with systemic lupus erythematosus. *Am. J. Hum. Genet.* **76**, 528–37 (2005).

168. Zhang, F. *et al.* Independent Replication on Genome-Wide Association Study Signals Identifies IRF3 as a Novel Locus for Systemic Lupus Erythematosus. *Front. Genet.* **11**, (2020).

169. Vazgiourakis, V. M. *et al.* A common SNP in the CD40 region is associated with systemic lupus erythematosus and correlates with altered CD40 expression: implications for the pathogenesis. *Ann. Rheum. Dis.* **70**, 2184–90 (2011).

170. Lewis, M. J. *et al.* UBE2L3 Polymorphism Amplifies NF-κB Activation and Promotes Plasma Cell Development, Linking Linear Ubiquitination to Multiple Autoimmune Diseases. *Am. J. Hum. Genet.* **96**, 221 (2015).

171. Zhang, Y. *et al.* Meta-analysis of two Chinese populations identifies an autoimmune disease risk allele in 22q11.21 as associated with systemic lupus erythematosus. *Arthritis Res. Ther.* **17**, 67 (2015).

172. Zhang, Y. *et al.* Meta-analysis of GWAS on two Chinese populations followed by replication identifies novel genetic variants on the X chromosome associated with systemic lupus erythematosus. *Hum. Mol. Genet.* **24**, 274–84 (2015).

173. Deng, Y. *et al.* MicroRNA-3148 modulates allelic expression of toll-like receptor 7 variant associated with systemic lupus erythematosus. *PLoS Genet.* **9**, e1003336 (2013).

174. Shen, N. *et al.* Sex-specific association of X-linked toll-like receptor 7 (TLR7) with male systemic lupus erythematosus. *Proc. Natl. Acad. Sci. U. S. A.* **107**, 15838–15843 (2010).

175. Lee, Y. H., Choi, S. J., Ji, J. D. & Song, G. G. Association between toll-like receptor polymorphisms and systemic lupus erythematosus: a meta-analysis update. *Lupus* **25**, 593–601 (2016).

176. Lee, Y. H., Lee, H.-S., Choi, S. J., Ji, J. D. & Song, G. G. Associations between TLR polymorphisms and systemic lupus erythematosus: a systematic review and meta-analysis. *Clin. Exp. Rheumatol.* **30**, 262–5.

177. Zhang, H. *et al.* Meta-analysis of GWAS on both Chinese and European populations identifies GPR173 as a novel X chromosome susceptibility gene for SLE. *Arthritis Res. Ther.* **20**, 92 (2018).

178. Zhu, Z. *et al.* Discovery of a novel genetic susceptibility locus on X chromosome for systemic lupus erythematosus. *Arthritis Res. Ther.* **17**, 349 (2015).

179. Liu, G., Tsuruta, Y., Gao, Z., Park, Y.-J. & Abraham, E. Variant IL-1 receptor-associated kinase-1 mediates increased NF-kappa B activity. *J. Immunol.* **179**, 4125–4134 (2007).

180. Kaufman, K. M. *et al.* Fine mapping of Xq28: both MECP2 and IRAK1 contribute to risk for systemic lupus erythematosus in multiple ancestral groups. *Ann. Rheum. Dis.* **72**, 437–44 (2013).

181. Jacob, C. O. *et al.* Identification of IRAK1 as a risk gene with critical role in the pathogenesis of systemic lupus erythematosus. *Proc. Natl. Acad. Sci. U. S. A.* **106**, 6256–61 (2009).

182. Sawalha, A. H. *et al.* Common variants within MECP2 confer risk of systemic lupus erythematosus. *PLoS One* **3**, e1727 (2008).

183. Webb, R. *et al.* Variants within MECP2, a key transcription regulator, are associated with increased susceptibility to lupus and differential gene expression in patients with systemic lupus erythematosus. *Arthritis Rheum.* **60**, 1076–84 (2009).

184. Pers, T. H. *et al.* Biological interpretation of genome-wide association studies using predicted gene functions. *Nat. Commun. 2015 61* **6**, 1–9 (2015).

185. Iotchkova, V. *et al.* Discovery and refinement of genetic loci associated with cardiometabolic risk using dense imputation maps. *Nat. Genet.* **48**, 1303–1312 (2016).

186. Wang, L., Norris, E. T. & Jordan, I. K. Human retrotransposon insertion polymorphisms are associated with health and disease via gene regulatory phenotypes. *Front. Microbiol.* **8**, (2017).
